# Supplementary material for: Genotyping-by-sequencing enables linkage mapping in three octoploid cultivated strawberry families
Source: PeerJ. 2017 Aug 30;5:e3731. doi: 10.7717/peerj.3731 (PMC5581533; doi:10.7717/peerj.3731)

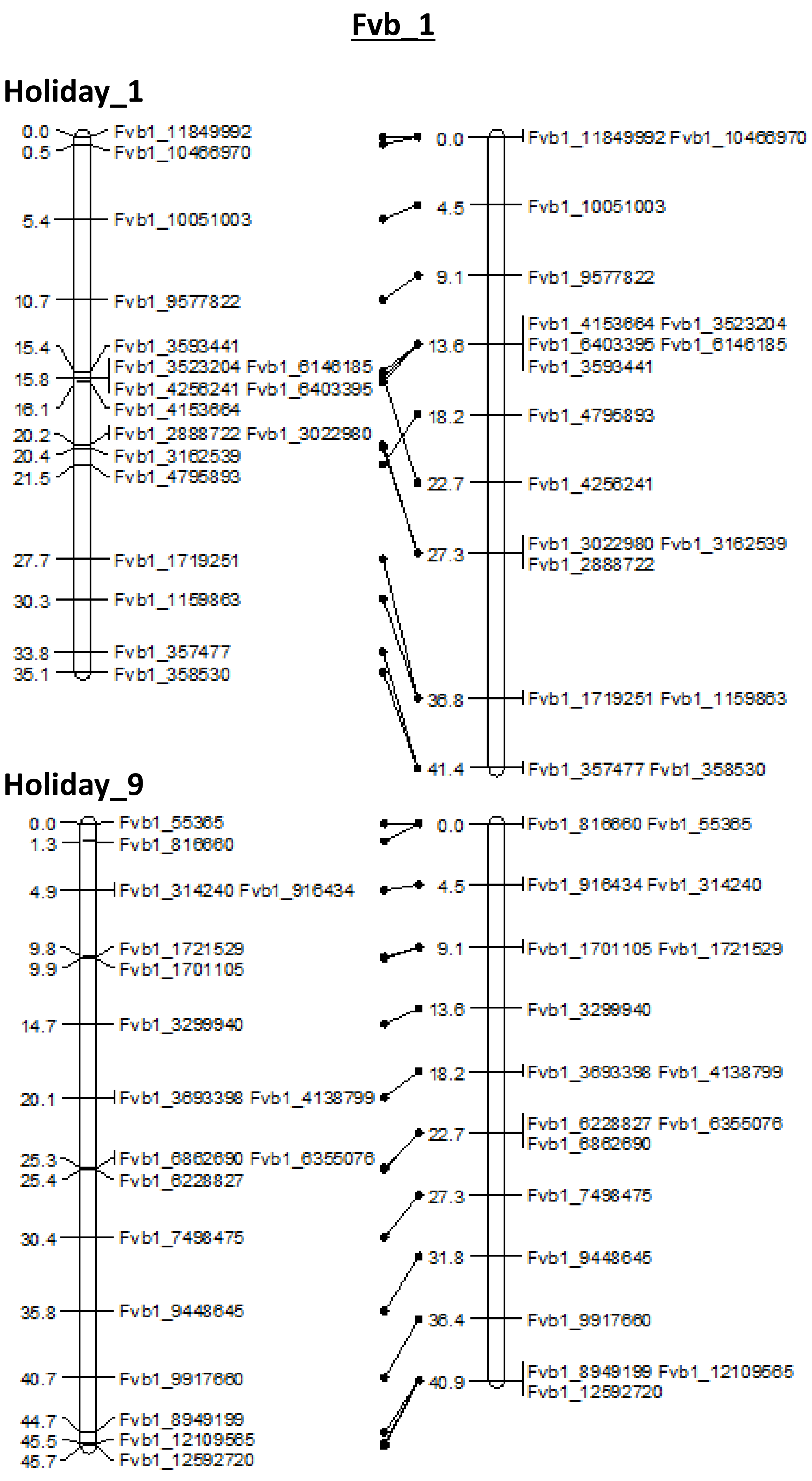
Fvb_1
Holiday_1
0.0 Fvb1_11849992
0.5 Fvb1_10466970
5.4 Fvb1_10051003
10.7 Fvb1_9577822
15.4 Fvb1_3593441
15.8 Fvb1_3523204 Fvb1_6146185
Fvb1_4256241 Fvb1_6403395
16.1 Fvb1_4153664
20.2 Fvb1_2888722 Fvb1_3022980
20.4 Fvb1_3162539
21.5 Fvb1_4795893
27.7 Fvb1_1719251
30.3 Fvb1_1159863
33.8 Fvb1_357477
35.1 Fvb1_358530
0.0 Fvb1_11849992 Fvb1_10466970
4.5 Fvb1_10051003
9.1 Fvb1_9577822
13.6 Fvb1_4153664 Fvb1_3523204
Fvb1_6403395 Fvb1_6146185
Fvb1_3593441
18.2 Fvb1_4795893
22.7 Fvb1_4256241
27.3 Fvb1_3022980 Fvb1_3162539
Fvb1_2888722
36.8 Fvb1_1719251 Fvb1_1159863
41.4 Fvb1_357477 Fvb1_358530
Holiday_9
0.0 Fvb1_55365
1.3 Fvb1_816660
4.9 Fvb1_314240 Fvb1_916434
9.8 Fvb1_1721529
9.9 Fvb1_1701105
14.7 Fvb1_3299940
20.1 Fvb1_3693398 Fvb1_4138799
25.3 Fvb1_6862690 Fvb1_6355076
25.4 Fvb1_6228827
30.4 Fvb1_7498475
35.8 Fvb1_9448645
40.7 Fvb1_9917660
44.7 Fvb1_8949199
45.5 Fvb1_12109565
45.7 Fvb1_12592720
0.0 Fvb1_816660 Fvb1_55365
4.5 Fvb1_916434 Fvb1_314240
9.1 Fvb1_1701105 Fvb1_1721529
13.6 Fvb1_3299940
18.2 Fvb1_3693398 Fvb1_4138799
22.7 Fvb1_6228827 Fvb1_6355076
Fvb1_6862690
27.3 Fvb1_7498475
31.8 Fvb1_9448645
36.4 Fvb1_9917660
40.9 Fvb1_8949199 Fvb1_12109565
Fvb1_12592720

Fvb_1

Holiday_25

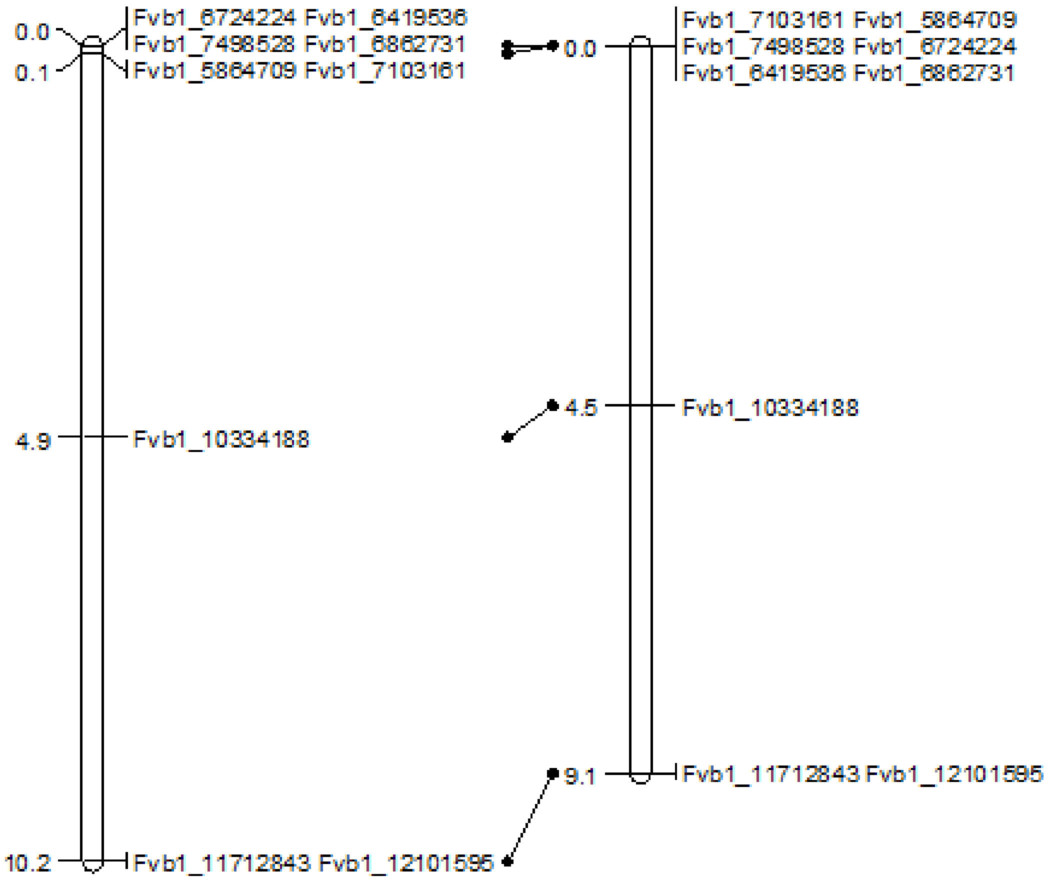

Holiday_33

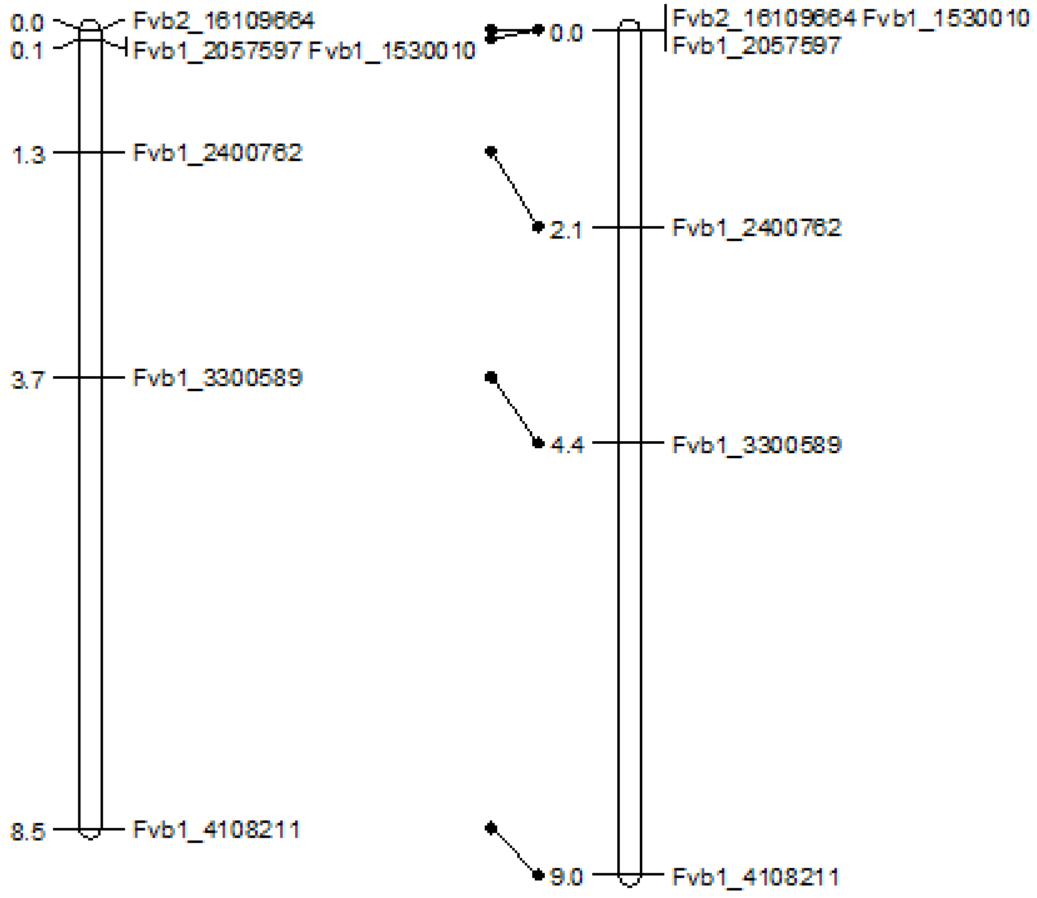

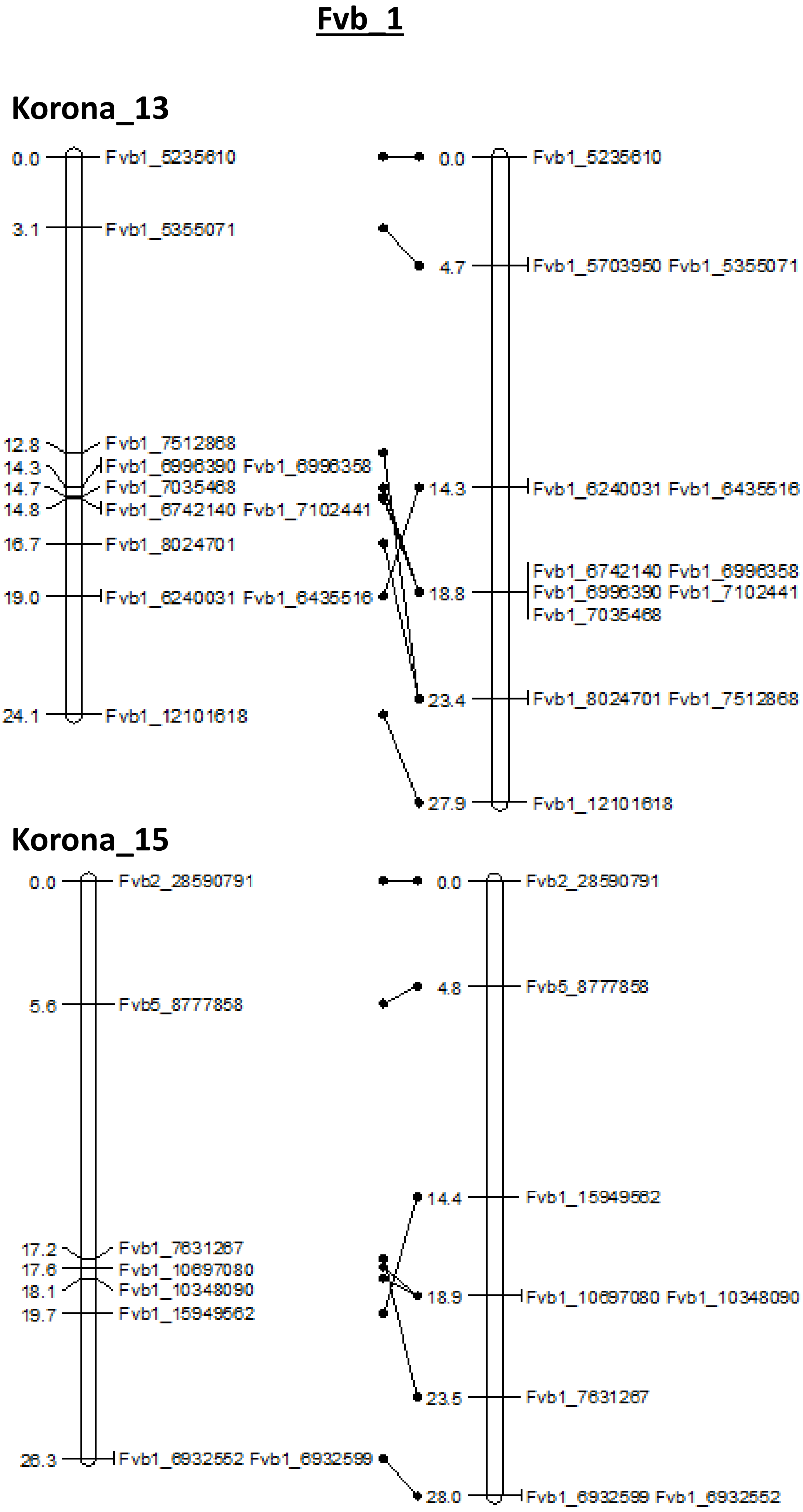
Fvb_1
Korona_13
0.0 Fvb1_5235610
3.1 Fvb1_5355071
12.8 Fvb1_7512868
14.3 Fvb1_6996390 Fvb1_6996358
14.7 Fvb1_7035468
14.8 Fvb1_6742140 Fvb1_7102441
16.7 Fvb1_8024701
19.0 Fvb1_6240031 Fvb1_6435516
24.1 Fvb1_12101618
0.0 Fvb1_5235610
4.7 Fvb1_5703950 Fvb1_5355071
14.3 Fvb1_6240031 Fvb1_6435516
18.8 Fvb1_6742140 Fvb1_6996358 Fvb1_6996390 Fvb1_7102441 Fvb1_7035468
23.4 Fvb1_8024701 Fvb1_7512868
27.9 Fvb1_12101618
Korona_15
0.0 Fvb2_28590791
5.6 Fvb5_8777858
17.2 Fvb1_7631267
17.6 Fvb1_10697080
18.1 Fvb1_10348090
19.7 Fvb1_15949562
26.3 Fvb1_6932552 Fvb1_6932599
0.0 Fvb2_28590791
4.8 Fvb5_8777858
14.4 Fvb1_15949562
18.9 Fvb1_10697080 Fvb1_10348090
23.5 Fvb1_7631267
28.0 Fvb1_6932599 Fvb1_6932552

Fvb_1

Korona_16

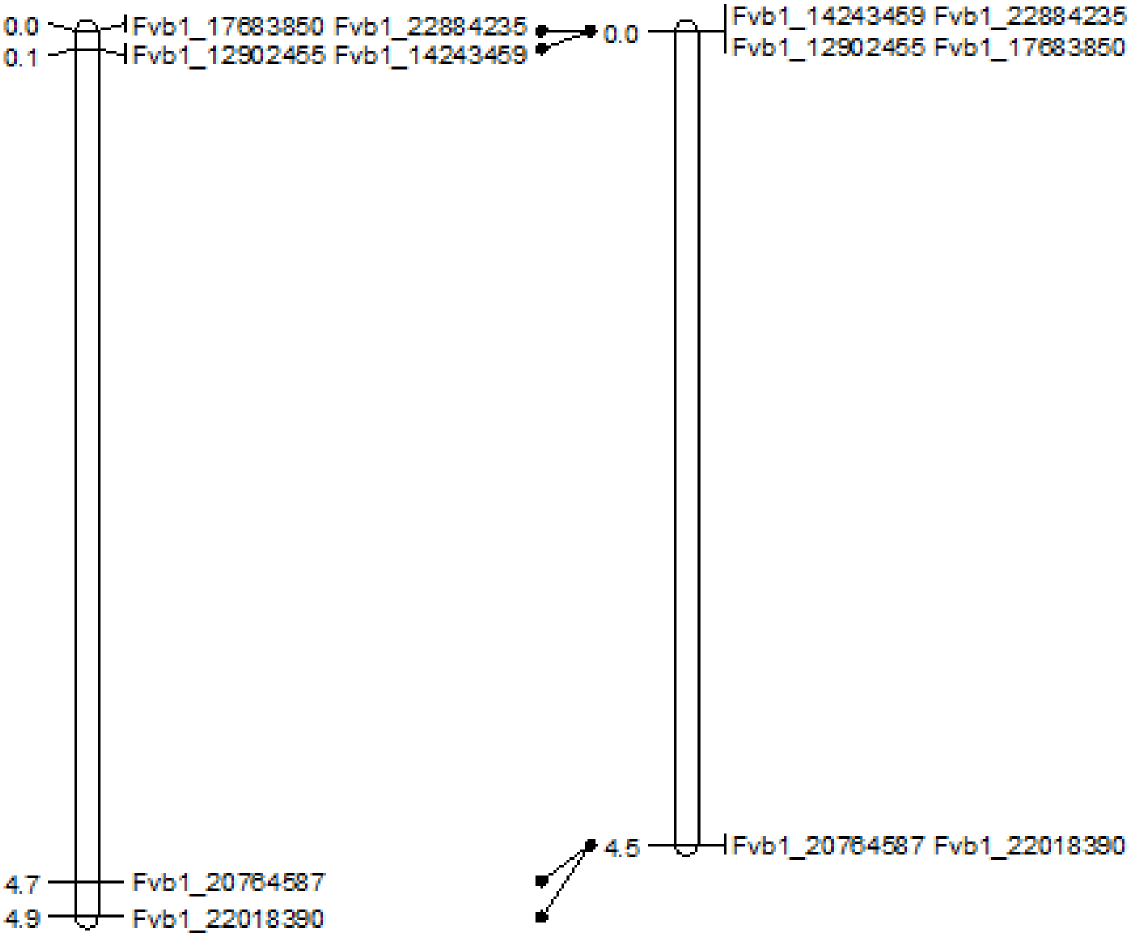

Korona_32

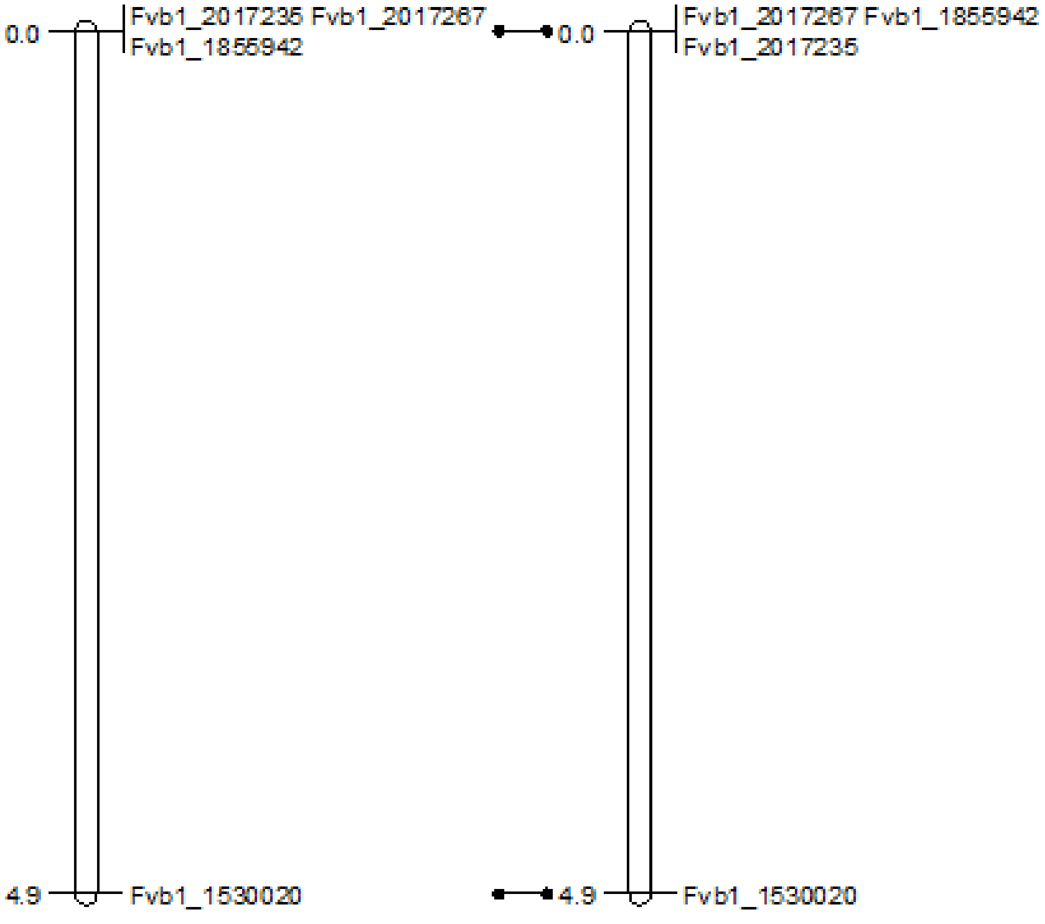

Fvb_2

Holiday_30

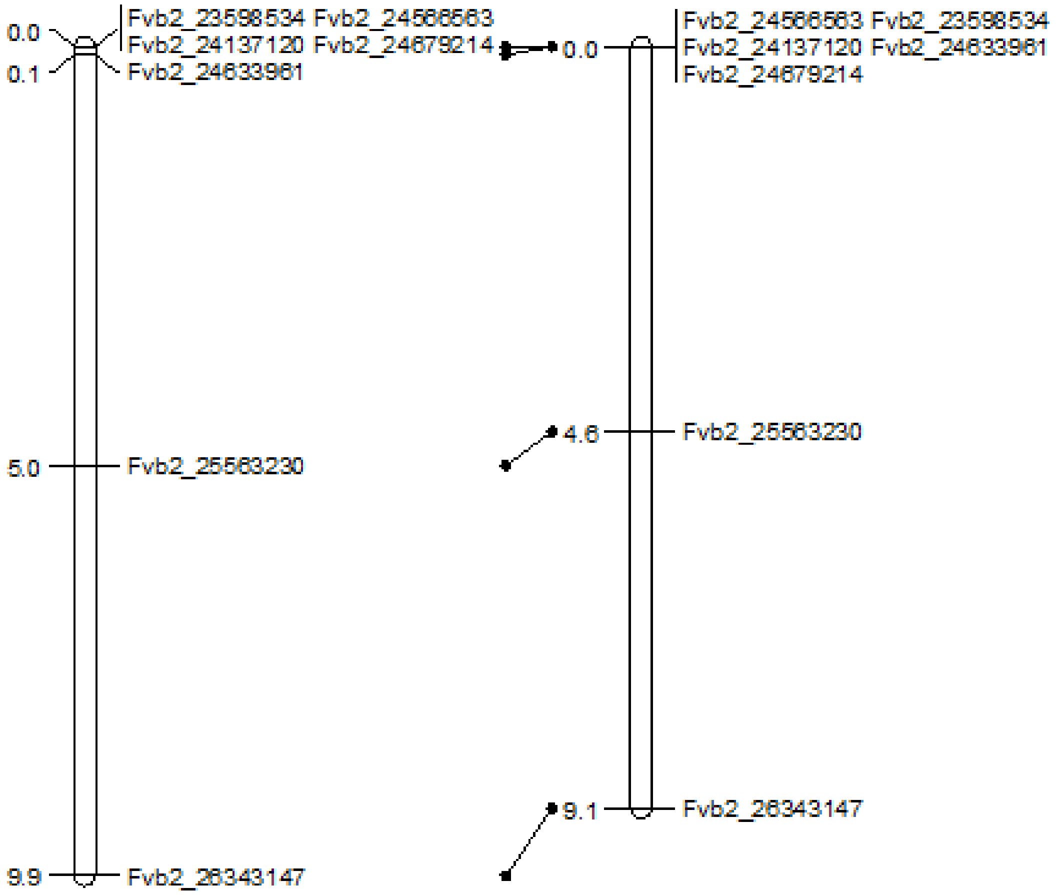

Holiday_31

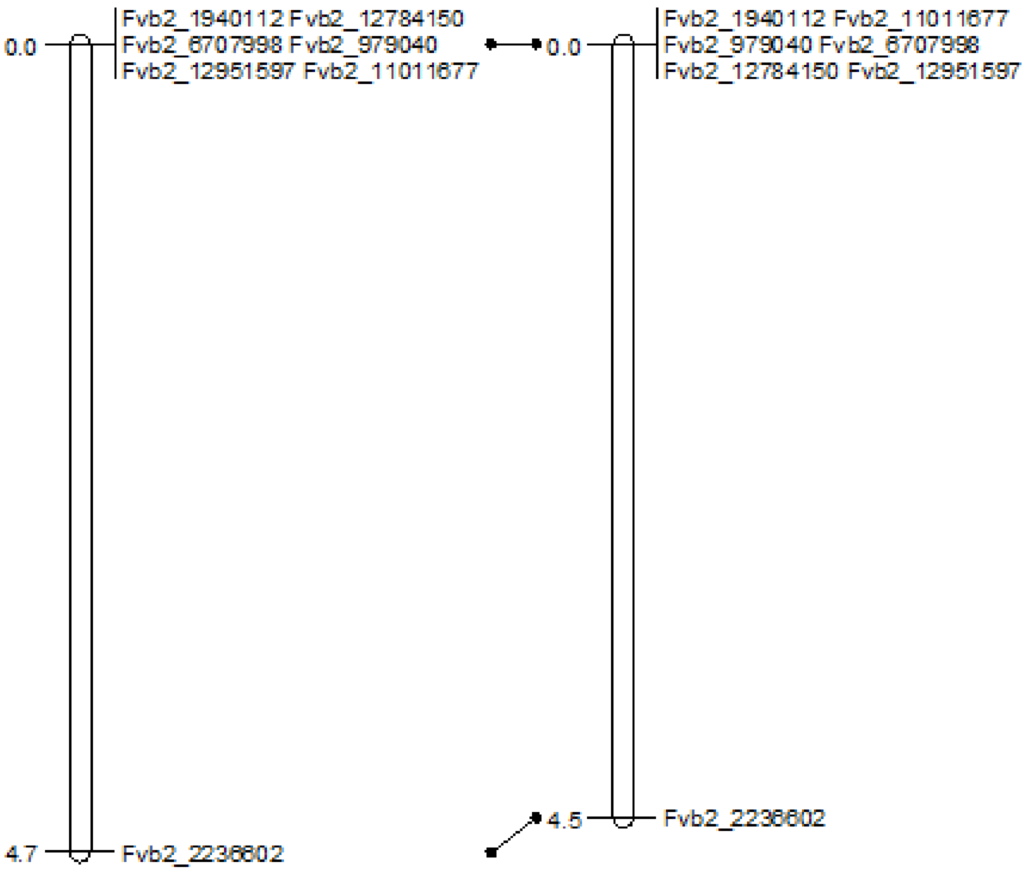

Fvb_2

Holiday_34

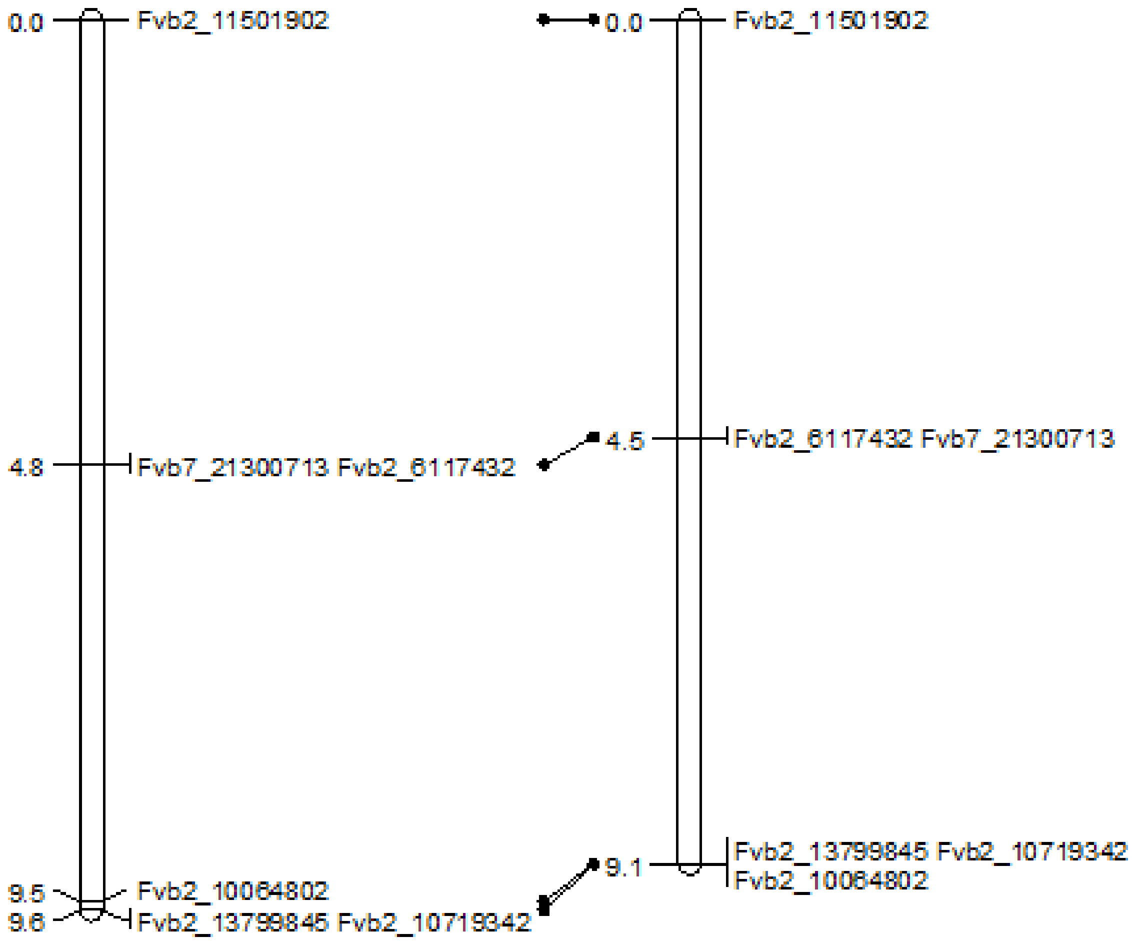

Holiday_35

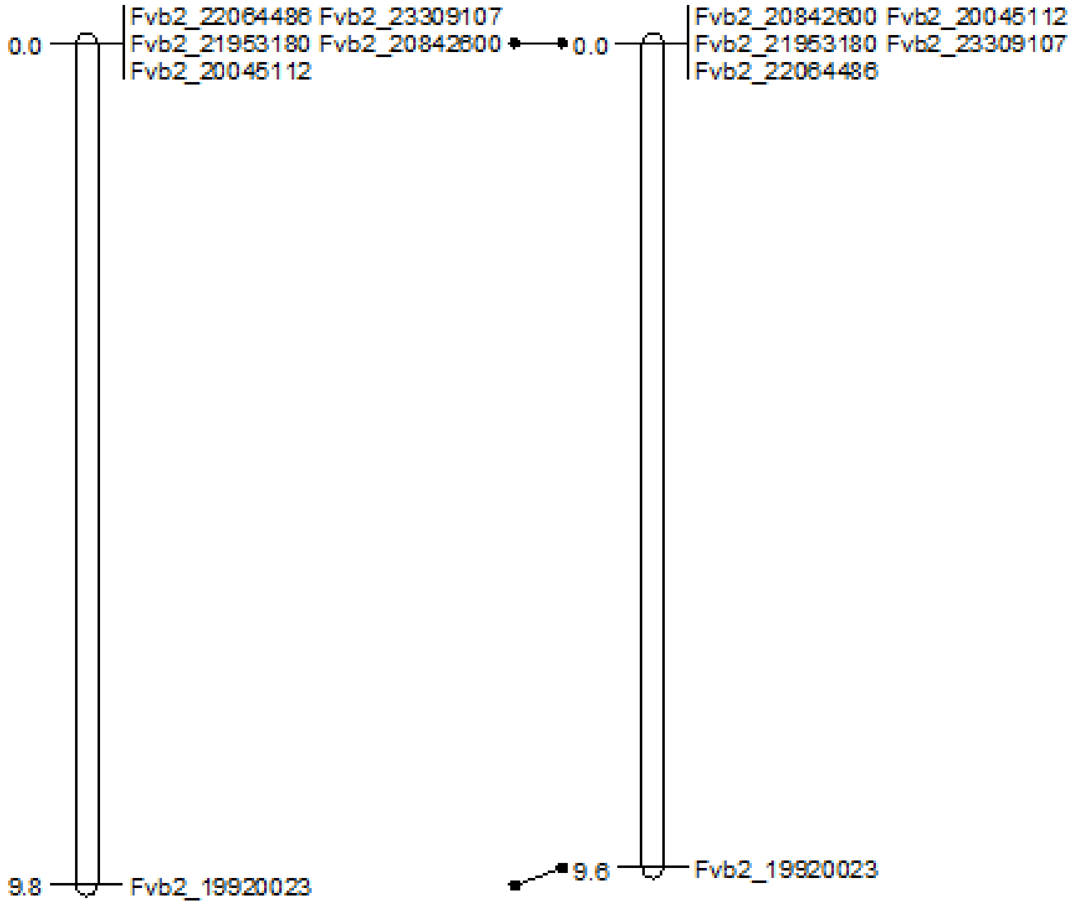

Fvb_2

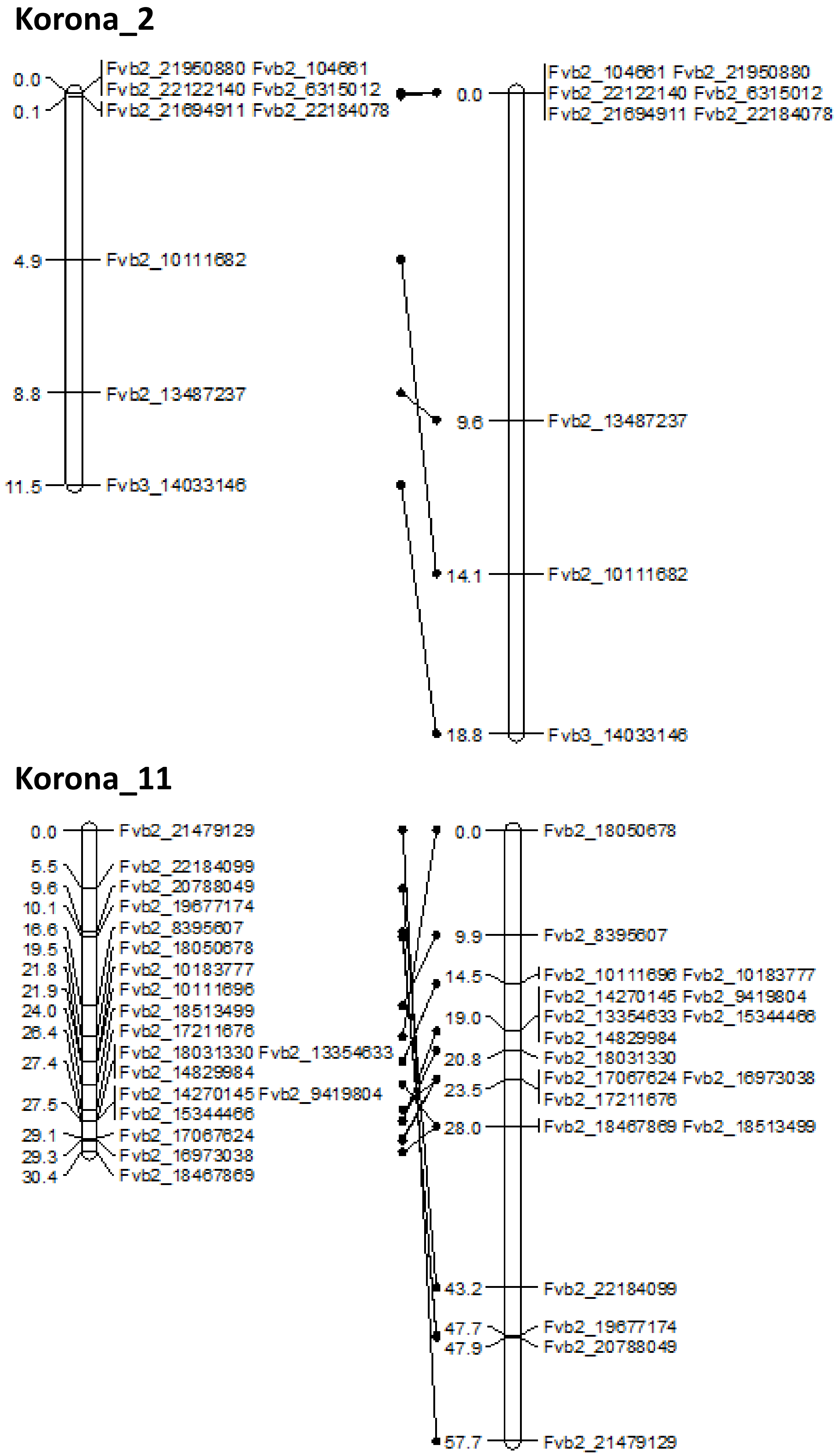
Korona_2
0.0
0.1
Fvb2_21950880 Fvb2_104661
Fvb2_22122140 Fvb2_6315012
Fvb2_21694911 Fvb2_22184078
4.9 Fvb2_10111682
8.8 Fvb2_13487237
11.5 Fvb3_14033146
0.0
Fvb2_104661 Fvb2_21950880
Fvb2_22122140 Fvb2_6315012
Fvb2_21694911 Fvb2_22184078
9.6 Fvb2_13487237
14.1 Fvb2_10111682
18.8 Fvb3_14033146
Korona_11
0.0 Fvb2_21479129
5.5 Fvb2_22184099
9.6 Fvb2_20788049
10.1 Fvb2_19677174
16.6 Fvb2_8395607
19.5 Fvb2_18050678
21.8 Fvb2_10183777
21.9 Fvb2_10111696
24.0 Fvb2_18513499
26.4 Fvb2_17211676
27.4 Fvb2_18031330 Fvb2_13354633
Fvb2_14829984
27.5 Fvb2_14270145 Fvb2_9419804
Fvb2_15344466
29.1 Fvb2_17067624
29.3 Fvb2_16973038
30.4 Fvb2_18467869
0.0 Fvb2_18050678
9.9 Fvb2_8395607
14.5 Fvb2_10111696 Fvb2_10183777
19.0 Fvb2_14270145 Fvb2_9419804
Fvb2_13354633 Fvb2_15344466
Fvb2_14829984
20.8 Fvb2_18031330
23.5 Fvb2_17067624 Fvb2_16973038
Fvb2_17211676
28.0 Fvb2_18467869 Fvb2_18513499
43.2 Fvb2_22184099
47.7 Fvb2_19677174
47.9 Fvb2_20788049
57.7 Fvb2_21479129

Fvb_2

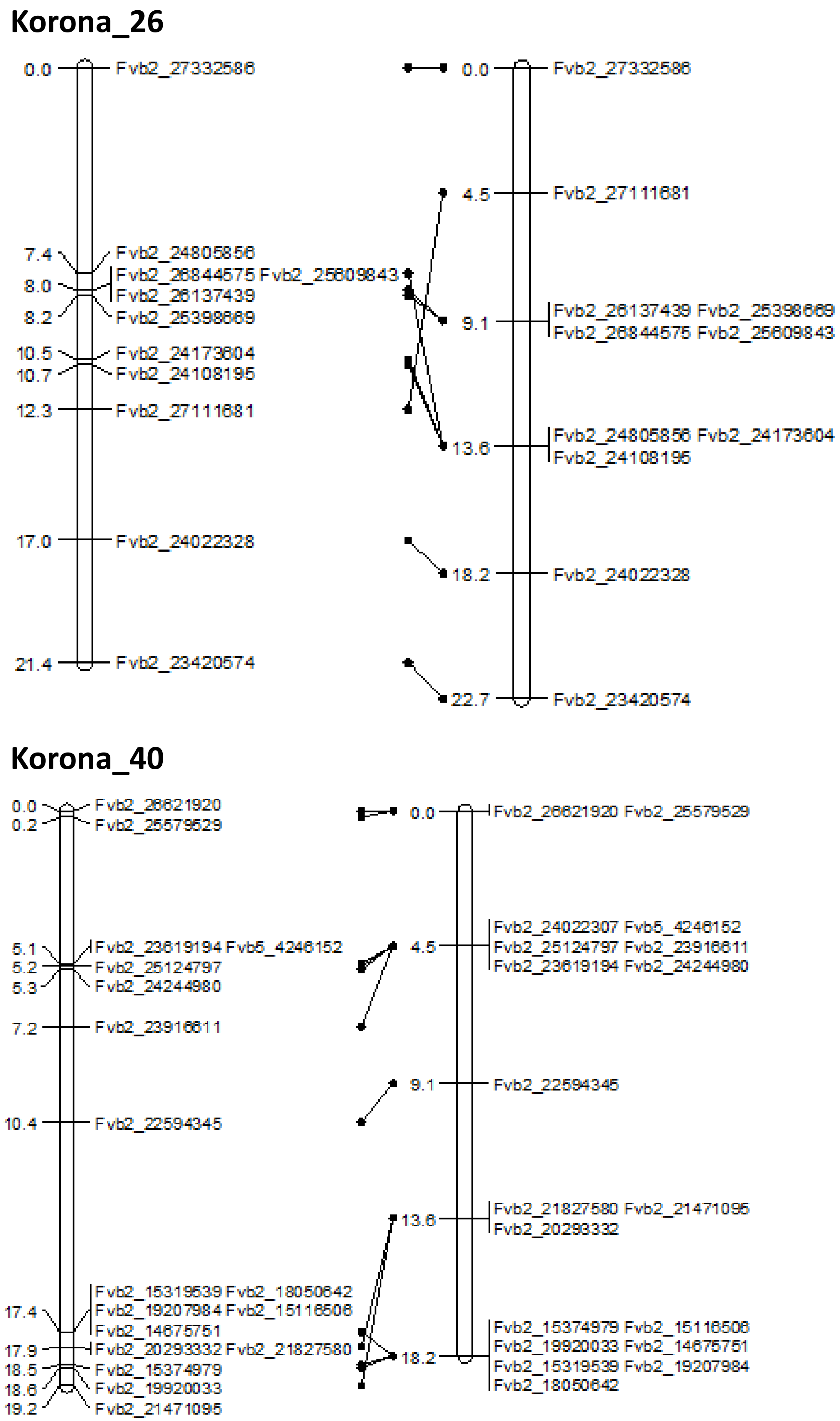
Korona_26
0.0
Fvb2_27332586
7.4
Fvb2_24805856
8.0
Fvb2_26844575 Fvb2_25609843
Fvb2_26137439
8.2
Fvb2_25398669
10.5
Fvb2_24173604
10.7
Fvb2_24108195
12.3
Fvb2_27111681
17.0
Fvb2_24022328
21.4
Fvb2_23420574
0.0
Fvb2_27332586
4.5
Fvb2_27111681
9.1
Fvb2_26137439 Fvb2_25398669
Fvb2_26844575 Fvb2_25609843
13.6
Fvb2_24805856 Fvb2_24173604
Fvb2_24108195
18.2
Fvb2_24022328
22.7
Fvb2_23420574
Korona_40
0.0
Fvb2_26621920
0.2
Fvb2_25579529
5.1
Fvb2_23619194 Fvb5_4246152
5.2
Fvb2_25124797
5.3
Fvb2_24244980
7.2
Fvb2_23916611
10.4
Fvb2_22594345
17.4
Fvb2_15319539 Fvb2_18050642
Fvb2_19207984 Fvb2_15116506
Fvb2_14675751
17.9
Fvb2_20293332 Fvb2_21827580
18.5
Fvb2_15374979
18.6
Fvb2_19920033
19.2
Fvb2_21471095
0.0
Fvb2_26621920 Fvb2_25579529
4.5
Fvb2_24022307 Fvb5_4246152
Fvb2_25124797 Fvb2_23916611
Fvb2_23619194 Fvb2_24244980
9.1
Fvb2_22594345
13.6
Fvb2_21827580 Fvb2_21471095
Fvb2_20293332
18.2
Fvb2_15374979 Fvb2_15116506
Fvb2_19920033 Fvb2_14675751
Fvb2_15319539 Fvb2_19207984
Fvb2_18050642

Fvb_2

Korona_49

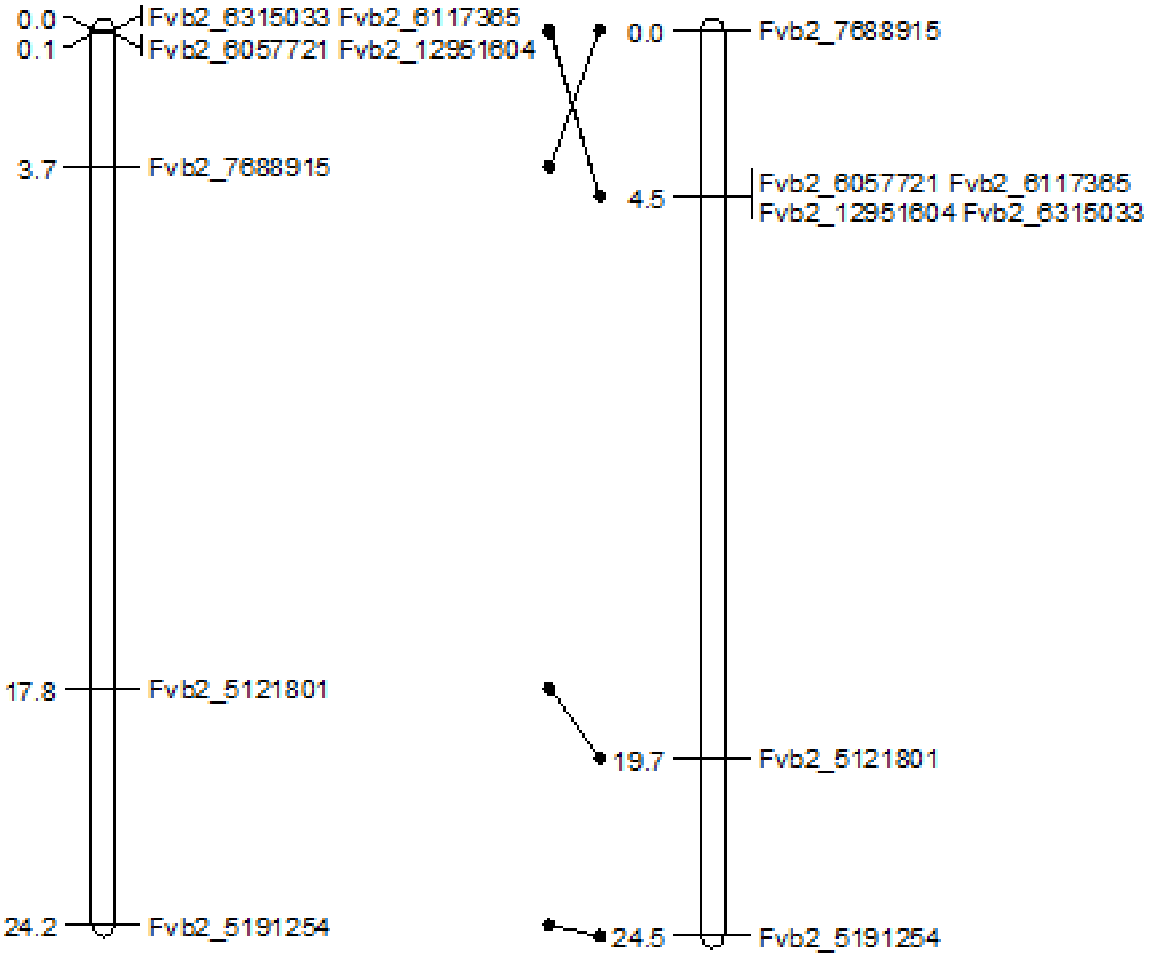

Fvb_3

Holiday_8

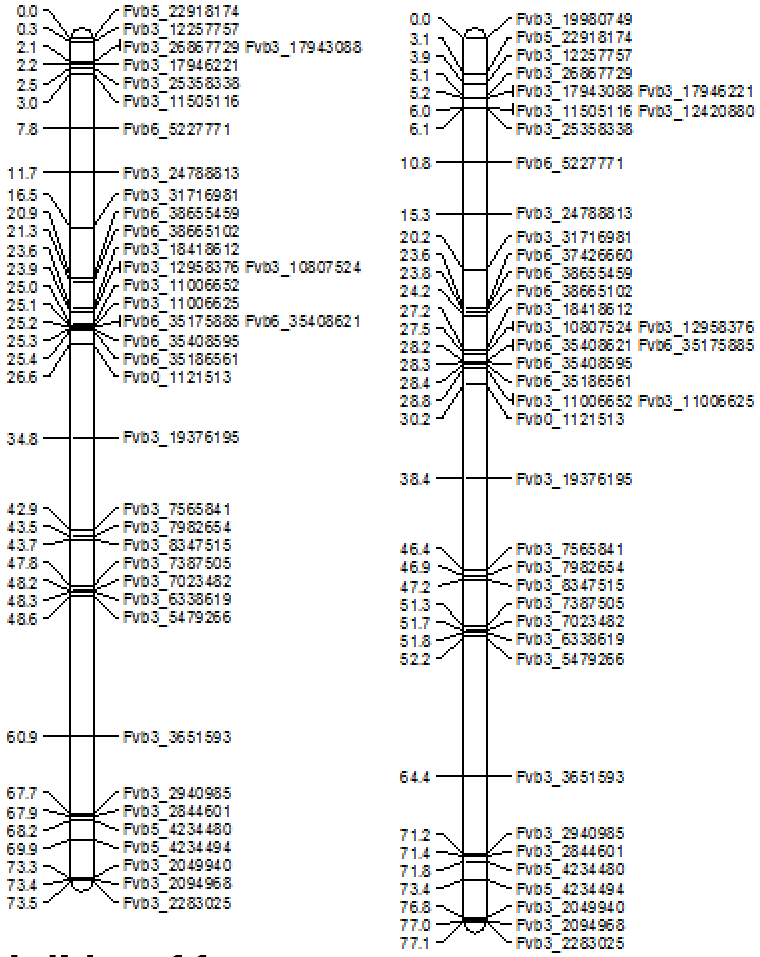

Holiday_11

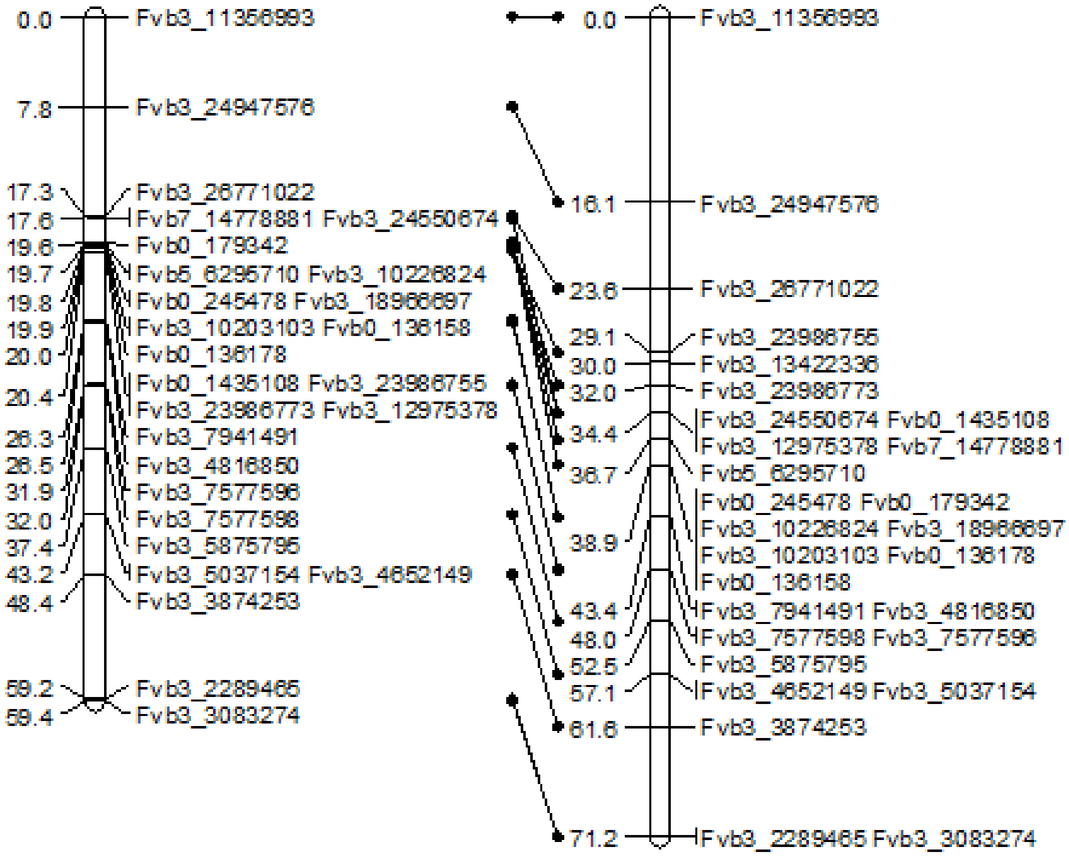

Fvb_3

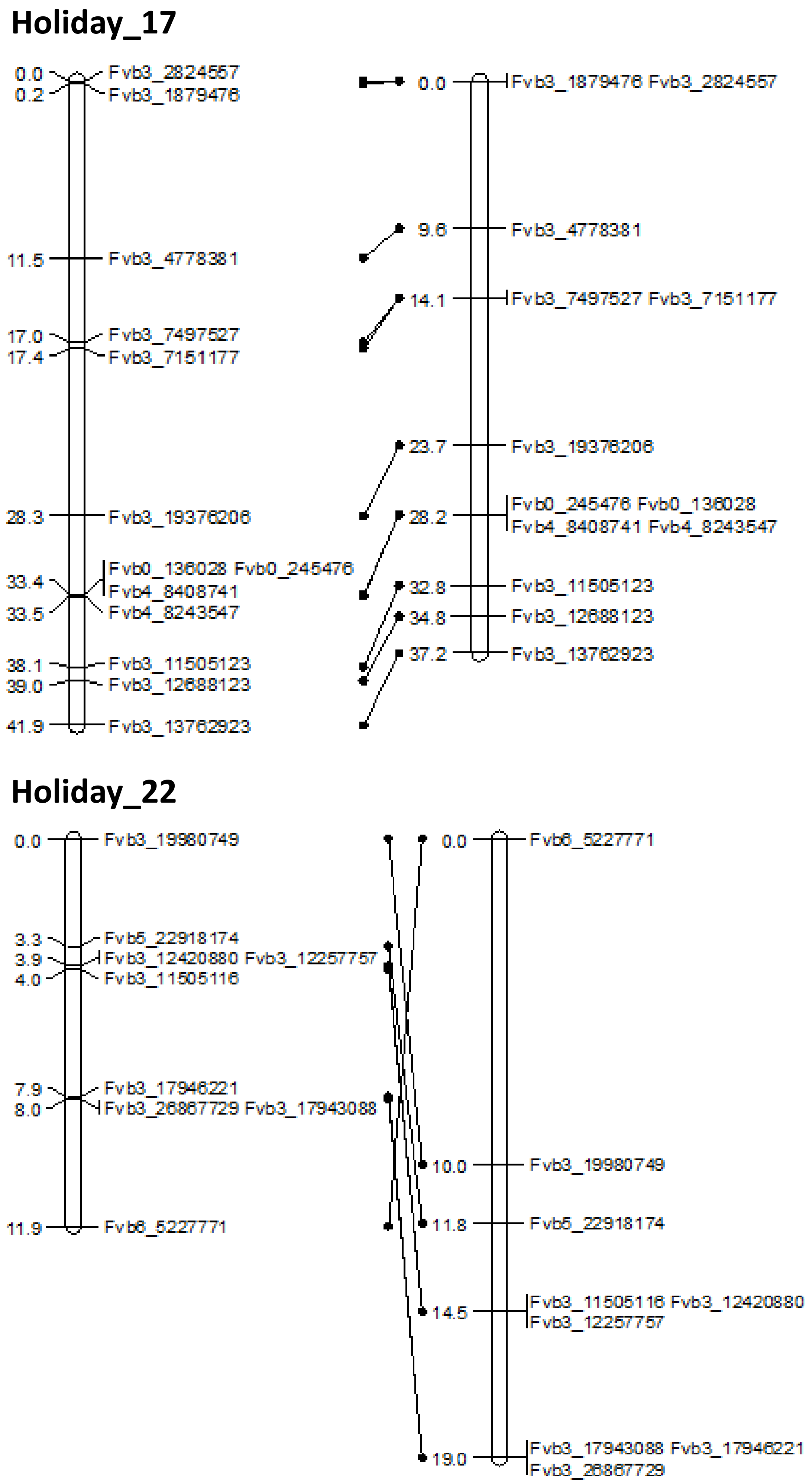

Holiday_17
0.0 Fvb3_2824557
0.2 Fvb3_1879476
11.5 Fvb3_4778381
17.0 Fvb3_7497527
17.4 Fvb3_7151177
28.3 Fvb3_19376206
33.4 Fvb0_136028 Fvb0_245476
Fvb4_8408741
33.5 Fvb4_8243547
38.1 Fvb3_11505123
39.0 Fvb3_12688123
41.9 Fvb3_13762923
0.0 Fvb3_1879476 Fvb3_2824557
9.6 Fvb3_4778381
14.1 Fvb3_7497527 Fvb3_7151177
23.7 Fvb3_19376206
28.2 Fvb0_245476 Fvb0_136028
Fvb4_8408741 Fvb4_8243547
32.8 Fvb3_11505123
34.8 Fvb3_12688123
37.2 Fvb3_13762923
Holiday_22
0.0 Fvb3_19980749
3.3 Fvb5_22918174
3.9 Fvb3_12420880 Fvb3_12257757
4.0 Fvb3_11505116
7.9 Fvb3_17946221
8.0 Fvb3_26867729 Fvb3_17943088
11.9 Fvb6_5227771
0.0 Fvb6_5227771
10.0 Fvb3_19980749
11.8 Fvb5_22918174
14.5 Fvb3_11505116 Fvb3_12420880
Fvb3_12257757
19.0 Fvb3_17943088 Fvb3_17946221
Fvb3_26867729

Fvb_3

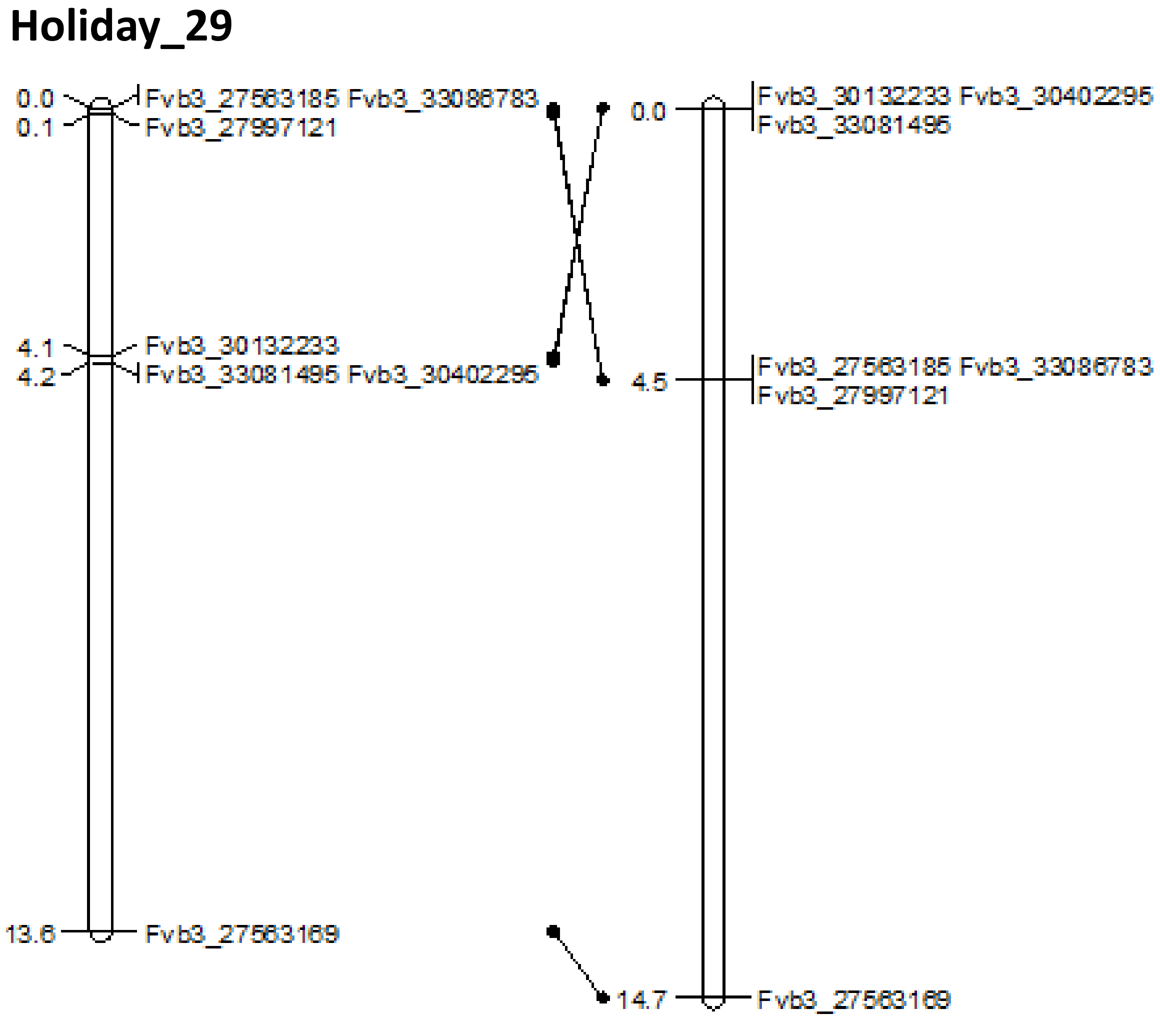
Holiday_29
0.0
0.1
Fvb3_27563185 Fvb3_33086783
Fvb3_27997121
4.1
4.2
Fvb3_30132233
Fvb3_33081495 Fvb3_30402295
13.6
Fvb3_27563169
0.0
Fvb3_30132233 Fvb3_30402295
Fvb3_33081495
4.5
Fvb3_27563185 Fvb3_33086783
Fvb3_27997121
14.7
Fvb3_27563169

Fvb_3

Korona_4

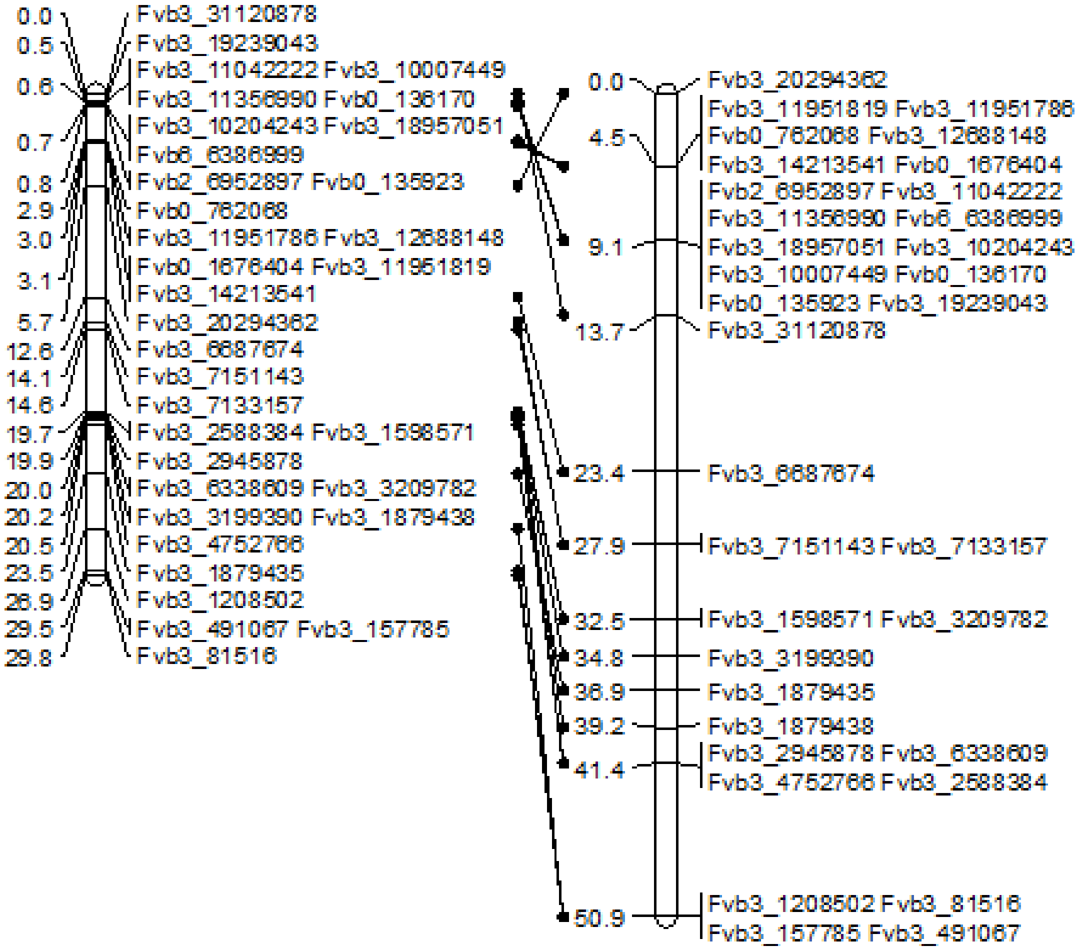

Korona_21

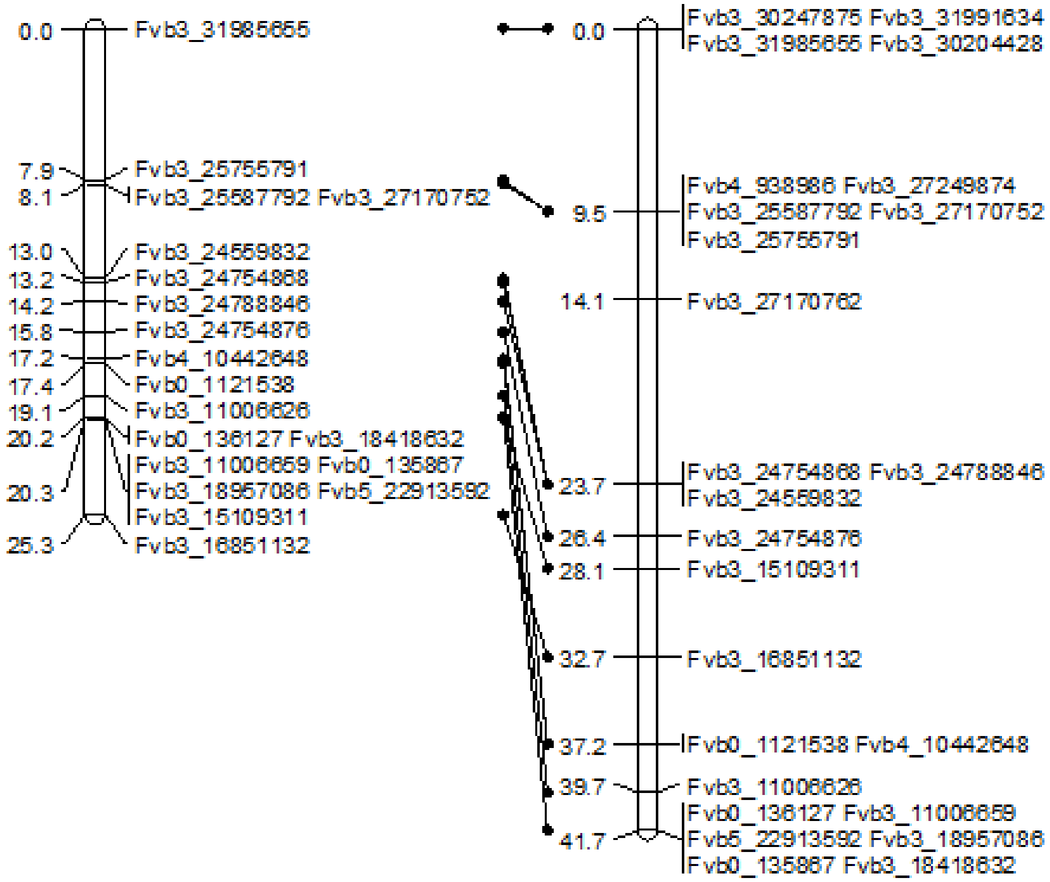

Fvb_3

Korona_22

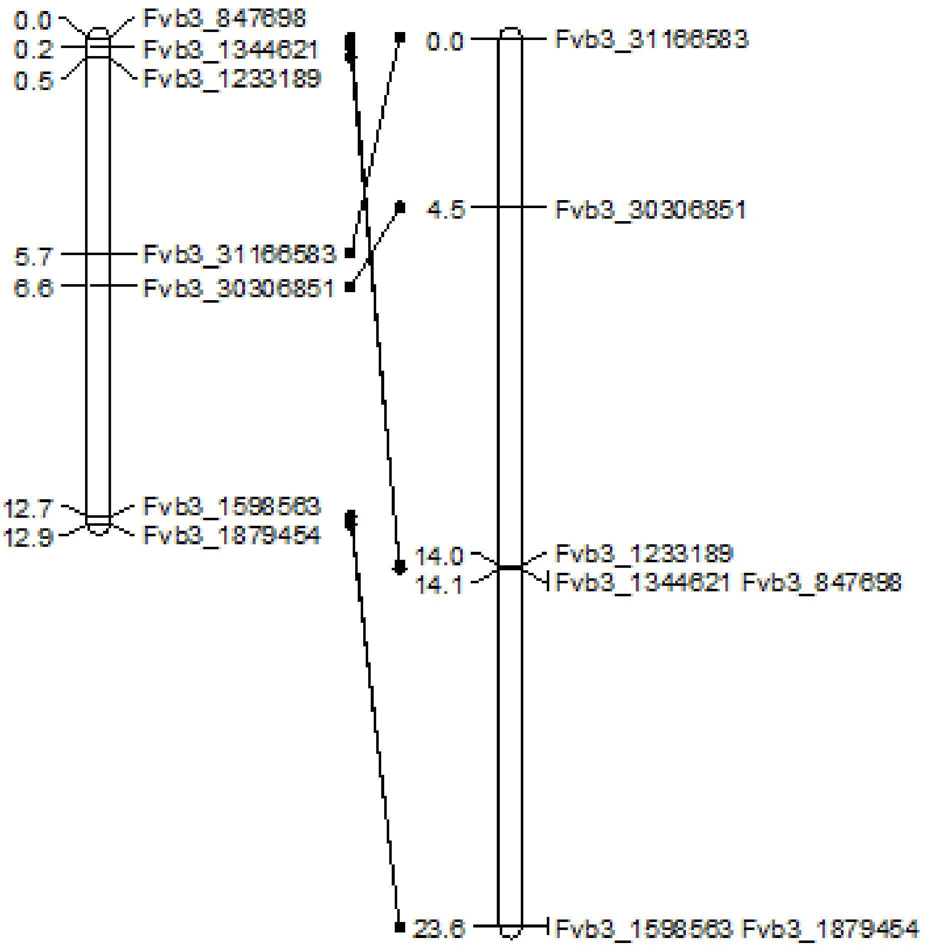

Korona_31

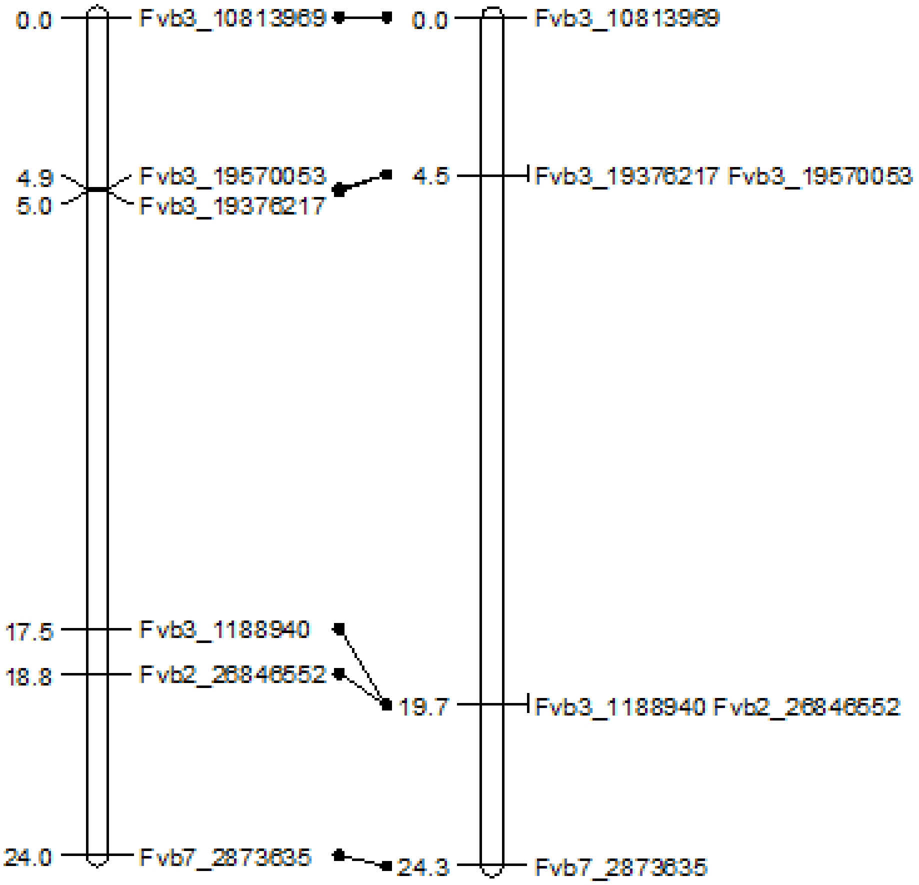

Fvb_3

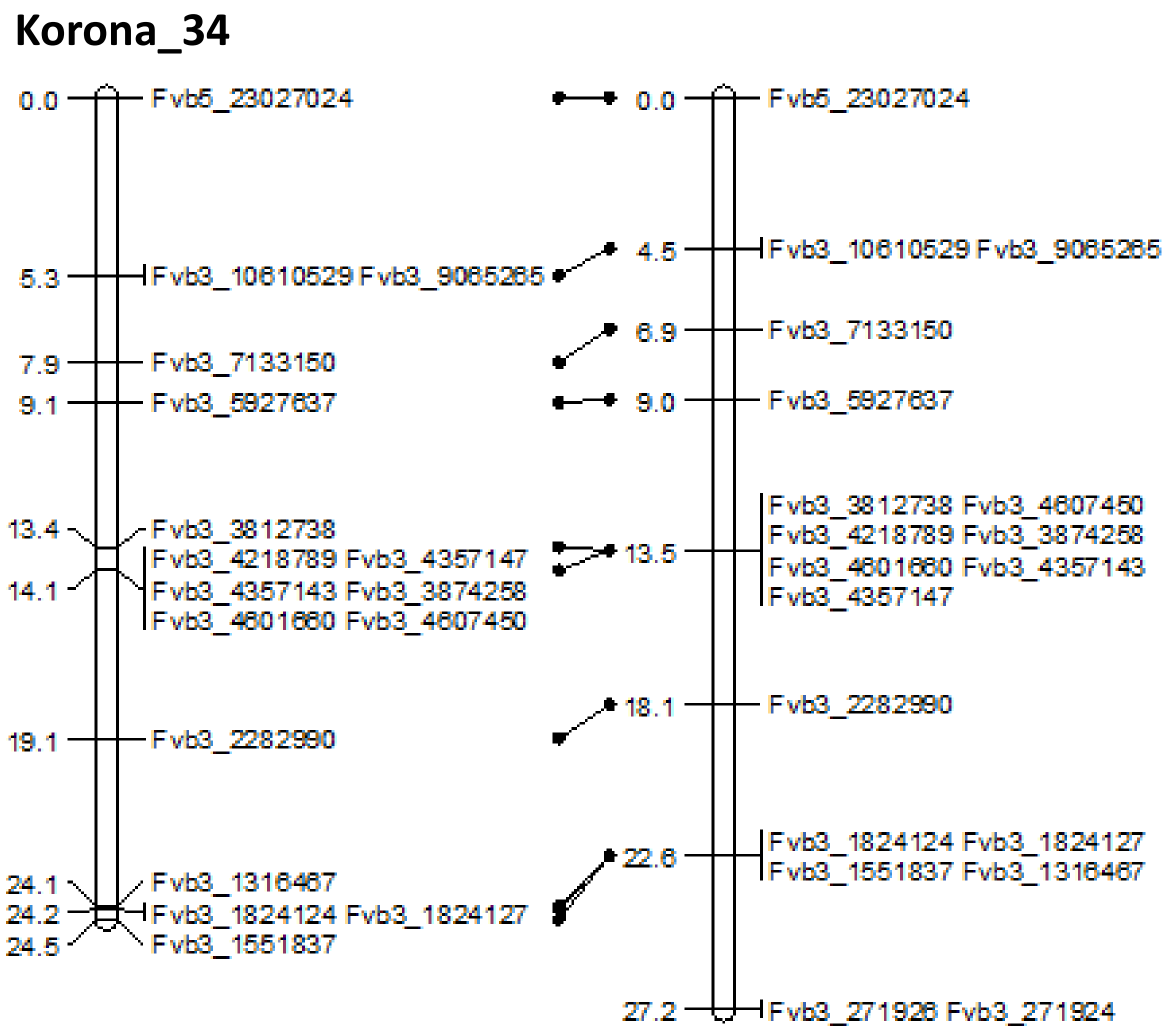
Korona_34
0.0 Fvb5_23027024
5.3 Fvb3_10610529 Fvb3_9065265
7.9 Fvb3_7133150
9.1 Fvb3_5927637
13.4 Fvb3_3812738
14.1 Fvb3_4218789 Fvb3_4357147
Fvb3_4357143 Fvb3_3874258
Fvb3_4601660 Fvb3_4607450
19.1 Fvb3_2282990
24.1 Fvb3_1316467
24.2 Fvb3_1824124 Fvb3_1824127
24.5 Fvb3_1551837
0.0 Fvb5_23027024
4.5 Fvb3_10610529 Fvb3_9065265
6.9 Fvb3_7133150
9.0 Fvb3_5927637
13.5 Fvb3_3812738 Fvb3_4607450
Fvb3_4218789 Fvb3_3874258
Fvb3_4601660 Fvb3_4357143
Fvb3_4357147
18.1 Fvb3_2282990
22.6 Fvb3_1824124 Fvb3_1824127
Fvb3_1551837 Fvb3_1316467
27.2 Fvb3_271926 Fvb3_271924

Fvb_4

Holiday_13

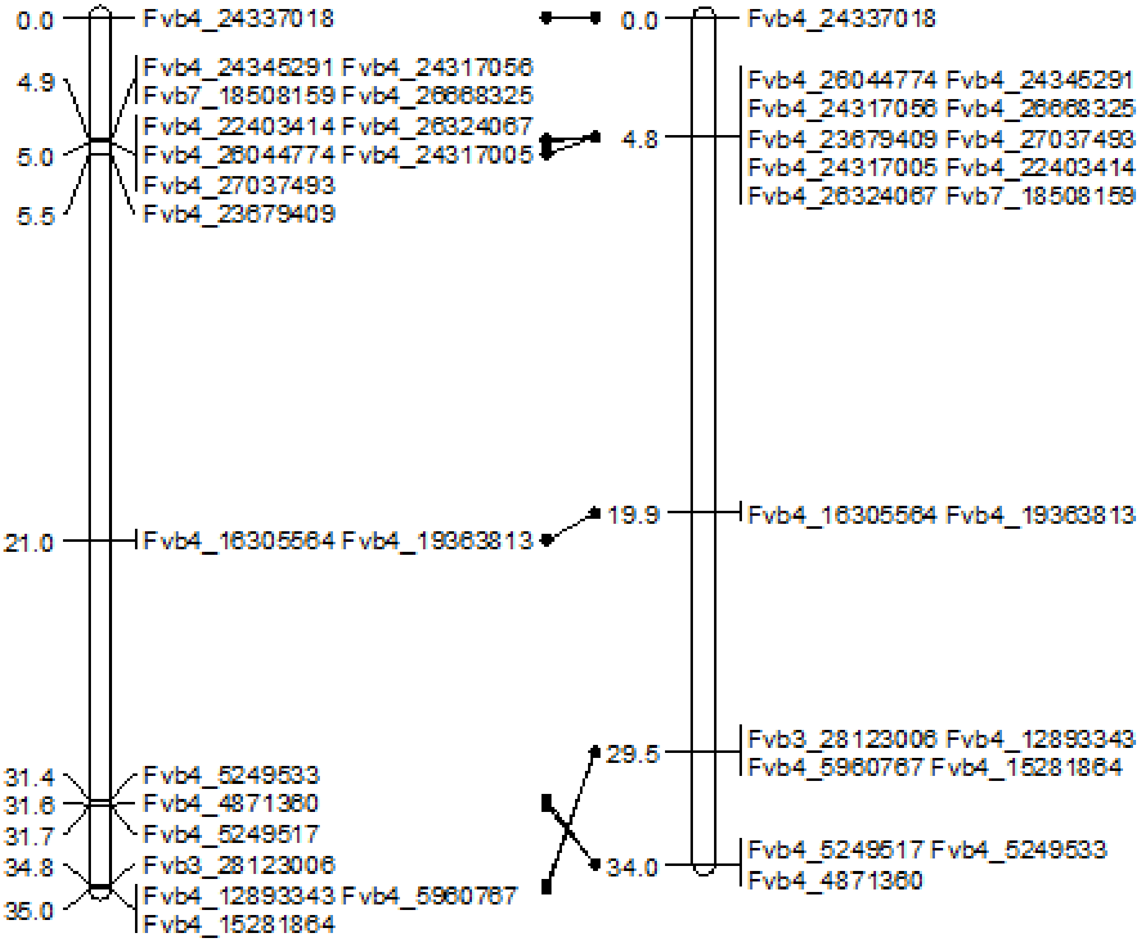

Holiday_27

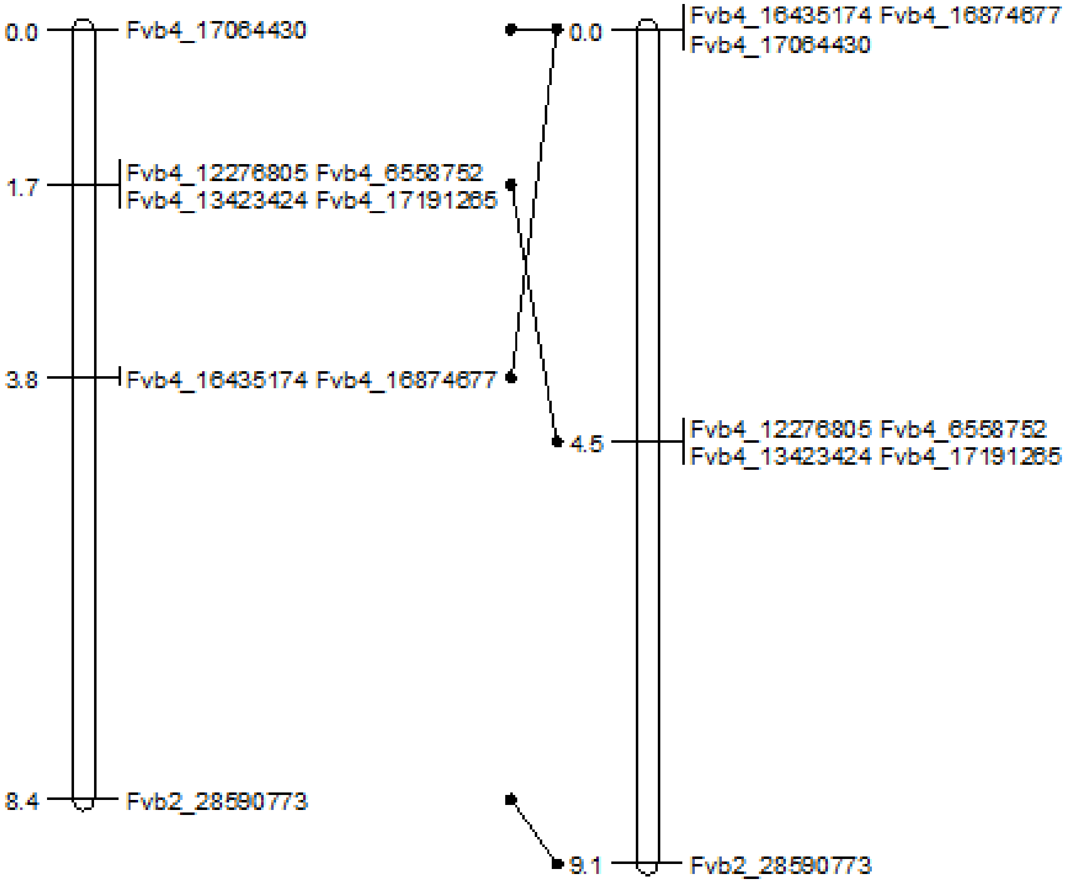

Fvb_4

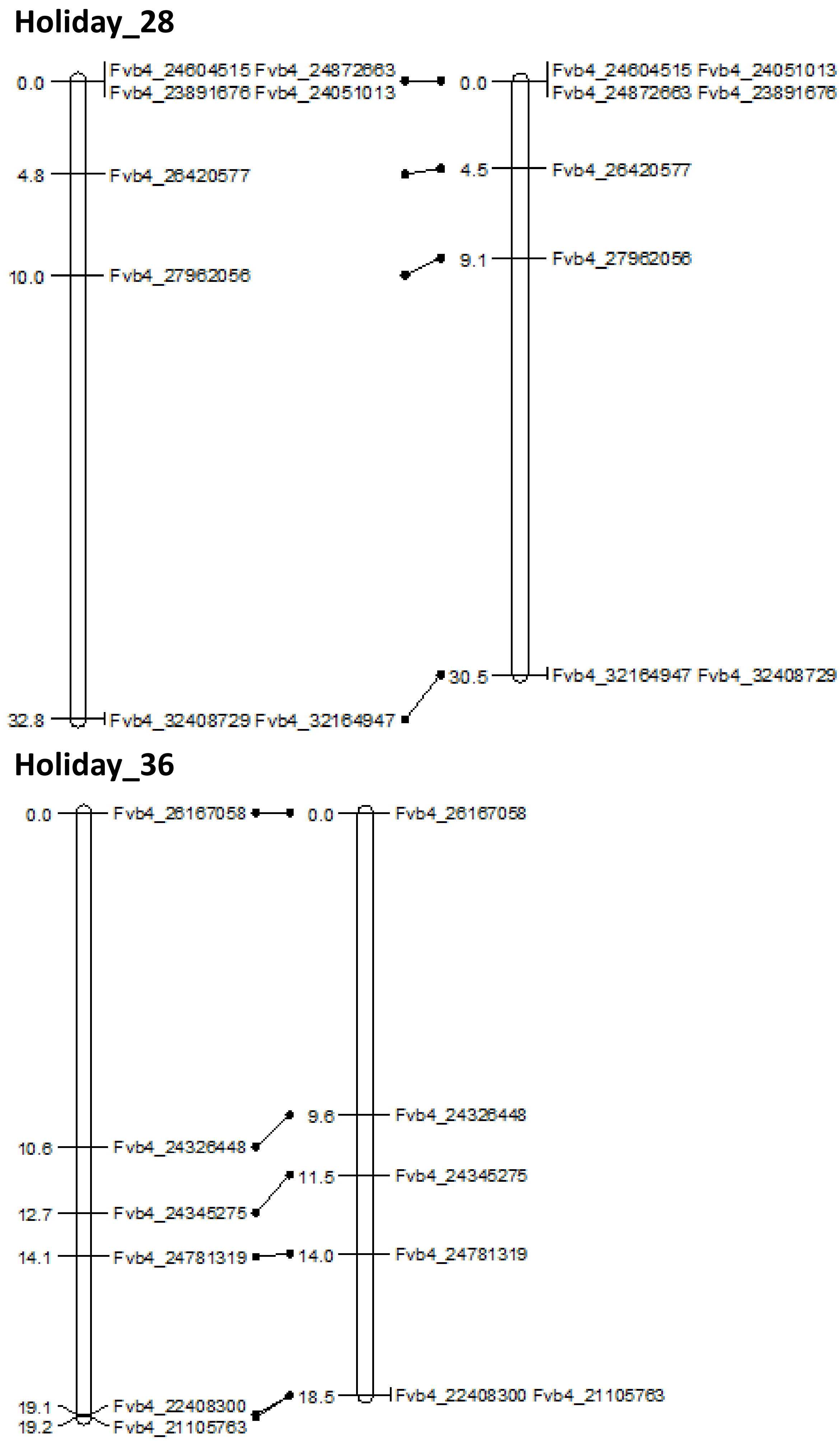
Holiday_28
0.0
Fvb4_24604515 Fvb4_24872663
Fvb4_23891676 Fvb4_24051013
4.8
Fvb4_26420577
10.0
Fvb4_27962056
32.8
Fvb4_32408729 Fvb4_32164947
0.0
Fvb4_24604515 Fvb4_24051013
Fvb4_24872663 Fvb4_23891676
4.5
Fvb4_26420577
9.1
Fvb4_27962056
30.5
Fvb4_32164947 Fvb4_32408729
Holiday_36
0.0
Fvb4_26167058
10.6
Fvb4_24326448
12.7
Fvb4_24345275
14.1
Fvb4_24781319
19.1
Fvb4_22408300
19.2
Fvb4_21105763
0.0
Fvb4_26167058
9.6
Fvb4_24326448
11.5
Fvb4_24345275
14.0
Fvb4_24781319
18.5
Fvb4_22408300 Fvb4_21105763

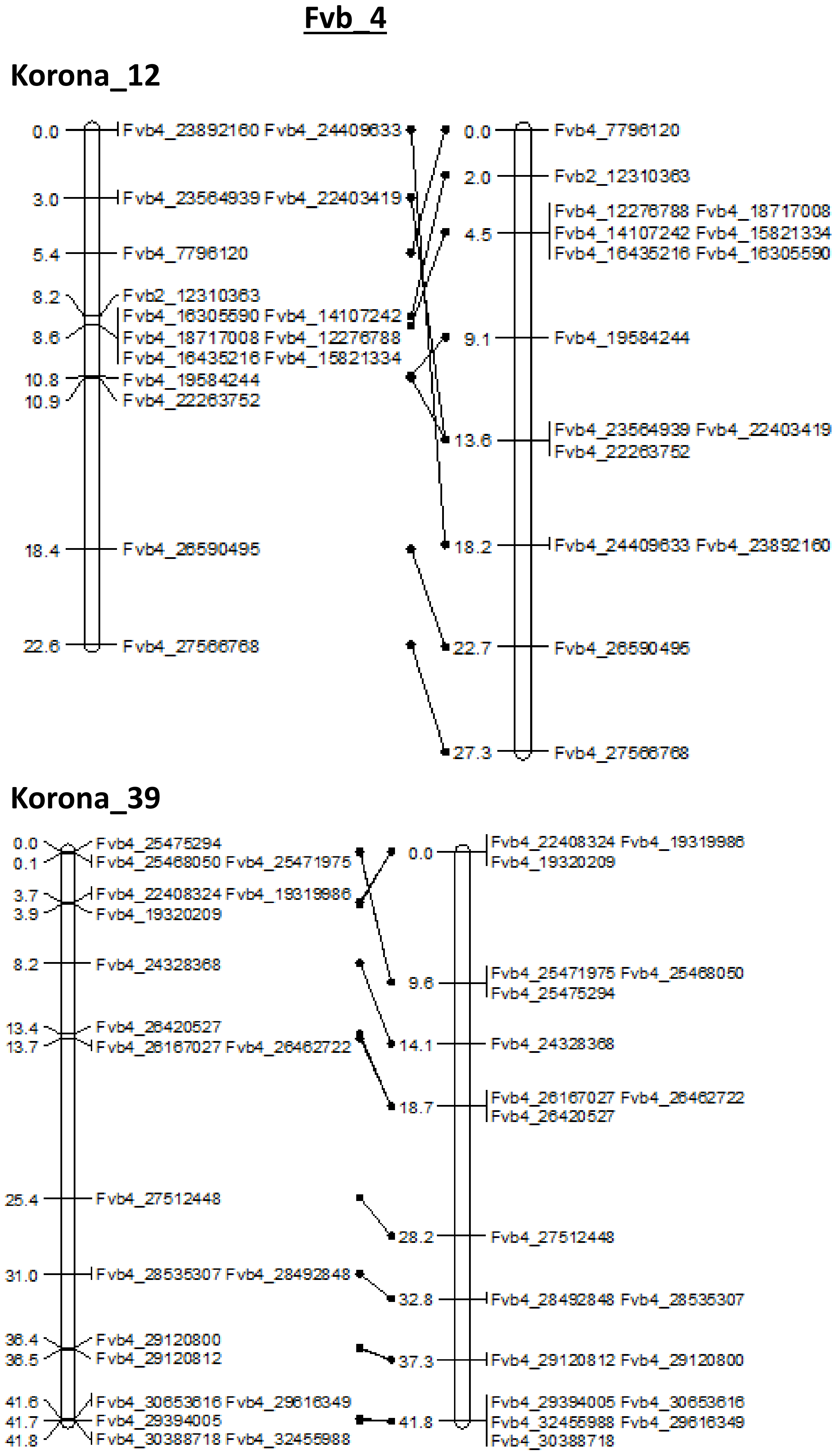

Fvb_4
Korona_12
0.0 Fvb4_23892160 Fvb4_24409633
3.0 Fvb4_23564939 Fvb4_22403419
5.4 Fvb4_7796120
8.2 Fvb2_12310363
8.6 Fvb4_16305590 Fvb4_14107242
Fvb4_18717008 Fvb4_12276788
Fvb4_16435216 Fvb4_15821334
10.8 Fvb4_19584244
10.9 Fvb4_22263752
18.4 Fvb4_26590495
22.6 Fvb4_27566768
0.0 Fvb4_7796120
2.0 Fvb2_12310363
4.5 Fvb4_12276788 Fvb4_18717008
Fvb4_14107242 Fvb4_15821334
Fvb4_16435216 Fvb4_16305590
9.1 Fvb4_19584244
13.6 Fvb4_23564939 Fvb4_22403419
Fvb4_22263752
18.2 Fvb4_24409633 Fvb4_23892160
22.7 Fvb4_26590495
27.3 Fvb4_27566768
Korona_39
0.0 Fvb4_25475294
0.1 Fvb4_25468050 Fvb4_25471975
3.7 Fvb4_22408324 Fvb4_19319986
3.9 Fvb4_19320209
8.2 Fvb4_24328368
13.4 Fvb4_26420527
13.7 Fvb4_26167027 Fvb4_26462722
25.4 Fvb4_27512448
31.0 Fvb4_28535307 Fvb4_28492848
36.4 Fvb4_29120800
36.5 Fvb4_29120812
41.6 Fvb4_30653616 Fvb4_29616349
41.7 Fvb4_29394005
41.8 Fvb4_30388718 Fvb4_32455988
0.0 Fvb4_22408324 Fvb4_19319986
Fvb4_19320209
9.6 Fvb4_25471975 Fvb4_25468050
Fvb4_25475294
14.1 Fvb4_24328368
18.7 Fvb4_26167027 Fvb4_26462722
Fvb4_26420527
28.2 Fvb4_27512448
32.8 Fvb4_28492848 Fvb4_28535307
37.3 Fvb4_29120812 Fvb4_29120800
41.8 Fvb4_29394005 Fvb4_30653616
Fvb4_32455988 Fvb4_29616349
Fvb4_30388718

Fvb_4

Korona_43

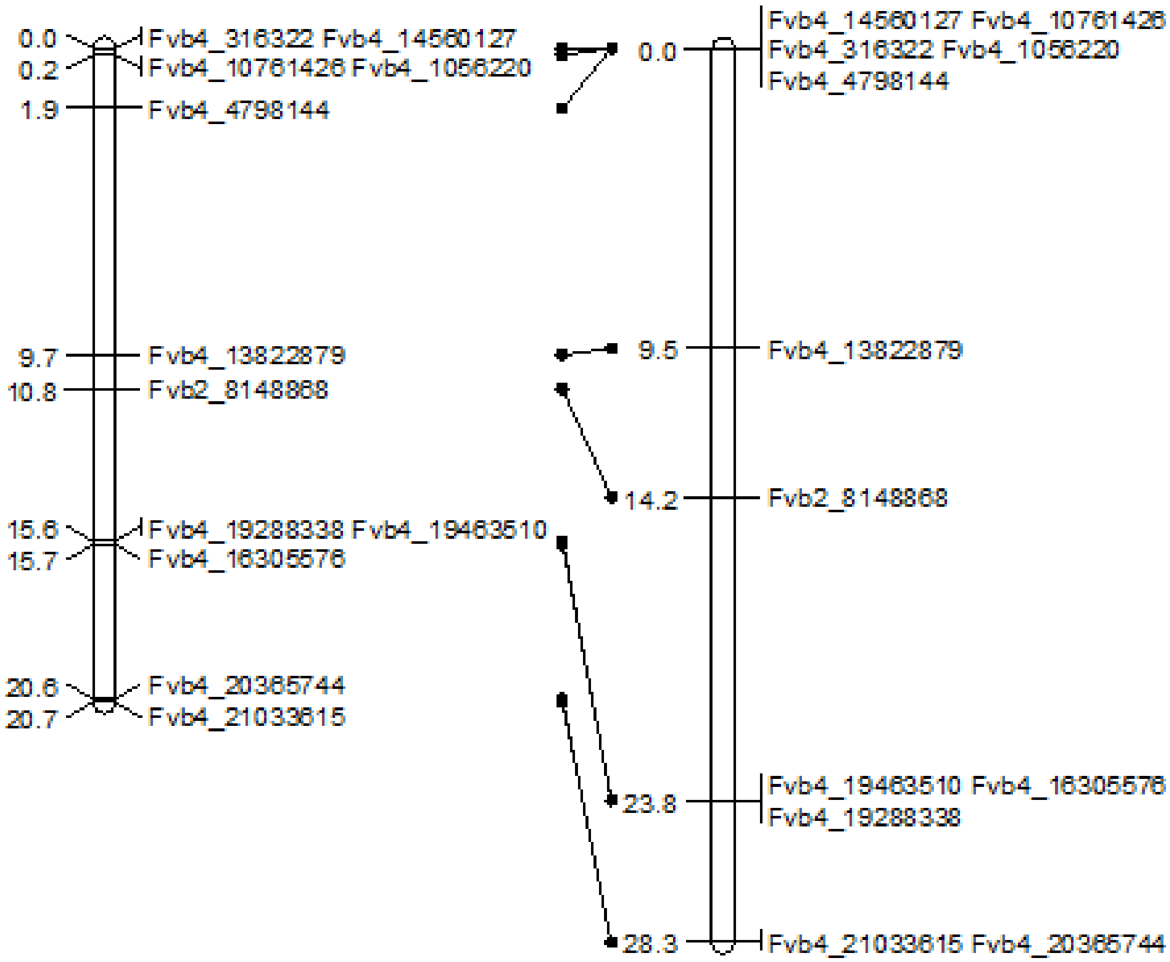

Korona_48

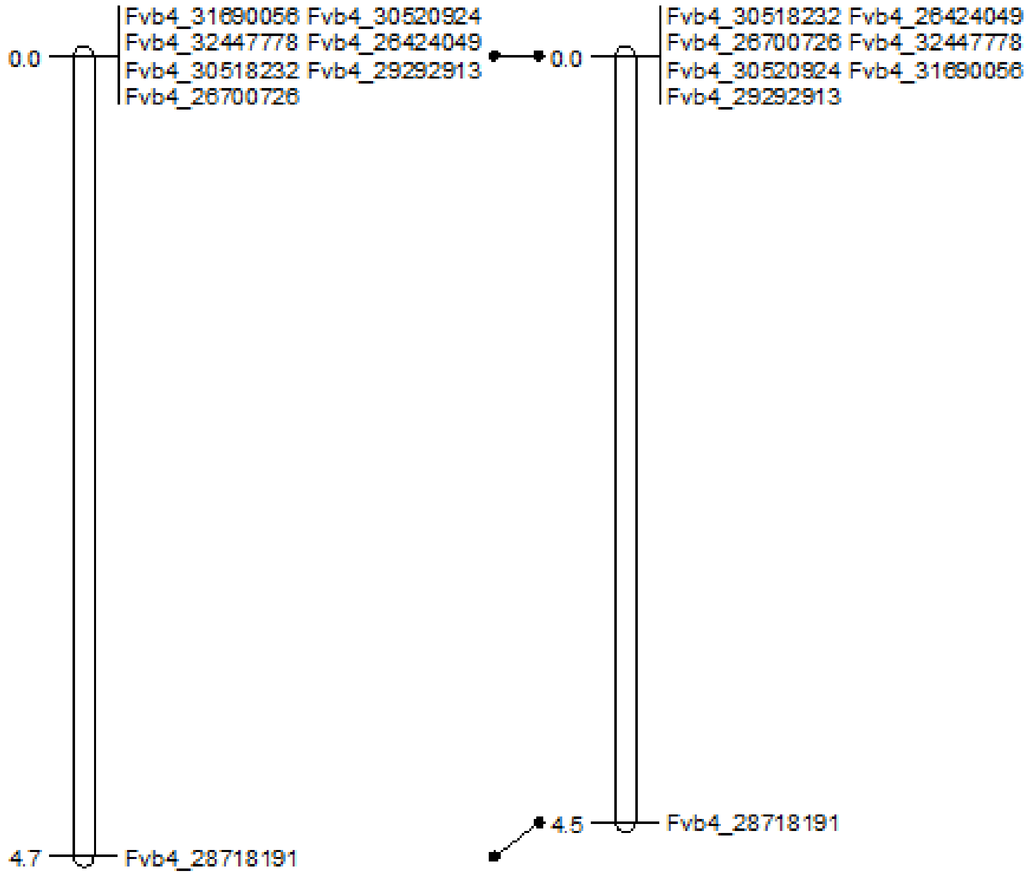

Fvb_5

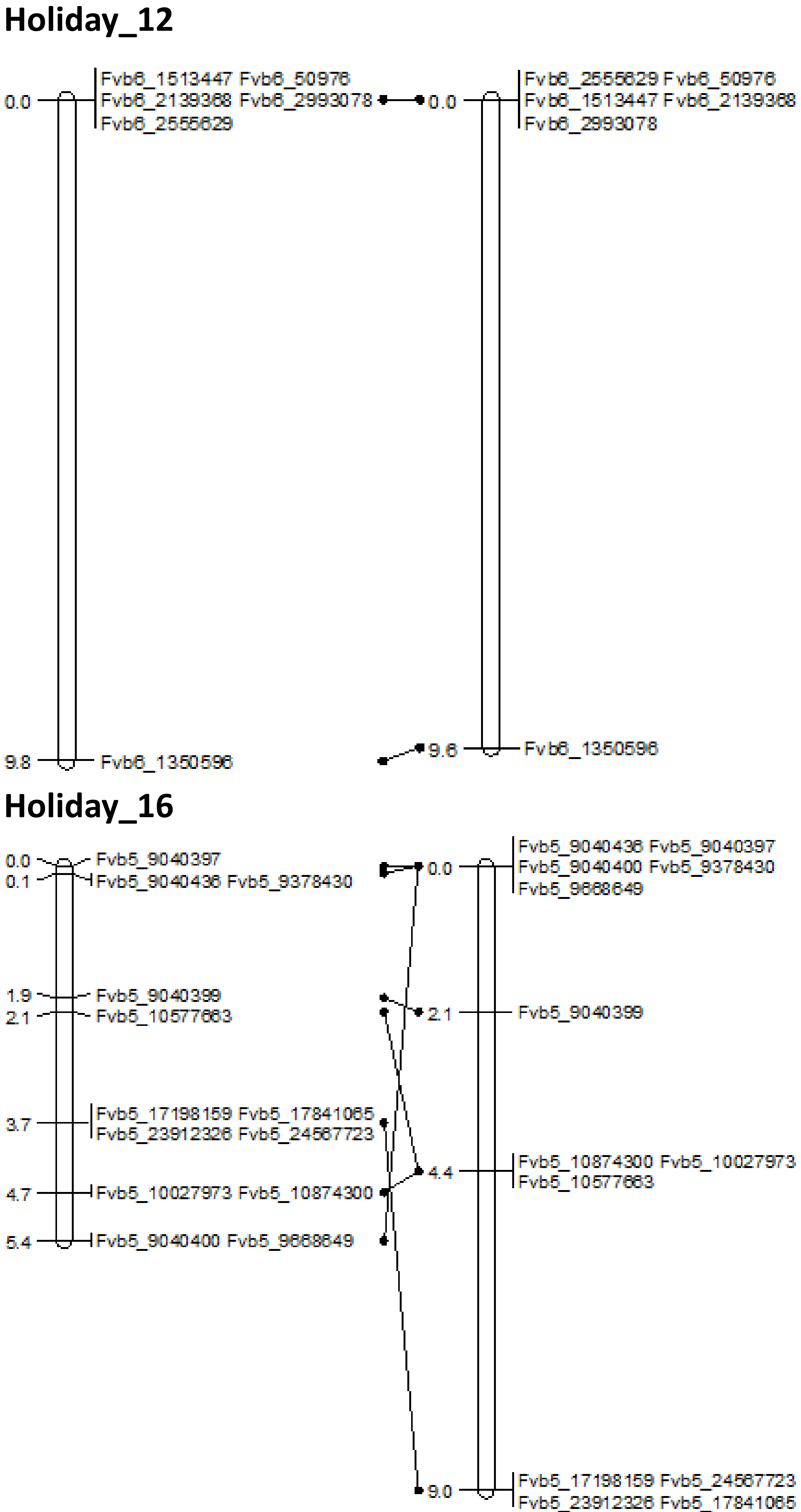
Holiday_12
0.0
Fvb6_1513447 Fvb6_50976
Fvb6_2139368 Fvb6_2993078
Fvb6_2555629
9.8
Fvb6_1350596
0.0
Fvb6_2555629 Fvb6_50976
Fvb6_1513447 Fvb6_2139368
Fvb6_2993078
9.6
Fvb6_1350596
Holiday_16
0.0
Fvb5_9040397
0.1
Fvb5_9040436 Fvb5_9378430
1.9
Fvb5_9040399
2.1
Fvb5_10577663
3.7
Fvb5_17198159 Fvb5_17841065
Fvb5_23912326 Fvb5_24567723
4.7
Fvb5_10027973 Fvb5_10874300
5.4
Fvb5_9040400 Fvb5_9668649
0.0
Fvb5_9040436 Fvb5_9040397
Fvb5_9040400 Fvb5_9378430
Fvb5_9668649
2.1
Fvb5_9040399
4.4
Fvb5_10874300 Fvb5_10027973
Fvb5_10577663
9.0
Fvb5_17198159 Fvb5_24567723
Fvb5_23912326 Fvb5_17841065

Fvb_5

Holiday_19

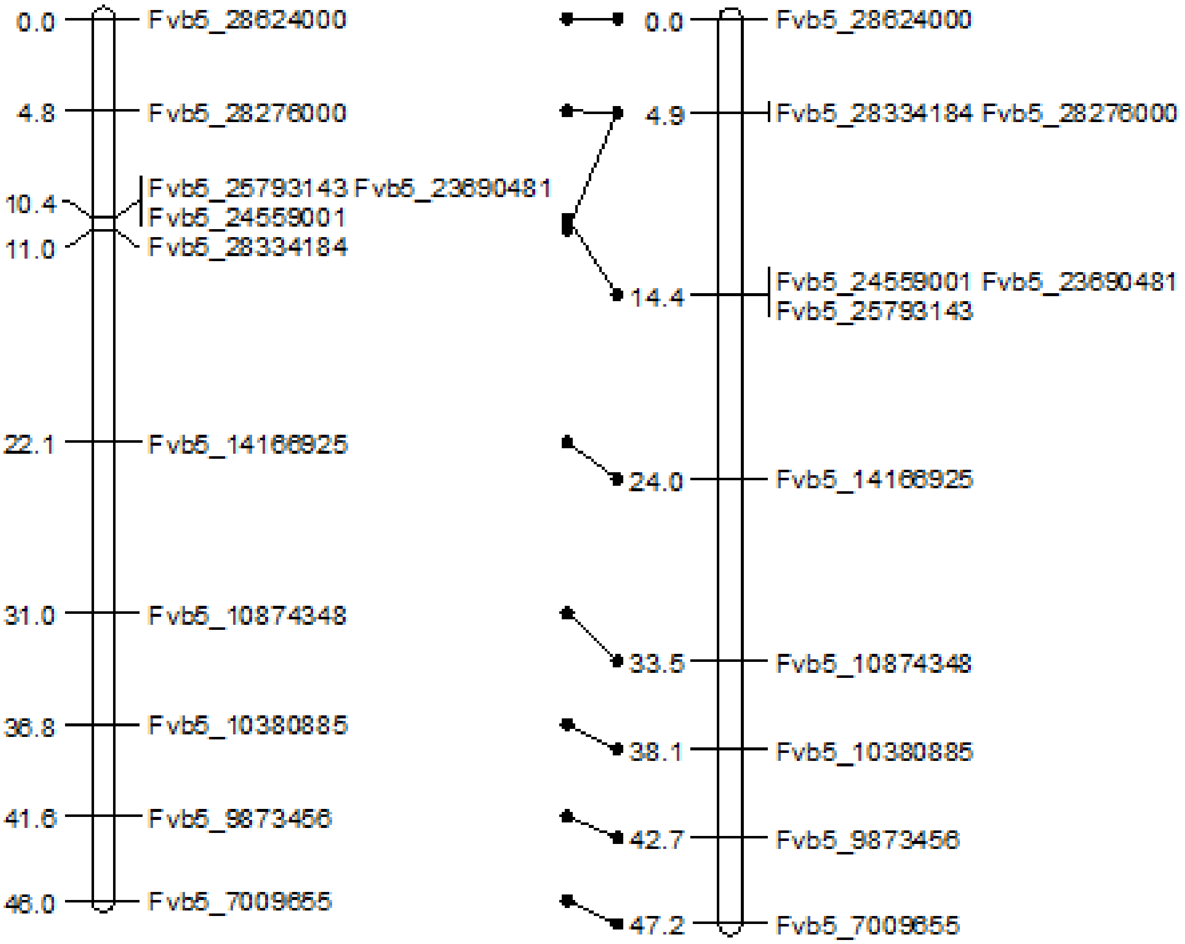

Holiday_23

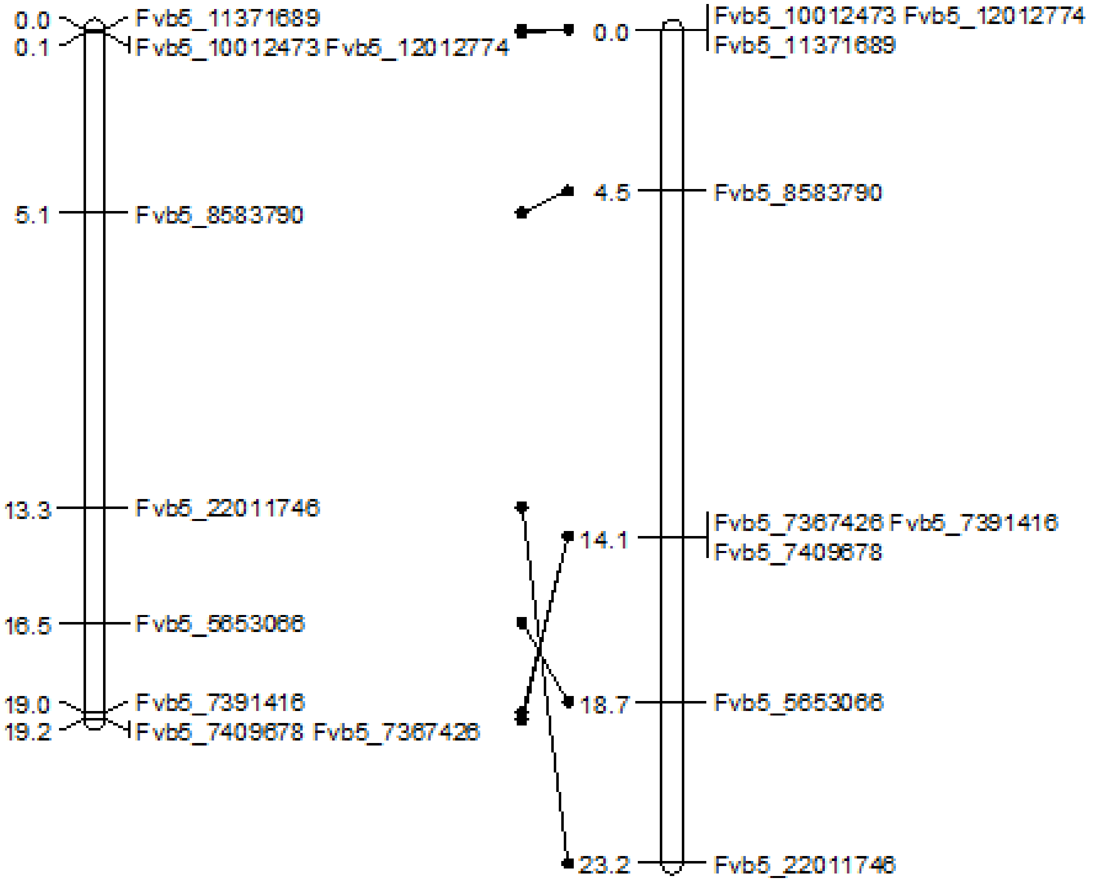

Fvb_5

Korona_1

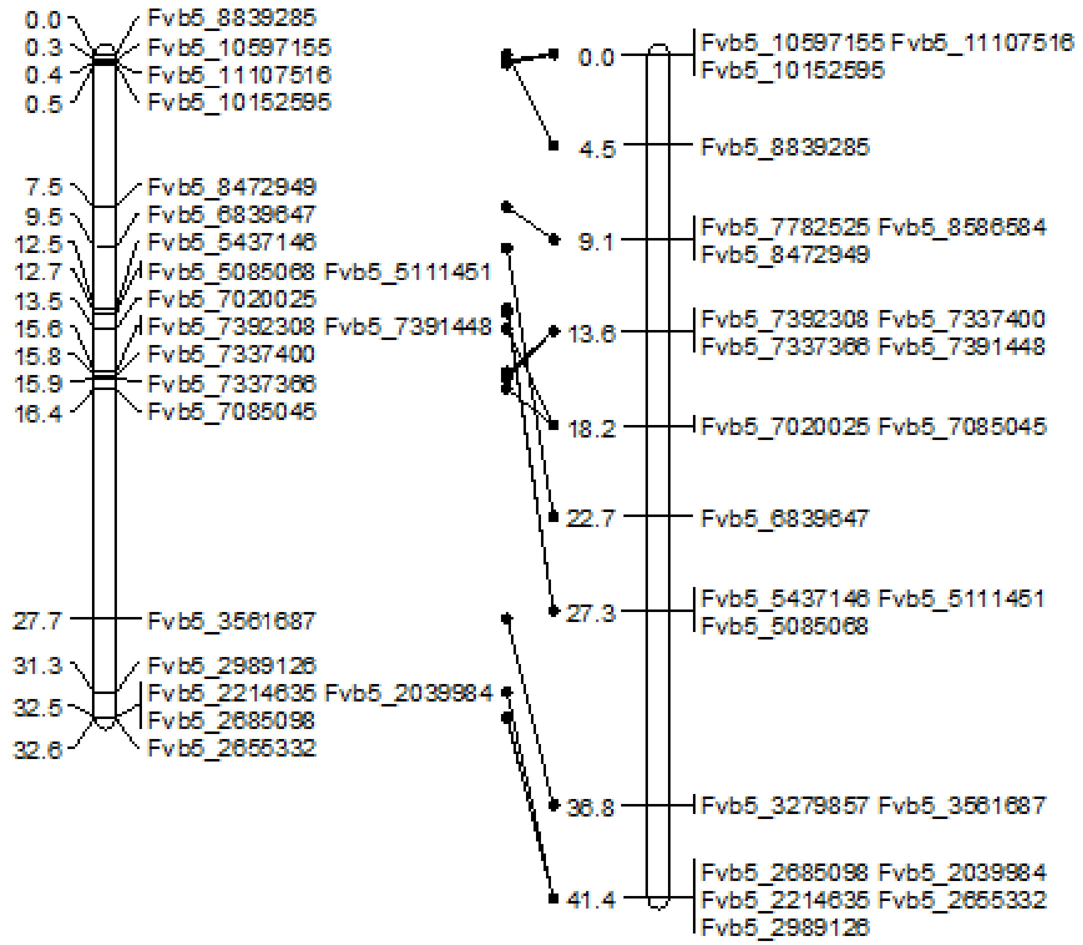

Korona_5

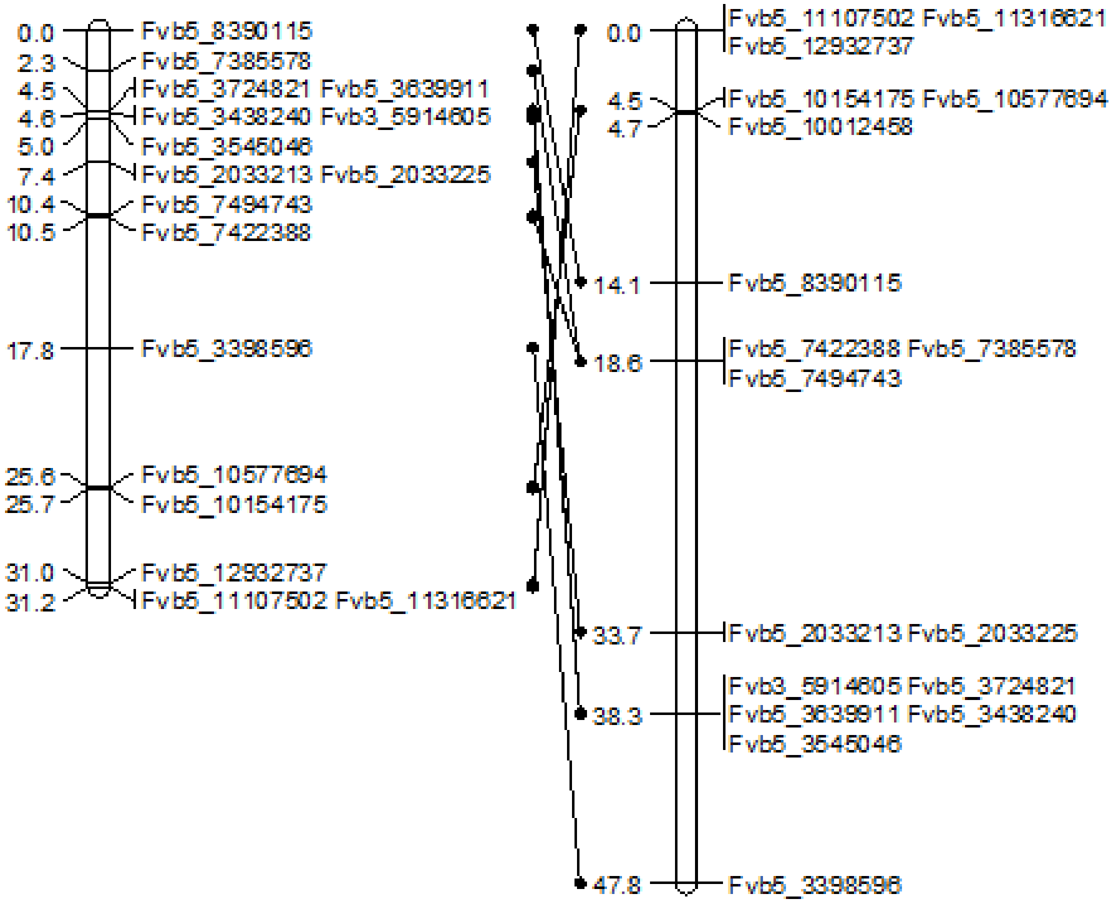

Fvb_5

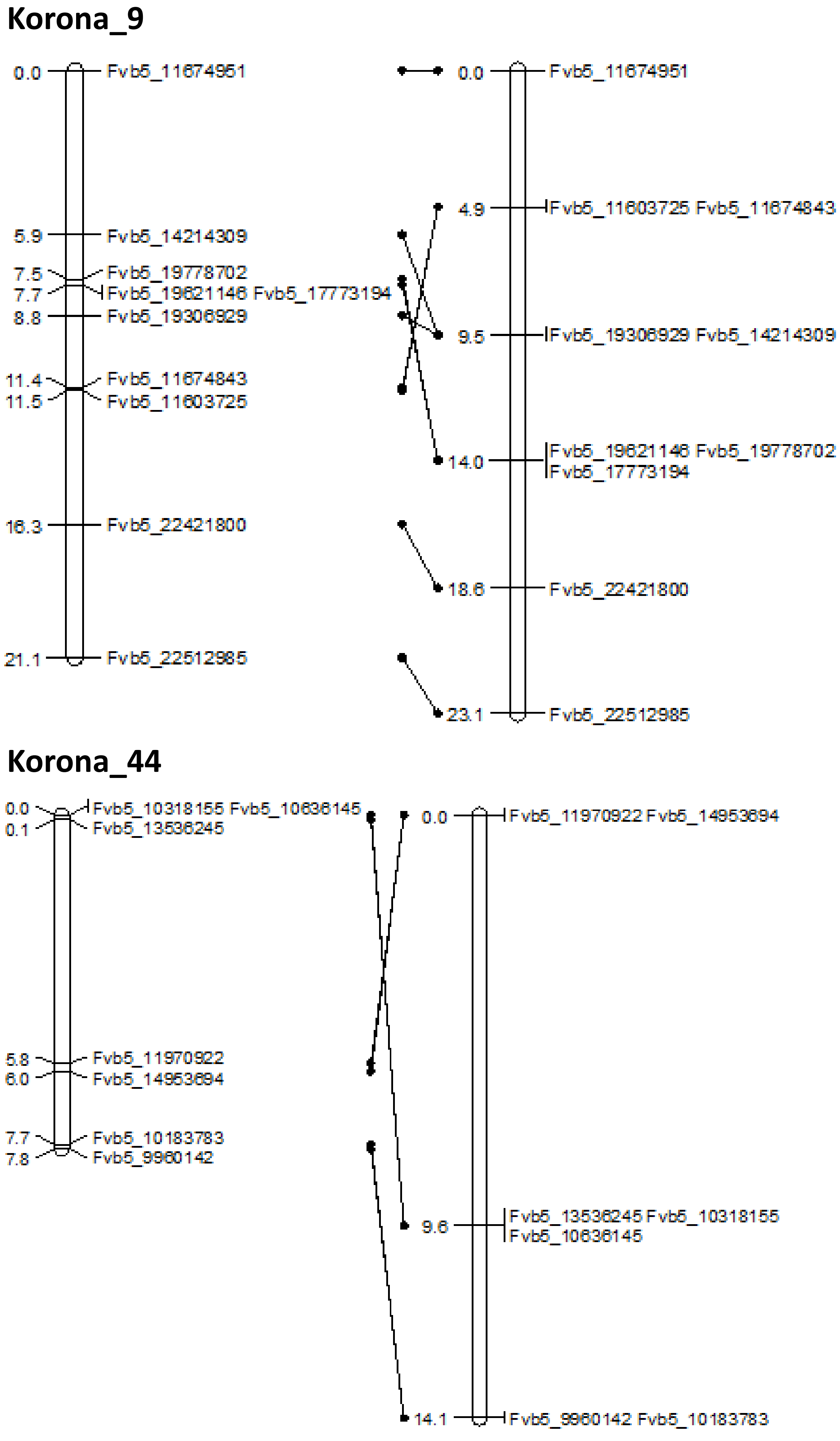
Korona_9
0.0
Fvb5_11674951
5.9
Fvb5_14214309
7.5
Fvb5_19778702
7.7
Fvb5_19621146 Fvb5_17773194
8.8
Fvb5_19306929
11.4
Fvb5_11674843
11.5
Fvb5_11603725
16.3
Fvb5_22421800
21.1
Fvb5_22512985
0.0
Fvb5_11674951
4.9
Fvb5_11603725 Fvb5_11674843
9.5
Fvb5_19306929 Fvb5_14214309
14.0
Fvb5_19621146 Fvb5_19778702
Fvb5_17773194
18.6
Fvb5_22421800
23.1
Fvb5_22512985
Korona_44
0.0
Fvb5_10318155 Fvb5_10636145
0.1
Fvb5_13536245
5.8
Fvb5_11970922
6.0
Fvb5_14953694
7.7
Fvb5_10183783
7.8
Fvb5_9960142
0.0
Fvb5_11970922 Fvb5_14953694
9.6
Fvb5_13536245 Fvb5_10318155
Fvb5_10636145
14.1
Fvb5_9960142 Fvb5_10183783

Fvb_5

Korona_47

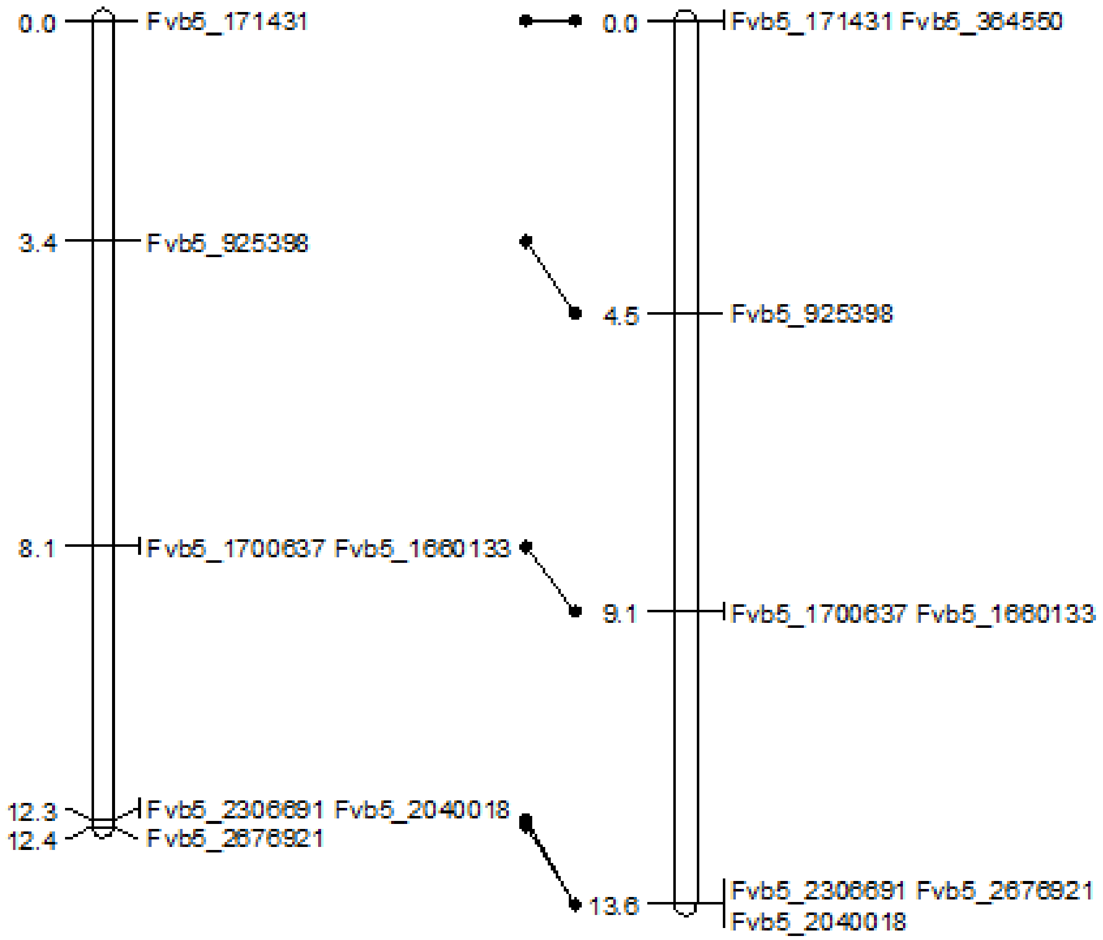

Korona_55

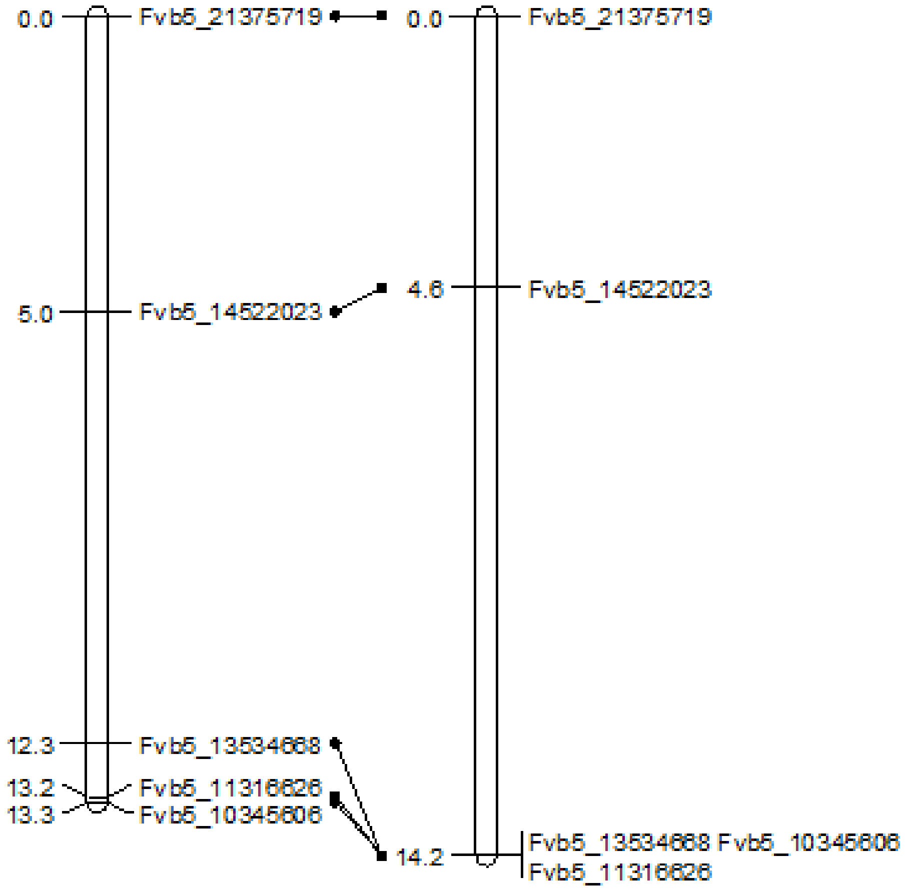

Fvb_6

Holiday_2

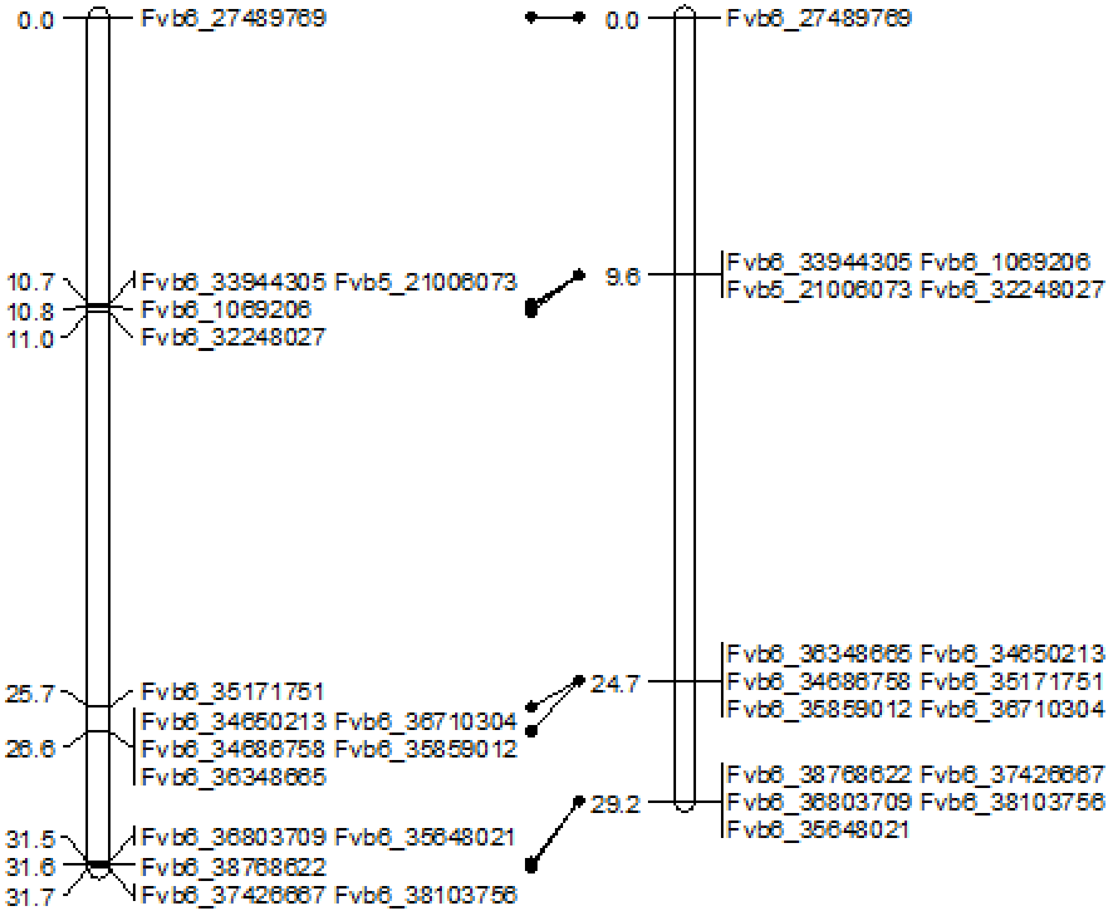

Holiday_3

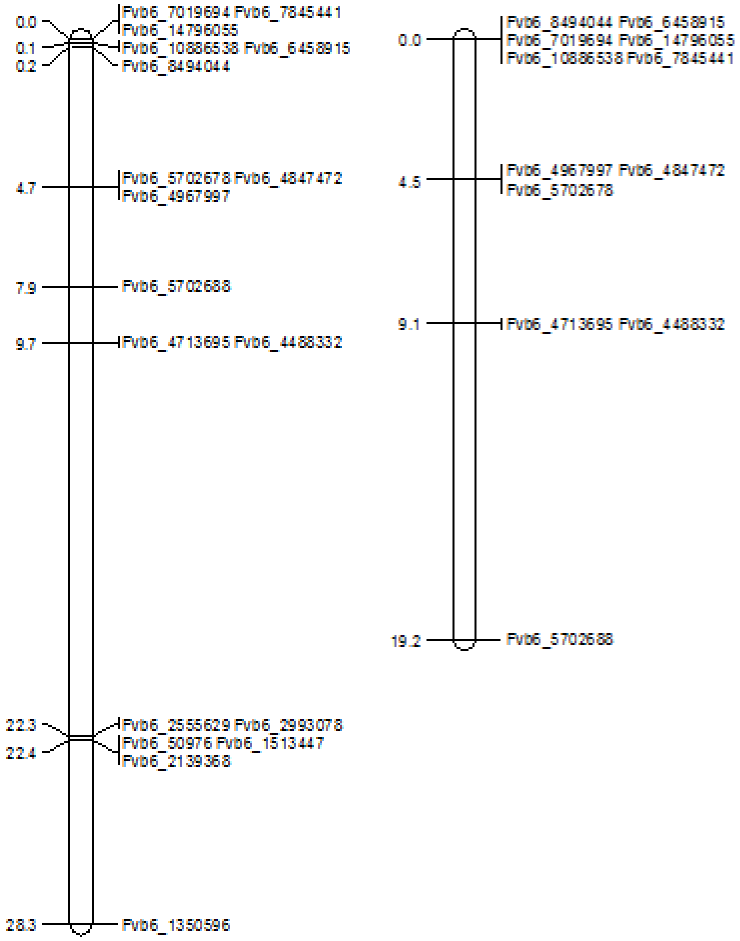

Fvb_6

Holiday_5

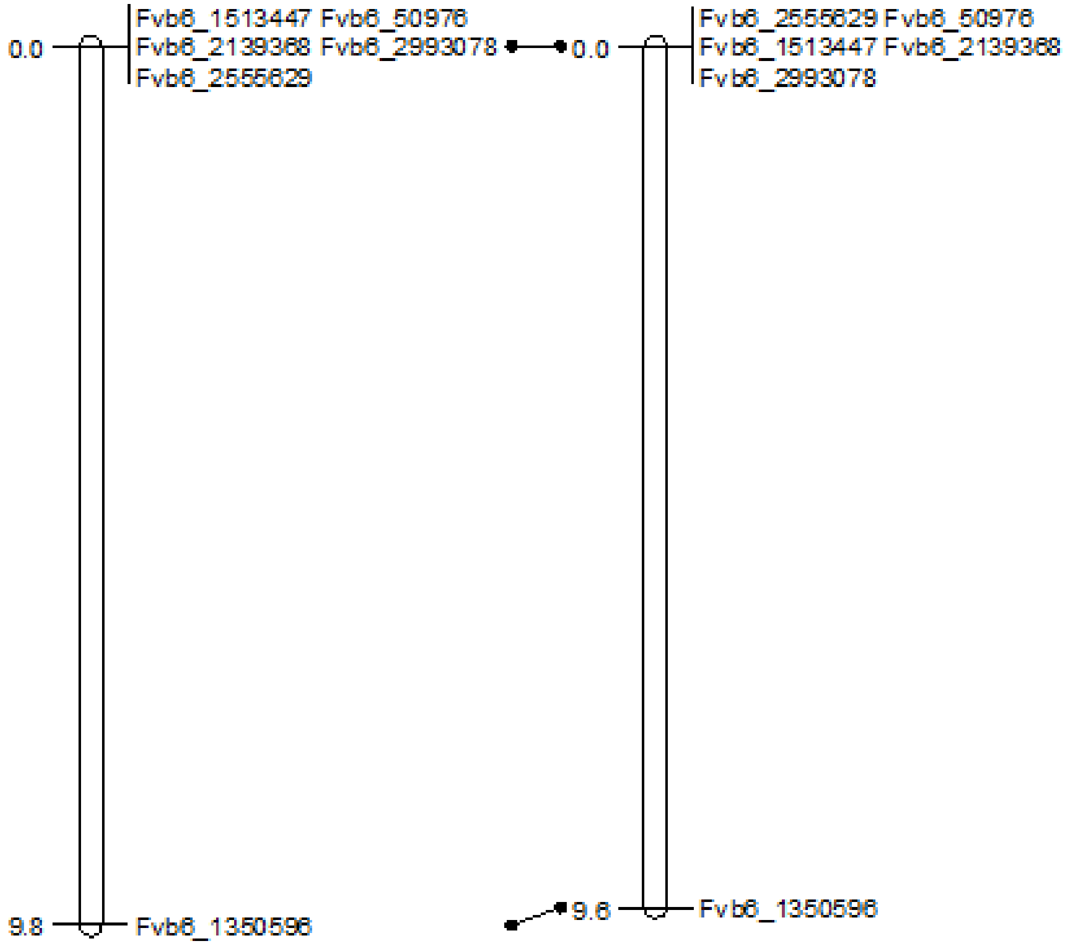

Holiday_8

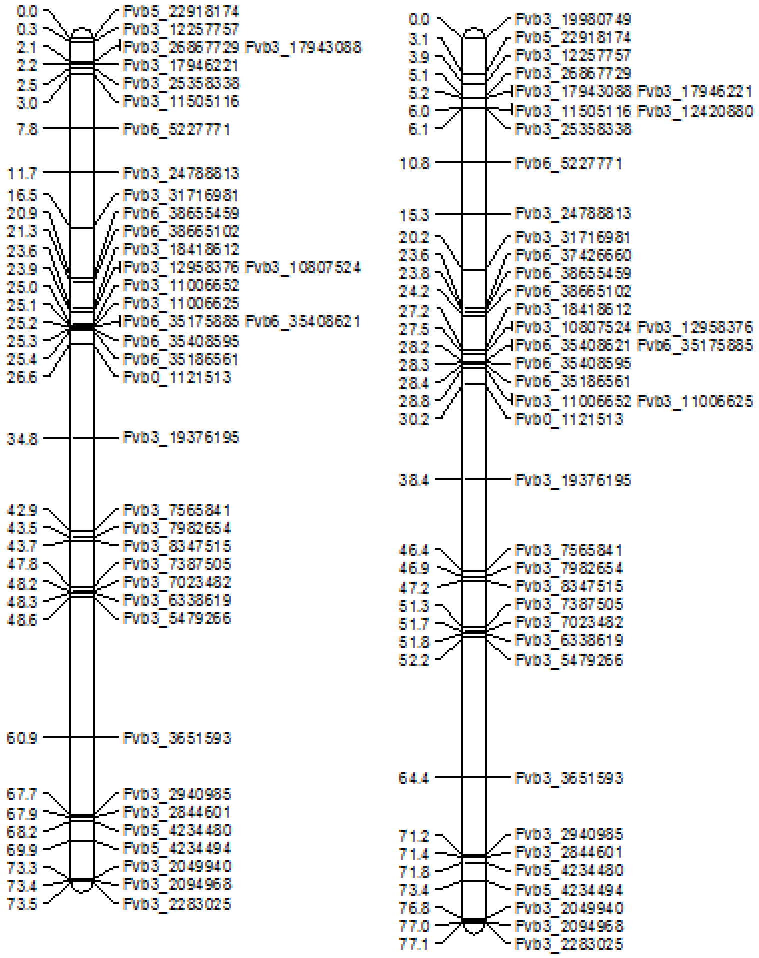

Fvb_6

Holiday_14

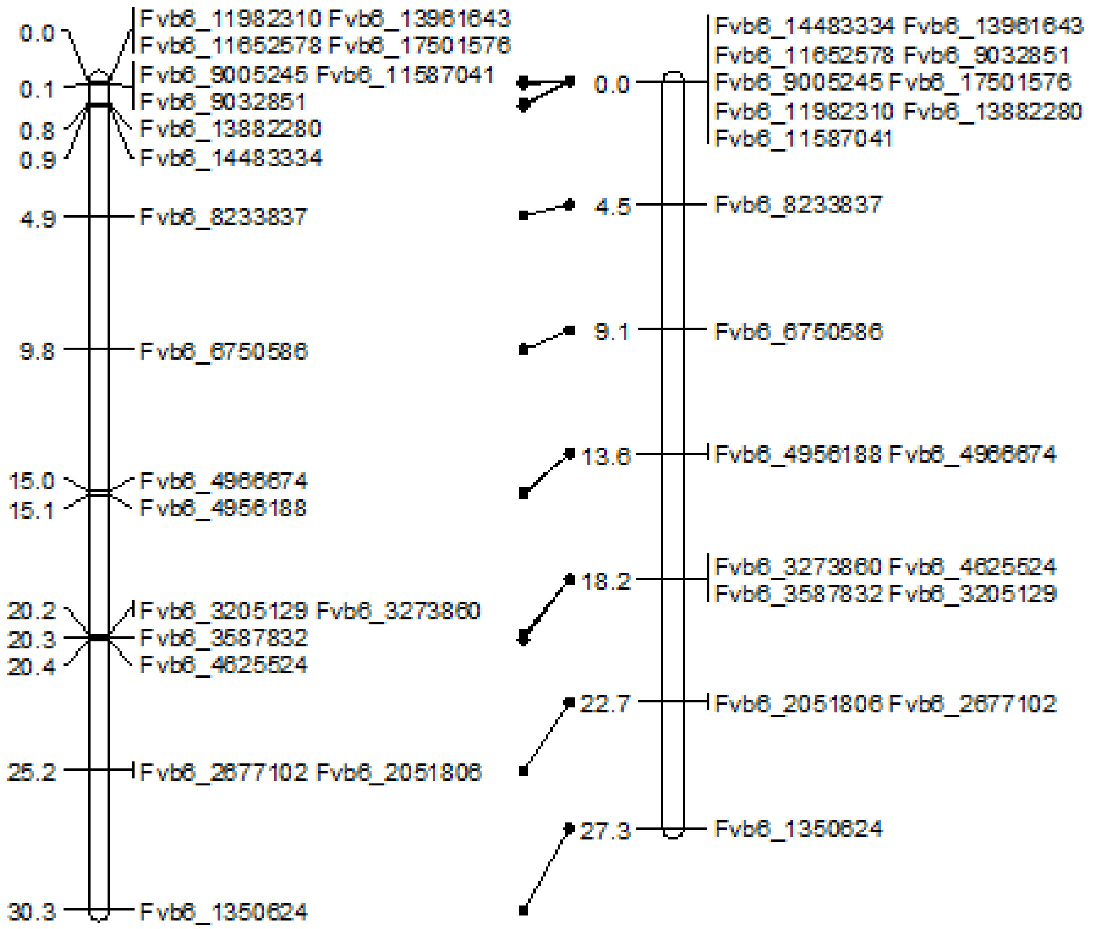

Holiday_21

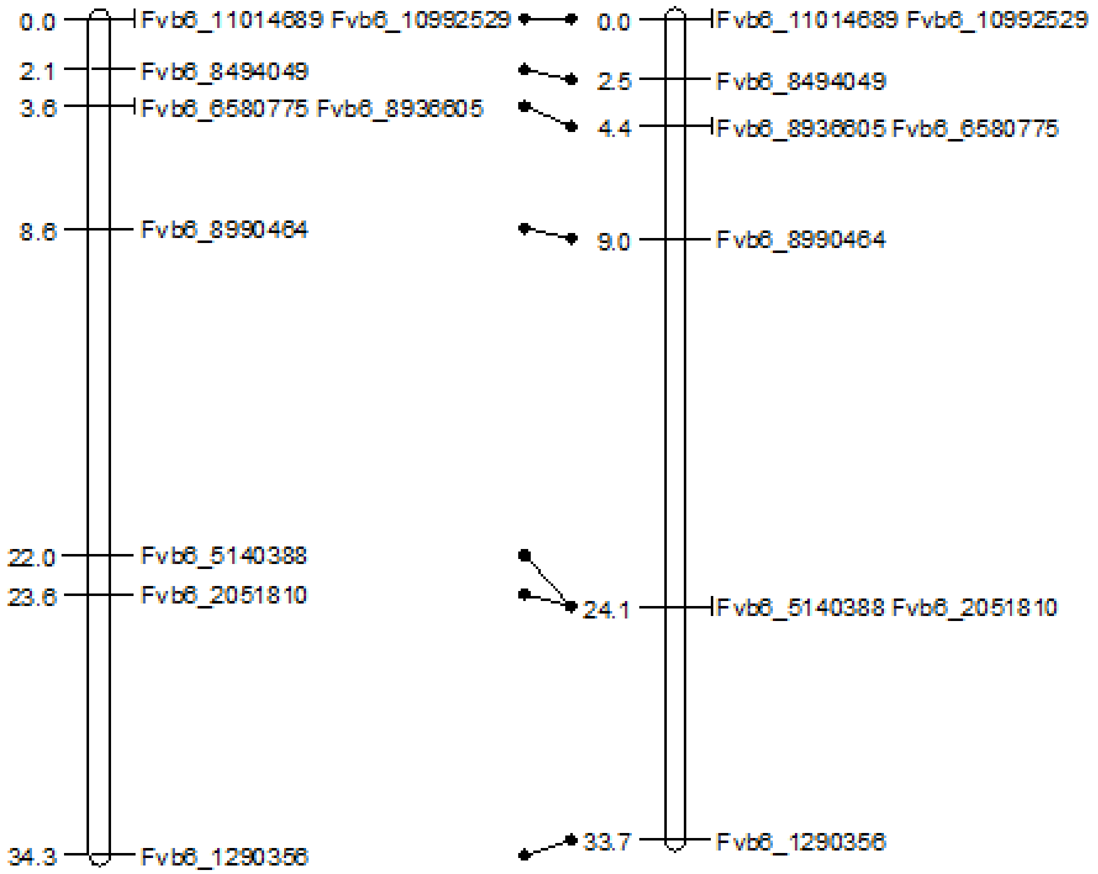

Fvb_6

Korona_20

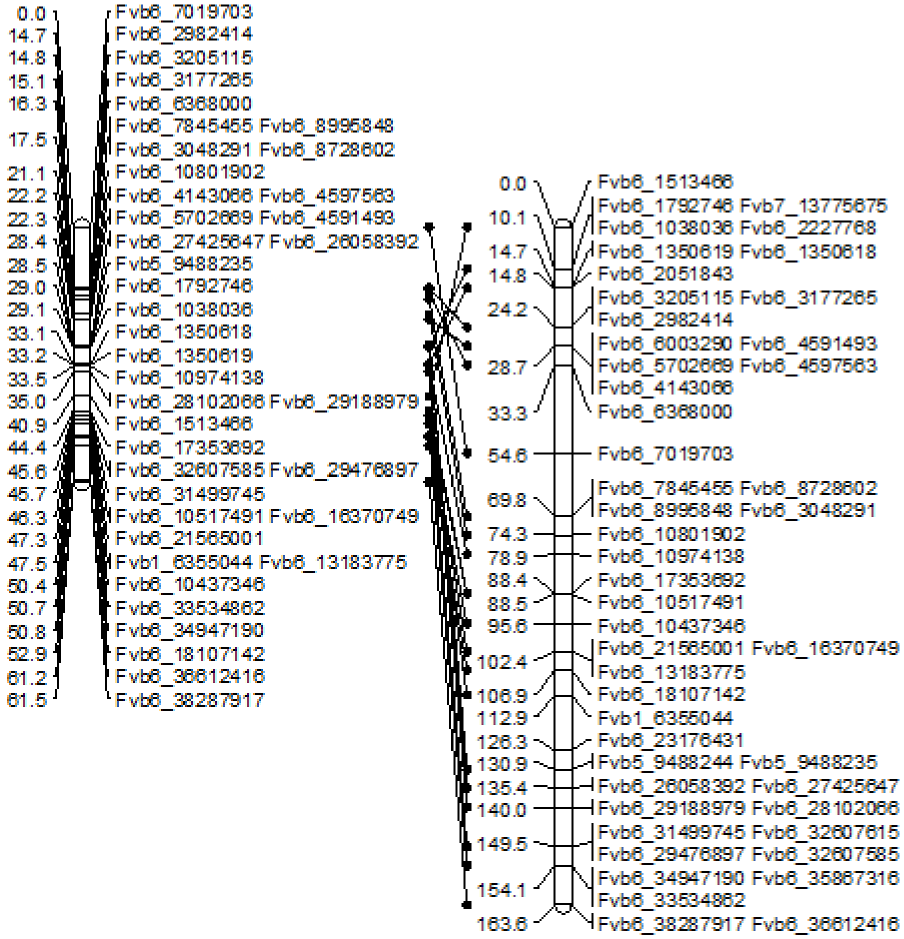

Korona_28

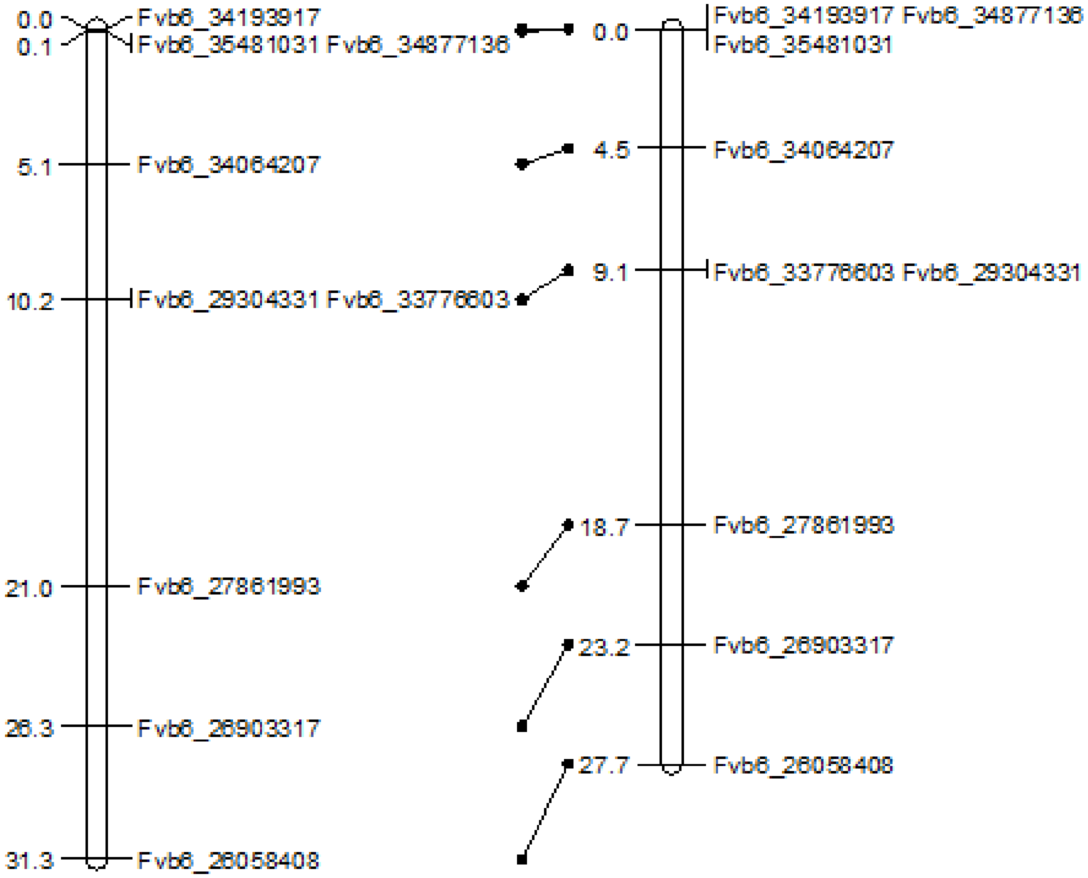

Fvb_6

Korona_38

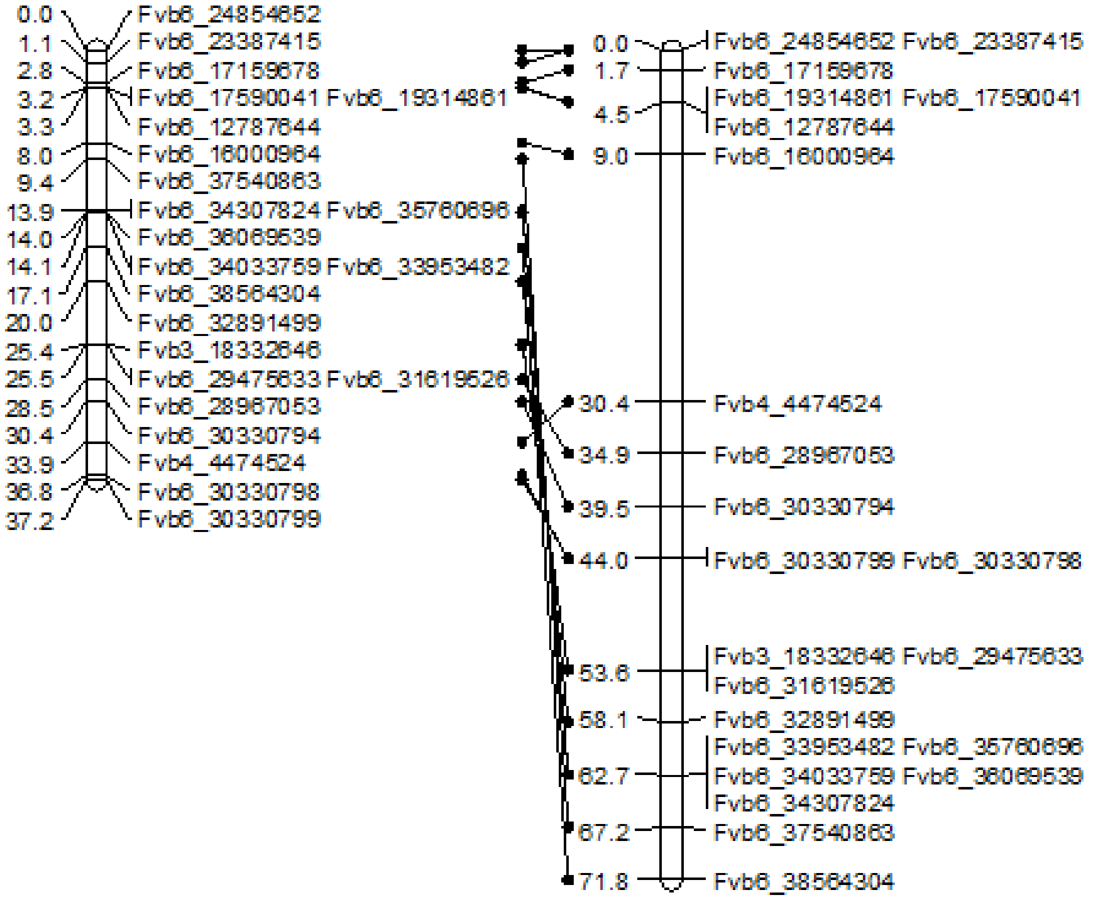

Korona_42

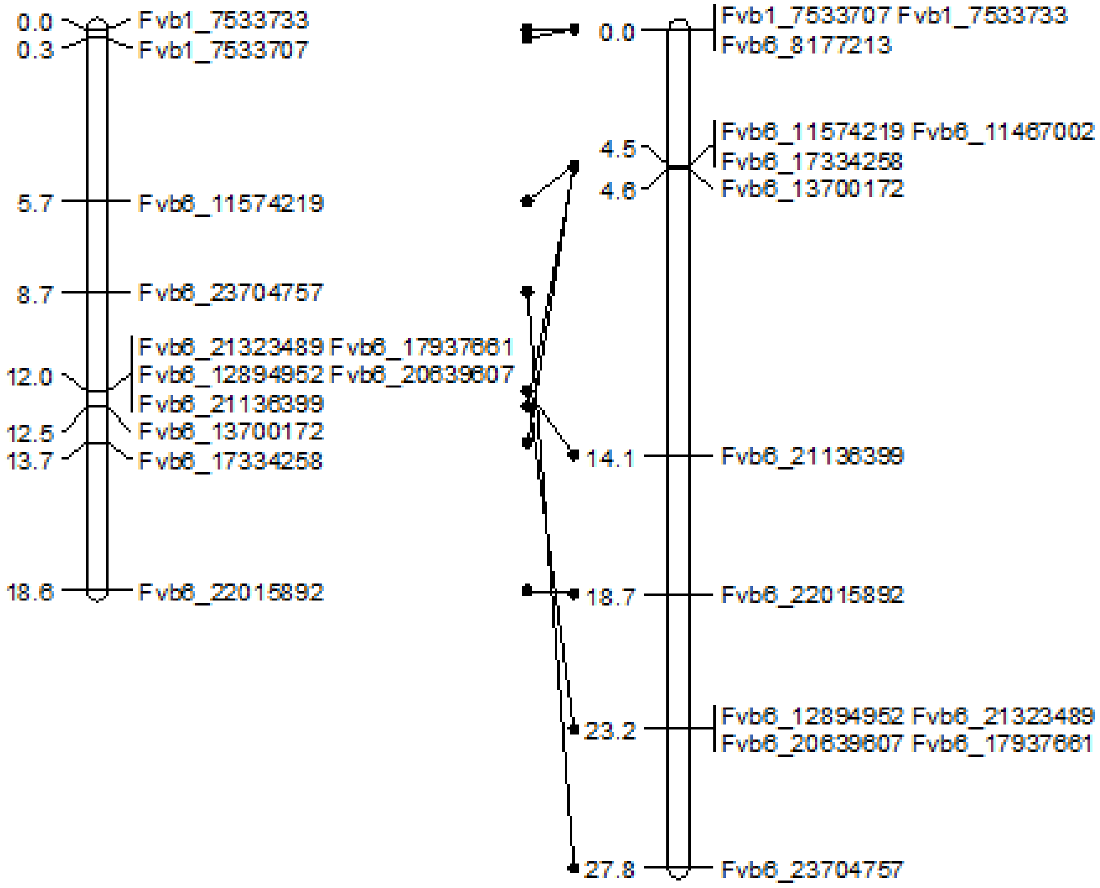

Fvb_7

Holiday_18

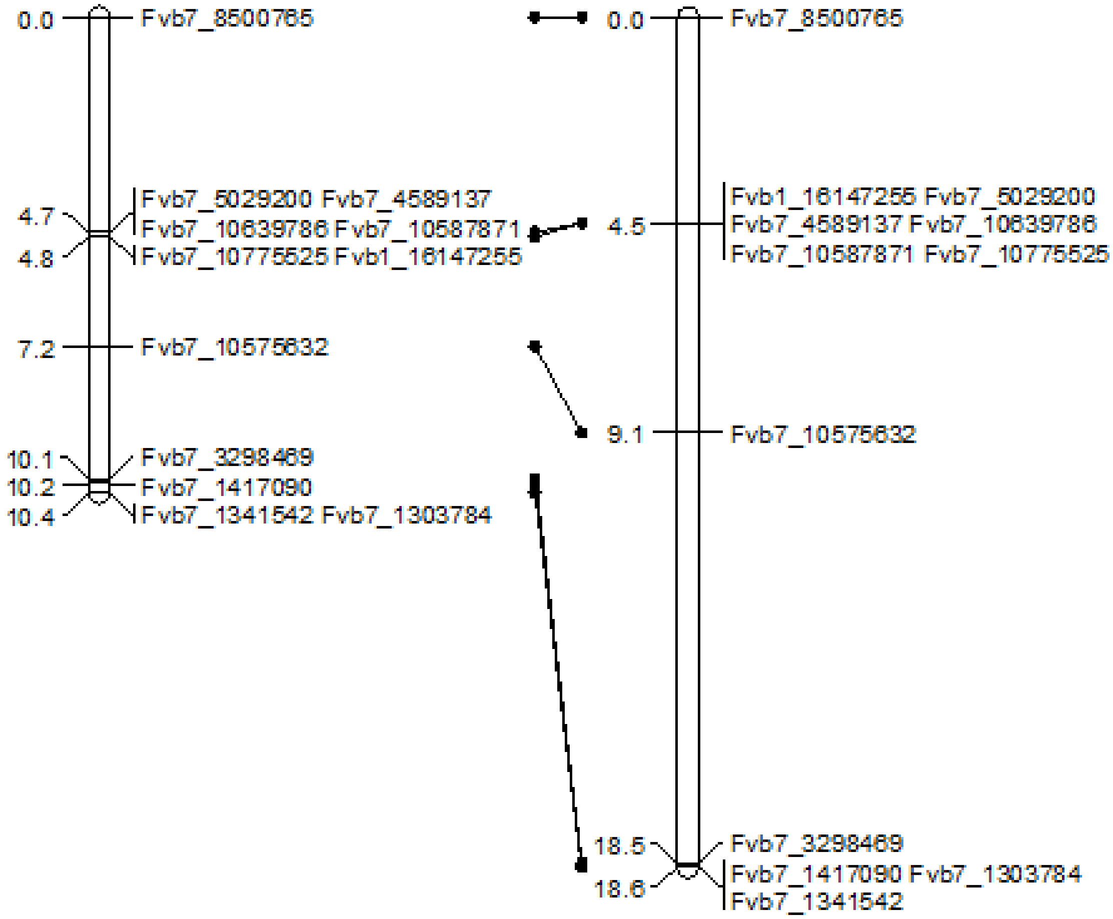

Holiday_26

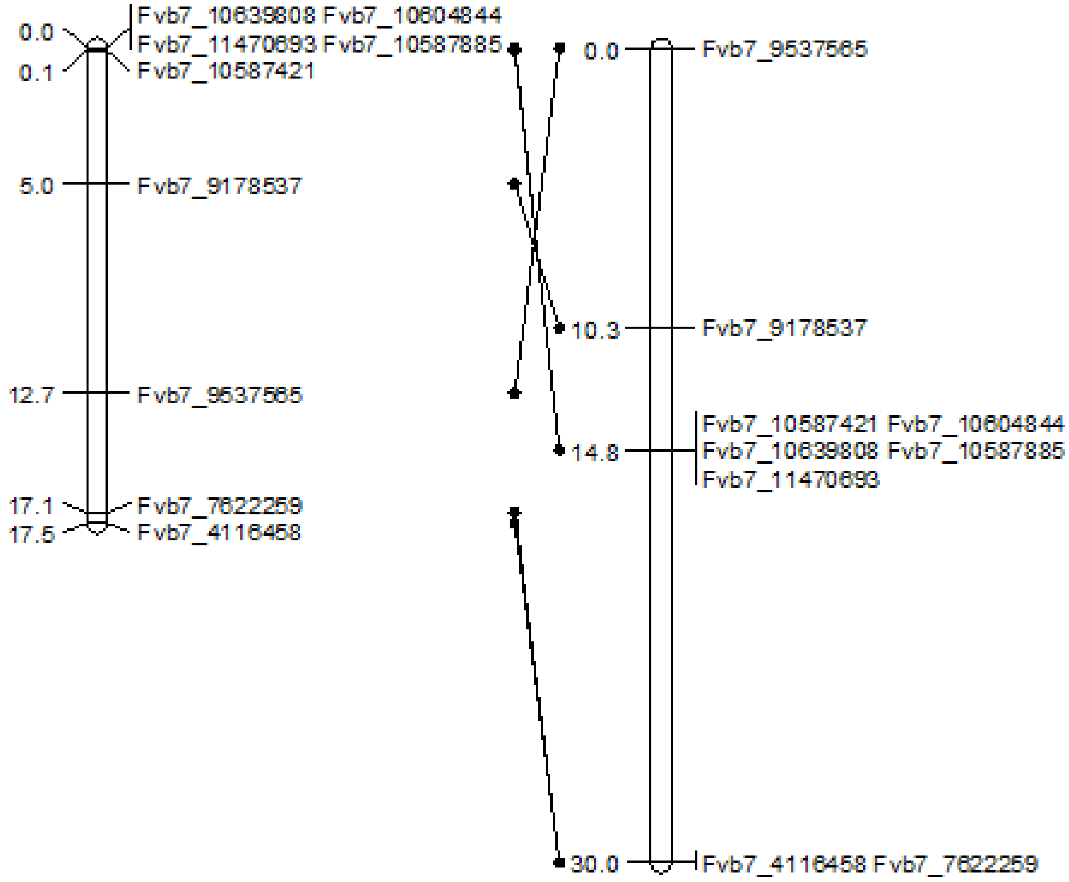

Fvb_7

Korona_7

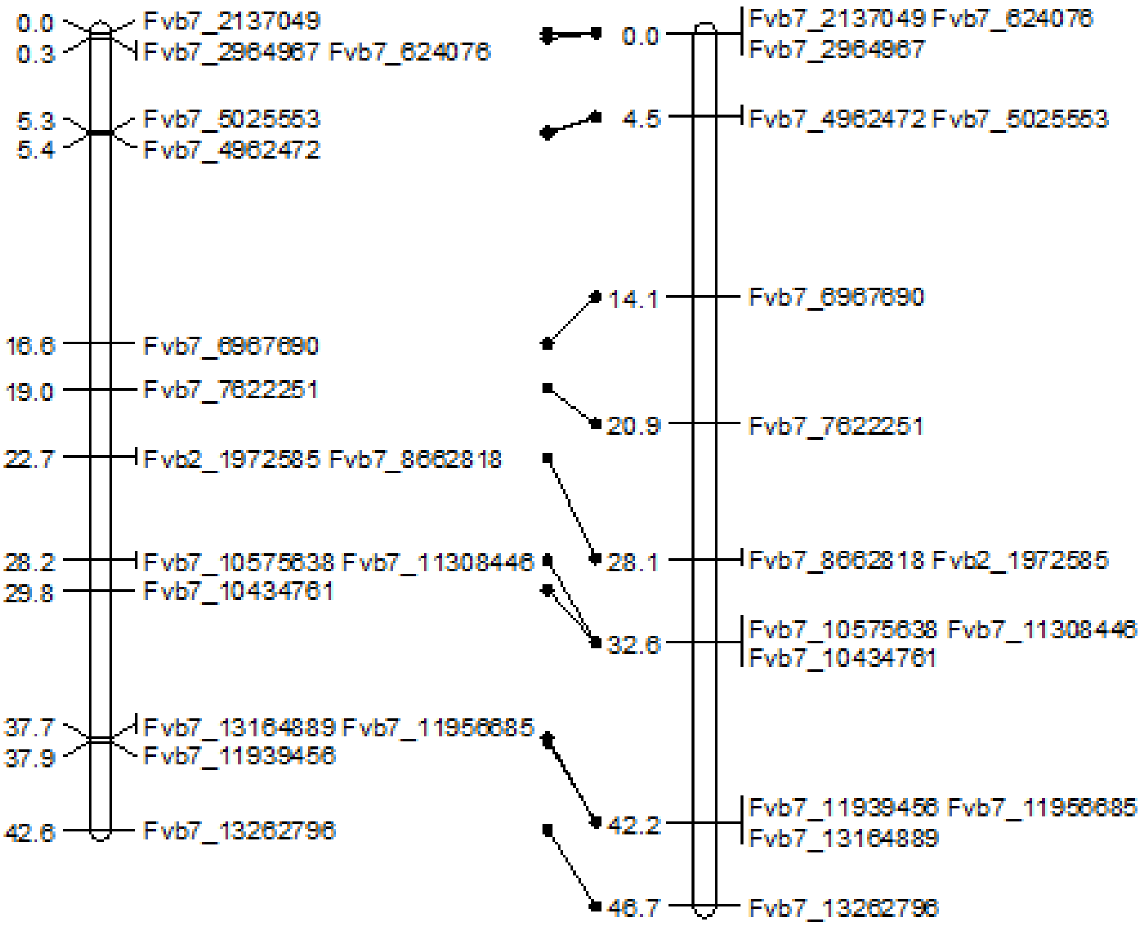

Korona_50

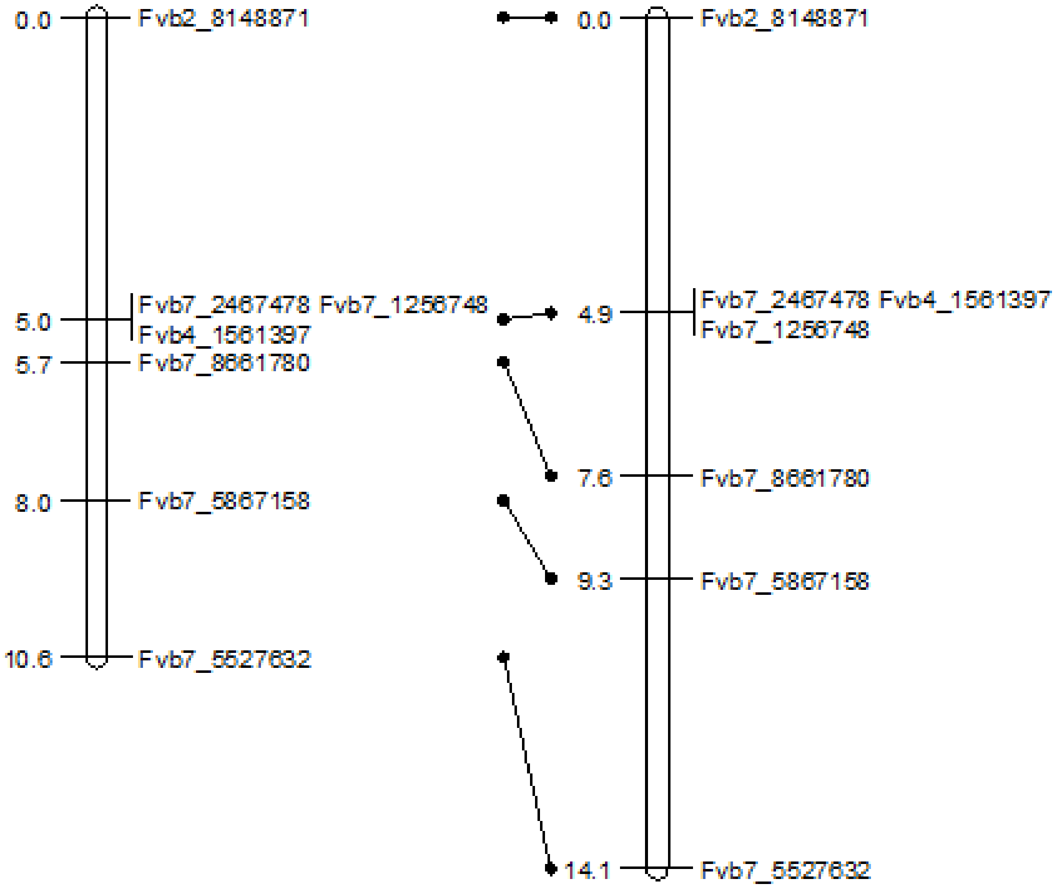

Fvb_7

Korona_53

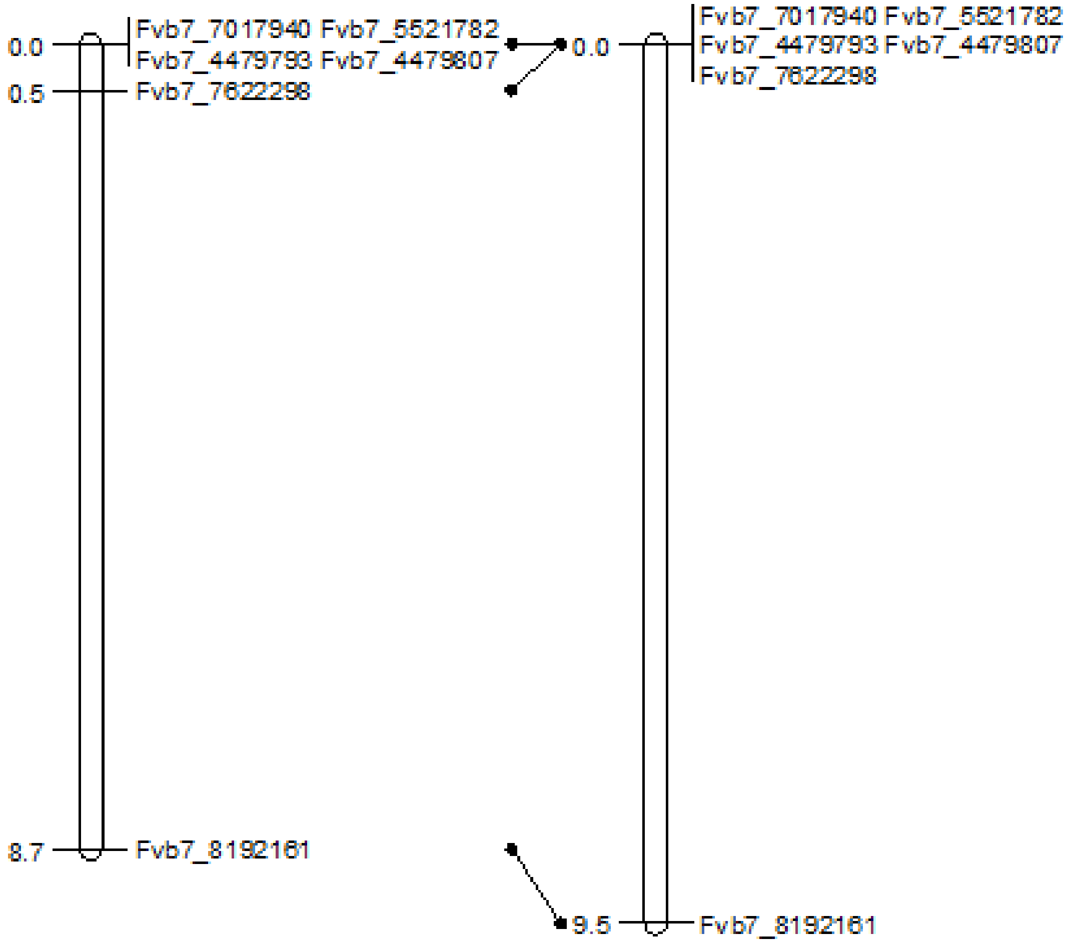

Supplement: File S1 [file peerj-05-3731-s002.pdf]
