# Supplementary material for: Genotyping-by-sequencing enables linkage mapping in three octoploid cultivated strawberry families
Source: PeerJ. 2017 Aug 30;5:e3731. doi: 10.7717/peerj.3731 (PMC5581533; doi:10.7717/peerj.3731)

Fvb_1

Tribute_6

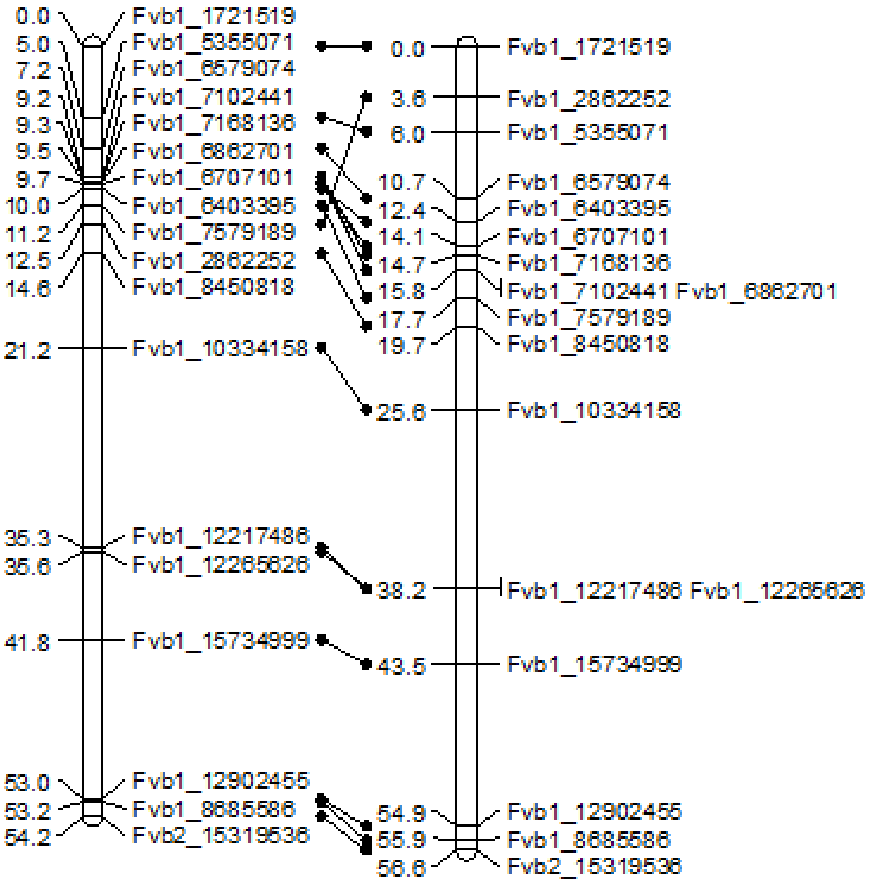

Tribute_8

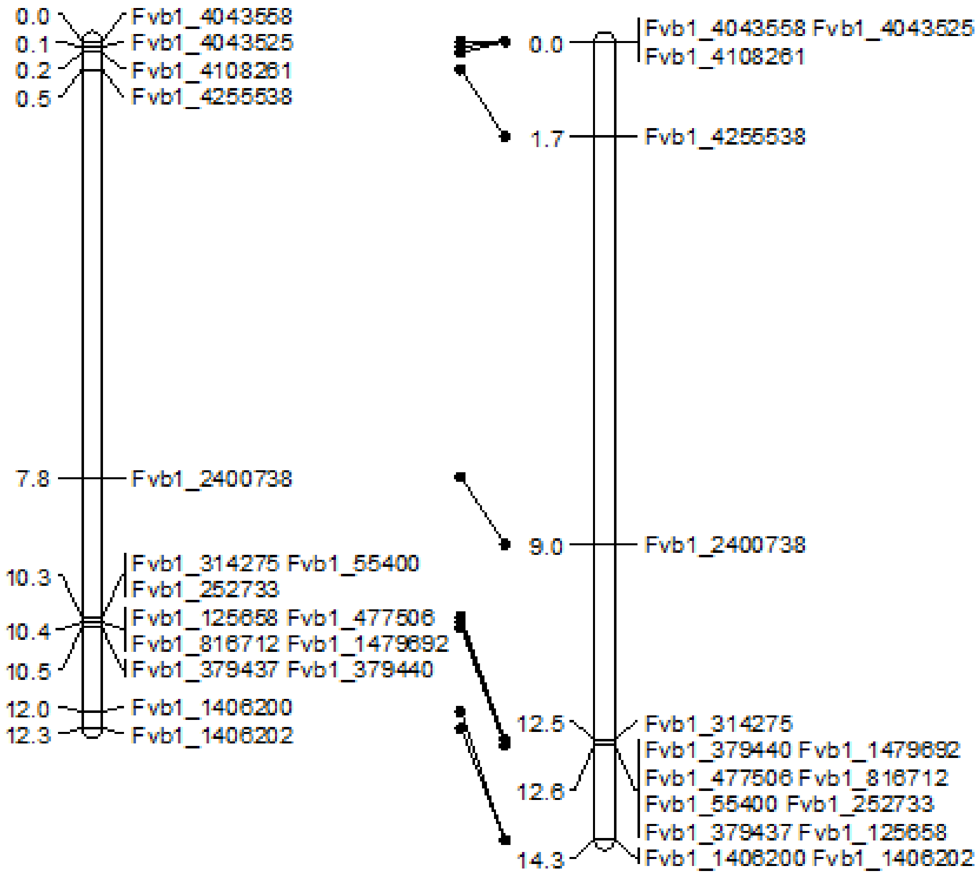

Fvb_1

Tribute_17

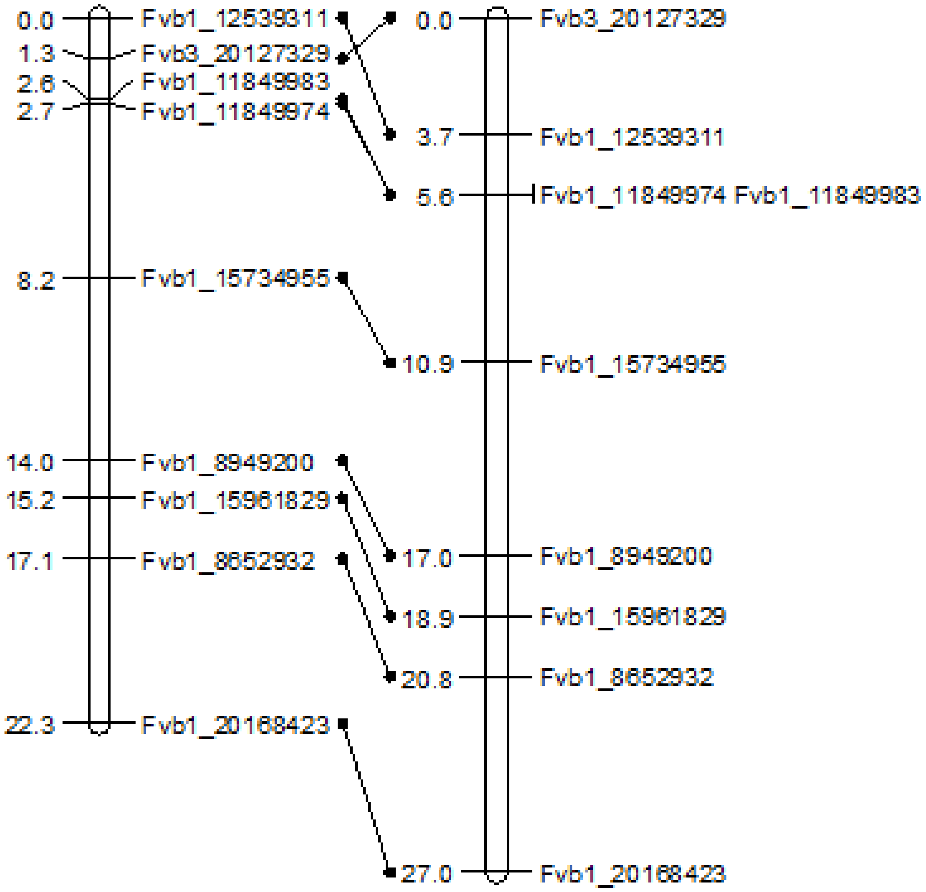

Tribute_19

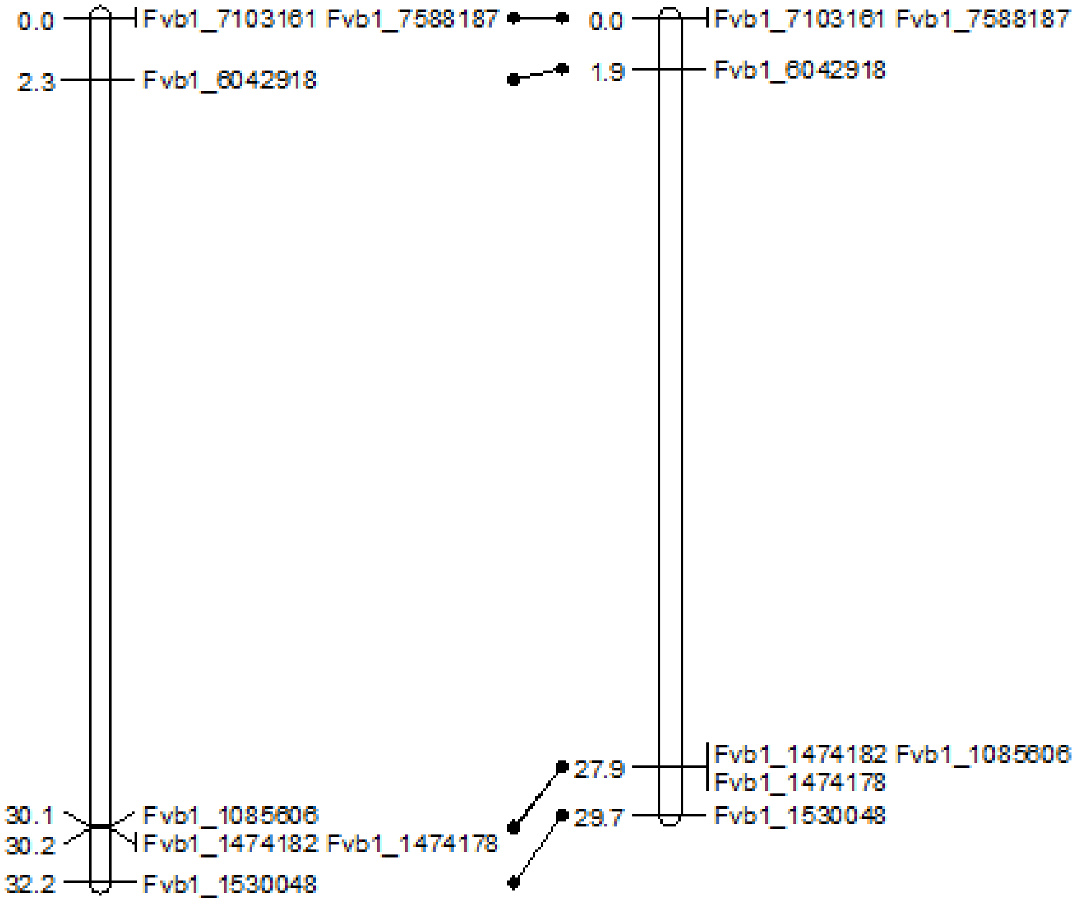

Fvb_1

Honeoye_15

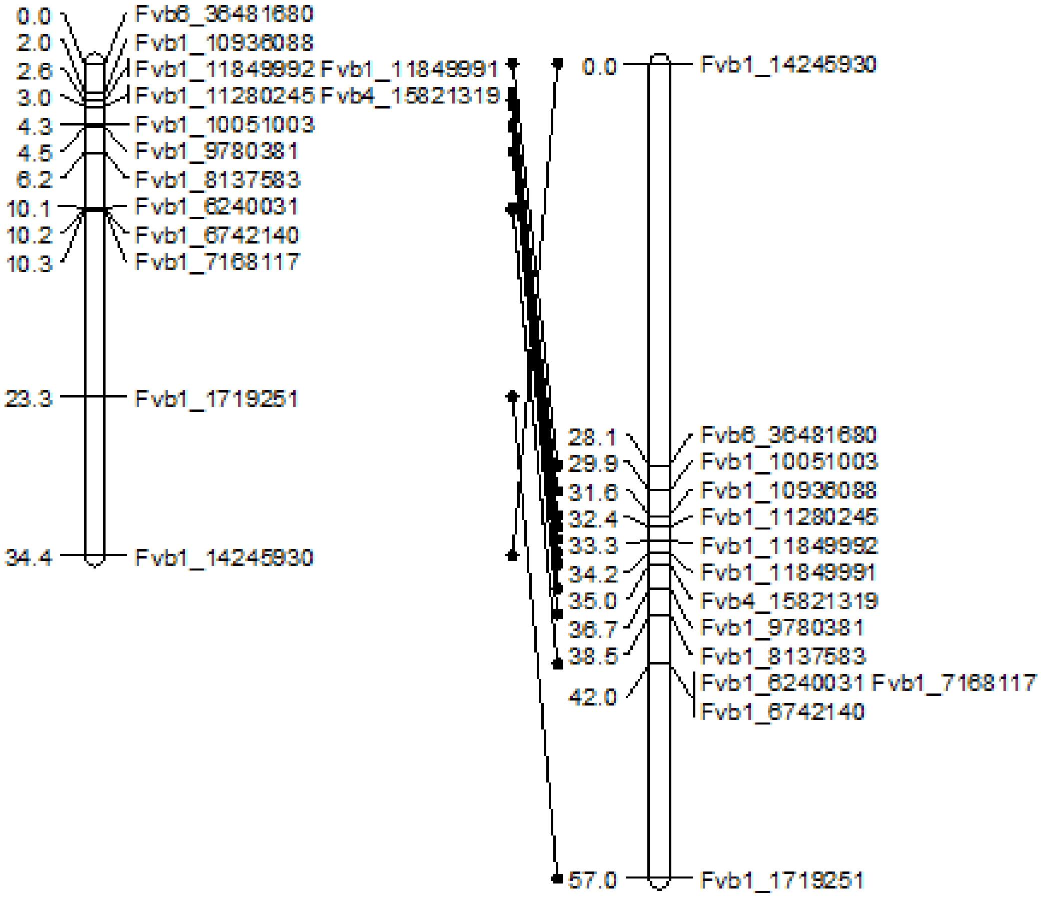

Honeoye_21

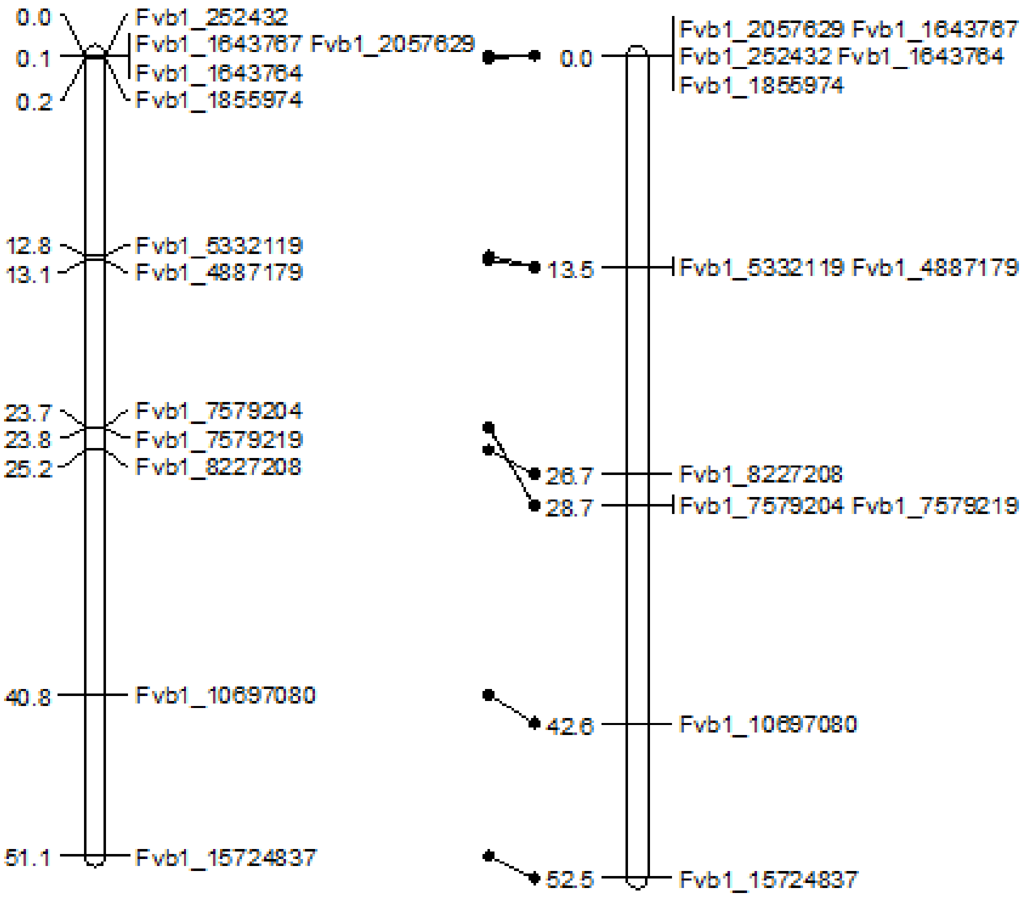

Fvb_1

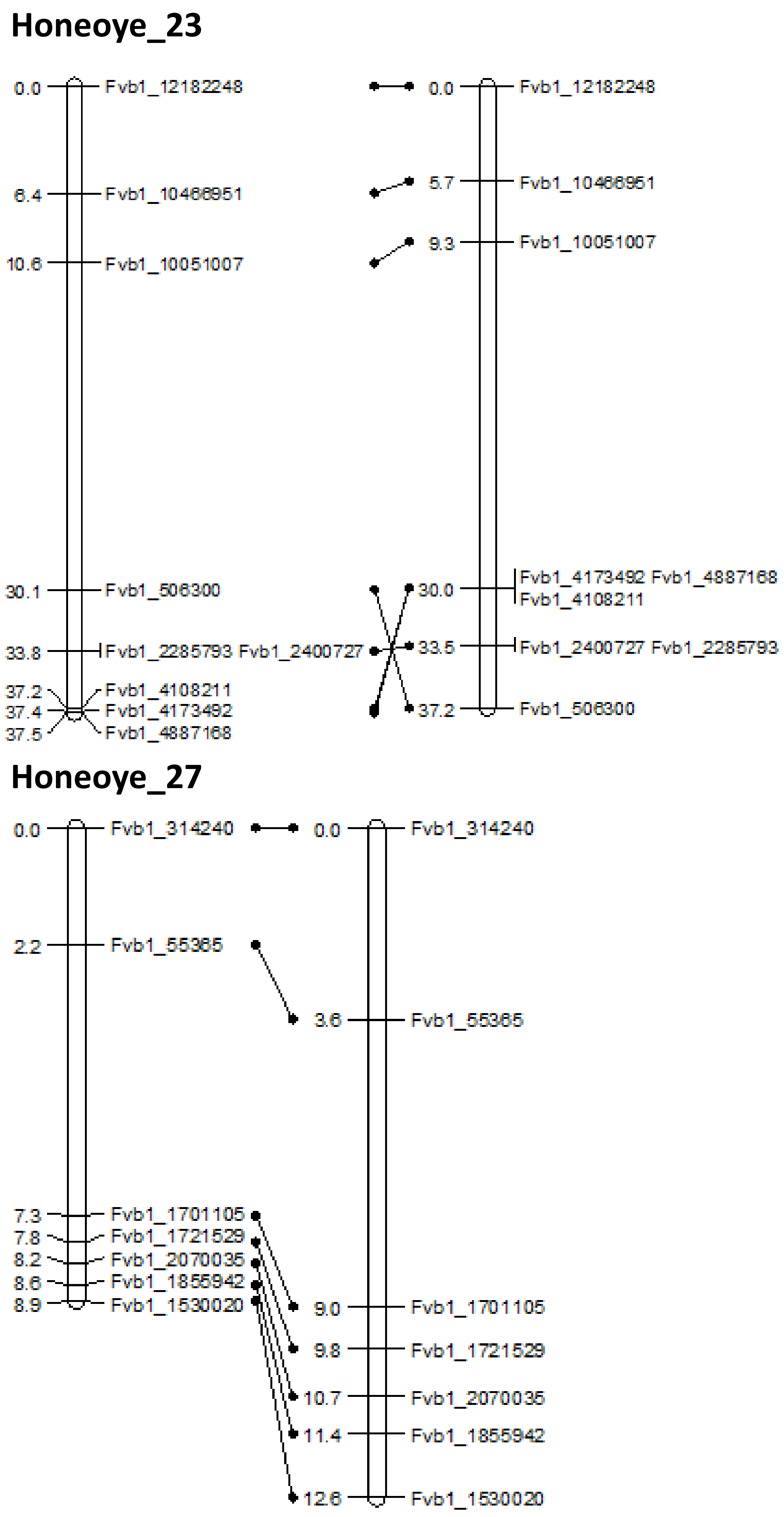

Honeoye_23
0.0
Fvb1_12182248
6.4
Fvb1_10466951
10.6
Fvb1_10051007
30.1
Fvb1_506300
33.8
Fvb1_2285793 Fvb1_2400727
37.2
37.4
37.5
Fvb1_4108211
Fvb1_4173492
Fvb1_4887168
0.0
Fvb1_12182248
5.7
Fvb1_10466951
9.3
Fvb1_10051007
30.0
Fvb1_4173492 Fvb1_4887168
Fvb1_4108211
33.5
Fvb1_2400727 Fvb1_2285793
37.2
Fvb1_506300
Honeoye_27
0.0
Fvb1_314240
2.2
Fvb1_55365
7.3
7.8
8.2
8.6
8.9
Fvb1_1701105
Fvb1_1721529
Fvb1_2070035
Fvb1_1855942
Fvb1_1530020
0.0
Fvb1_314240
3.6
Fvb1_55365
9.0
Fvb1_1701105
9.8
Fvb1_1721529
10.7
Fvb1_2070035
11.4
Fvb1_1855942
12.6
Fvb1_1530020

Fvb_2

Tribute_13

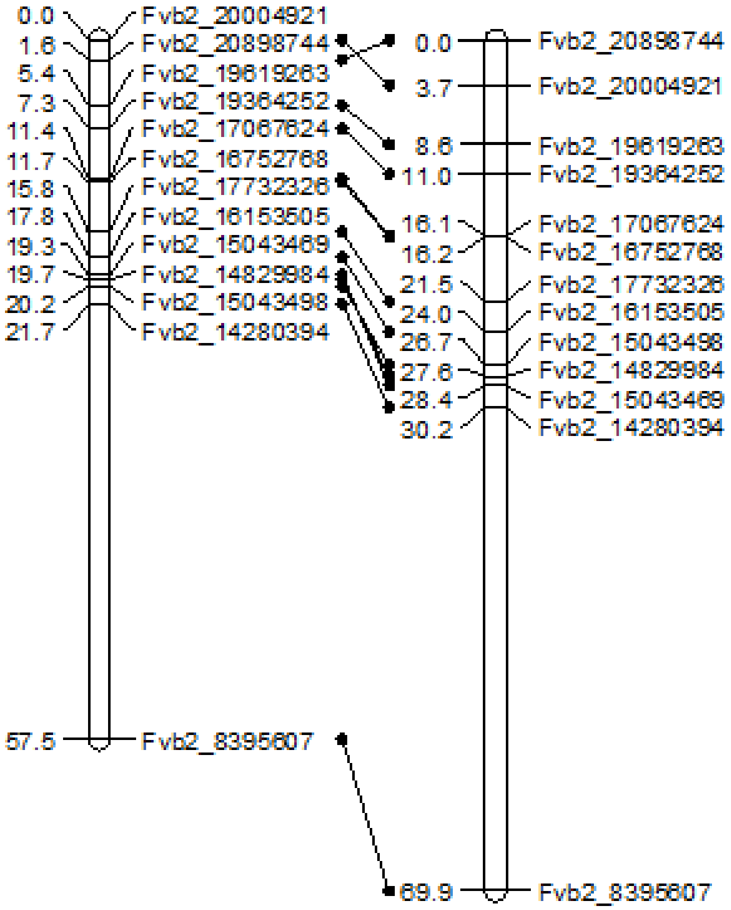

Tribute_23

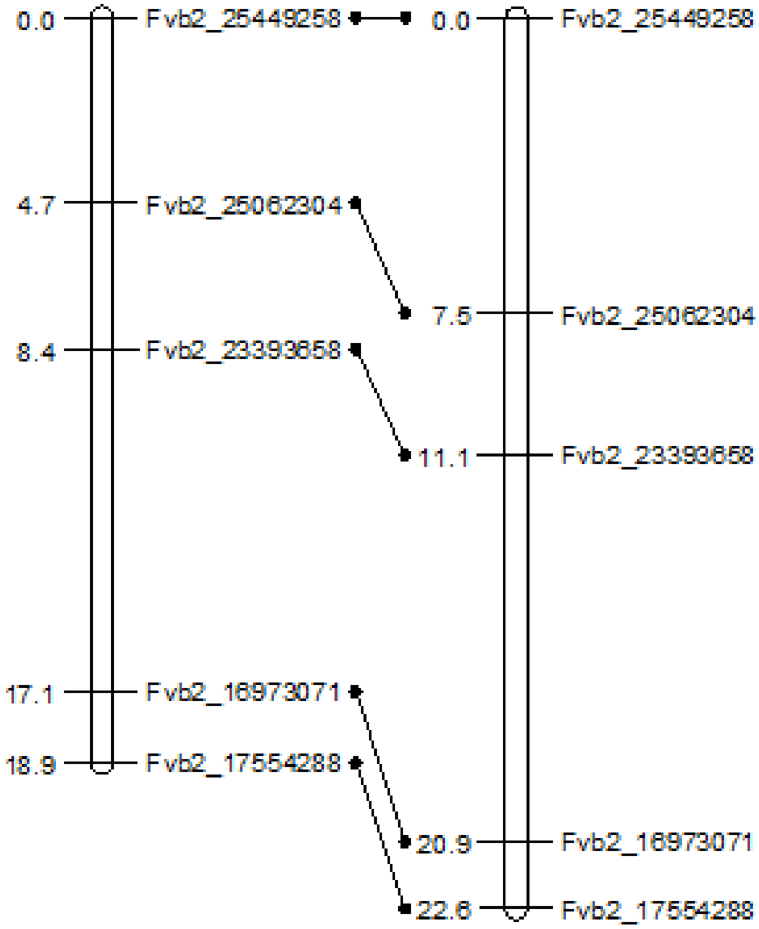

Fvb_2

Tribute_26

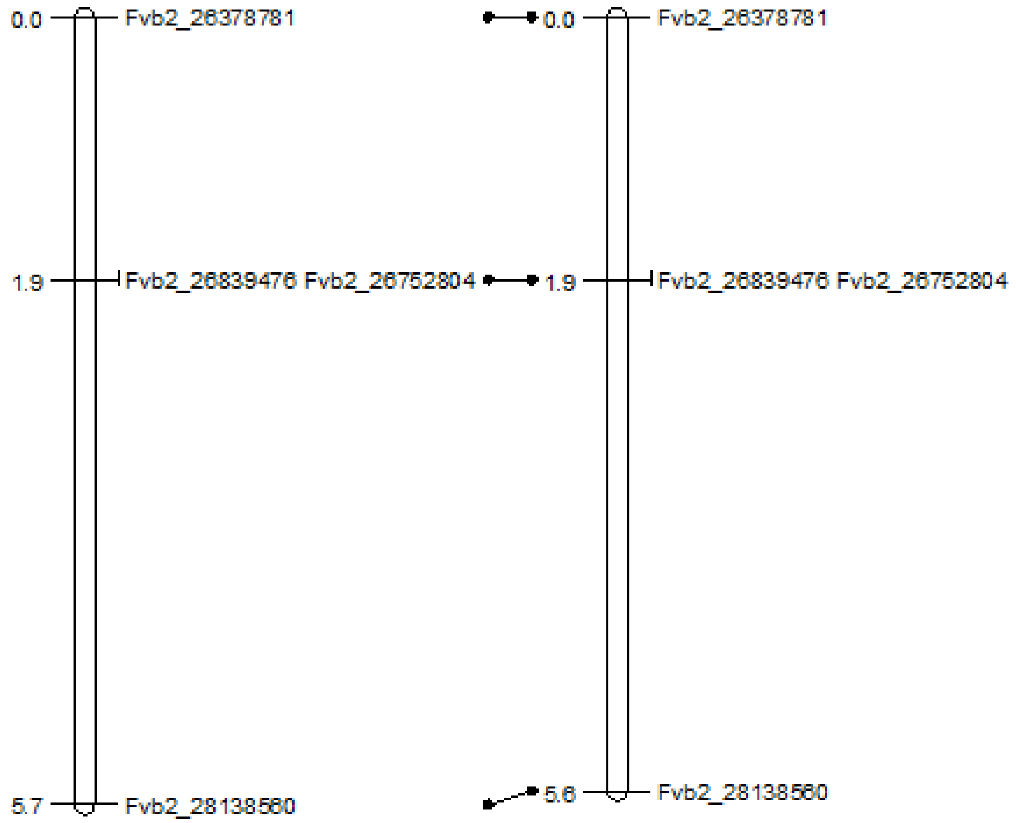

Tribute_28

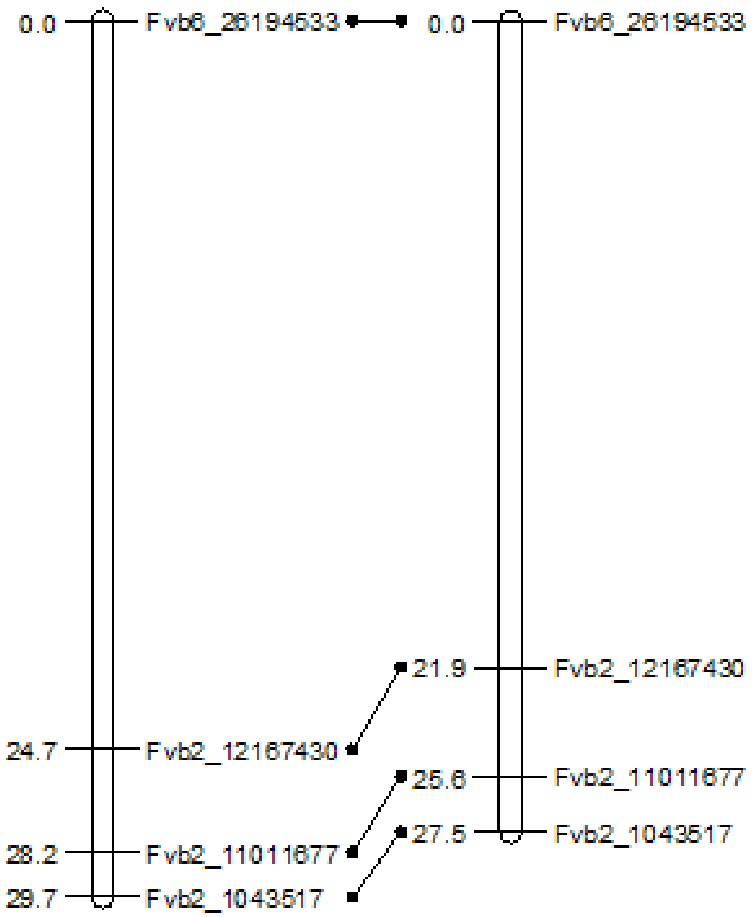

Fvb_2

Honeoye_4

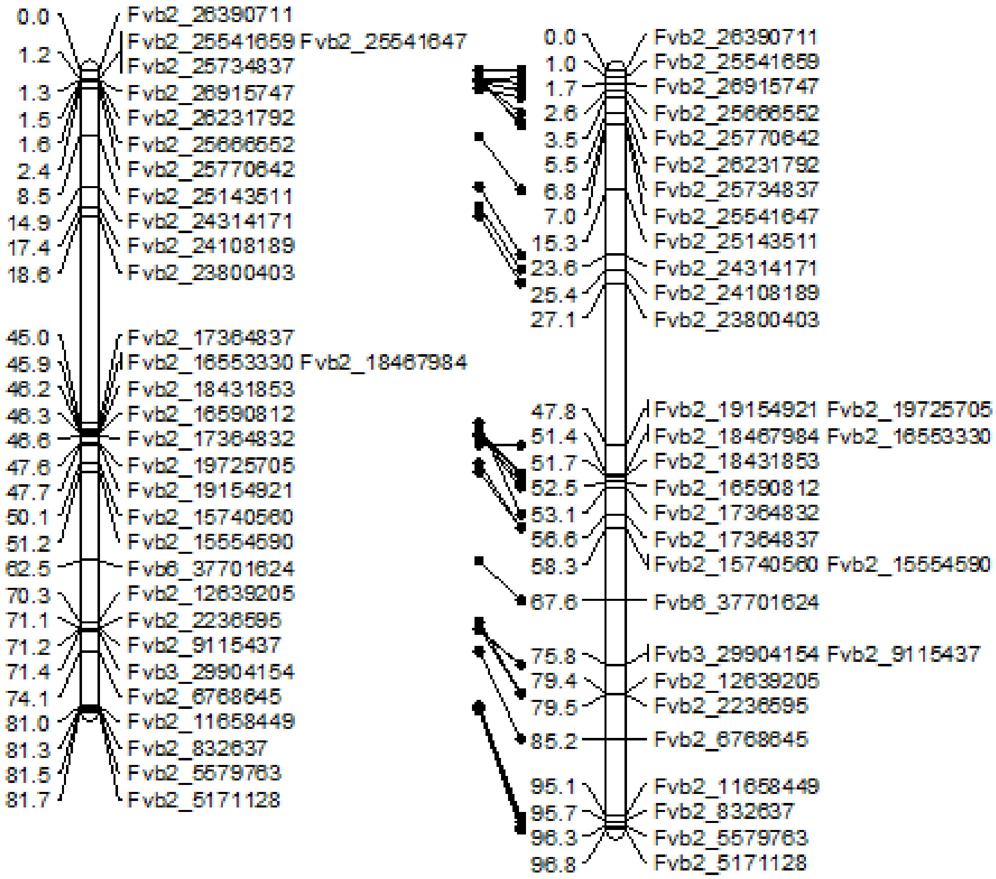

Honeoye_7

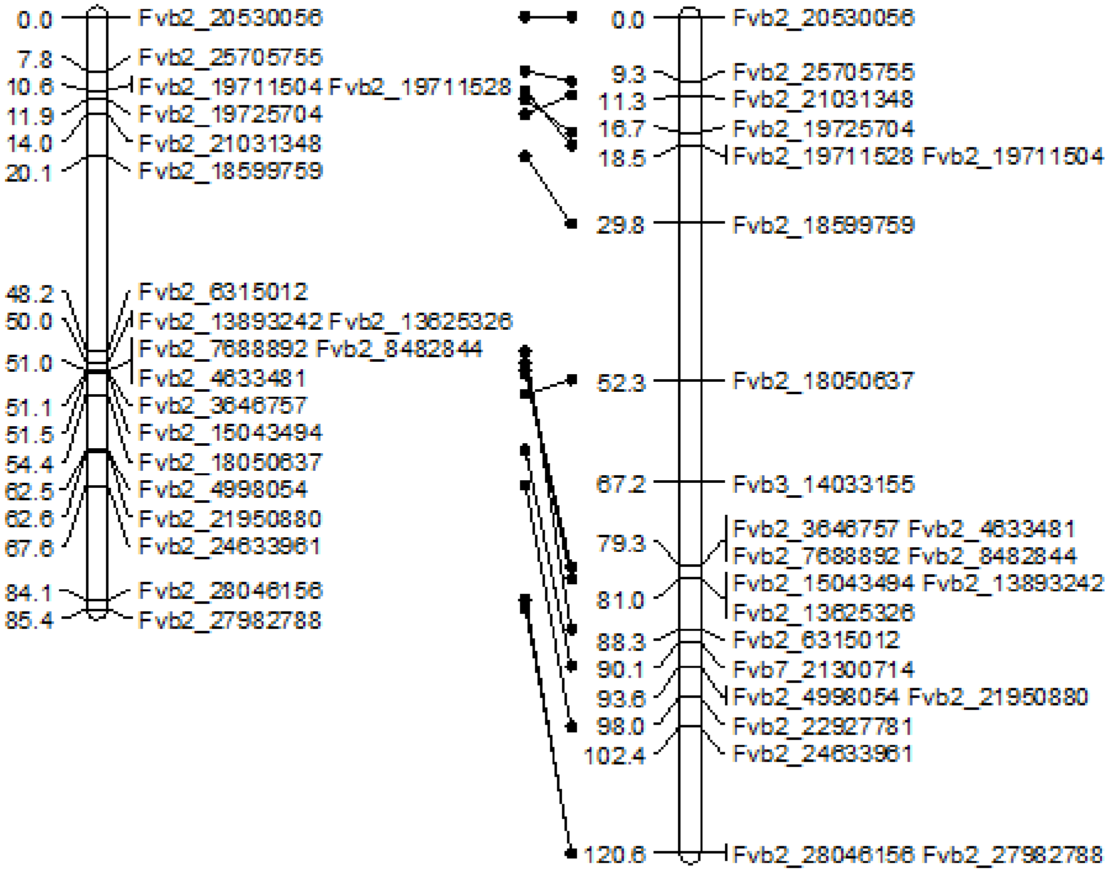

Fvb_2

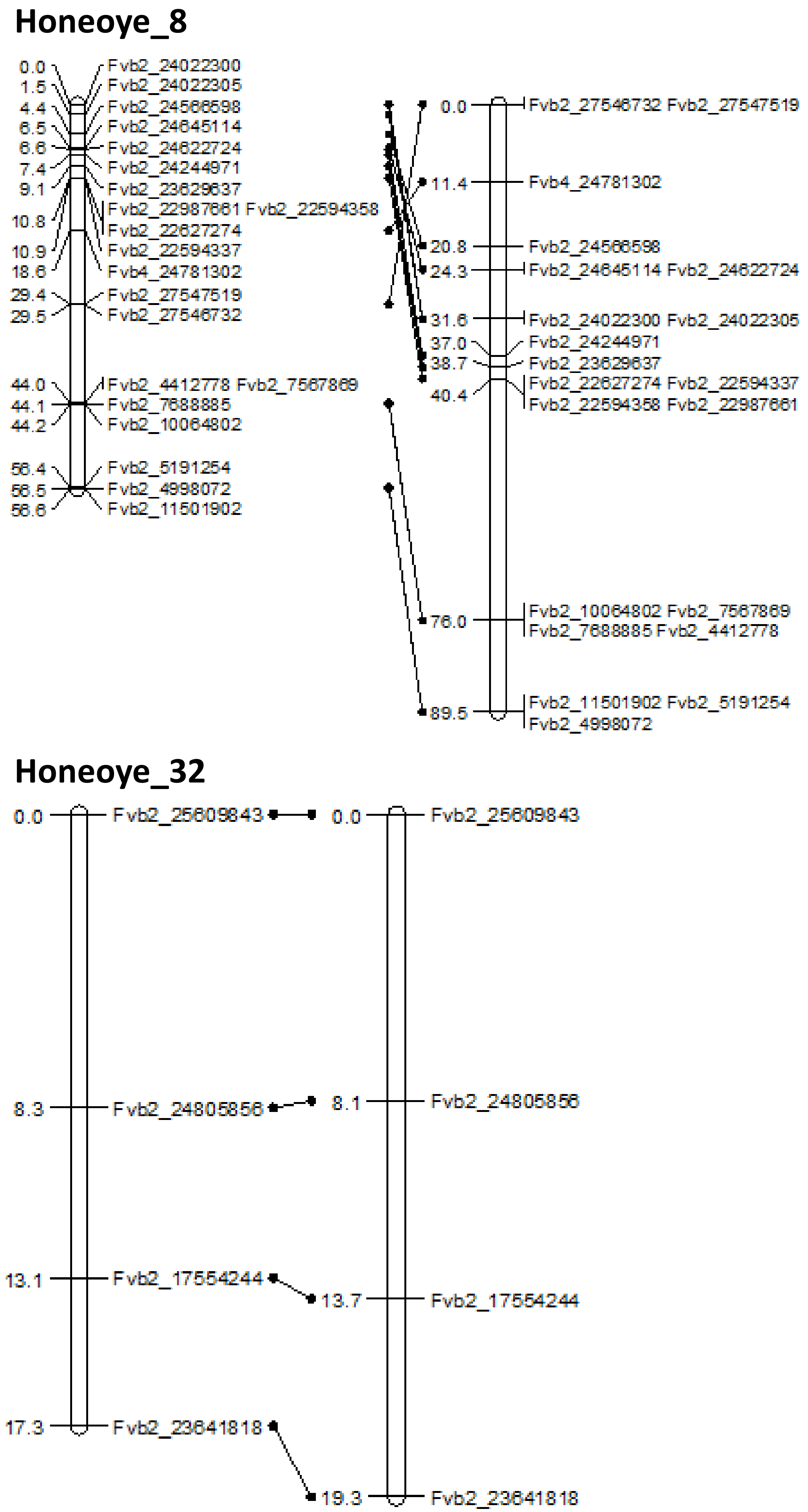
Honeoye_8
0.0 Fvb2_24022300
1.5 Fvb2_24022305
4.4 Fvb2_24566598
6.5 Fvb2_24645114
6.6 Fvb2_24622724
7.4 Fvb2_24244971
9.1 Fvb2_23629637
10.8 Fvb2_22987661 Fvb2_22594358
Fvb2_22627274
10.9 Fvb2_22594337
18.6 Fvb4_24781302
29.4 Fvb2_27547519
29.5 Fvb2_27546732
44.0 Fvb2_4412778 Fvb2_7567869
44.1 Fvb2_7688885
44.2 Fvb2_10064802
56.4 Fvb2_5191254
56.5 Fvb2_4998072
56.6 Fvb2_11501902
0.0 Fvb2_27546732 Fvb2_27547519
11.4 Fvb4_24781302
20.8 Fvb2_24566598
24.3 Fvb2_24645114 Fvb2_24622724
31.6 Fvb2_24022300 Fvb2_24022305
37.0 Fvb2_24244971
38.7 Fvb2_23629637
40.4 Fvb2_22627274 Fvb2_22594337
Fvb2_22594358 Fvb2_22987661
76.0 Fvb2_10064802 Fvb2_7567869
Fvb2_7688885 Fvb2_4412778
89.5 Fvb2_11501902 Fvb2_5191254
Fvb2_4998072
Honeoye_32
0.0 Fvb2_25609843
8.3 Fvb2_24805856
13.1 Fvb2_17554244
17.3 Fvb2_23641818
0.0 Fvb2_25609843
8.1 Fvb2_24805856
13.7 Fvb2_17554244
19.3 Fvb2_23641818

Fvb_3

Tribute_11

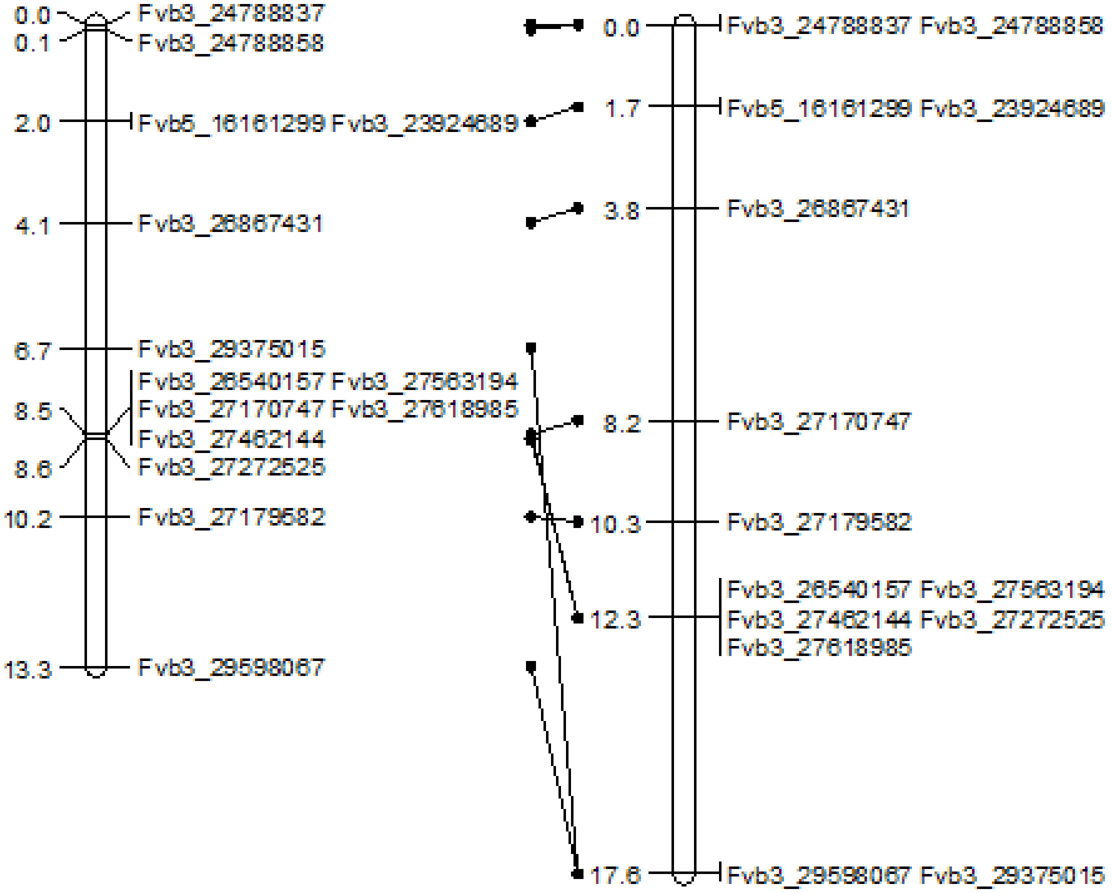

Tribute_14

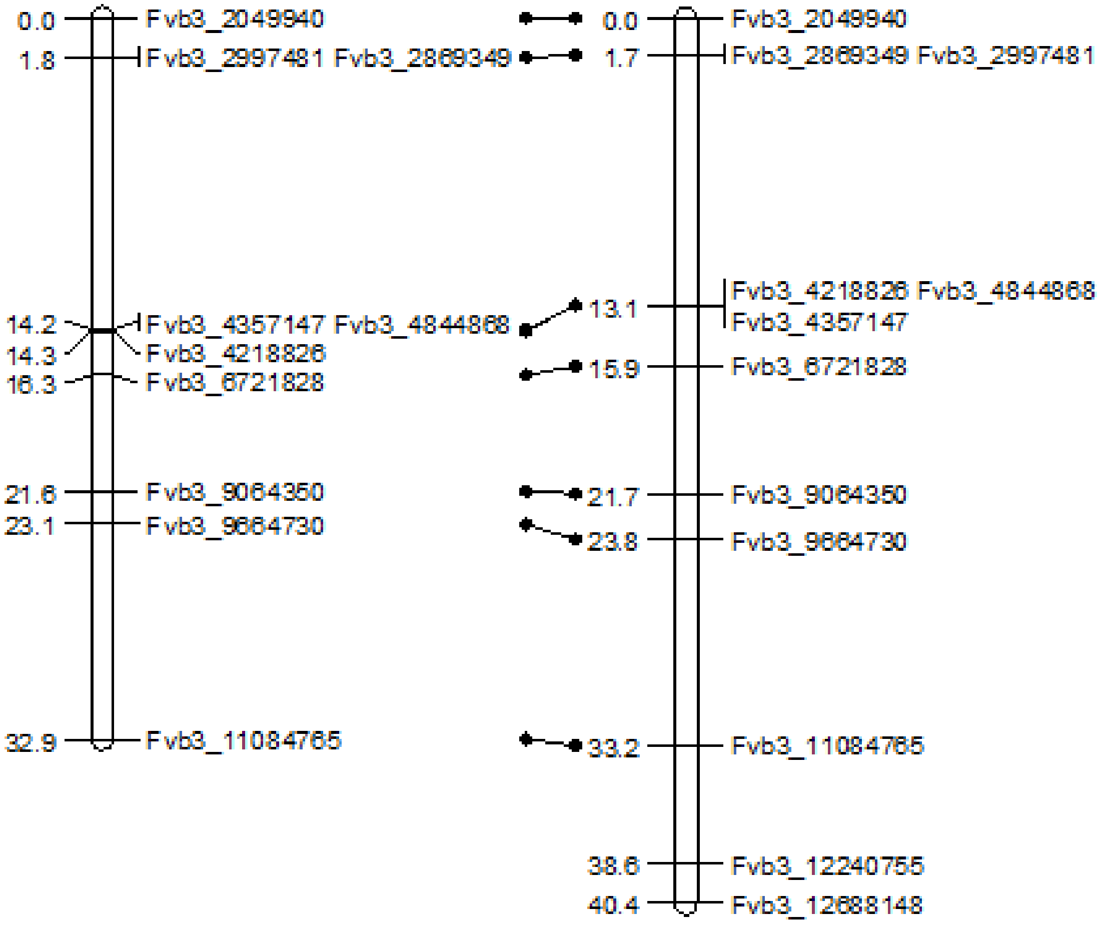

Fvb_3

Tribute_16

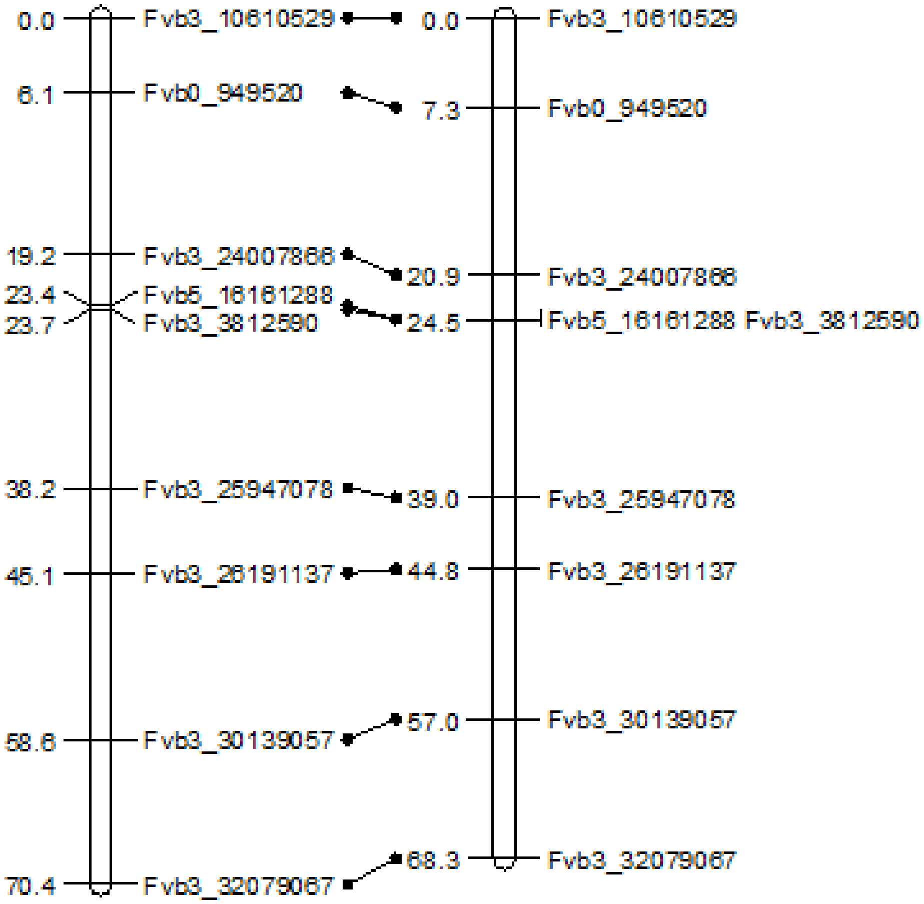

Tribute_21

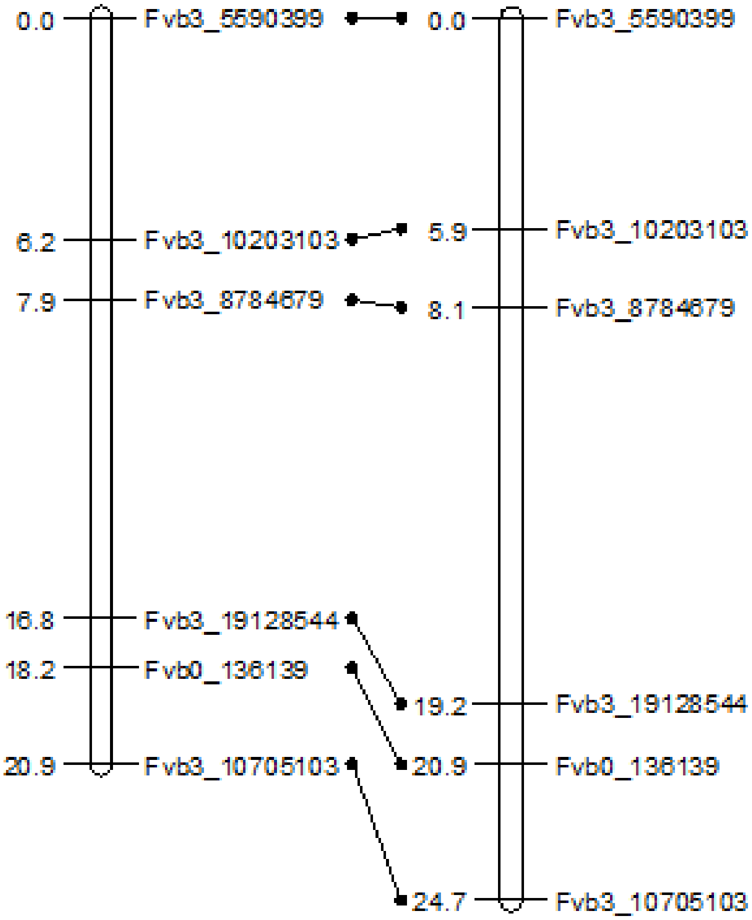

Fvb_3

Tribute_22

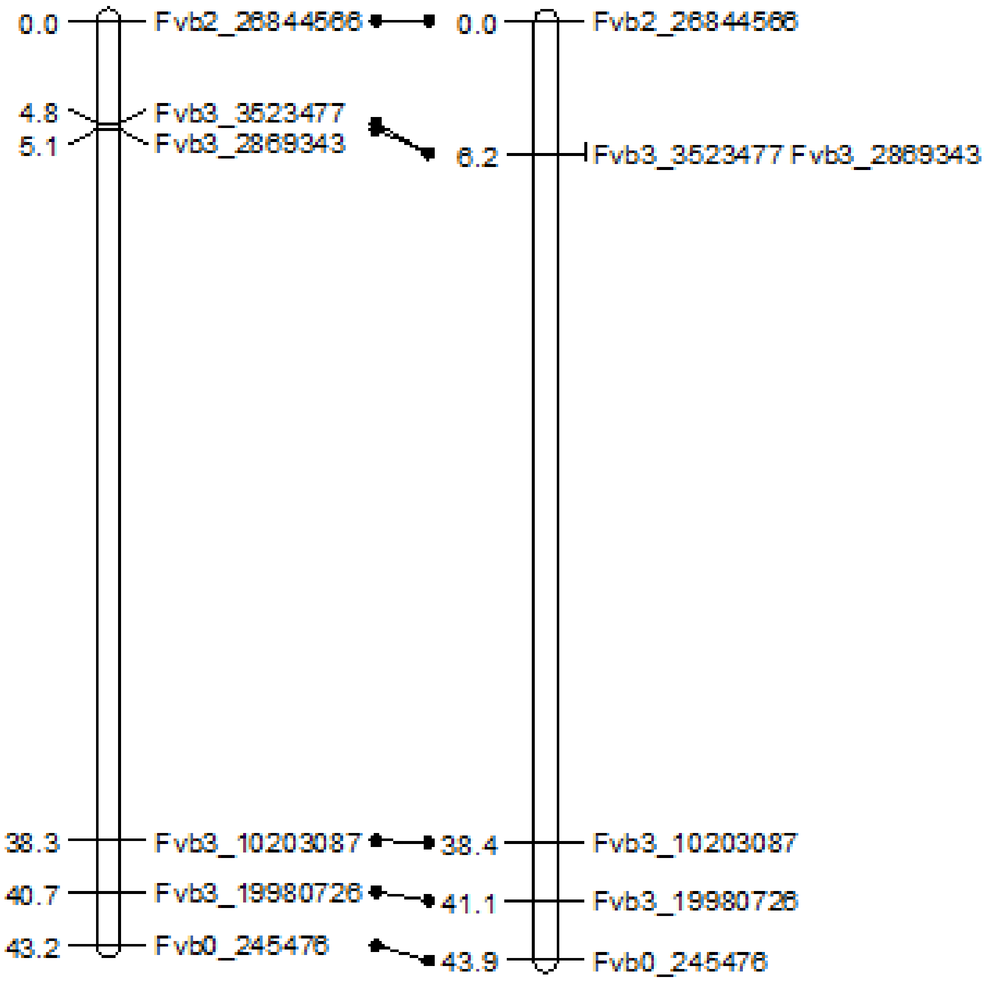

Fvb_3

Honeoye_2

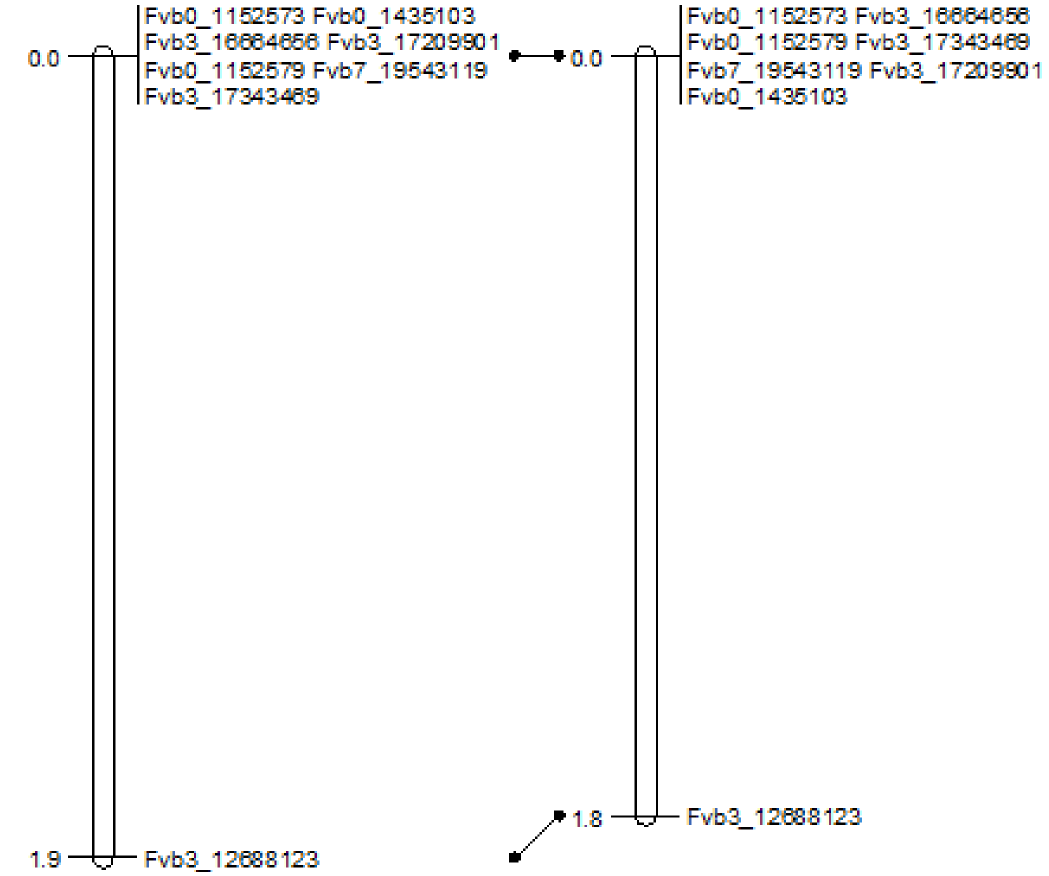

Honeoye_3

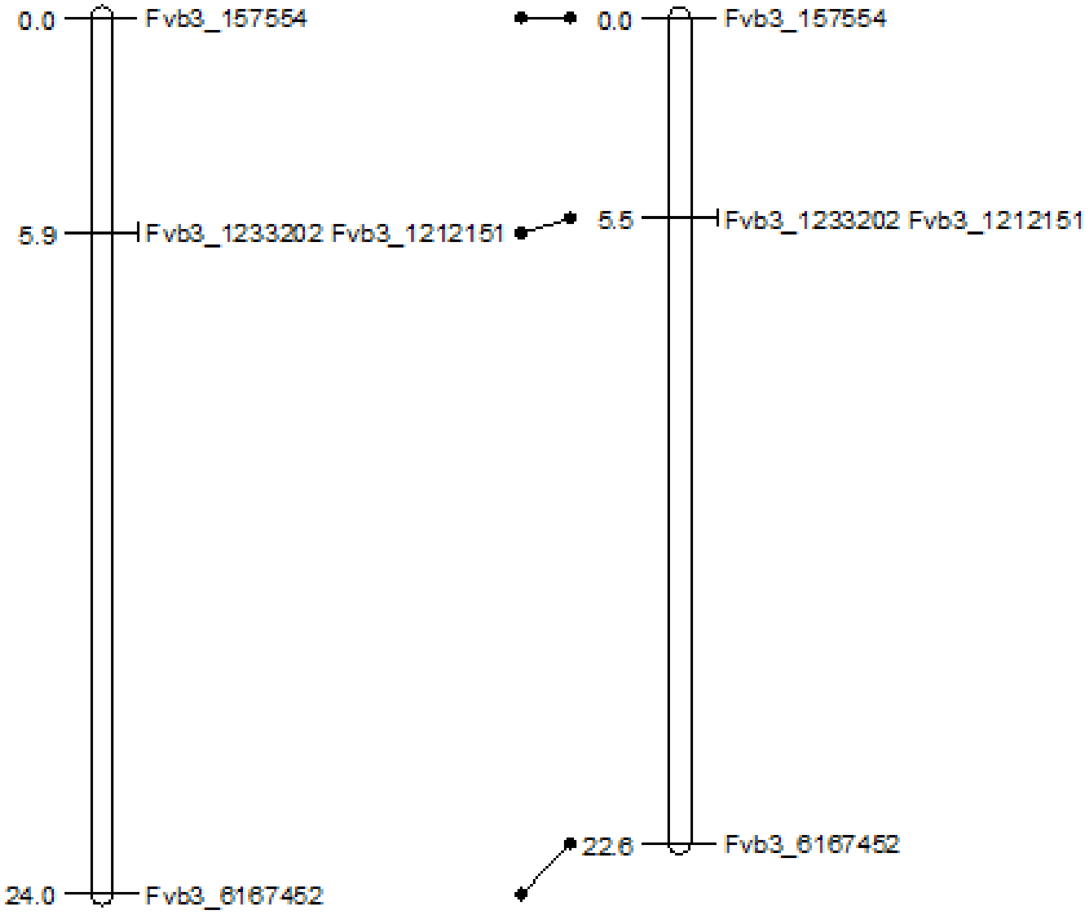

Fvb_3

Honeoye_6

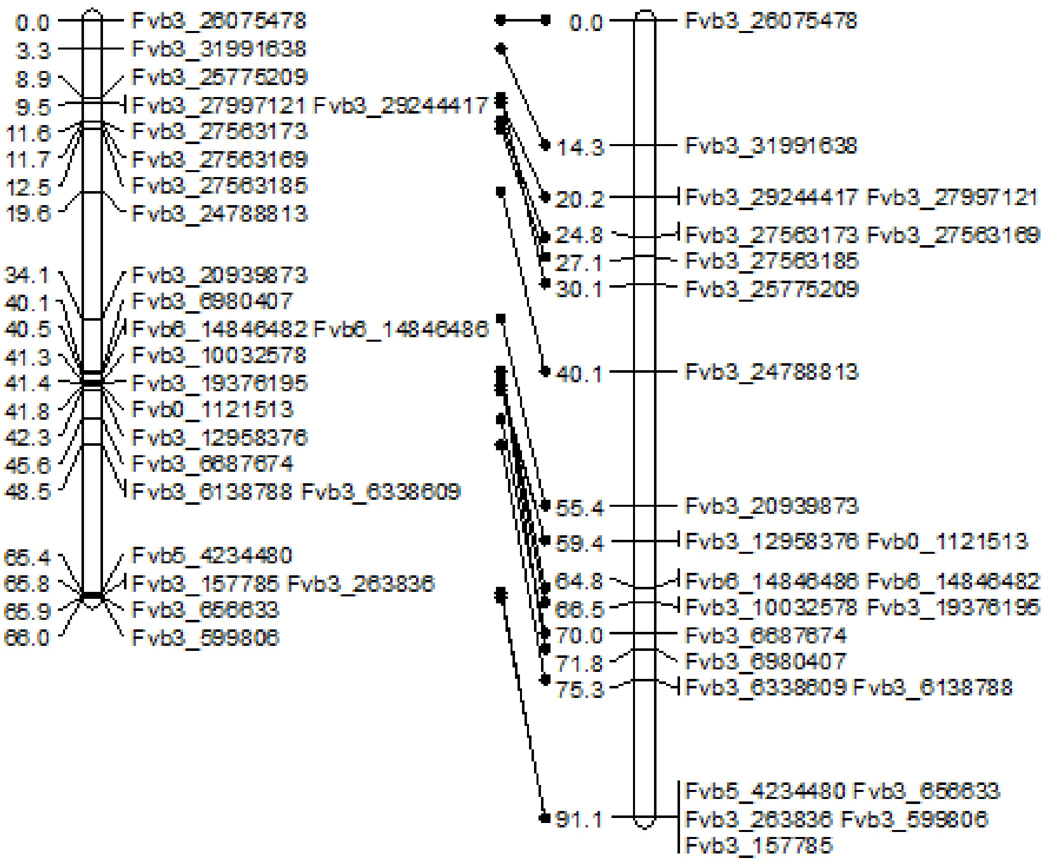

Honeoye_10

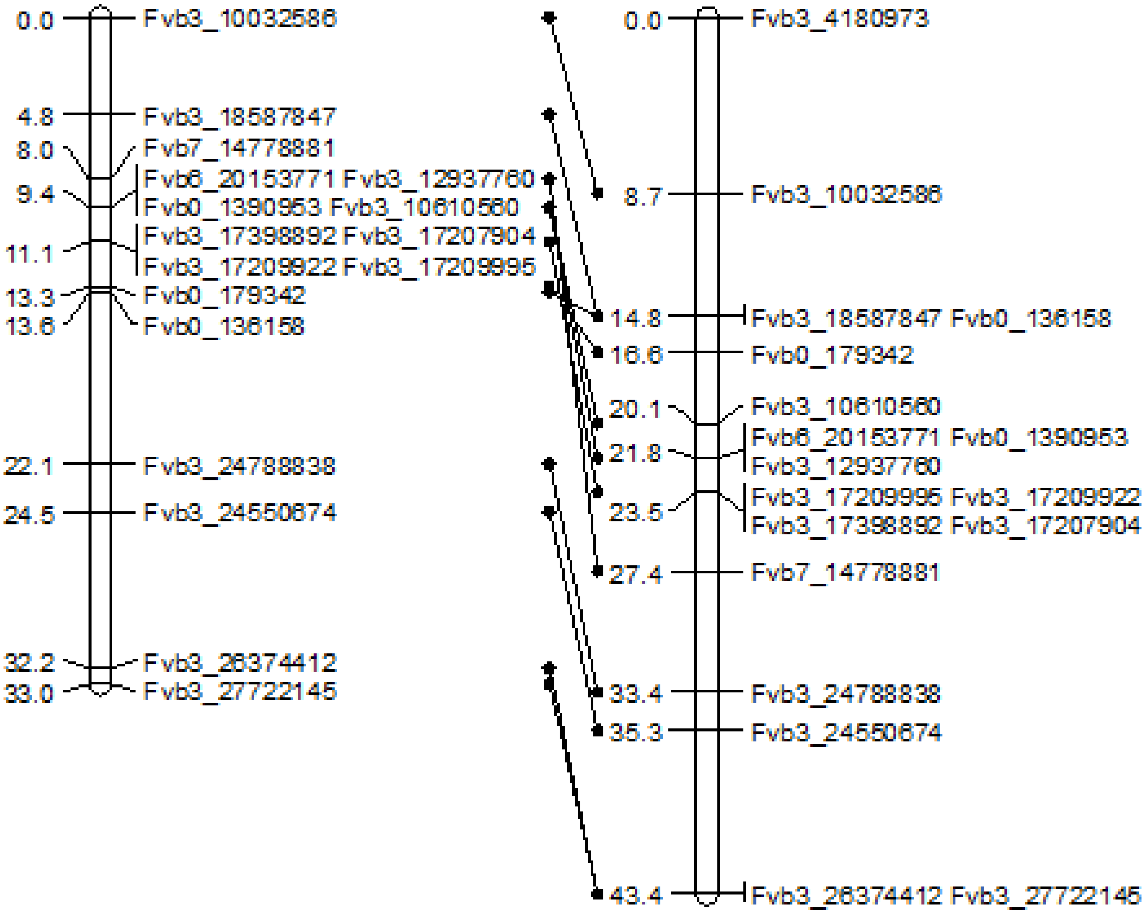

Fvb_4

Tribute_2

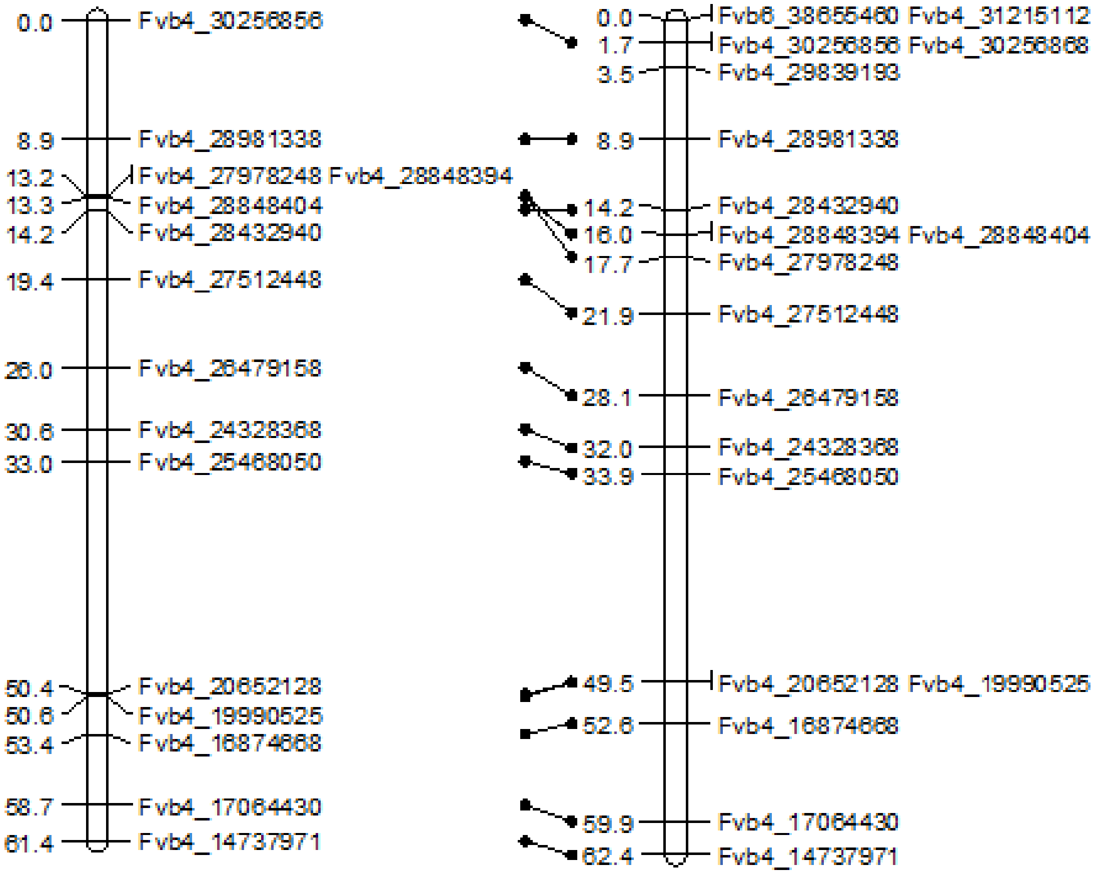

Tribute_7

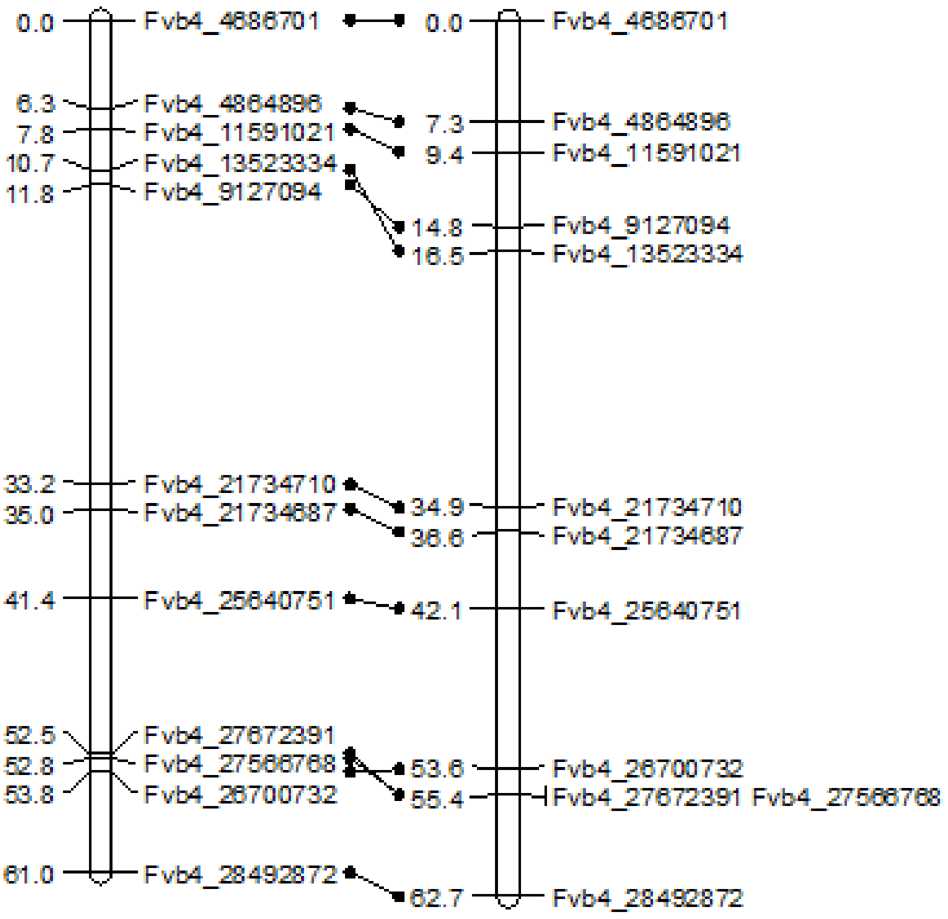

Fvb_4

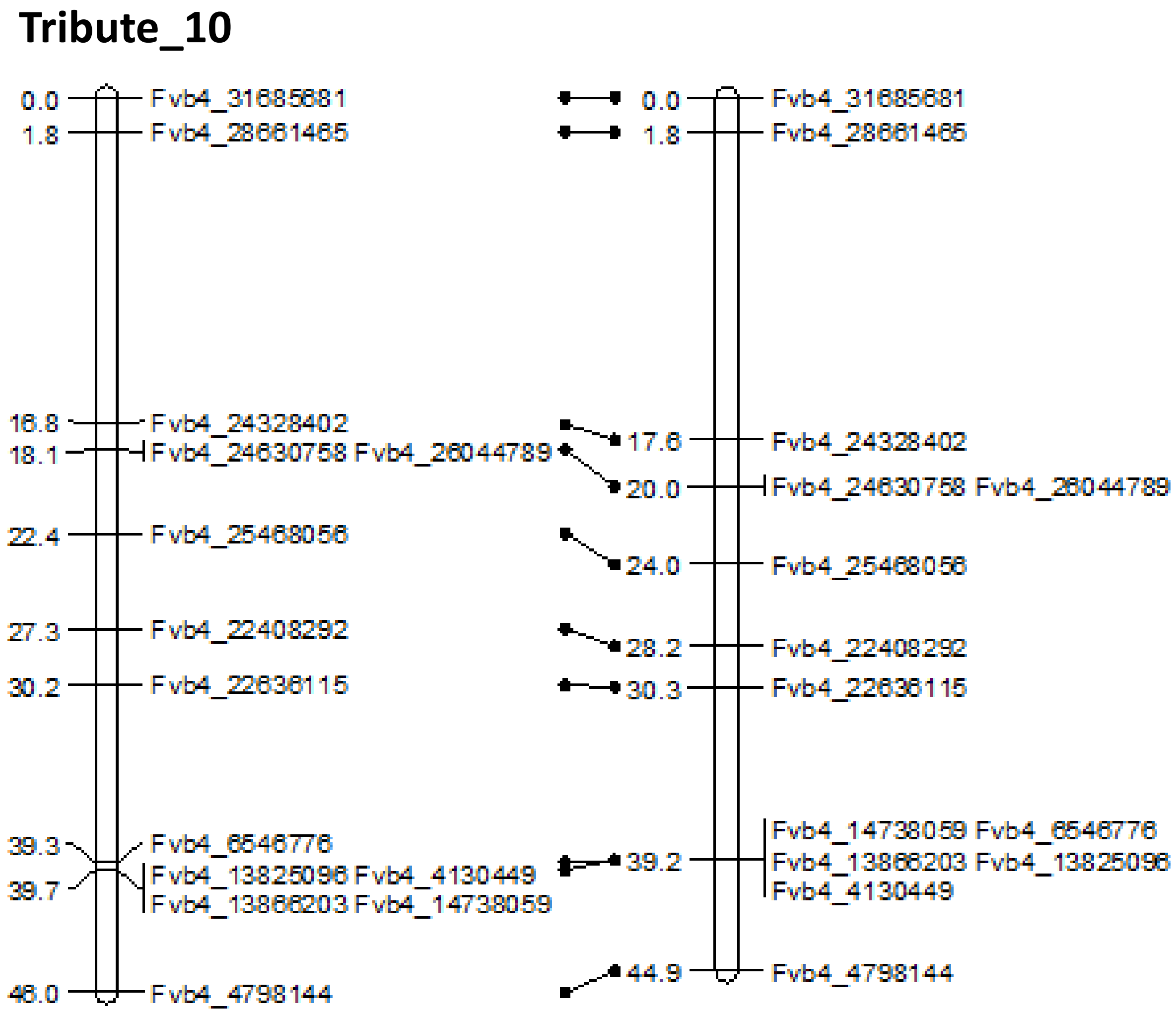
Tribute_10
0.0 Fvb4_31685681
1.8 Fvb4_28661465
16.8 Fvb4_24328402
18.1 Fvb4_24630758 Fvb4_26044789
22.4 Fvb4_25468056
27.3 Fvb4_22408292
30.2 Fvb4_22636115
39.3 Fvb4_6546776
39.7 Fvb4_13825096 Fvb4_4130449
Fvb4_13866203 Fvb4_14738059
46.0 Fvb4_4798144
0.0 Fvb4_31685681
1.8 Fvb4_28661465
17.6 Fvb4_24328402
20.0 Fvb4_24630758 Fvb4_26044789
24.0 Fvb4_25468056
28.2 Fvb4_22408292
30.3 Fvb4_22636115
39.2 Fvb4_14738059 Fvb4_6546776
Fvb4_13866203 Fvb4_13825096
Fvb4_4130449
44.9 Fvb4_4798144

Fvb_4

Honeoye_12

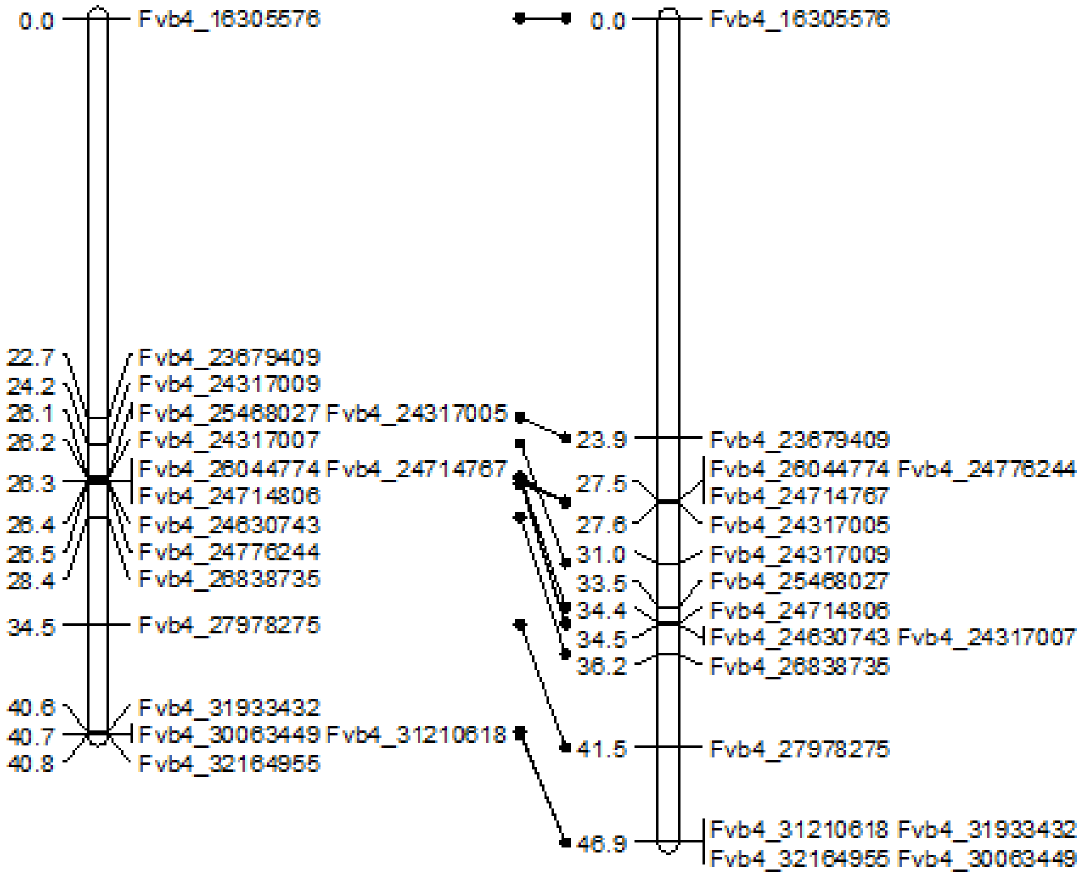

Honeoye_19

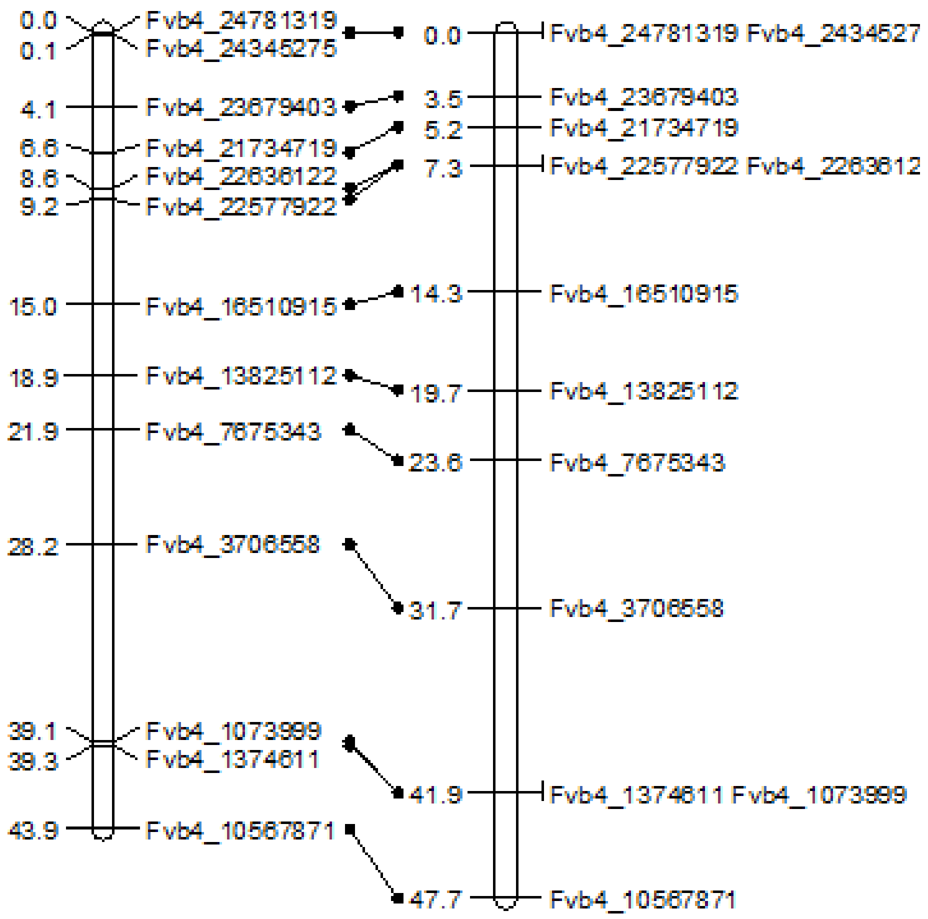

Fvb_4

Honeoye_28

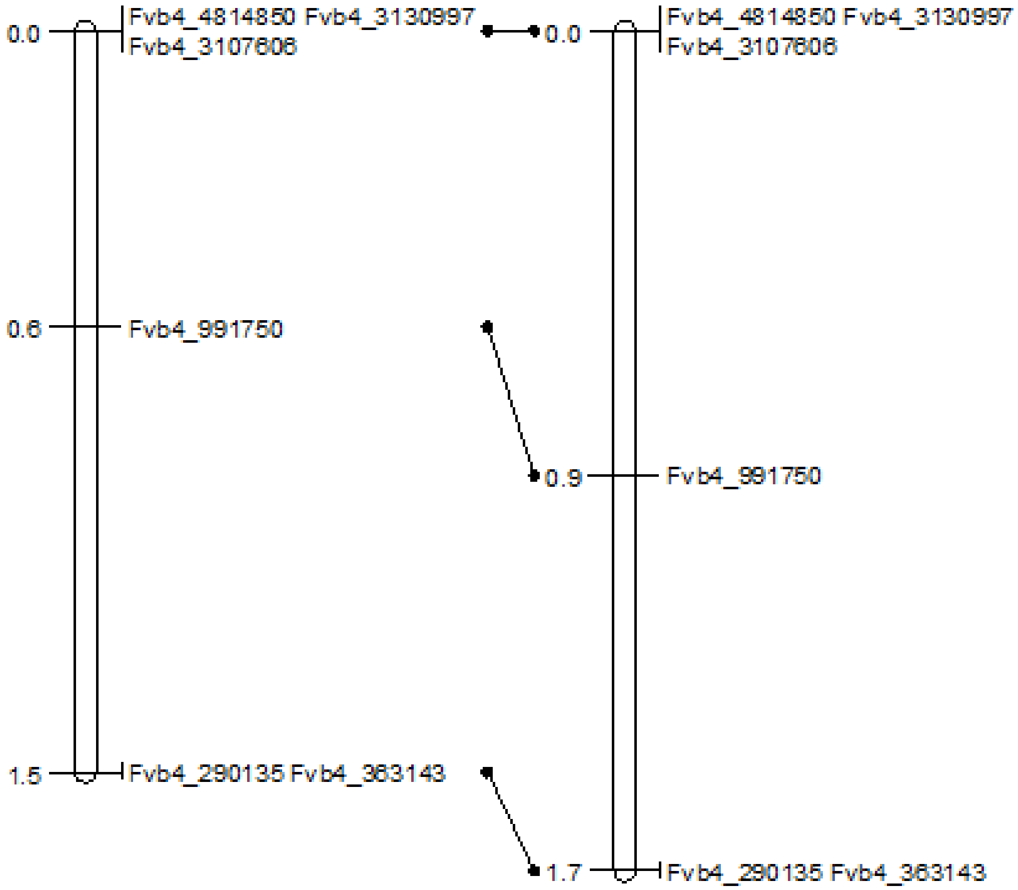

Honeoye_30

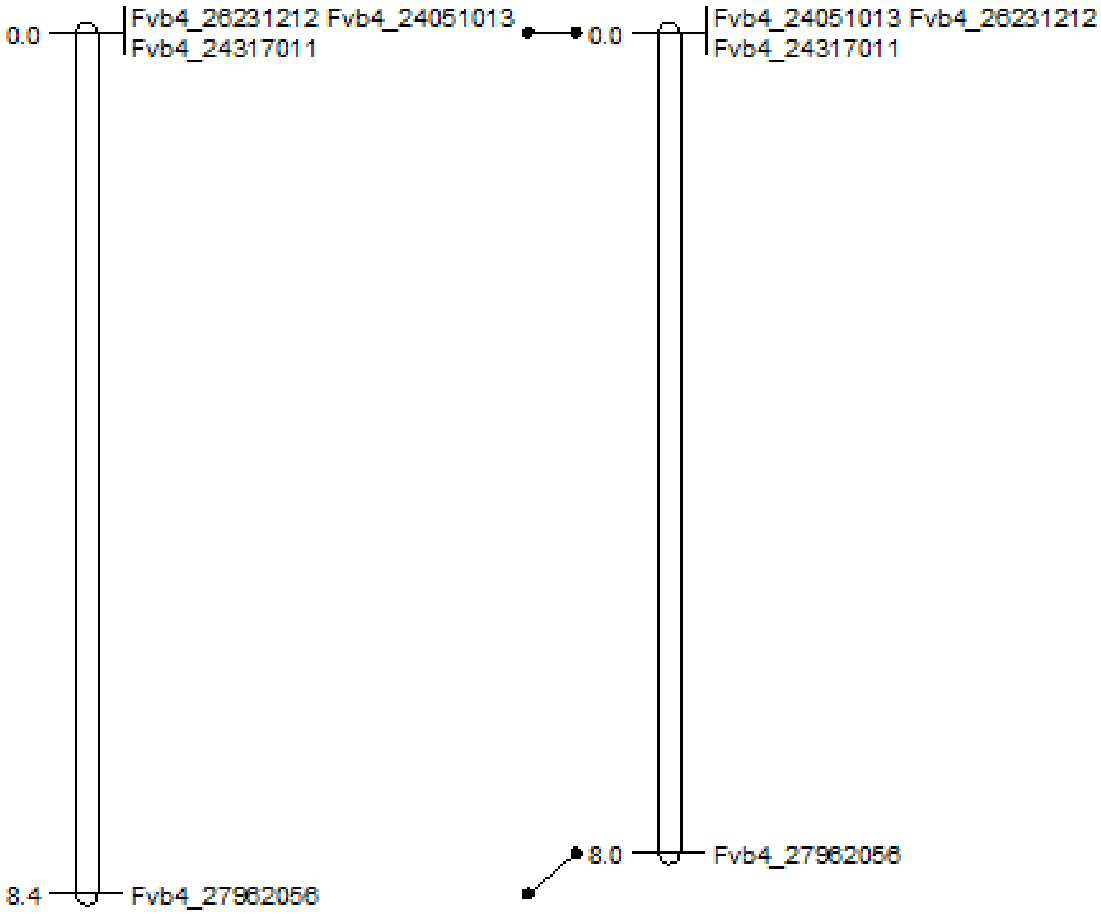

Fvb_5

Tribute_4

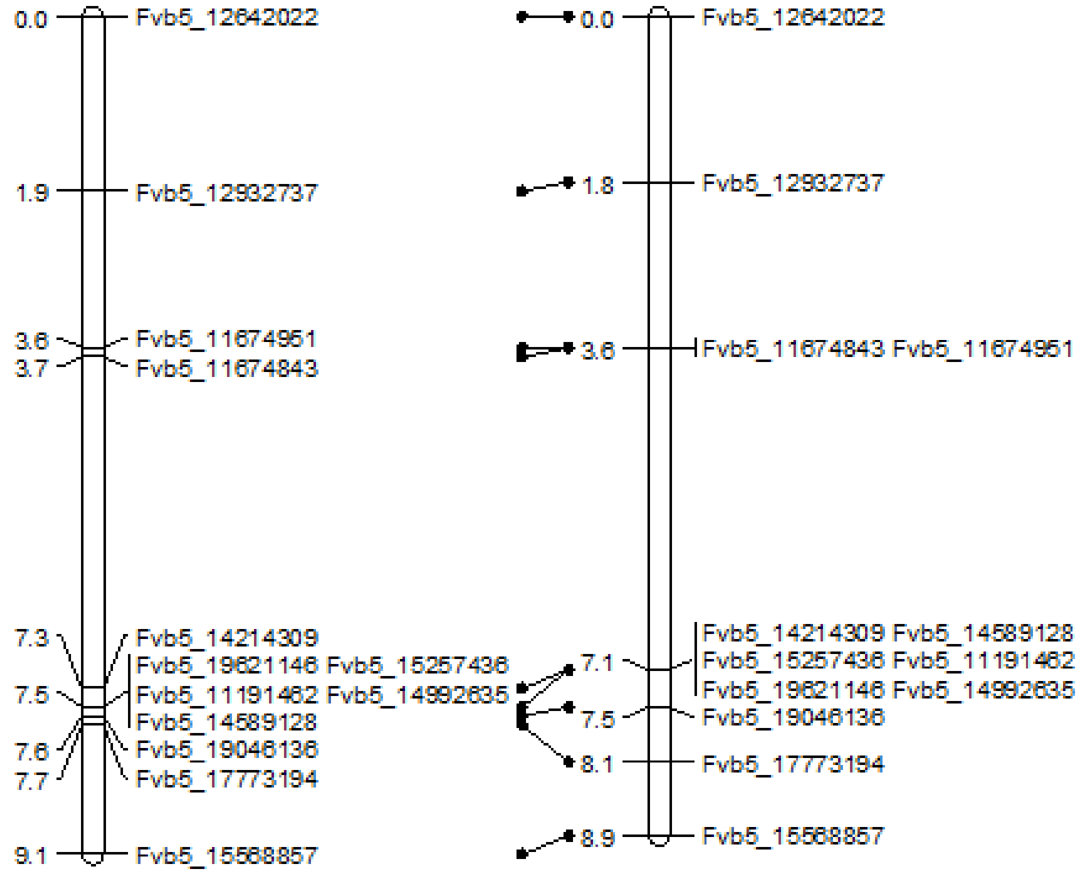

Tribute_5

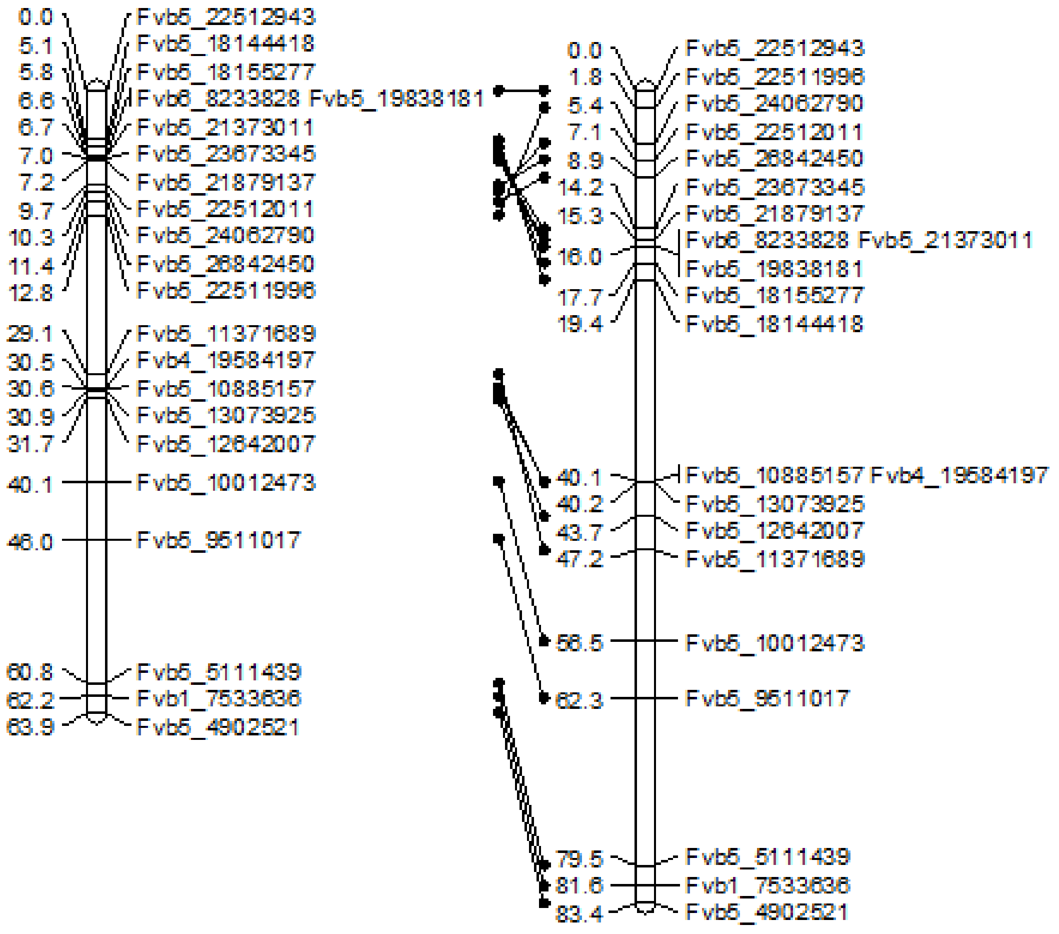

Fvb_5

Tribute_15

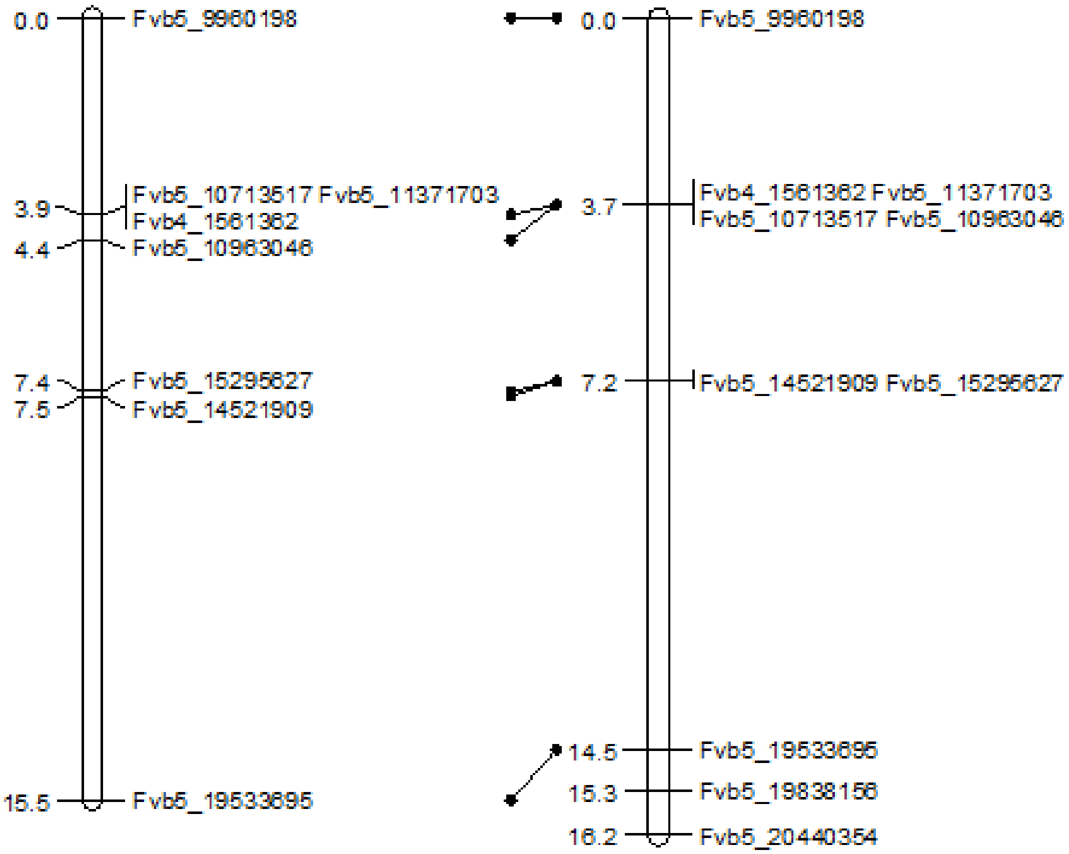

Fvb_5

Honeoye_14

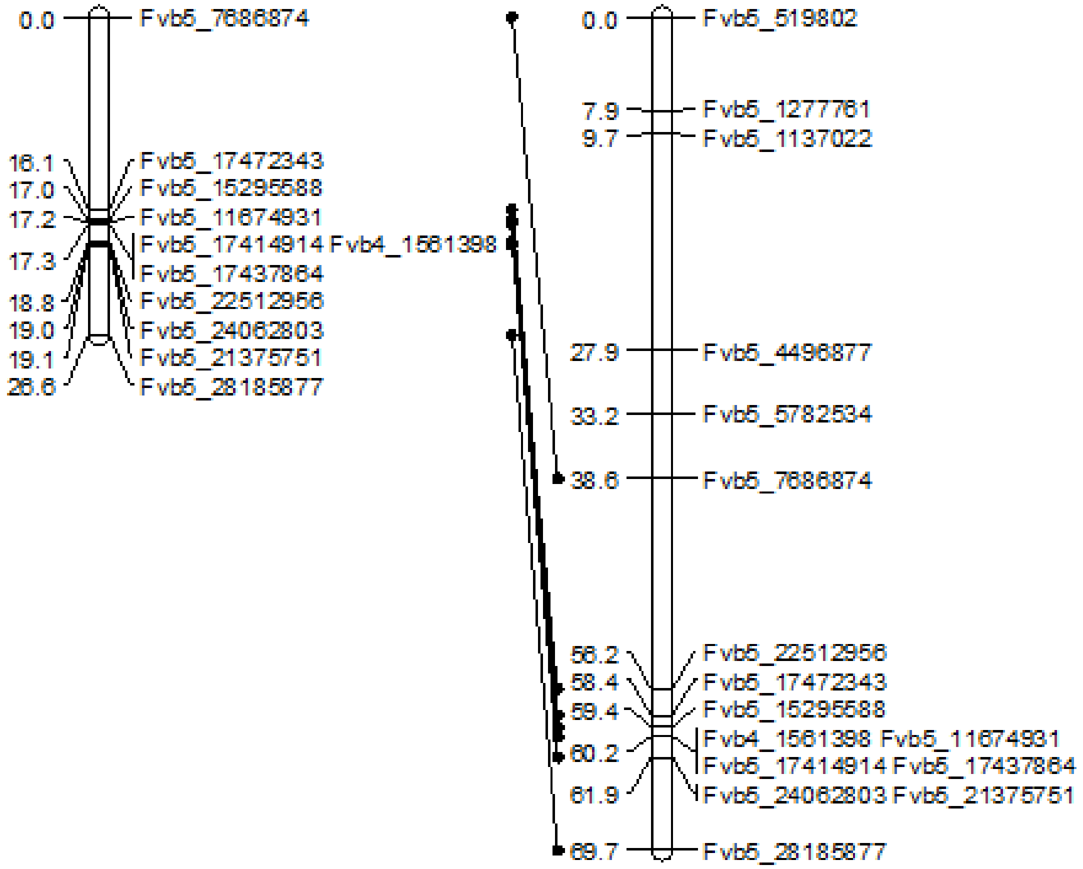

Honeoye_17

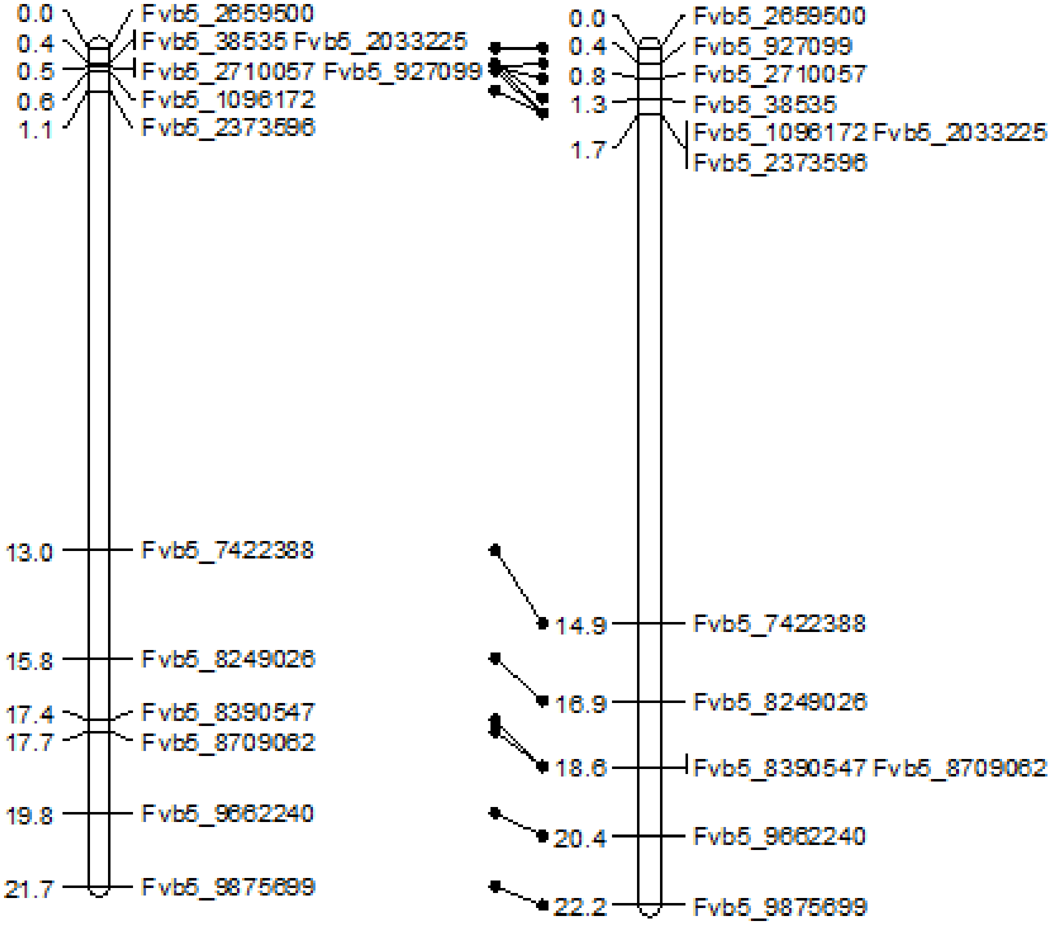

Fvb_5

Honeoye_22

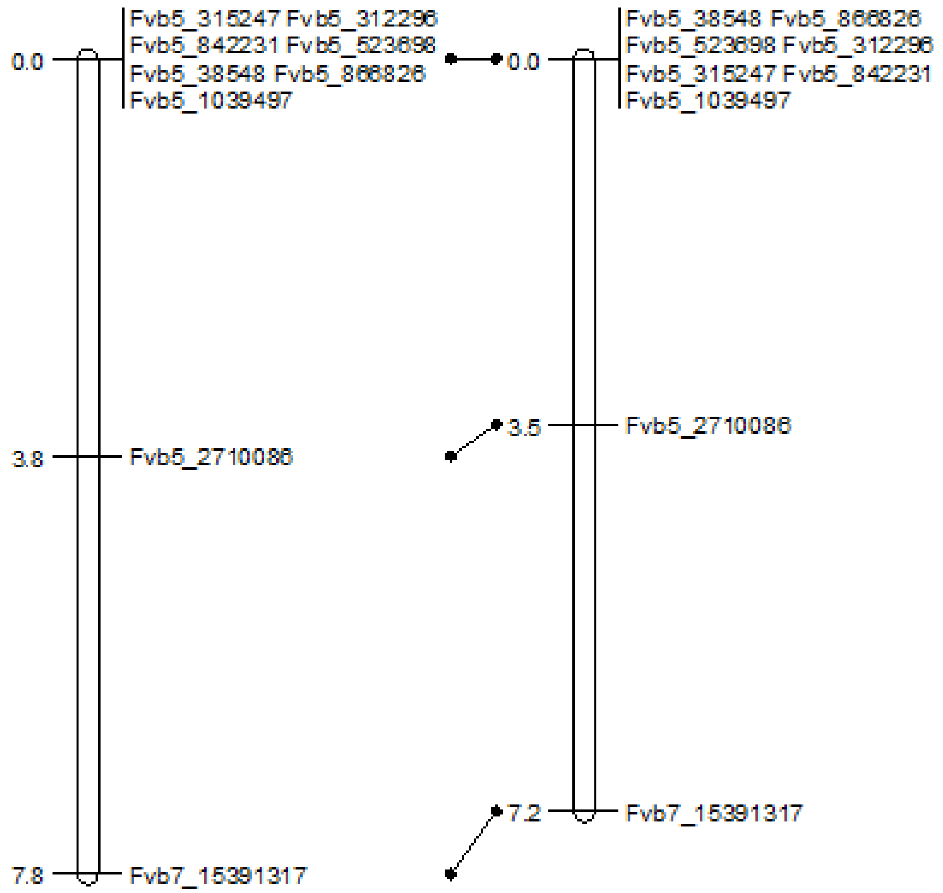

Honeoye_25

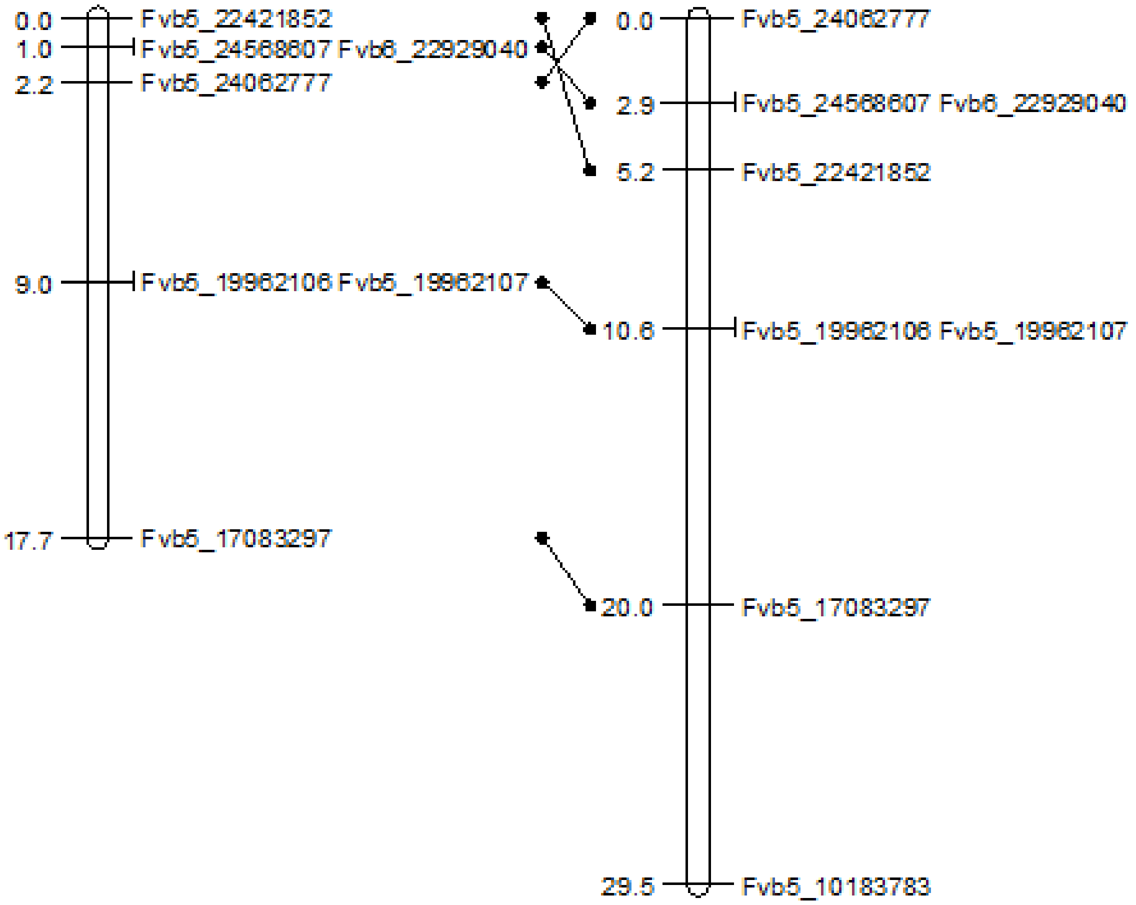

Fvb_6

Tribute_1

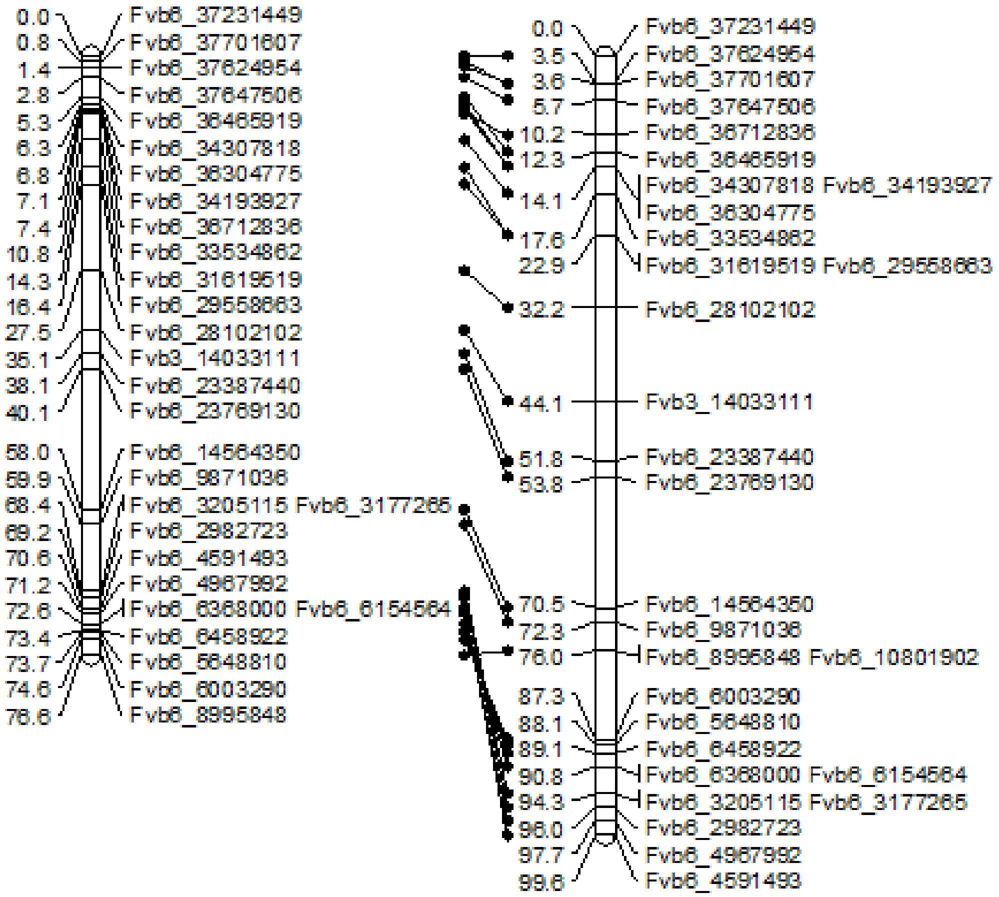

Tribute_3

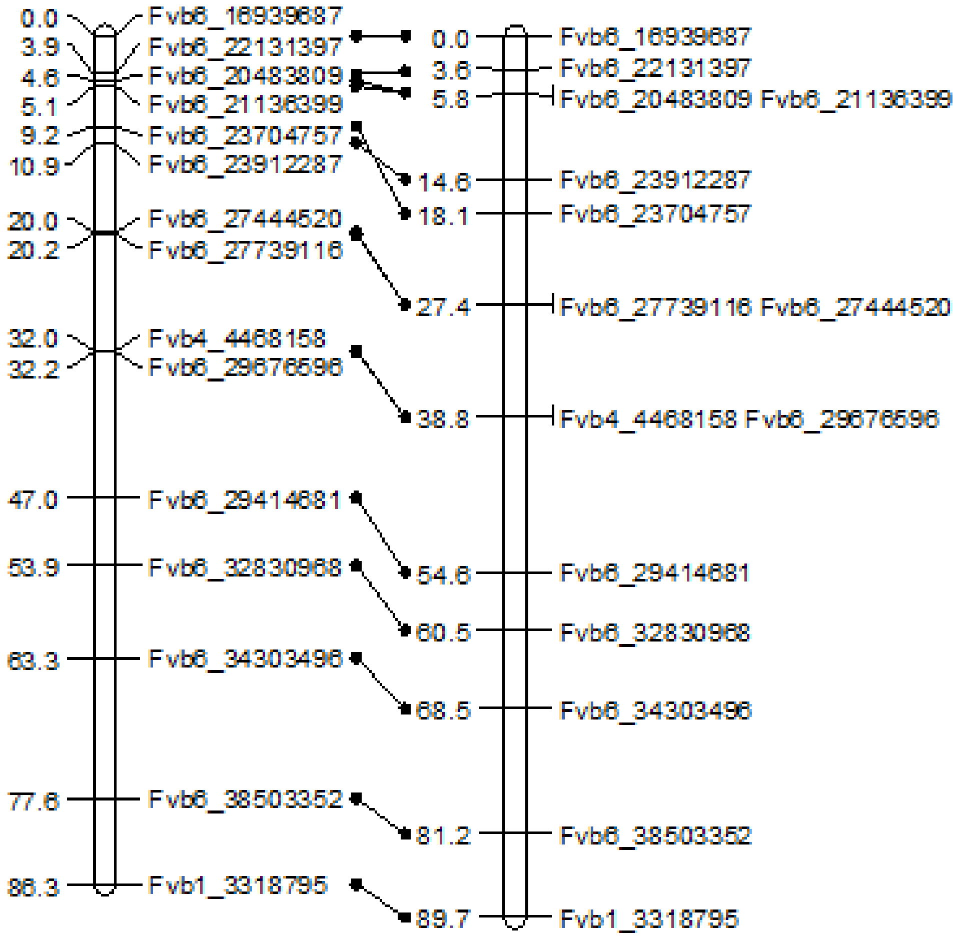

Fvb_6

Tribute_9

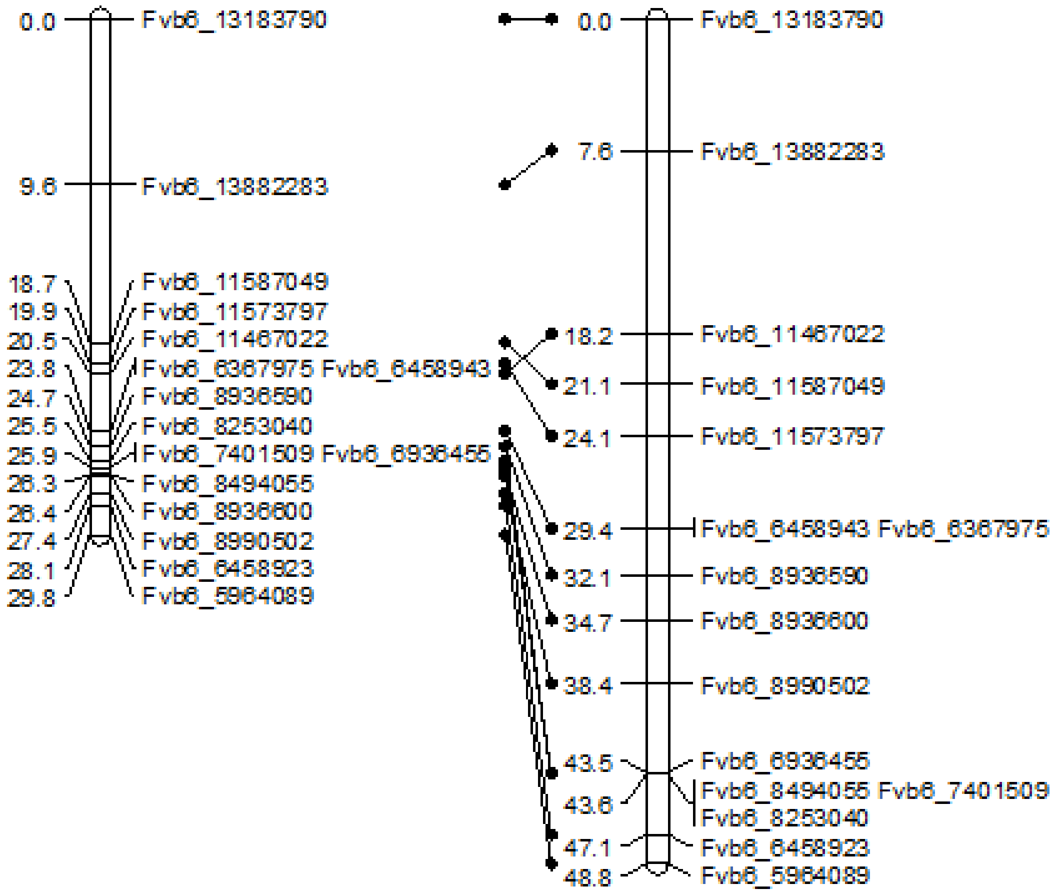

Tribute_27

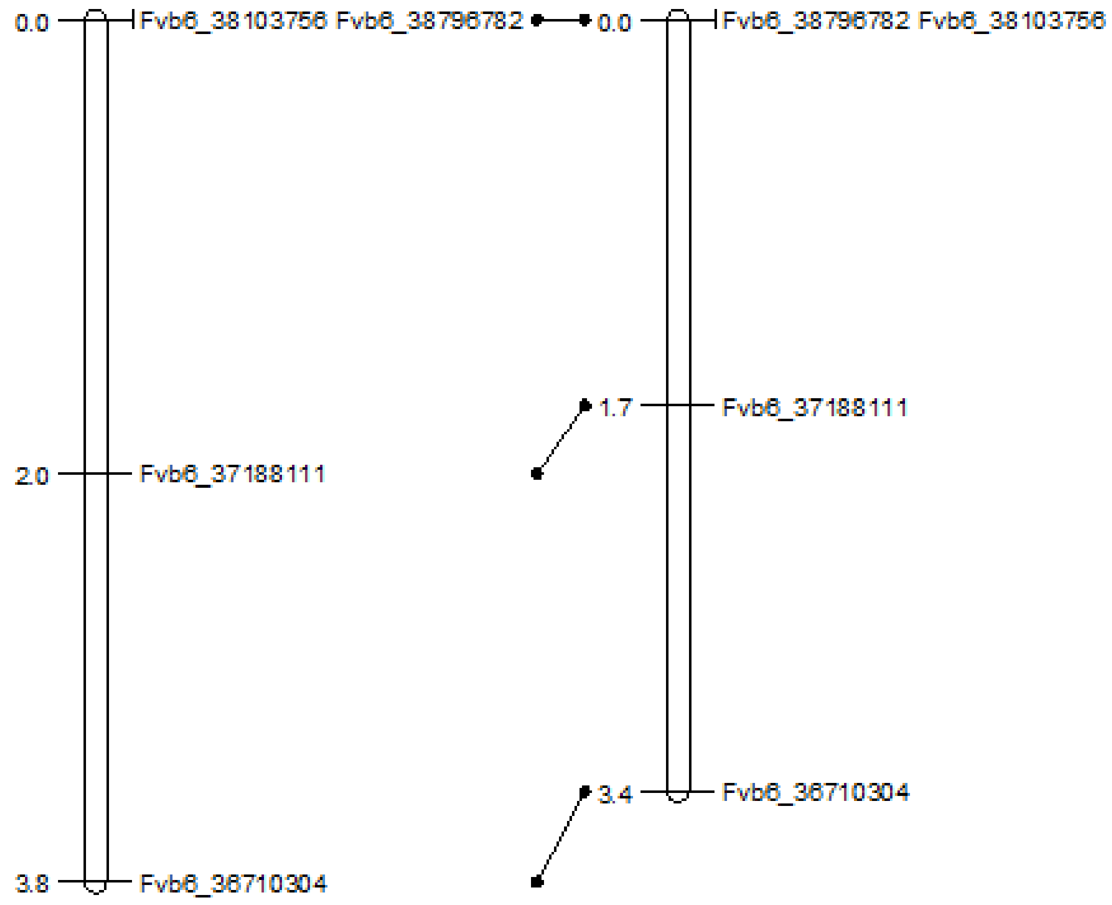

Fvb_6

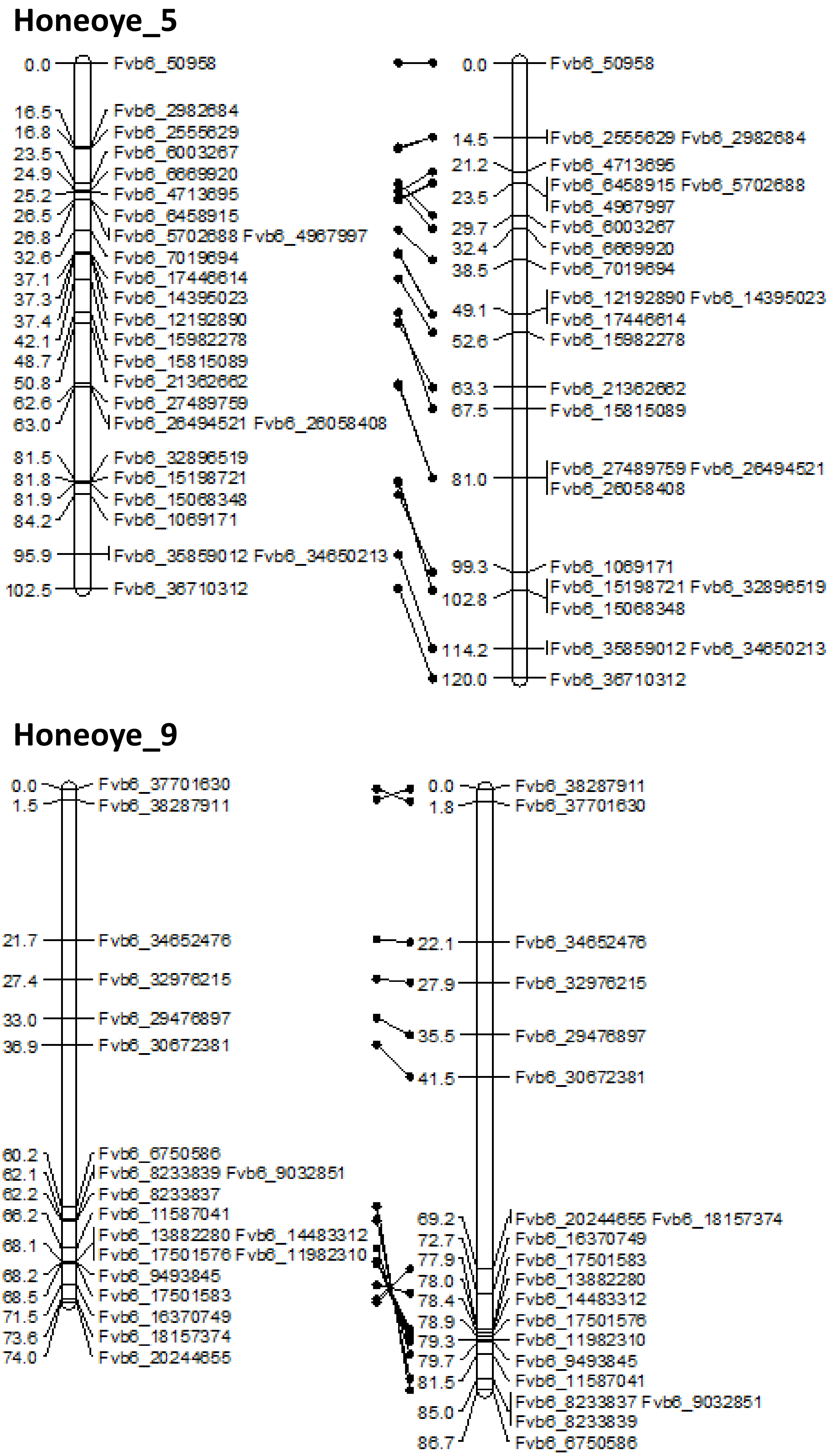

Fvb_6

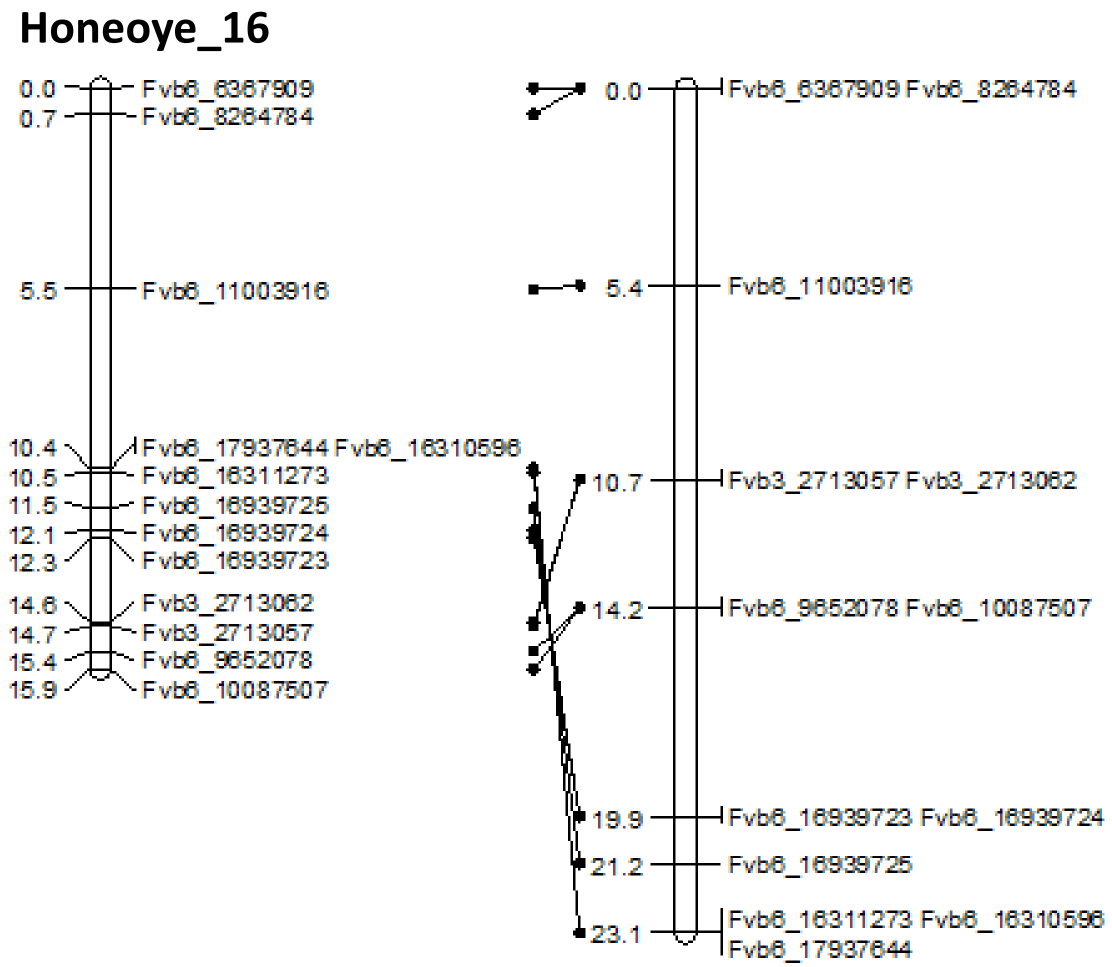
Honeoye_16
0.0 Fvb6_6367909
0.7 Fvb6_8264784
5.5 Fvb6_11003916
10.4 Fvb6_17937644 Fvb6_16310596
10.5 Fvb6_16311273
11.5 Fvb6_16939725
12.1 Fvb6_16939724
12.3 Fvb6_16939723
14.6 Fvb3_2713062
14.7 Fvb3_2713057
15.4 Fvb6_9652078
15.9 Fvb6_10087507
0.0 Fvb6_6367909 Fvb6_8264784
5.4 Fvb6_11003916
10.7 Fvb3_2713057 Fvb3_2713062
14.2 Fvb6_9652078 Fvb6_10087507
19.9 Fvb6_16939723 Fvb6_16939724
21.2 Fvb6_16939725
23.1 Fvb6_16311273 Fvb6_16310596
Fvb6_17937644

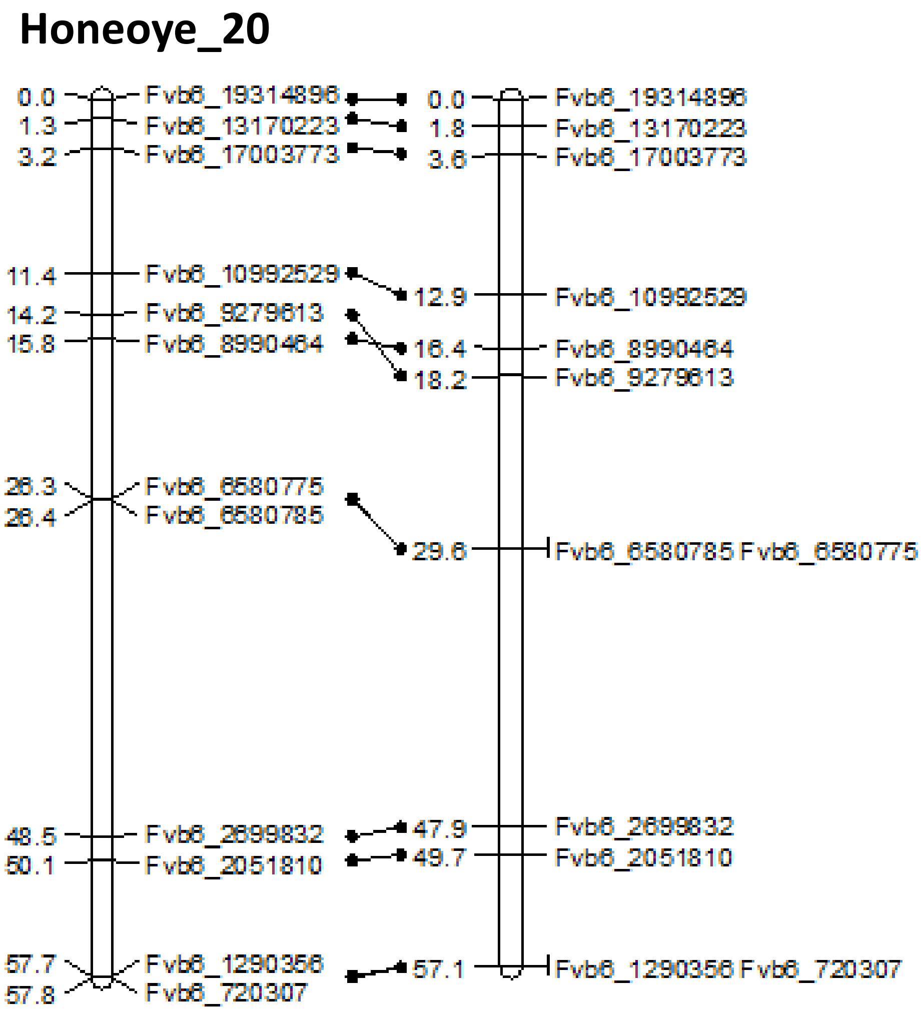
Honeoye_20
0.0 Fvb6_19314896
1.3 Fvb6_13170223
3.2 Fvb6_17003773
11.4 Fvb6_10992529
14.2 Fvb6_9279613
15.8 Fvb6_8990464
26.3 Fvb6_6580775
26.4 Fvb6_6580785
48.5 Fvb6_2699832
50.1 Fvb6_2051810
57.7 Fvb6_1290356
57.8 Fvb6_720307
0.0 Fvb6_19314896
1.8 Fvb6_13170223
3.6 Fvb6_17003773
12.9 Fvb6_10992529
16.4 Fvb6_8990464
18.2 Fvb6_9279613
29.6 Fvb6_6580785 Fvb6_6580775
47.9 Fvb6_2699832
49.7 Fvb6_2051810
57.1 Fvb6_1290356 Fvb6_720307

Fvb_7

Tribute_12

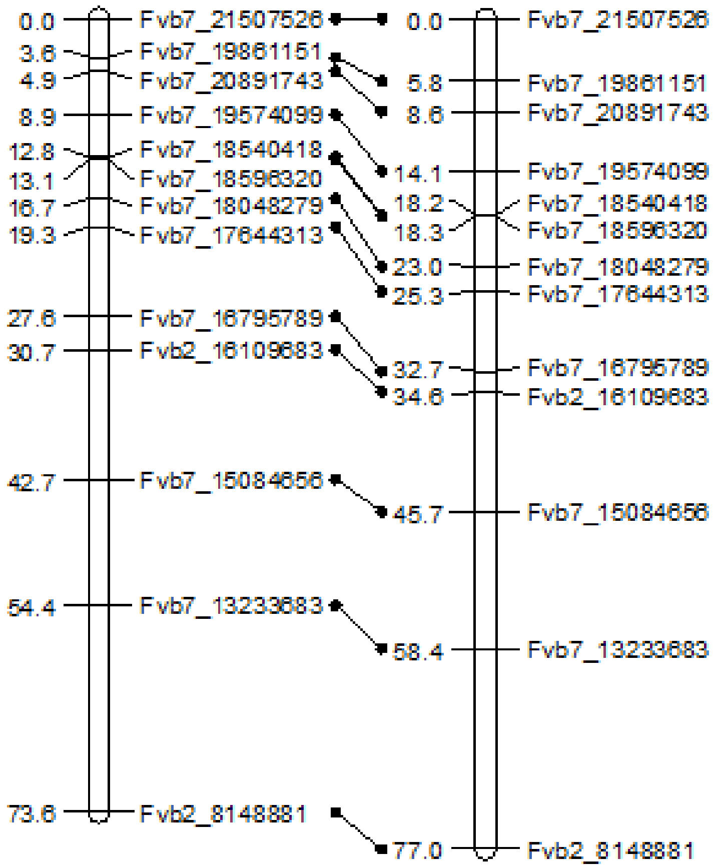

Tribute_18

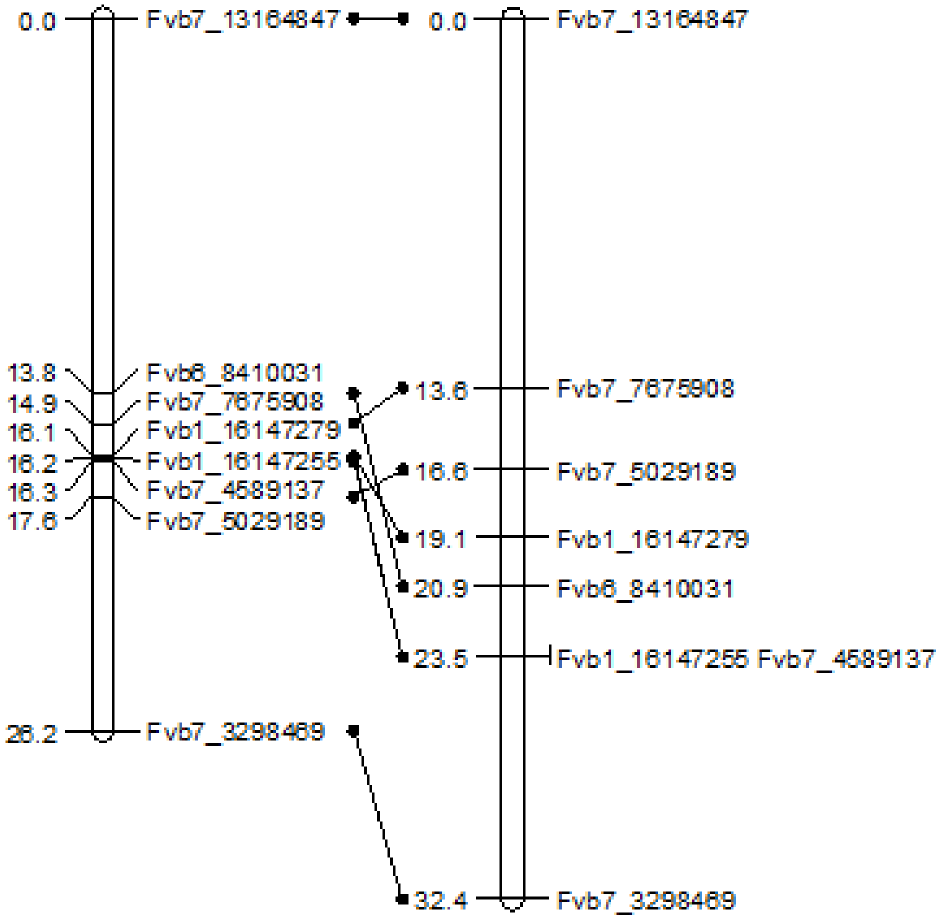

Fvb_7

Tribute_20

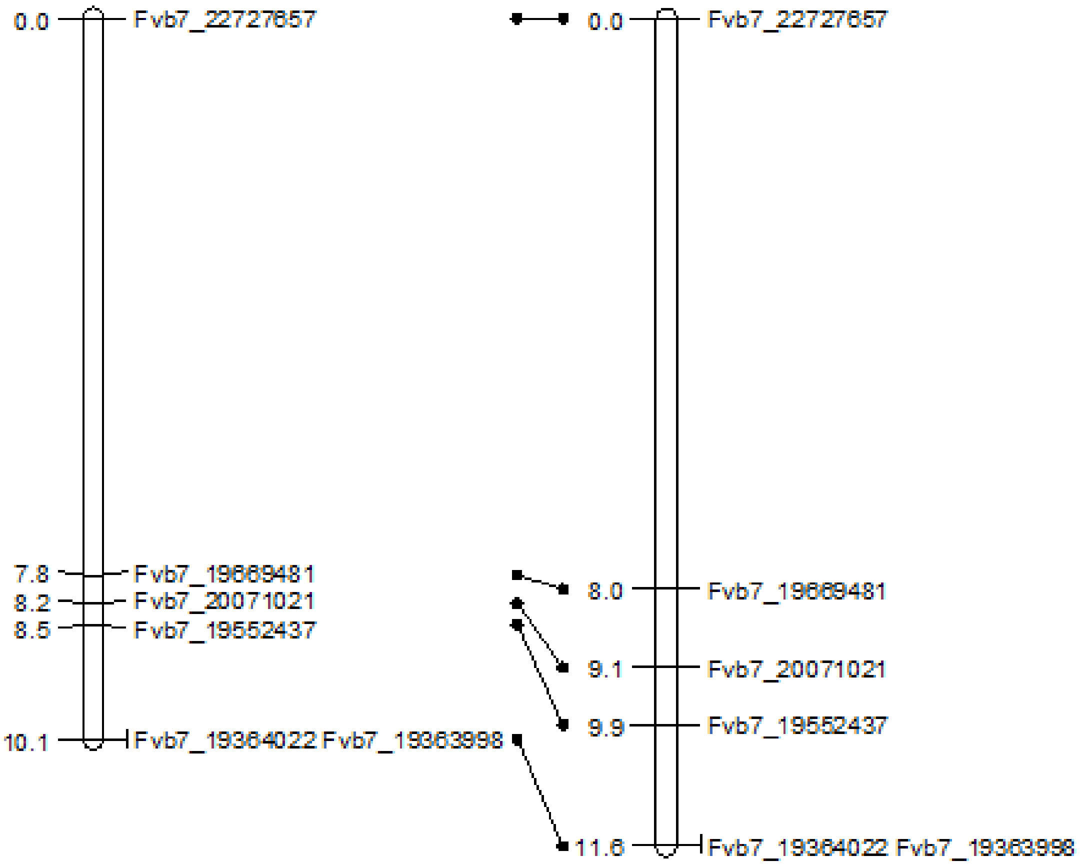

Fvb_7

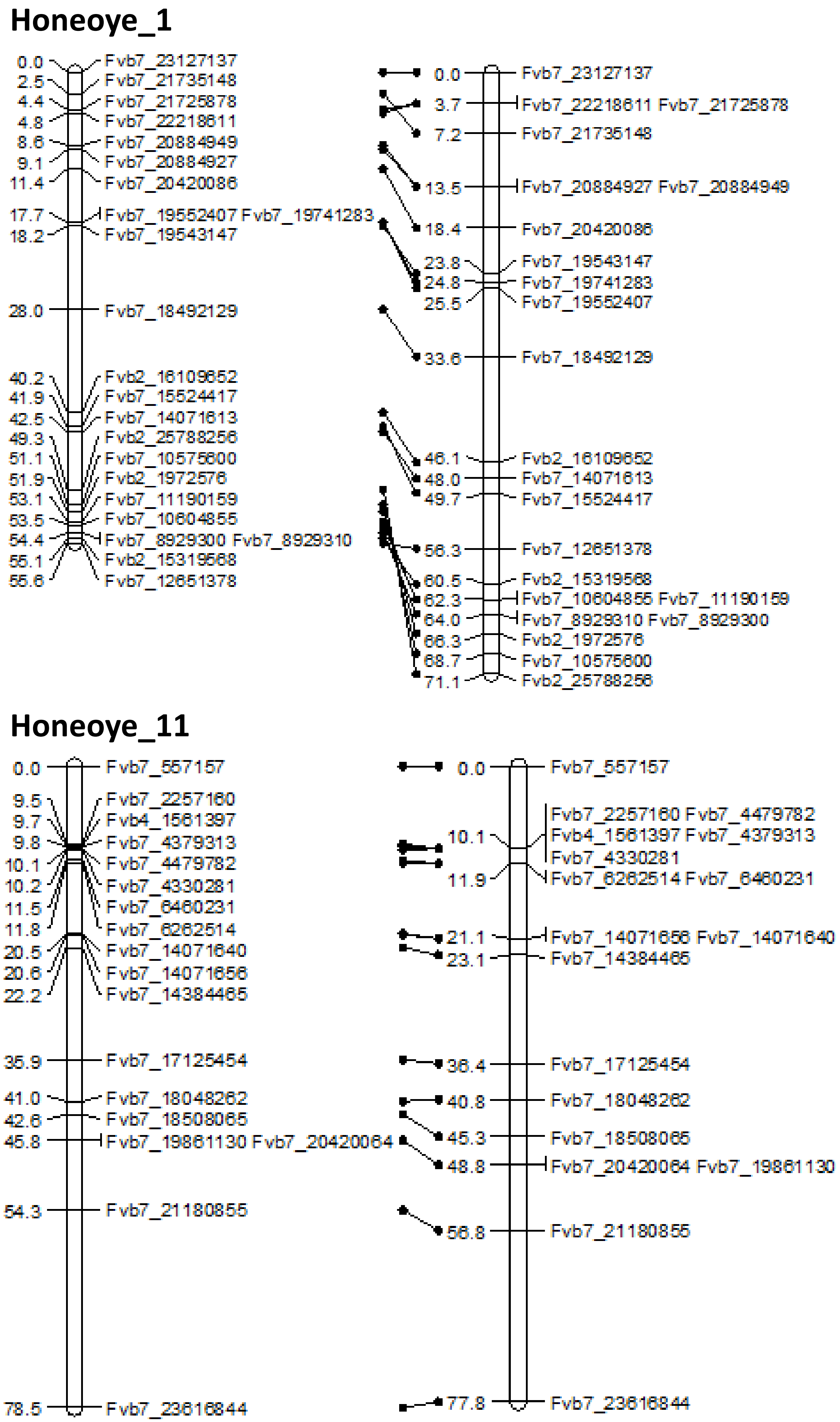
Honeoye_1
0.0 Fvb7_23127137
2.5 Fvb7_21735148
4.4 Fvb7_21725878
4.8 Fvb7_22218611
8.6 Fvb7_20884949
9.1 Fvb7_20884927
11.4 Fvb7_20420086
17.7 Fvb7_19552407 Fvb7_19741283
18.2 Fvb7_19543147
28.0 Fvb7_18492129
40.2 Fvb2_16109652
41.9 Fvb7_15524417
42.5 Fvb7_14071613
49.3 Fvb2_25788256
51.1 Fvb7_10575600
51.9 Fvb2_1972576
53.1 Fvb7_11190159
53.5 Fvb7_10604855
54.4 Fvb7_8929300 Fvb7_8929310
55.1 Fvb2_15319568
55.6 Fvb7_12651378
0.0 Fvb7_23127137
3.7 Fvb7_22218611 Fvb7_21725878
7.2 Fvb7_21735148
13.5 Fvb7_20884927 Fvb7_20884949
18.4 Fvb7_20420086
23.8 Fvb7_19543147
24.8 Fvb7_19741283
25.5 Fvb7_19552407
33.6 Fvb7_18492129
46.1 Fvb2_16109652
48.0 Fvb7_14071613
49.7 Fvb7_15524417
56.3 Fvb7_12651378
60.5 Fvb2_15319568
62.3 Fvb7_10604855 Fvb7_11190159
64.0 Fvb7_8929310 Fvb7_8929300
66.3 Fvb2_1972576
68.7 Fvb7_10575600
71.1 Fvb2_25788256
Honeoye_11
0.0 Fvb7_557157
9.5 Fvb7_2257160
9.7 Fvb4_1561397
9.8 Fvb7_4379313
10.1 Fvb7_4479782
10.2 Fvb7_4330281
11.5 Fvb7_6460231
11.8 Fvb7_6262514
20.5 Fvb7_14071640
20.6 Fvb7_14071656
22.2 Fvb7_14384465
35.9 Fvb7_17125454
41.0 Fvb7_18048262
42.6 Fvb7_18508065
45.8 Fvb7_19861130 Fvb7_20420064
54.3 Fvb7_21180855
78.5 Fvb7_23616844
0.0 Fvb7_557157
10.1 Fvb7_2257160 Fvb7_4479782
Fvb4_1561397 Fvb7_4379313
Fvb7_4330281
11.9 Fvb7_6262514 Fvb7_6460231
21.1 Fvb7_14071656 Fvb7_14071640
23.1 Fvb7_14384465
36.4 Fvb7_17125454
40.8 Fvb7_18048262
45.3 Fvb7_18508065
48.8 Fvb7_20420064 Fvb7_19861130
56.8 Fvb7_21180855
77.8 Fvb7_23616844

Fvb_7

Honeoye_13

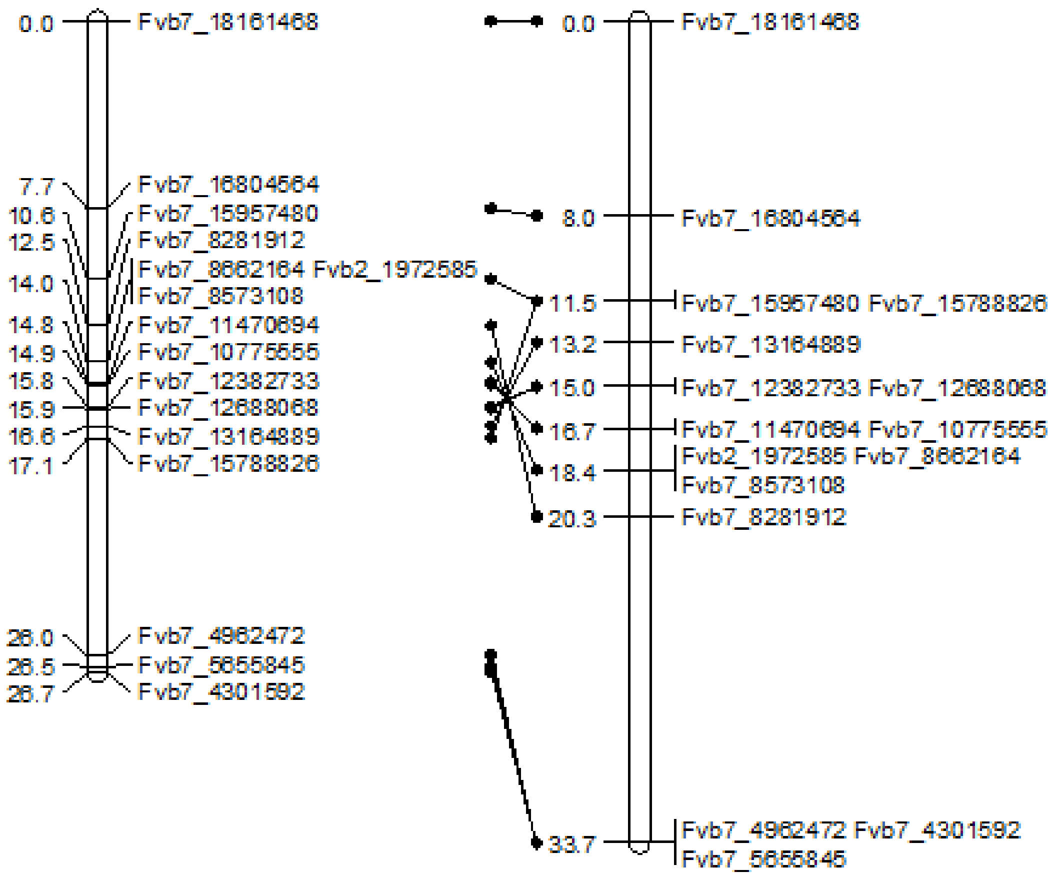

Honeoye_33

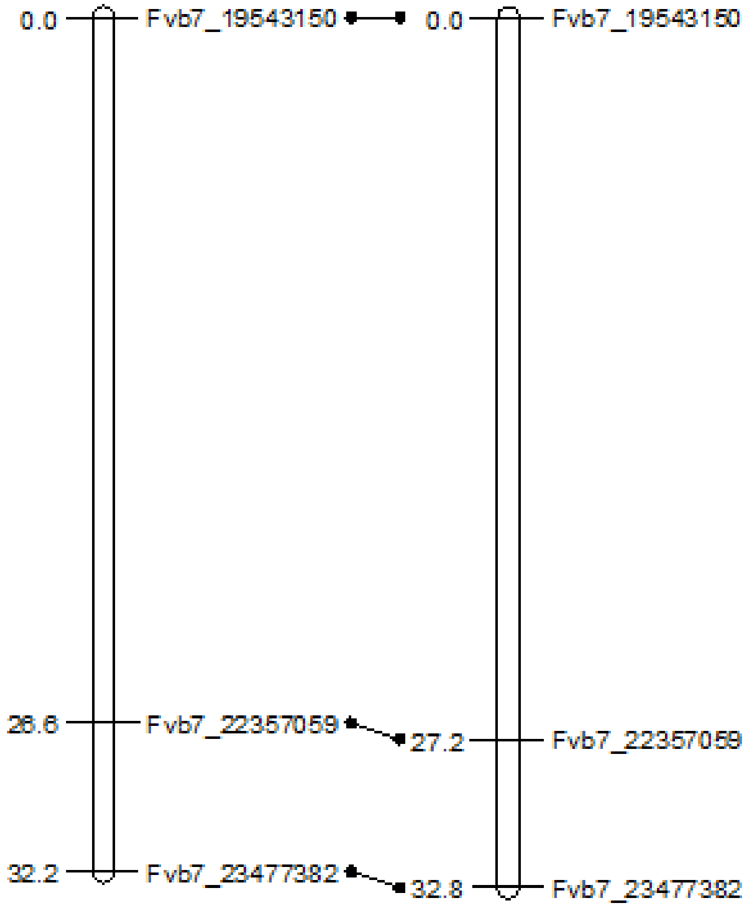

Supplement: File S2 [file peerj-05-3731-s003.pdf]
