# Supplementary material for: Genotyping-by-sequencing enables linkage mapping in three octoploid cultivated strawberry families
Source: PeerJ. 2017 Aug 30;5:e3731. doi: 10.7717/peerj.3731 (PMC5581533; doi:10.7717/peerj.3731)

Fvb_1

Redgauntlet_19

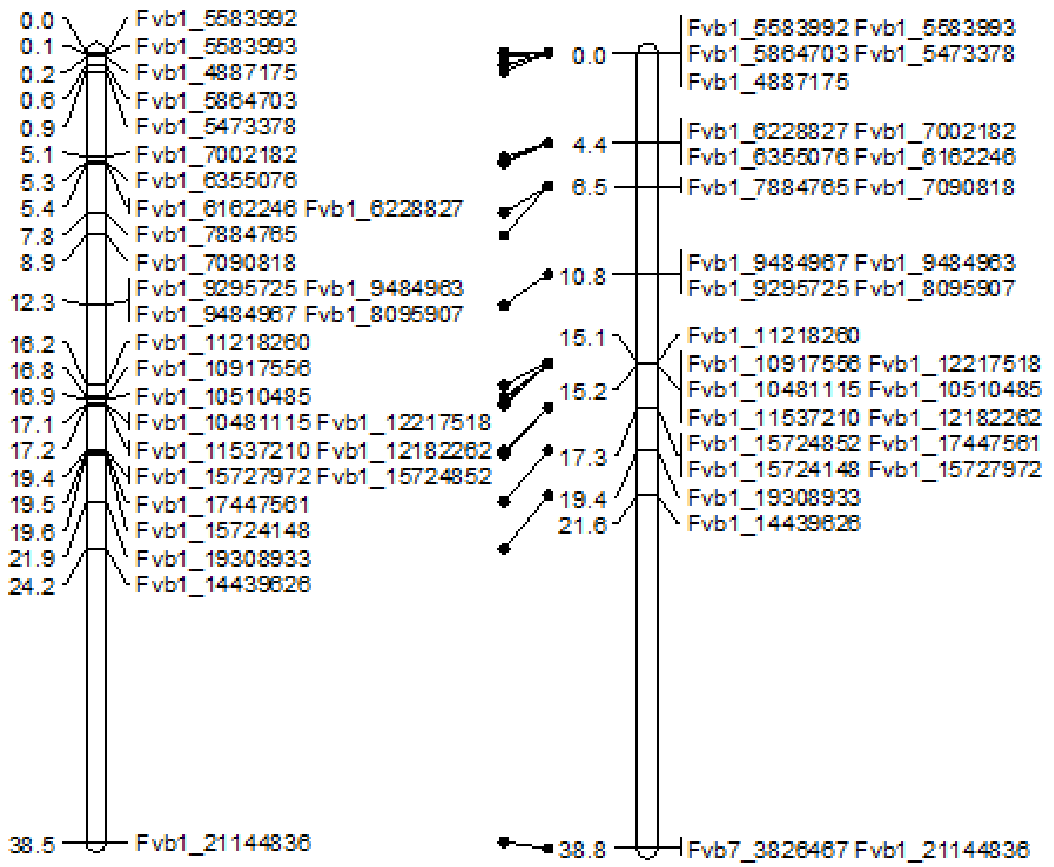

Redgauntlet_29

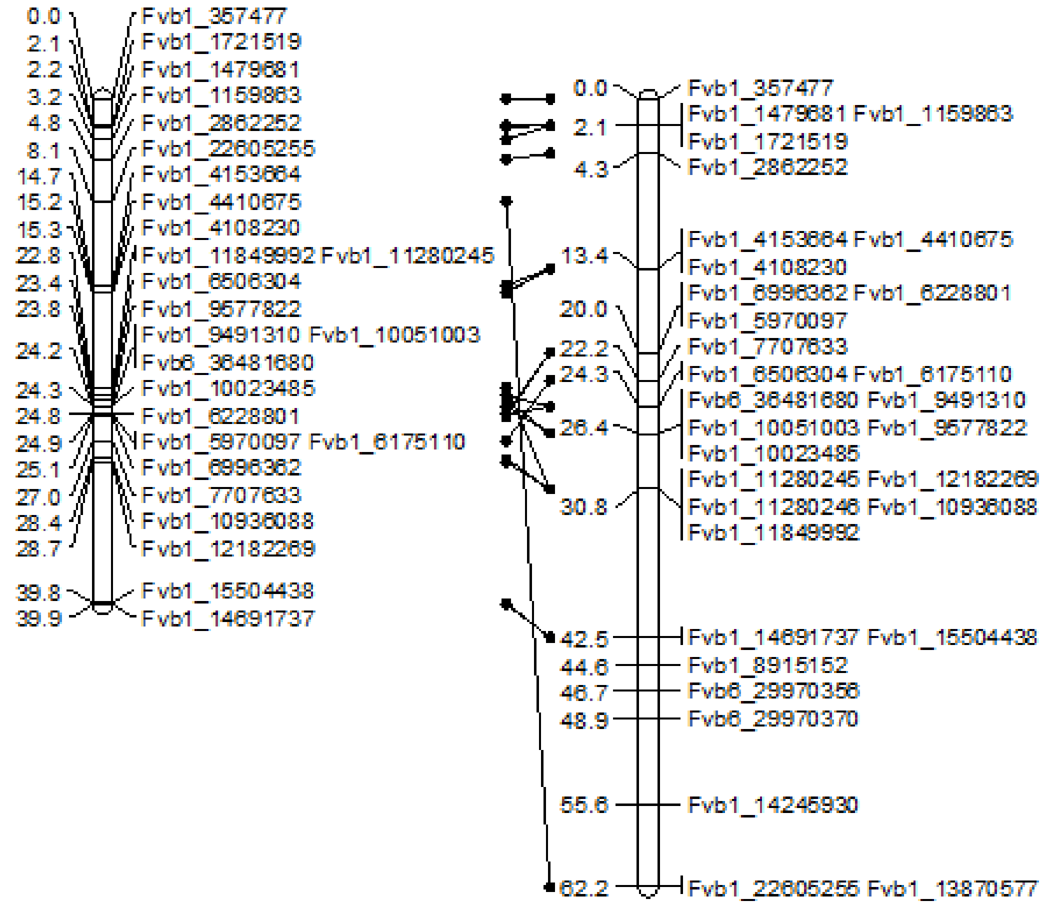

Fvb_1

Redgauntlet_38

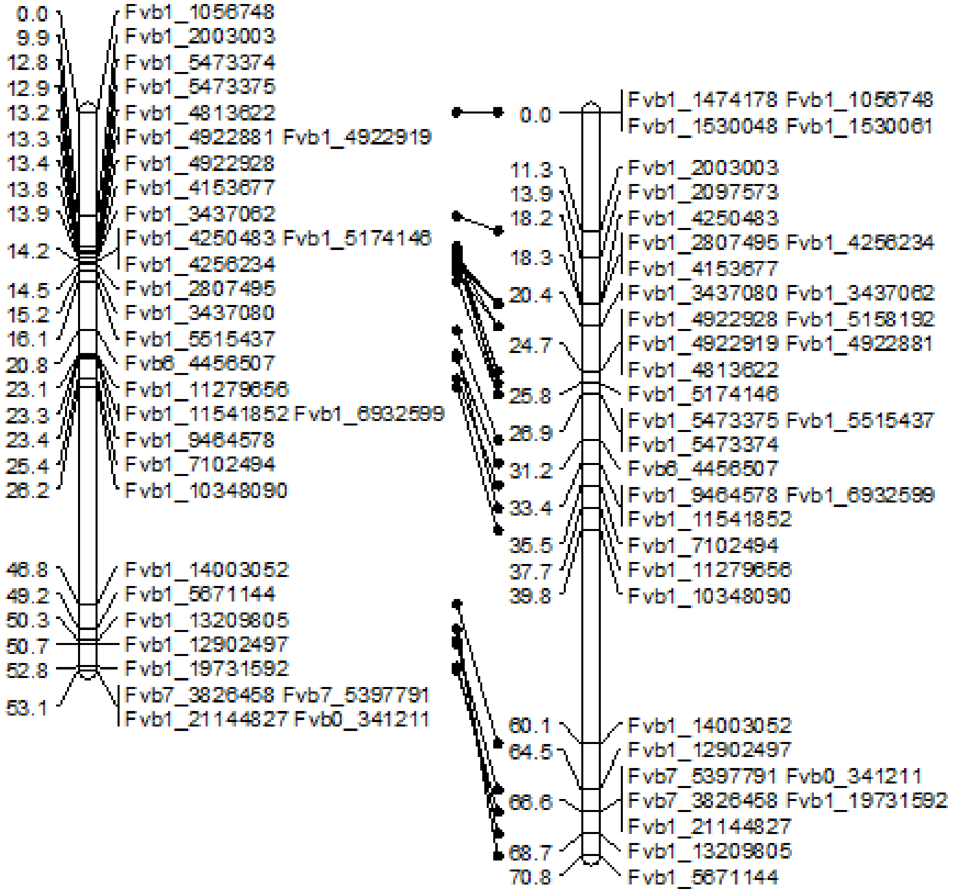

Redgauntlet_39

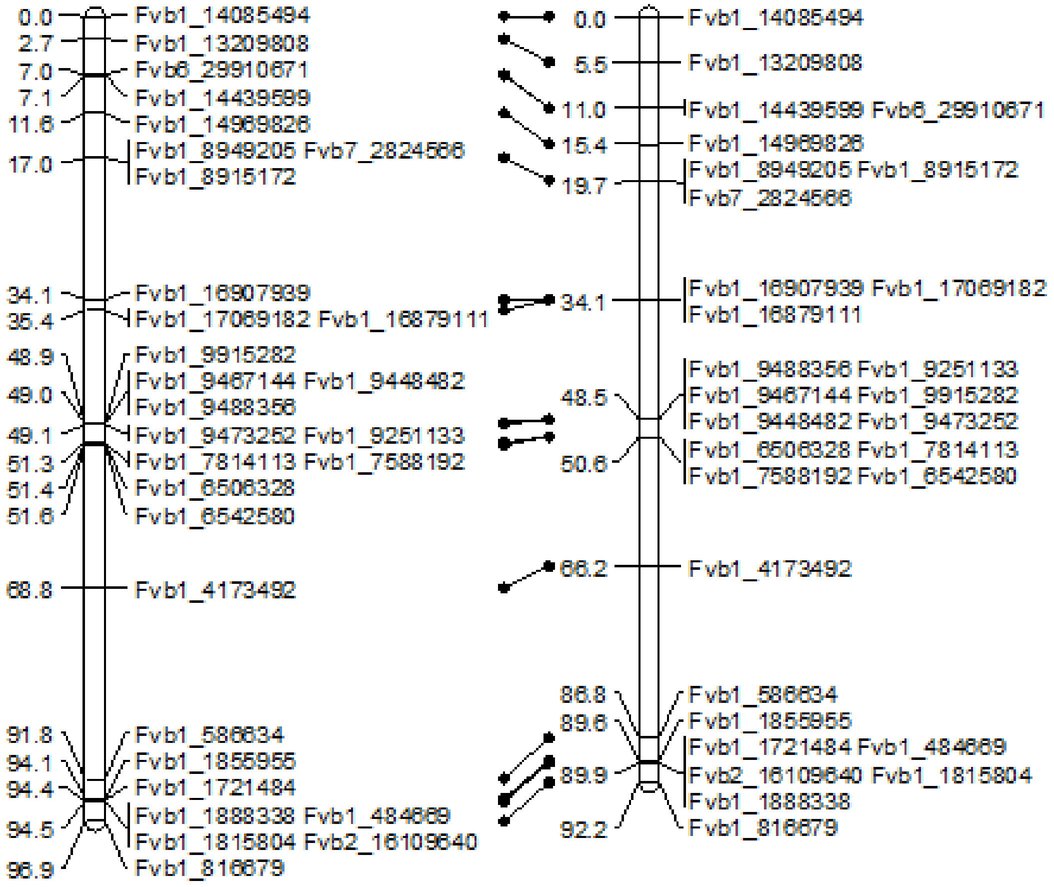

Fvb_1

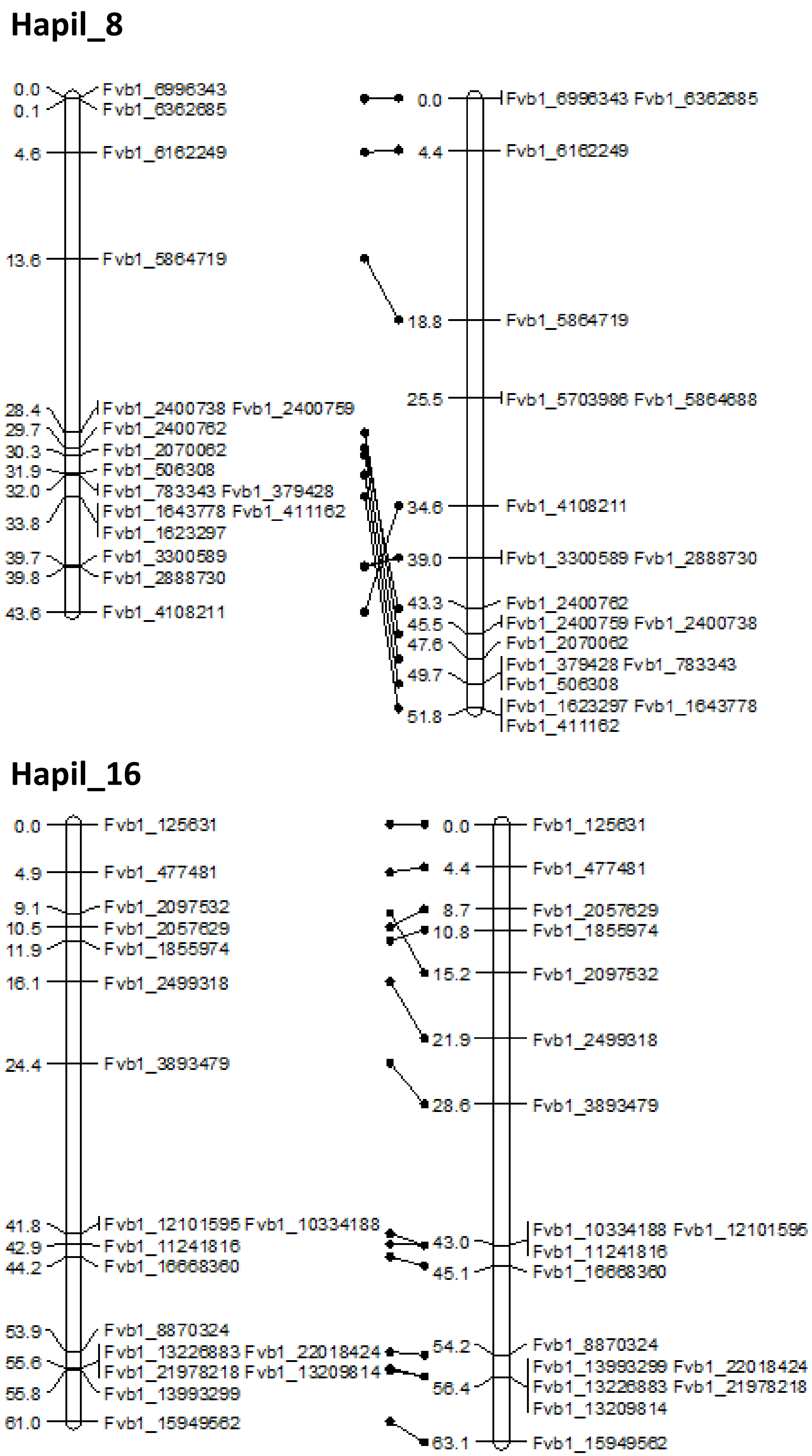
Hapil_8
0.0 Fvb1_6996343
0.1 Fvb1_6362685
4.6 Fvb1_6162249
13.6 Fvb1_5864719
28.4 Fvb1_2400738 Fvb1_2400759
29.7 Fvb1_2400762
30.3 Fvb1_2070062
31.9 Fvb1_506308
32.0 Fvb1_783343 Fvb1_379428
33.8 Fvb1_1643778 Fvb1_411162 Fvb1_1623297
39.7 Fvb1_3300589
39.8 Fvb1_2888730
43.6 Fvb1_4108211
0.0 Fvb1_6996343 Fvb1_6362685
4.4 Fvb1_6162249
18.8 Fvb1_5864719
25.5 Fvb1_5703986 Fvb1_5864688
34.6 Fvb1_4108211
39.0 Fvb1_3300589 Fvb1_2888730
43.3 Fvb1_2400762
45.5 Fvb1_2400759 Fvb1_2400738
47.6 Fvb1_2070062
49.7 Fvb1_379428 Fvb1_783343 Fvb1_506308
51.8 Fvb1_1623297 Fvb1_1643778 Fvb1_411162
Hapil_16
0.0 Fvb1_125631
4.9 Fvb1_477481
9.1 Fvb1_2097532
10.5 Fvb1_2057629
11.9 Fvb1_1855974
16.1 Fvb1_2499318
24.4 Fvb1_3893479
41.8 Fvb1_12101595 Fvb1_10334188
42.9 Fvb1_11241816
44.2 Fvb1_16668360
53.9 Fvb1_8870324
55.6 Fvb1_13226883 Fvb1_22018424 Fvb1_21978218 Fvb1_13209814
55.8 Fvb1_13993299
61.0 Fvb1_15949562
0.0 Fvb1_125631
4.4 Fvb1_477481
8.7 Fvb1_2057629
10.8 Fvb1_1855974
15.2 Fvb1_2097532
21.9 Fvb1_2499318
28.6 Fvb1_3893479
43.0 Fvb1_10334188 Fvb1_12101595 Fvb1_11241816
45.1 Fvb1_16668360
54.2 Fvb1_8870324
56.4 Fvb1_13993299 Fvb1_22018424 Fvb1_13226883 Fvb1_21978218 Fvb1_13209814
63.1 Fvb1_15949562

Fvb_1

Hapil_18

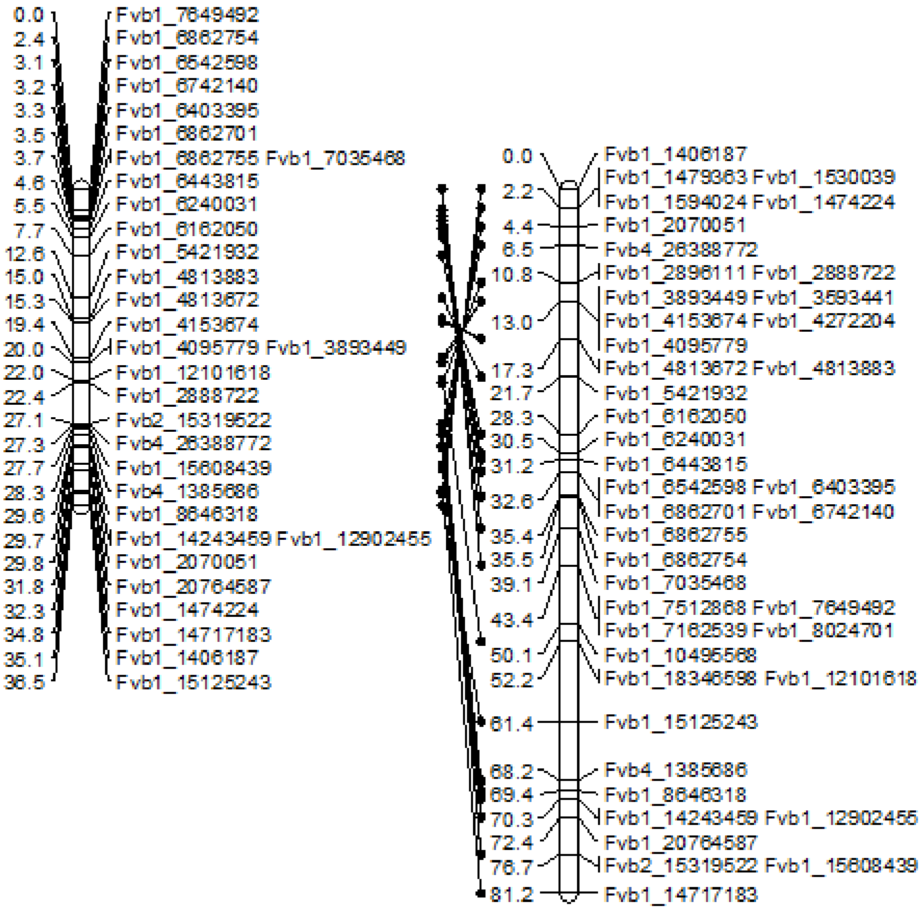

Hapil_27

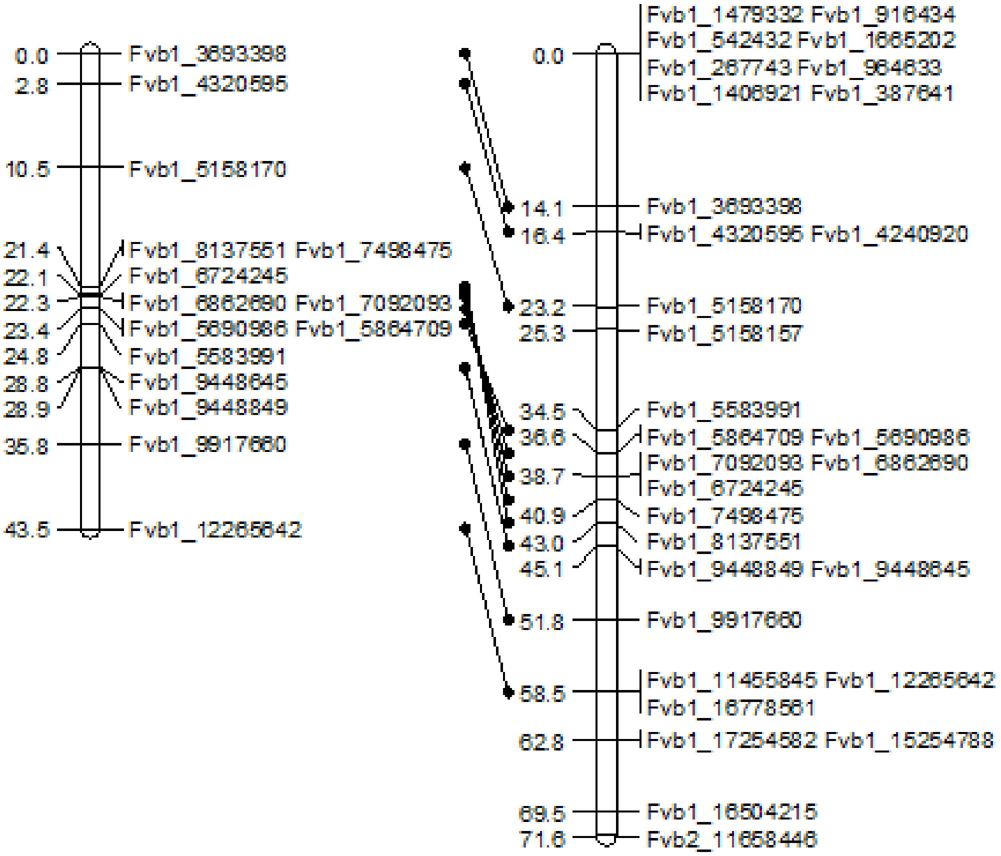

Fvb_2

Redgauntlet_1

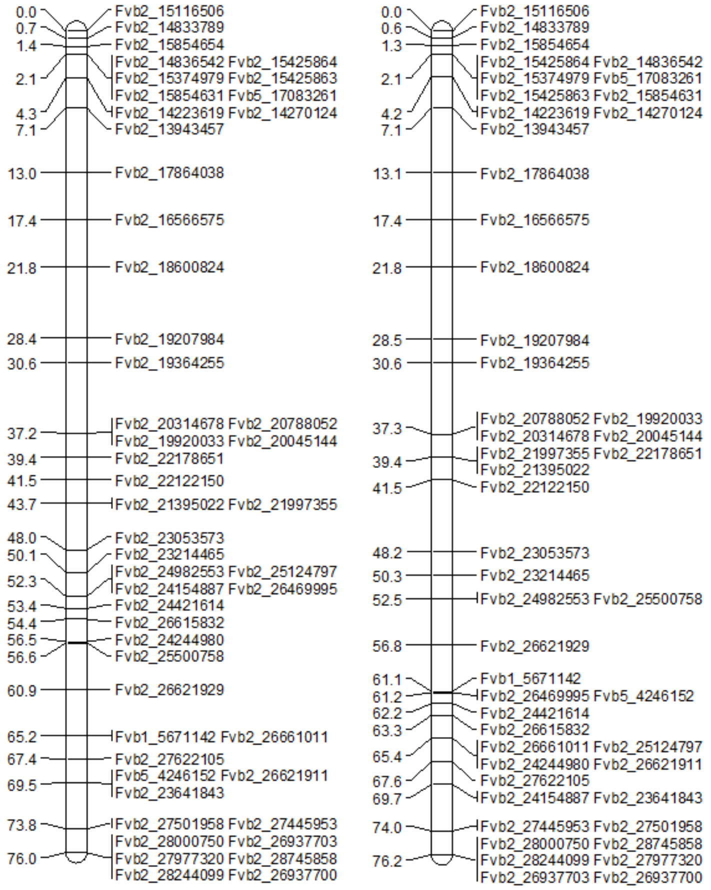

Redgauntlet_4

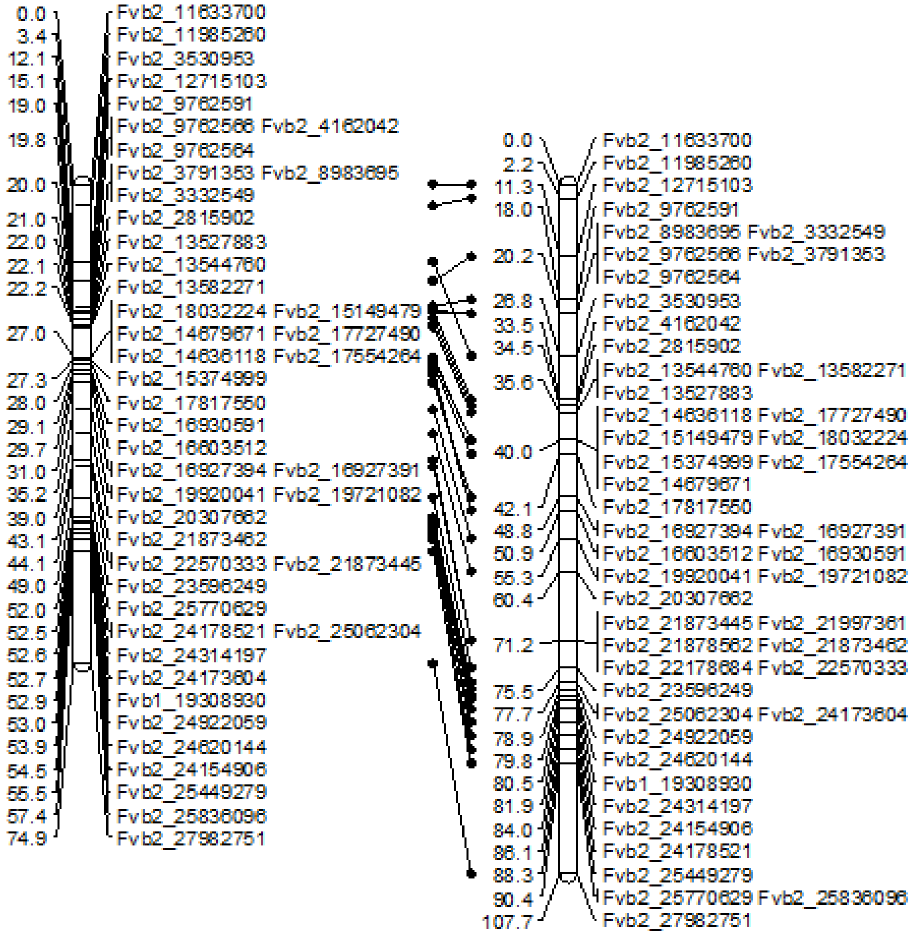

Fvb_2

Redgauntlet_9

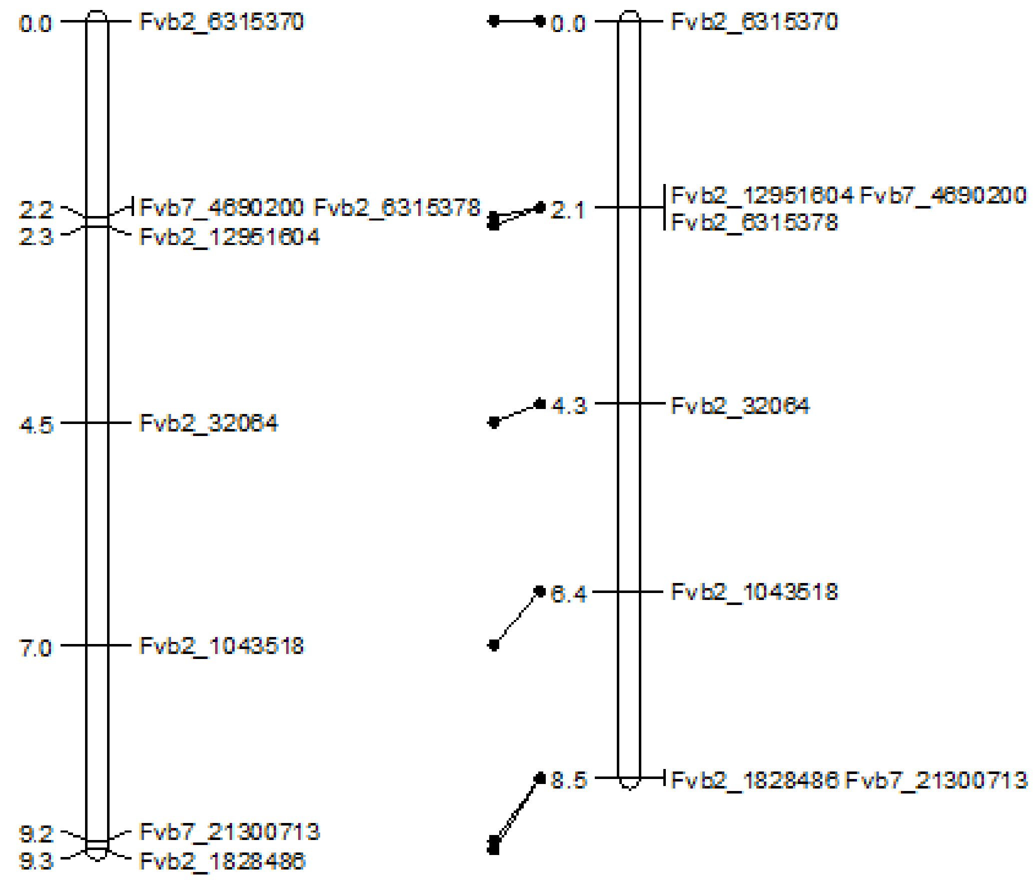

Redgauntlet_14

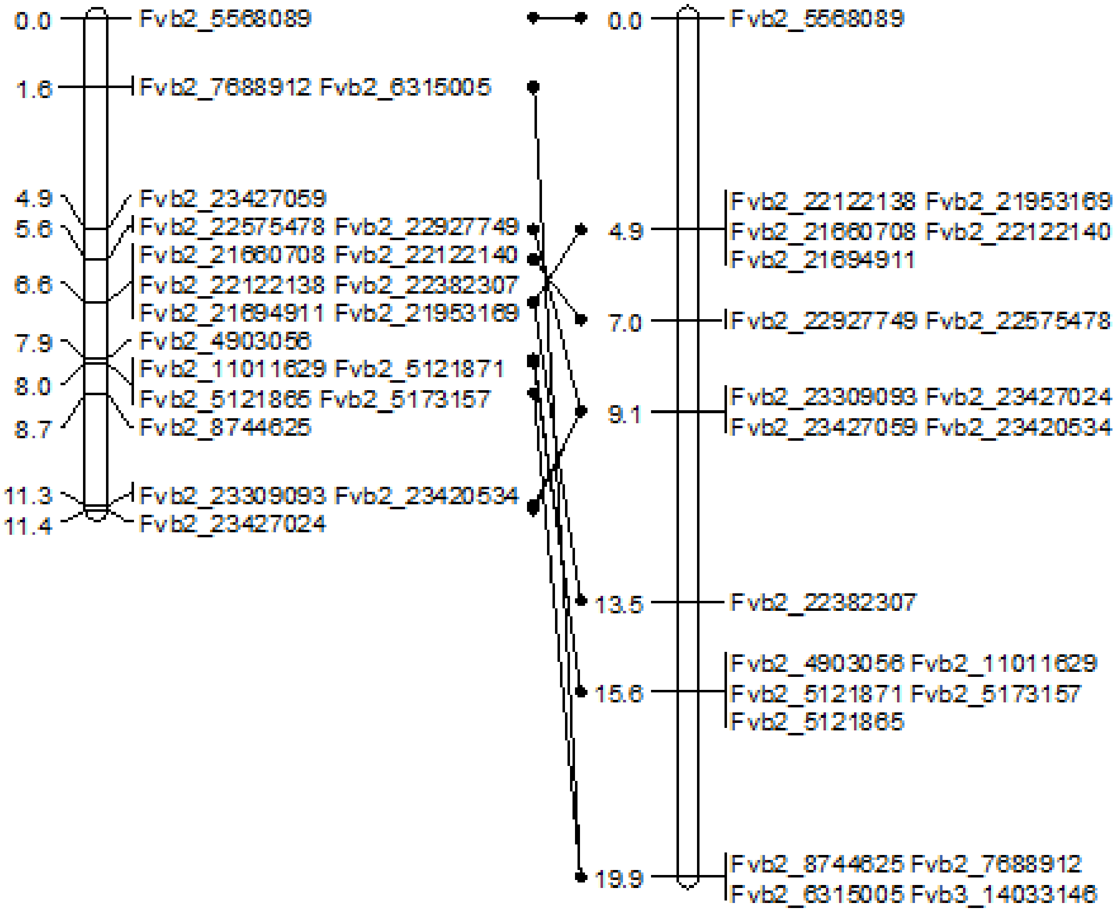

Fvb_2

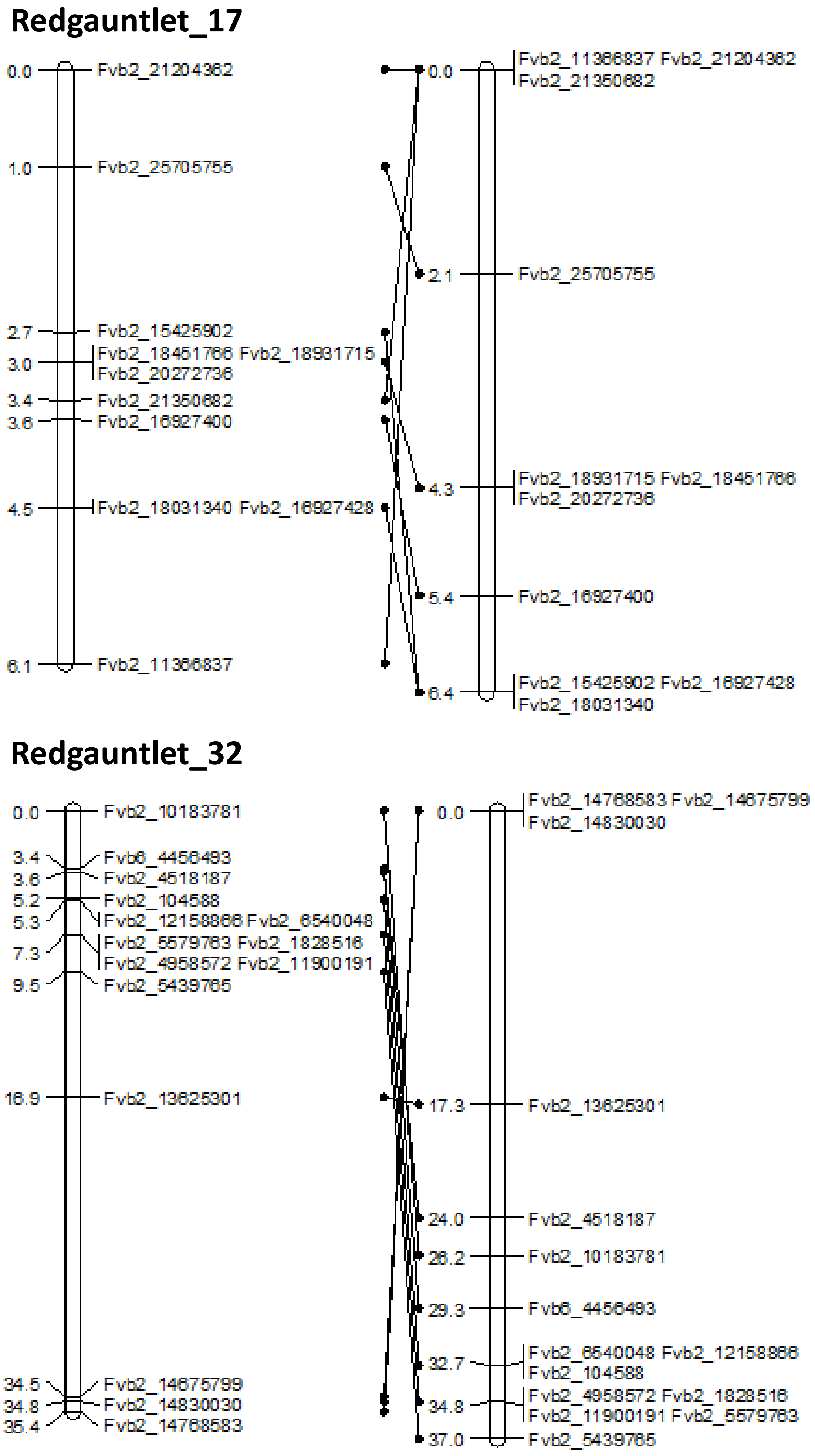
Redgauntlet_17
0.0 Fvb2_21204362
1.0 Fvb2_25705755
2.7 Fvb2_15425902
3.0 Fvb2_18451766 Fvb2_18931715 Fvb2_20272736
3.4 Fvb2_21350682
3.6 Fvb2_16927400
4.5 Fvb2_18031340 Fvb2_16927428
6.1 Fvb2_11366837
0.0 Fvb2_11366837 Fvb2_21204362 Fvb2_21350682
2.1 Fvb2_25705755
4.3 Fvb2_18931715 Fvb2_18451766 Fvb2_20272736
5.4 Fvb2_16927400
6.4 Fvb2_15425902 Fvb2_16927428 Fvb2_18031340
Redgauntlet_32
0.0 Fvb2_10183781
3.4 Fvb6_4456493
3.6 Fvb2_4518187
5.2 Fvb2_104588
5.3 Fvb2_12158866 Fvb2_6540048
7.3 Fvb2_5579763 Fvb2_1828516 Fvb2_4958572 Fvb2_11900191
9.5 Fvb2_5439765
16.9 Fvb2_13625301
34.5 Fvb2_14675799
34.8 Fvb2_14830030
35.4 Fvb2_14768583
0.0 Fvb2_14768583 Fvb2_14675799 Fvb2_14830030
17.3 Fvb2_13625301
24.0 Fvb2_4518187
26.2 Fvb2_10183781
29.3 Fvb6_4456493
32.7 Fvb2_6540048 Fvb2_12158866 Fvb2_104588
34.8 Fvb2_4958572 Fvb2_1828516 Fvb2_11900191 Fvb2_5579763
37.0 Fvb2_5439765

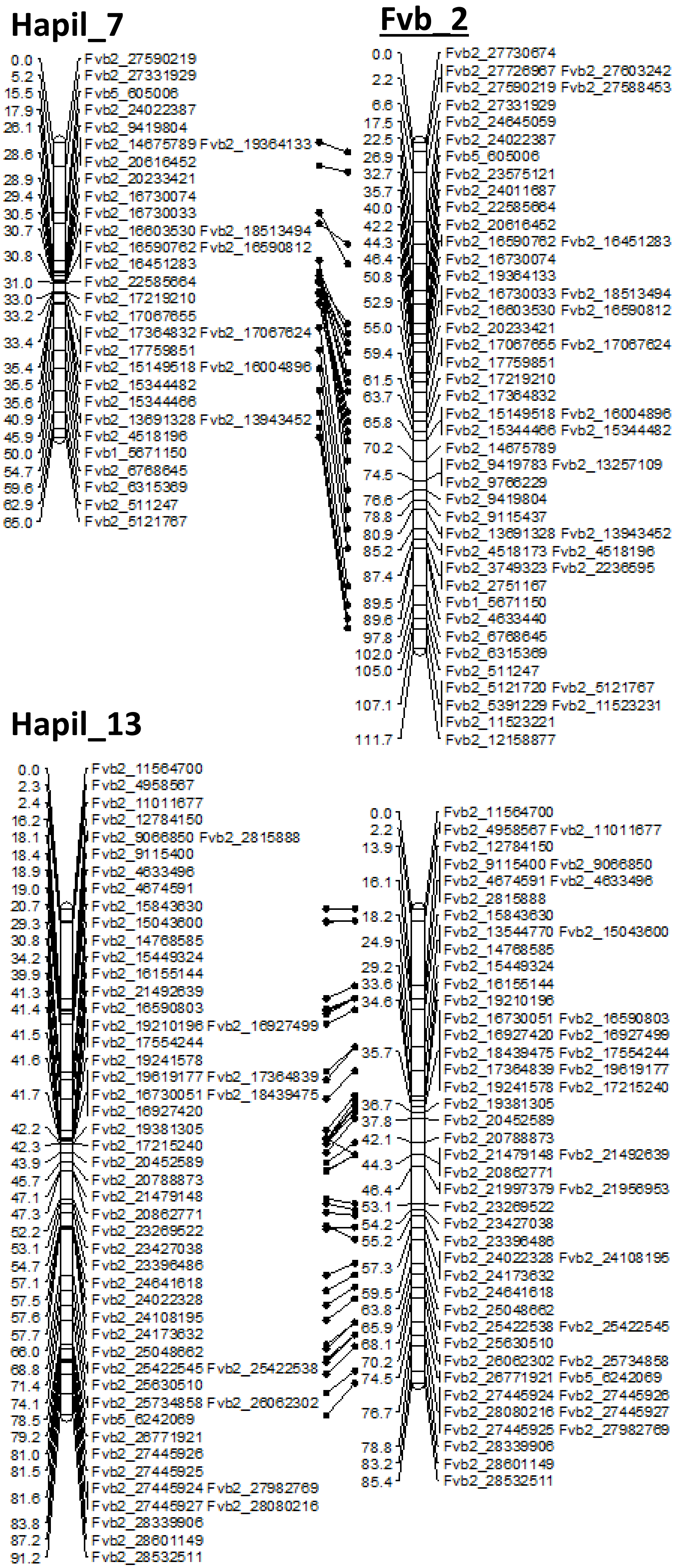

Hapil_7
0.0 Fvb2_27590219
5.2 Fvb2_27331929
15.5 Fvb5_605006
17.9 Fvb2_24022387
26.1 Fvb2_9419804
28.6 Fvb2_14675789 Fvb2_19364133
Fvb2_20616452
28.9 Fvb2_20233421
29.4 Fvb2_16730074
30.5 Fvb2_16730033
30.7 Fvb2_16603530 Fvb2_18513494
30.8 Fvb2_16590762 Fvb2_16590812
Fvb2_16451283
31.0 Fvb2_22585664
33.0 Fvb2_17219210
33.2 Fvb2_17067655
33.4 Fvb2_17364832 Fvb2_17067624
Fvb2_17759851
35.4 Fvb2_15149518 Fvb2_16004896
35.5 Fvb2_15344482
35.6 Fvb2_15344466
40.9 Fvb2_13691328 Fvb2_13943452
45.9 Fvb2_4518196
50.0 Fvb1_5671150
54.7 Fvb2_6768645
59.6 Fvb2_6315369
62.9 Fvb2_511247
65.0 Fvb2_5121767
Fvb_2
0.0 Fvb2_27730674
2.2 Fvb2_27726967 Fvb2_27603242
Fvb2_27590219 Fvb2_27588453
6.6 Fvb2_27331929
17.5 Fvb2_24645059
22.5 Fvb2_24022387
26.9 Fvb5_605006
32.7 Fvb2_23575121
35.7 Fvb2_24011687
40.0 Fvb2_22585664
42.2 Fvb2_20616452
44.3 Fvb2_16590762 Fvb2_16451283
46.4 Fvb2_16730074
50.8 Fvb2_19364133
52.9 Fvb2_16730033 Fvb2_18513494
Fvb2_16603530 Fvb2_16590812
55.0 Fvb2_20233421
59.4 Fvb2_17067655 Fvb2_17067624
Fvb2_17759851
61.5 Fvb2_17219210
63.7 Fvb2_17364832
65.8 Fvb2_15149518 Fvb2_16004896
Fvb2_15344466 Fvb2_15344482
70.2 Fvb2_14675789
74.5 Fvb2_9419783 Fvb2_13257109
Fvb2_9766229
76.6 Fvb2_9419804
78.8 Fvb2_9115437
80.9 Fvb2_13691328 Fvb2_13943452
85.2 Fvb2_4518173 Fvb2_4518196
87.4 Fvb2_3749323 Fvb2_2236595
Fvb2_2751167
89.5 Fvb1_5671150
89.6 Fvb2_4633440
97.8 Fvb2_6768645
102.0 Fvb2_6315369
105.0 Fvb2_511247
107.1 Fvb2_5121720 Fvb2_5121767
Fvb2_5391229 Fvb2_11523231
Fvb2_11523221
111.7 Fvb2_12158877
Hapil_13
0.0 Fvb2_11564700
2.3 Fvb2_4958567
2.4 Fvb2_11011677
16.2 Fvb2_12784150
18.1 Fvb2_9066850 Fvb2_2815888
18.4 Fvb2_9115400
18.9 Fvb2_4633496
19.0 Fvb2_4674591
20.7 Fvb2_15843630
29.3 Fvb2_15043600
30.8 Fvb2_14768585
34.2 Fvb2_15449324
39.9 Fvb2_16155144
41.3 Fvb2_21492639
41.4 Fvb2_16590803
41.5 Fvb2_19210196 Fvb2_16927499
Fvb2_17554244
41.6 Fvb2_19241578
41.7 Fvb2_19619177 Fvb2_17364839
Fvb2_16730051 Fvb2_18439475
Fvb2_16927420
42.2 Fvb2_19381305
42.3 Fvb2_17215240
43.9 Fvb2_20452589
45.7 Fvb2_20788873
47.1 Fvb2_21479148
47.3 Fvb2_20862771
52.2 Fvb2_23269522
53.1 Fvb2_23427038
54.7 Fvb2_23396486
57.1 Fvb2_24641618
57.5 Fvb2_24022328
57.6 Fvb2_24108195
57.7 Fvb2_24173632
66.0 Fvb2_25048662
68.8 Fvb2_25422545 Fvb2_25422538
71.4 Fvb2_25630510
74.1 Fvb2_25734858 Fvb2_26062302
78.5 Fvb5_6242069
79.2 Fvb2_26771921
81.0 Fvb2_27445926
81.5 Fvb2_27445925
81.6 Fvb2_27445924 Fvb2_27982769
Fvb2_27445927 Fvb2_28080216
83.8 Fvb2_28339906
87.2 Fvb2_28601149
91.2 Fvb2_28532511
0.0 Fvb2_11564700
2.2 Fvb2_4958567 Fvb2_11011677
13.9 Fvb2_12784150
16.1 Fvb2_9115400 Fvb2_9066850
Fvb2_4674591 Fvb2_4633496
Fvb2_2815888
18.2 Fvb2_15843630
24.9 Fvb2_13544770 Fvb2_15043600
Fvb2_14768585
29.2 Fvb2_15449324
33.6 Fvb2_16155144
34.6 Fvb2_19210196
35.7 Fvb2_16730051 Fvb2_16590803
Fvb2_16927420 Fvb2_16927499
Fvb2_18439475 Fvb2_17554244
Fvb2_17364839 Fvb2_19619177
Fvb2_19241578 Fvb2_17215240
36.7 Fvb2_19381305
37.8 Fvb2_20452589
42.1 Fvb2_20788873
44.3 Fvb2_21479148 Fvb2_21492639
Fvb2_20862771
46.4 Fvb2_21997379 Fvb2_21956953
53.1 Fvb2_23269522
54.2 Fvb2_23427038
55.2 Fvb2_23396486
57.3 Fvb2_24022328 Fvb2_24108195
Fvb2_24173632
59.5 Fvb2_24641618
63.8 Fvb2_25048662
65.9 Fvb2_25422538 Fvb2_25422545
68.1 Fvb2_25630510
70.2 Fvb2_26062302 Fvb2_25734858
74.5 Fvb2_26771921 Fvb5_6242069
76.7 Fvb2_27445924 Fvb2_27445926
Fvb2_28080216 Fvb2_27445927
Fvb2_27445925 Fvb2_27982769
78.8 Fvb2_28339906
83.2 Fvb2_28601149
85.4 Fvb2_28532511

Fvb_2

Hapil_15

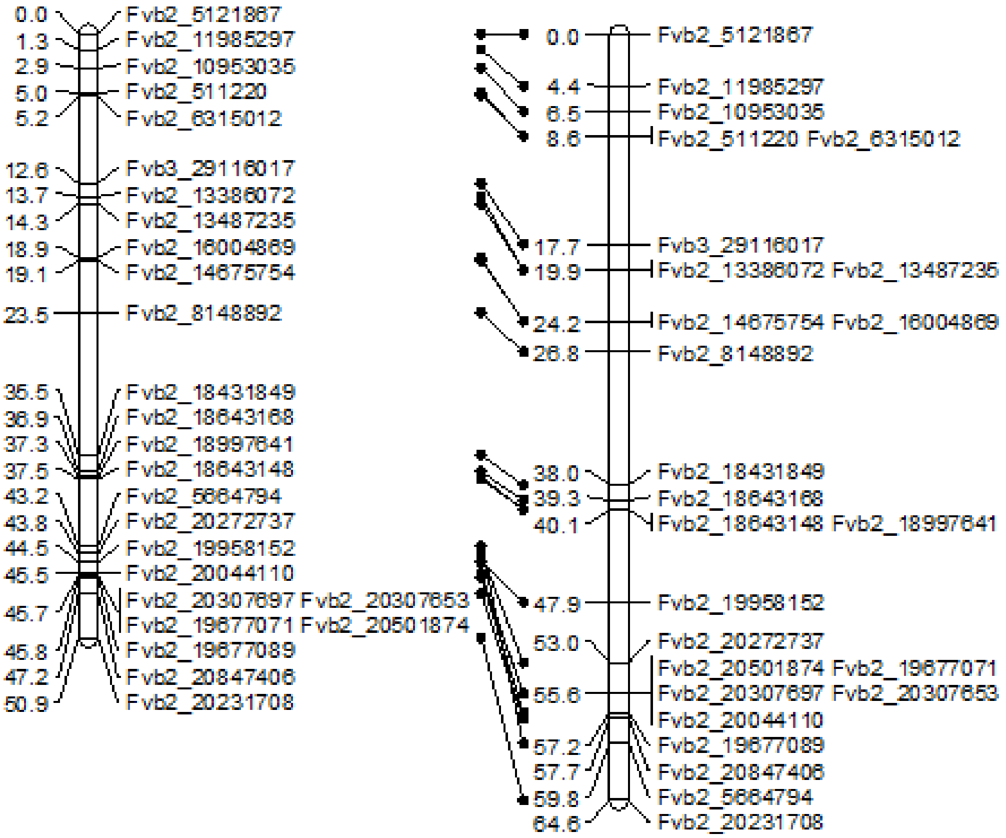

Hapil_26

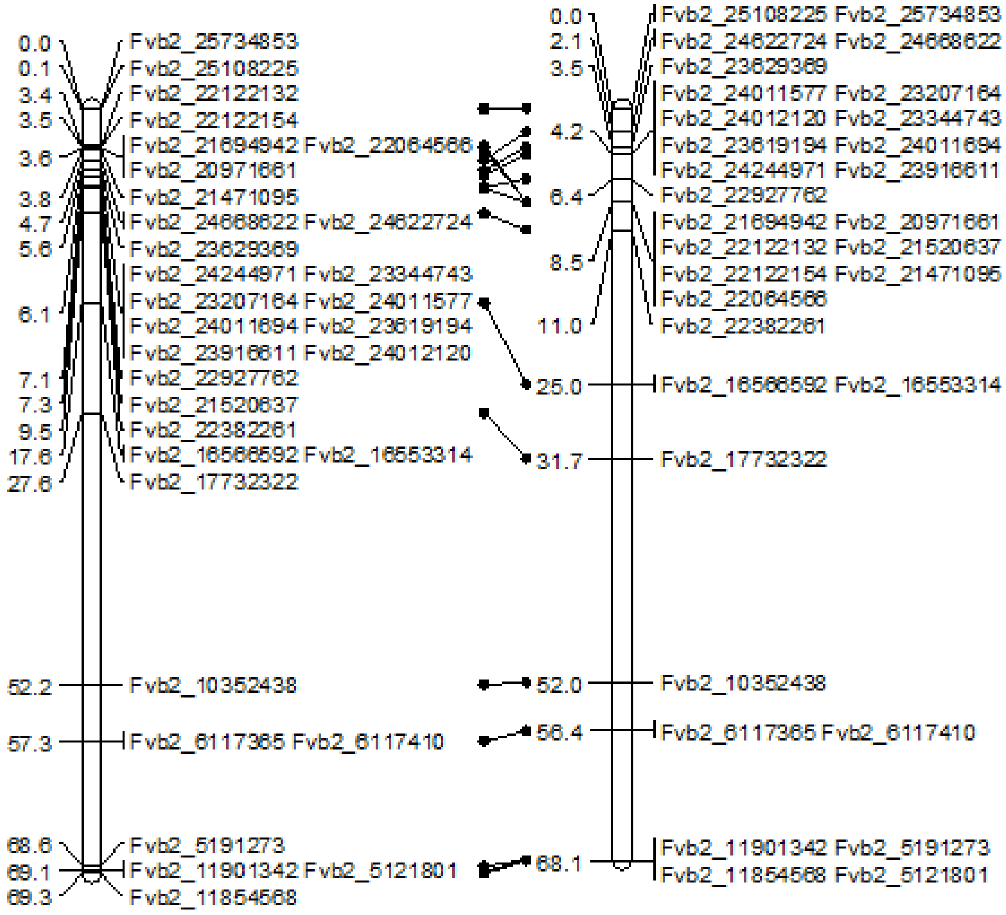

Fvb_3

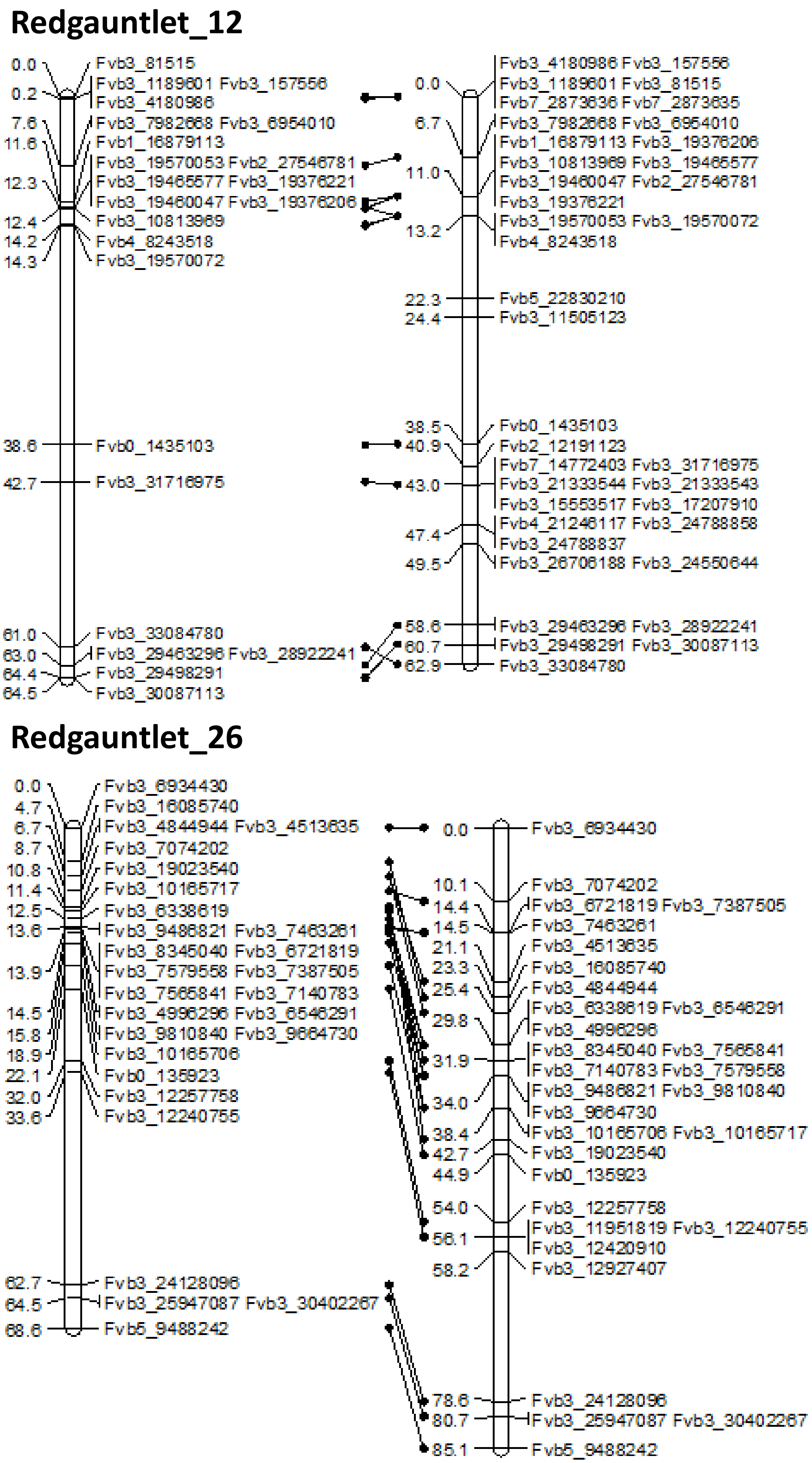
Redgauntlet_12
0.0 Fvb3_81515
0.2 Fvb3_1189601 Fvb3_157556
Fvb3_4180986
7.6 Fvb3_7982668 Fvb3_6954010
11.6 Fvb1_16879113
Fvb3_19570053 Fvb2_27546781
12.3 Fvb3_19465577 Fvb3_19376221
Fvb3_19460047 Fvb3_19376206
12.4 Fvb3_10813969
14.2 Fvb4_8243518
14.3 Fvb3_19570072
38.6 Fvb0_1435103
42.7 Fvb3_31716975
61.0 Fvb3_33084780
63.0 Fvb3_29463296 Fvb3_28922241
64.4 Fvb3_29498291
64.5 Fvb3_30087113
Fvb3_4180986 Fvb3_157556
0.0 Fvb3_1189601 Fvb3_81515
Fvb7_2873636 Fvb7_2873635
6.7 Fvb3_7982668 Fvb3_6954010
Fvb1_16879113 Fvb3_19376206
Fvb3_10813969 Fvb3_19465577
11.0 Fvb3_19460047 Fvb2_27546781
Fvb3_19376221
13.2 Fvb3_19570053 Fvb3_19570072
Fvb4_8243518
22.3 Fvb5_22830210
24.4 Fvb3_11505123
38.5 Fvb0_1435103
40.9 Fvb2_12191123
Fvb7_14772403 Fvb3_31716975
43.0 Fvb3_21333544 Fvb3_21333543
Fvb3_15553517 Fvb3_17207910
47.4 Fvb4_21246117 Fvb3_24788858
Fvb3_24788837
49.5 Fvb3_26706188 Fvb3_24550644
58.6 Fvb3_29463296 Fvb3_28922241
60.7 Fvb3_29498291 Fvb3_30087113
62.9 Fvb3_33084780
Redgauntlet_26
0.0 Fvb3_6934430
4.7 Fvb3_16085740
6.7 Fvb3_4844944 Fvb3_4513635
8.7 Fvb3_7074202
10.8 Fvb3_19023540
11.4 Fvb3_10165717
12.5 Fvb3_6338619
13.6 Fvb3_9486821 Fvb3_7463261
Fvb3_8345040 Fvb3_6721819
13.9 Fvb3_7579558 Fvb3_7387505
Fvb3_7565841 Fvb3_7140783
14.5 Fvb3_4996296 Fvb3_6546291
15.8 Fvb3_9810840 Fvb3_9664730
18.9 Fvb3_10165706
22.1 Fvb0_135923
32.0 Fvb3_12257758
33.6 Fvb3_12240755
62.7 Fvb3_24128096
64.5 Fvb3_25947087 Fvb3_30402267
68.6 Fvb5_9488242
0.0 Fvb3_6934430
10.1 Fvb3_7074202
14.4 Fvb3_6721819 Fvb3_7387505
14.5 Fvb3_7463261
21.1 Fvb3_4513635
23.3 Fvb3_16085740
25.4 Fvb3_4844944
Fvb3_6338619 Fvb3_6546291
29.8 Fvb3_4996296
Fvb3_8345040 Fvb3_7565841
31.9 Fvb3_7140783 Fvb3_7579558
Fvb3_9486821 Fvb3_9810840
34.0 Fvb3_9664730
38.4 Fvb3_10165706 Fvb3_10165717
42.7 Fvb3_19023540
44.9 Fvb0_135923
54.0 Fvb3_12257758
Fvb3_11951819 Fvb3_12240755
56.1 Fvb3_12420910
58.2 Fvb3_12927407
78.6 Fvb3_24128096
80.7 Fvb3_25947087 Fvb3_30402267
85.1 Fvb5_9488242

Fvb_3

Redgauntlet_34

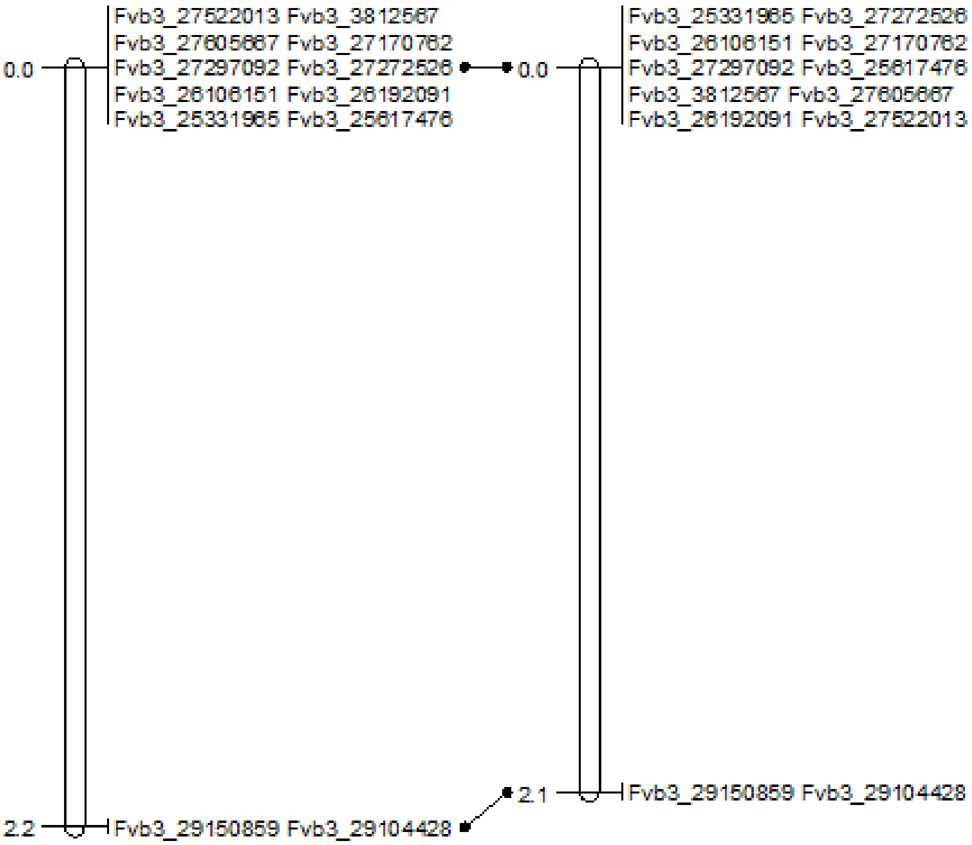

Redgauntlet_41

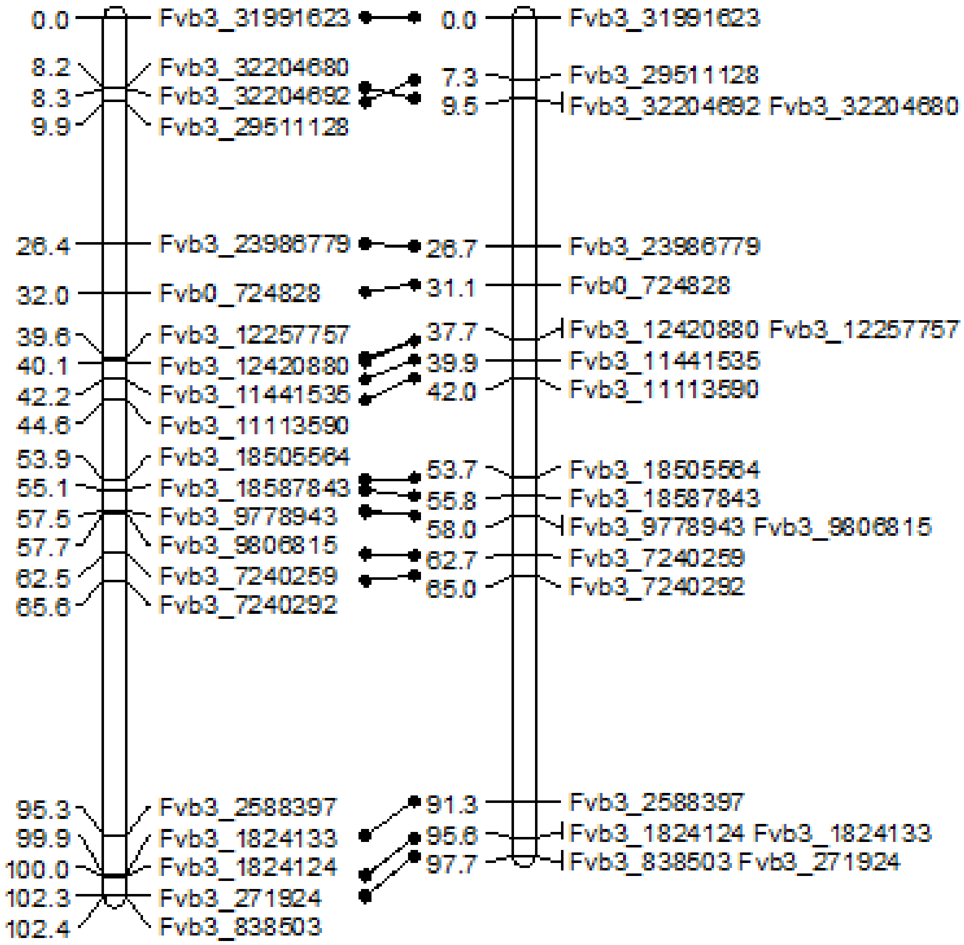

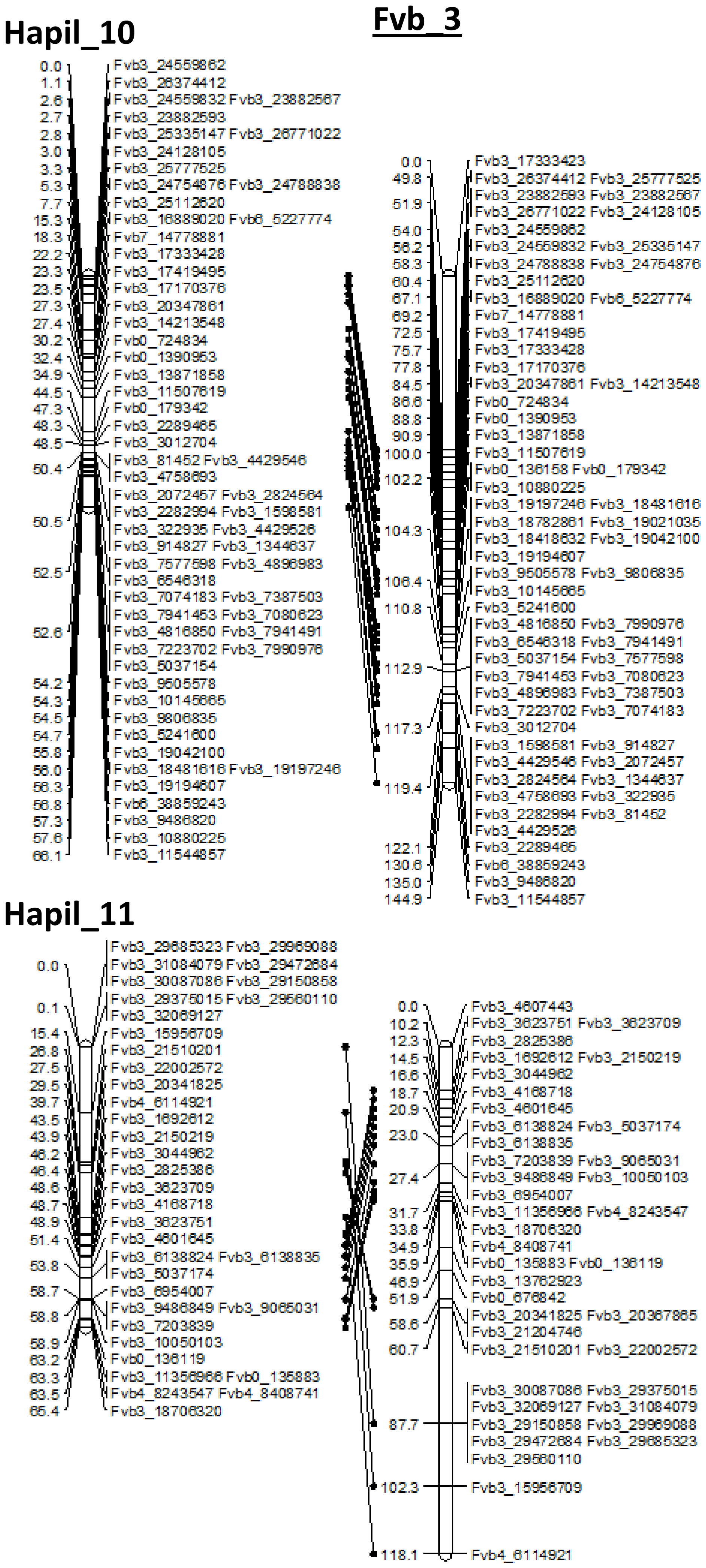
Hapil_10
Fvb_3
0.0 Fvb3_24559862
1.1 Fvb3_26374412
2.6 Fvb3_24559832 Fvb3_23882567
2.7 Fvb3_23882593
2.8 Fvb3_25335147 Fvb3_26771022
3.0 Fvb3_24128105
3.3 Fvb3_25777525
5.3 Fvb3_24754876 Fvb3_24788838
7.7 Fvb3_25112620
15.3 Fvb3_16889020 Fvb6_5227774
18.3 Fvb7_14778881
22.2 Fvb3_17333428
23.3 Fvb3_17419495
23.5 Fvb3_17170376
27.3 Fvb3_20347861
27.4 Fvb3_14213548
30.2 Fvb0_724834
32.4 Fvb0_1390953
34.9 Fvb3_13871858
44.5 Fvb3_11507619
47.3 Fvb0_179342
48.3 Fvb3_2289465
48.5 Fvb3_3012704
50.4 Fvb3_81452 Fvb3_4429546
Fvb3_4758693
50.5 Fvb3_2072457 Fvb3_2824564
Fvb3_2282994 Fvb3_1598581
Fvb3_322935 Fvb3_4429526
Fvb3_914827 Fvb3_1344637
52.5 Fvb3_7577598 Fvb3_4896983
Fvb3_6546318
52.6 Fvb3_7074183 Fvb3_7387503
Fvb3_7941453 Fvb3_7080623
Fvb3_4816850 Fvb3_7941491
Fvb3_7223702 Fvb3_7990976
Fvb3_5037154
54.2 Fvb3_9505578
54.3 Fvb3_10145665
54.5 Fvb3_9806835
54.7 Fvb3_5241600
55.8 Fvb3_19042100
56.0 Fvb3_18481616 Fvb3_19197246
56.3 Fvb3_19194607
56.8 Fvb6_38859243
57.3 Fvb3_9486820
57.6 Fvb3_10880225
66.1 Fvb3_11544857
0.0 Fvb3_17333423
49.8 Fvb3_26374412 Fvb3_25777525
51.9 Fvb3_23882593 Fvb3_23882567
Fvb3_26771022 Fvb3_24128105
54.0 Fvb3_24559862
56.2 Fvb3_24559832 Fvb3_25335147
58.3 Fvb3_24788838 Fvb3_24754876
60.4 Fvb3_25112620
67.1 Fvb3_16889020 Fvb6_5227774
69.2 Fvb7_14778881
72.5 Fvb3_17419495
75.7 Fvb3_17333428
77.8 Fvb3_17170376
84.5 Fvb3_20347861 Fvb3_14213548
86.6 Fvb0_724834
88.8 Fvb0_1390953
90.9 Fvb3_13871858
100.0 Fvb3_11507619
102.2 Fvb0_136158 Fvb0_179342
Fvb3_10880225
104.3 Fvb3_19197246 Fvb3_18481616
Fvb3_18782861 Fvb3_19021035
Fvb3_18418632 Fvb3_19042100
Fvb3_19194607
106.4 Fvb3_9505578 Fvb3_9806835
Fvb3_10145665
110.8 Fvb3_5241600
112.9 Fvb3_4816850 Fvb3_7990976
Fvb3_6546318 Fvb3_7941491
Fvb3_5037154 Fvb3_7577598
Fvb3_7941453 Fvb3_7080623
Fvb3_4896983 Fvb3_7387503
Fvb3_7223702 Fvb3_7074183
117.3 Fvb3_3012704
119.4 Fvb3_1598581 Fvb3_914827
Fvb3_4429546 Fvb3_2072457
Fvb3_2824564 Fvb3_1344637
Fvb3_4758693 Fvb3_322935
Fvb3_2282994 Fvb3_81452
Fvb3_4429526
122.1 Fvb3_2289465
130.6 Fvb6_38859243
135.0 Fvb3_9486820
144.9 Fvb3_11544857
Hapil_11
0.0 Fvb3_29685323 Fvb3_29969088
Fvb3_31084079 Fvb3_29472684
Fvb3_30087086 Fvb3_29150858
0.1 Fvb3_29375015 Fvb3_29560110
Fvb3_32069127
15.4 Fvb3_15956709
26.8 Fvb3_21510201
27.5 Fvb3_22002572
29.5 Fvb3_20341825
39.7 Fvb4_6114921
43.5 Fvb3_1692612
43.9 Fvb3_2150219
46.2 Fvb3_3044962
46.4 Fvb3_2825386
48.6 Fvb3_3623709
48.7 Fvb3_4168718
48.9 Fvb3_3623751
51.4 Fvb3_4601645
53.8 Fvb3_6138824 Fvb3_6138835
Fvb3_5037174
58.7 Fvb3_6954007
58.8 Fvb3_9486849 Fvb3_9065031
Fvb3_7203839
58.9 Fvb3_10050103
63.2 Fvb0_136119
63.3 Fvb3_11356966 Fvb0_135883
63.5 Fvb4_8243547 Fvb4_8408741
65.4 Fvb3_18706320
0.0 Fvb3_4607443
10.2 Fvb3_3623751 Fvb3_3623709
12.3 Fvb3_2825386
14.5 Fvb3_1692612 Fvb3_2150219
16.6 Fvb3_3044962
18.7 Fvb3_4168718
20.9 Fvb3_4601645
23.0 Fvb3_6138824 Fvb3_5037174
Fvb3_6138835
27.4 Fvb3_7203839 Fvb3_9065031
Fvb3_9486849 Fvb3_10050103
Fvb3_6954007
31.7 Fvb3_11356966 Fvb4_8243547
33.8 Fvb3_18706320
34.9 Fvb4_8408741
35.9 Fvb0_135883 Fvb0_136119
46.9 Fvb3_13762923
51.9 Fvb0_676842
58.6 Fvb3_20341825 Fvb3_20367865
Fvb3_21204746
60.7 Fvb3_21510201 Fvb3_22002572
87.7 Fvb3_30087086 Fvb3_29375015
Fvb3_32069127 Fvb3_31084079
Fvb3_29150858 Fvb3_29969088
Fvb3_29472684 Fvb3_29685323
Fvb3_29560110
102.3 Fvb3_15956709
118.1 Fvb4_6114921

Fvb_4

Redgauntlet_2

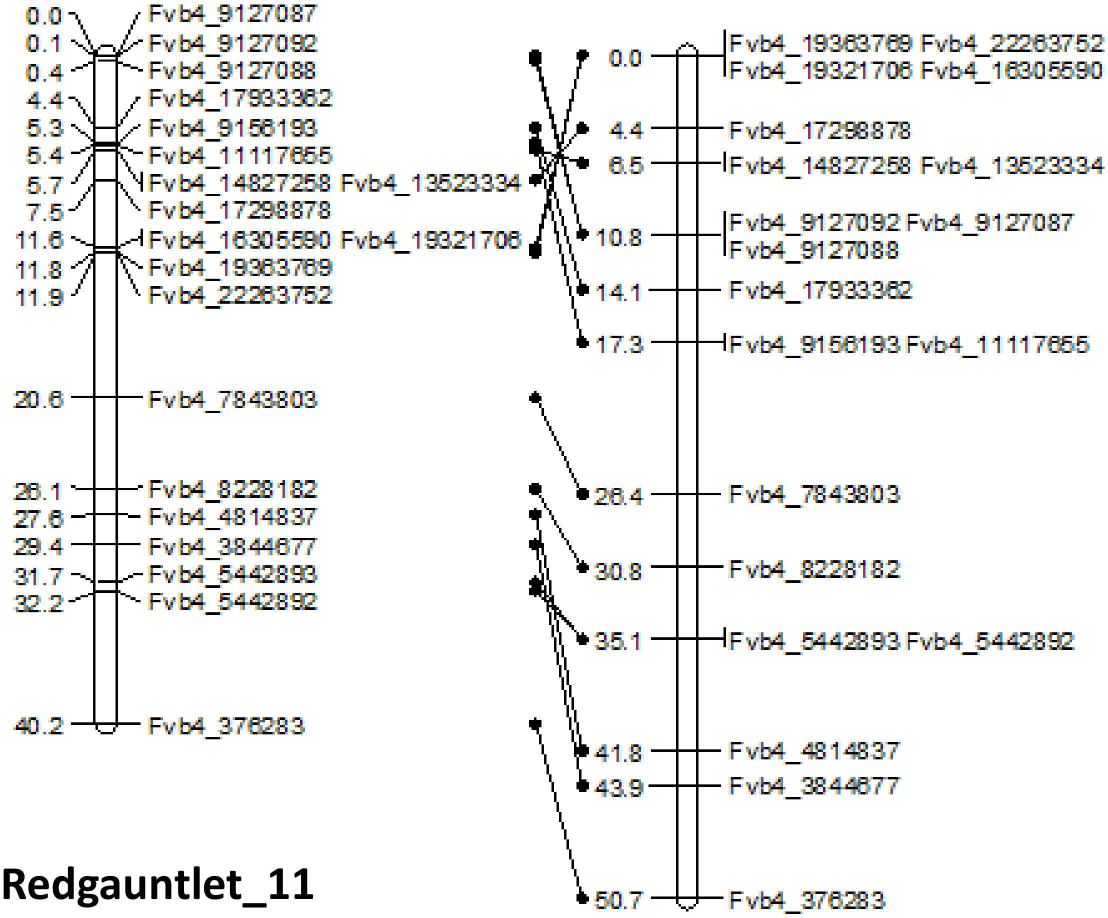

Redgauntlet_11

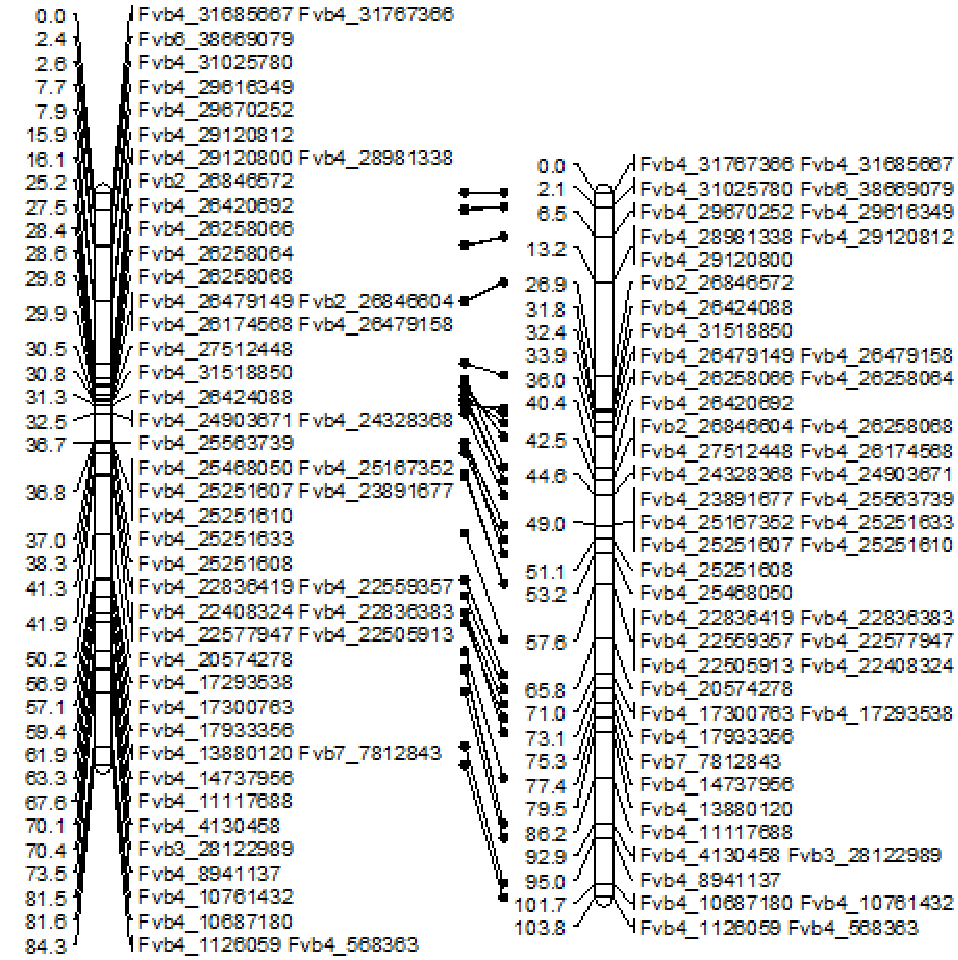

Fvb_4

Redgauntlet_27

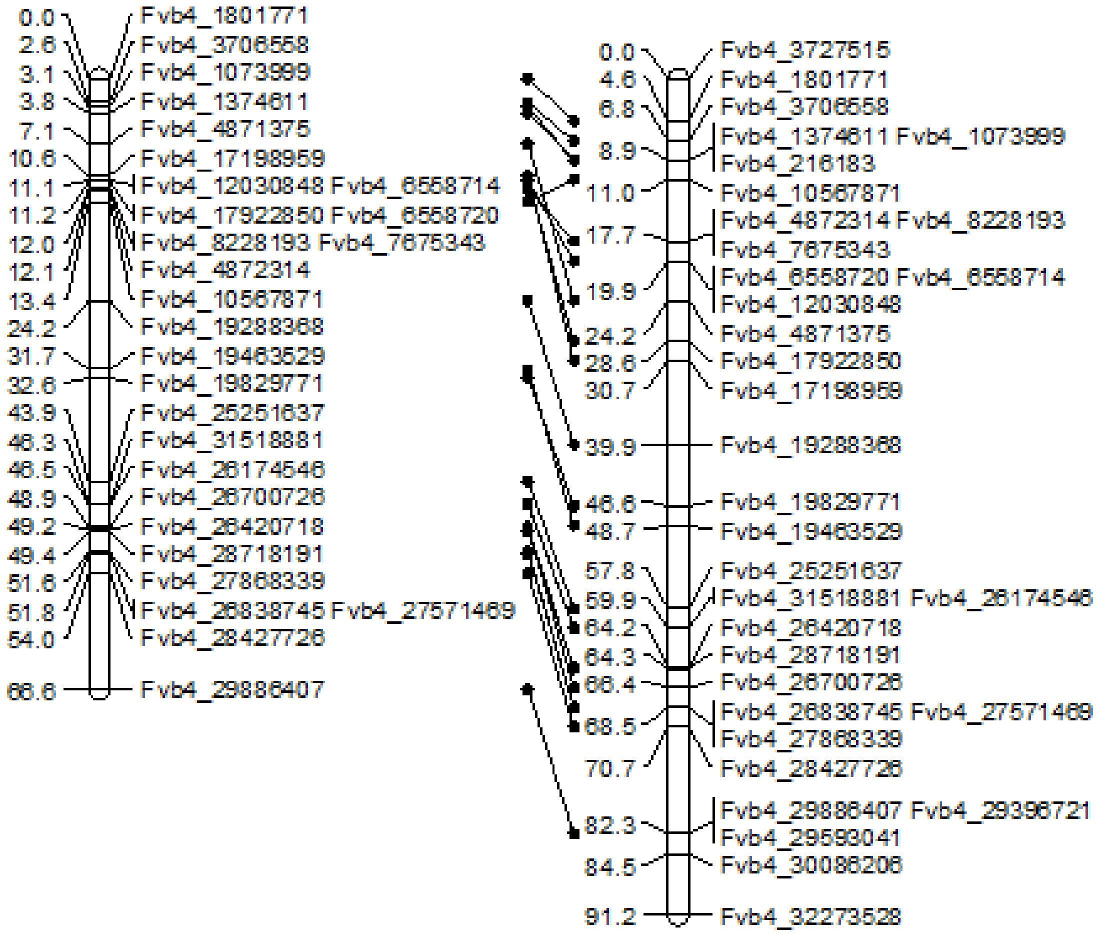

Redgauntlet_28

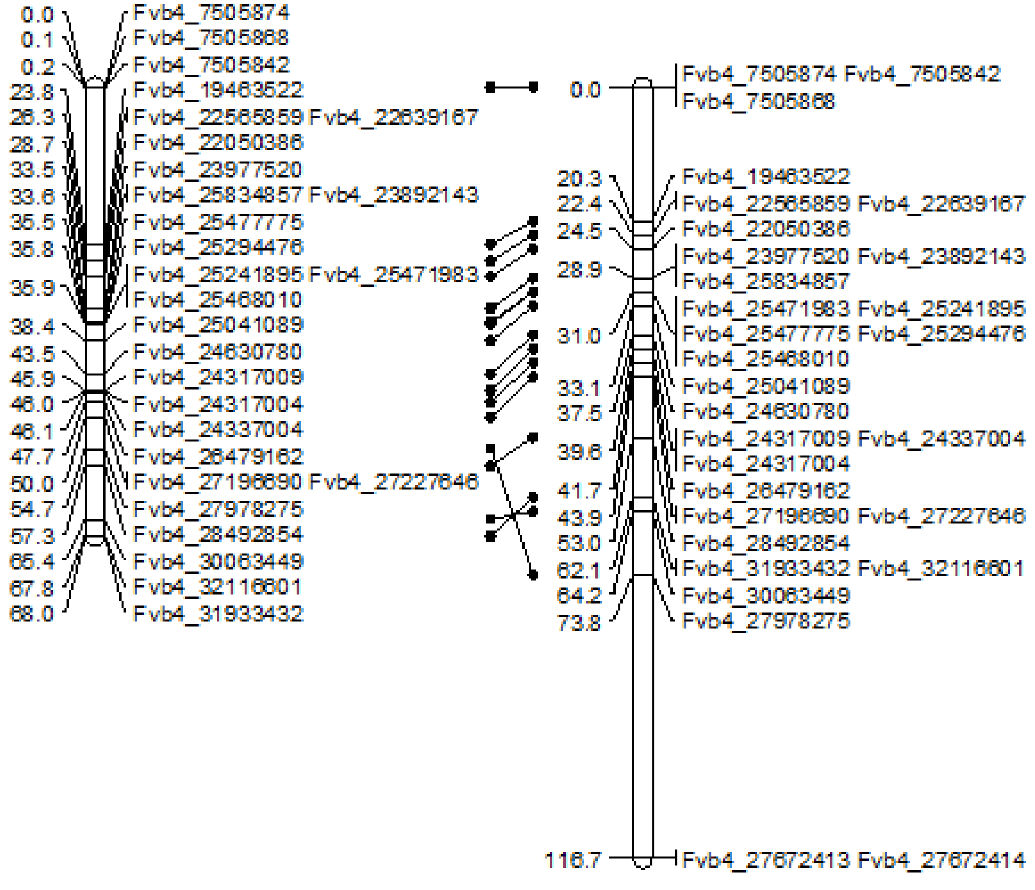

Fvb_4

Hapil_24

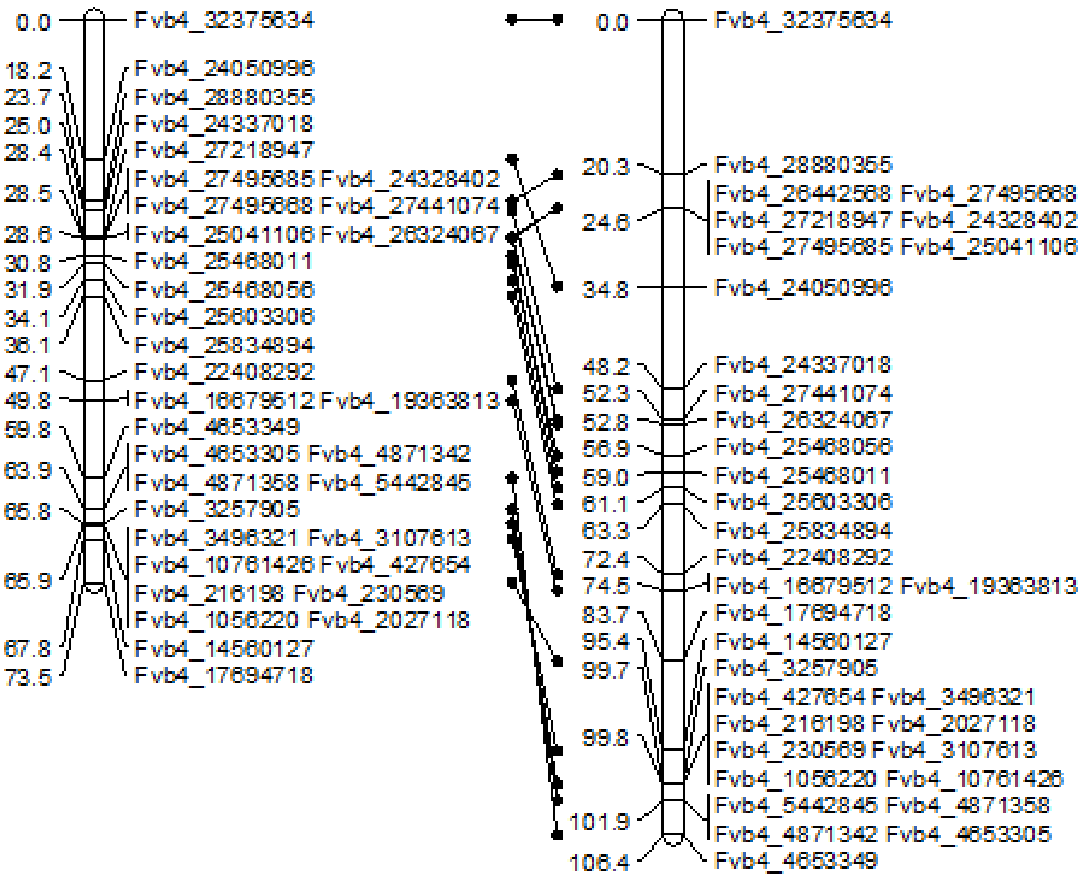

Hapil_30

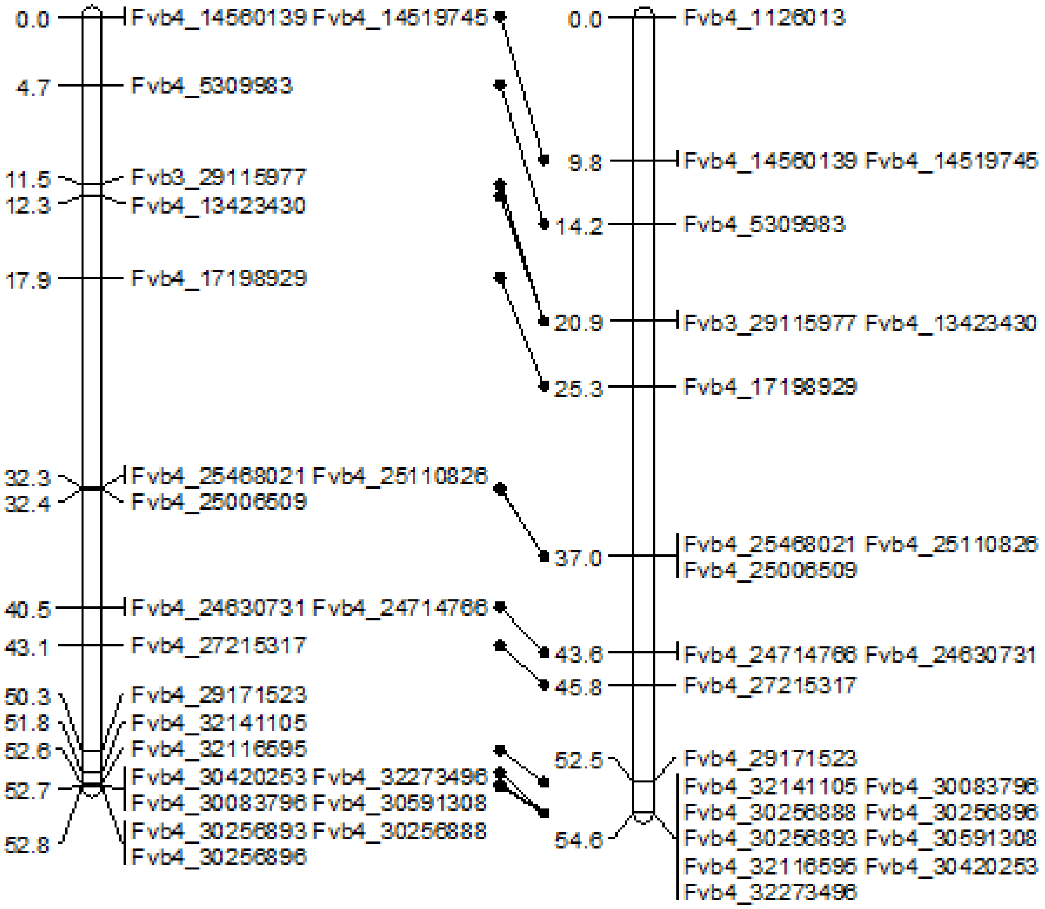

Fvb_4

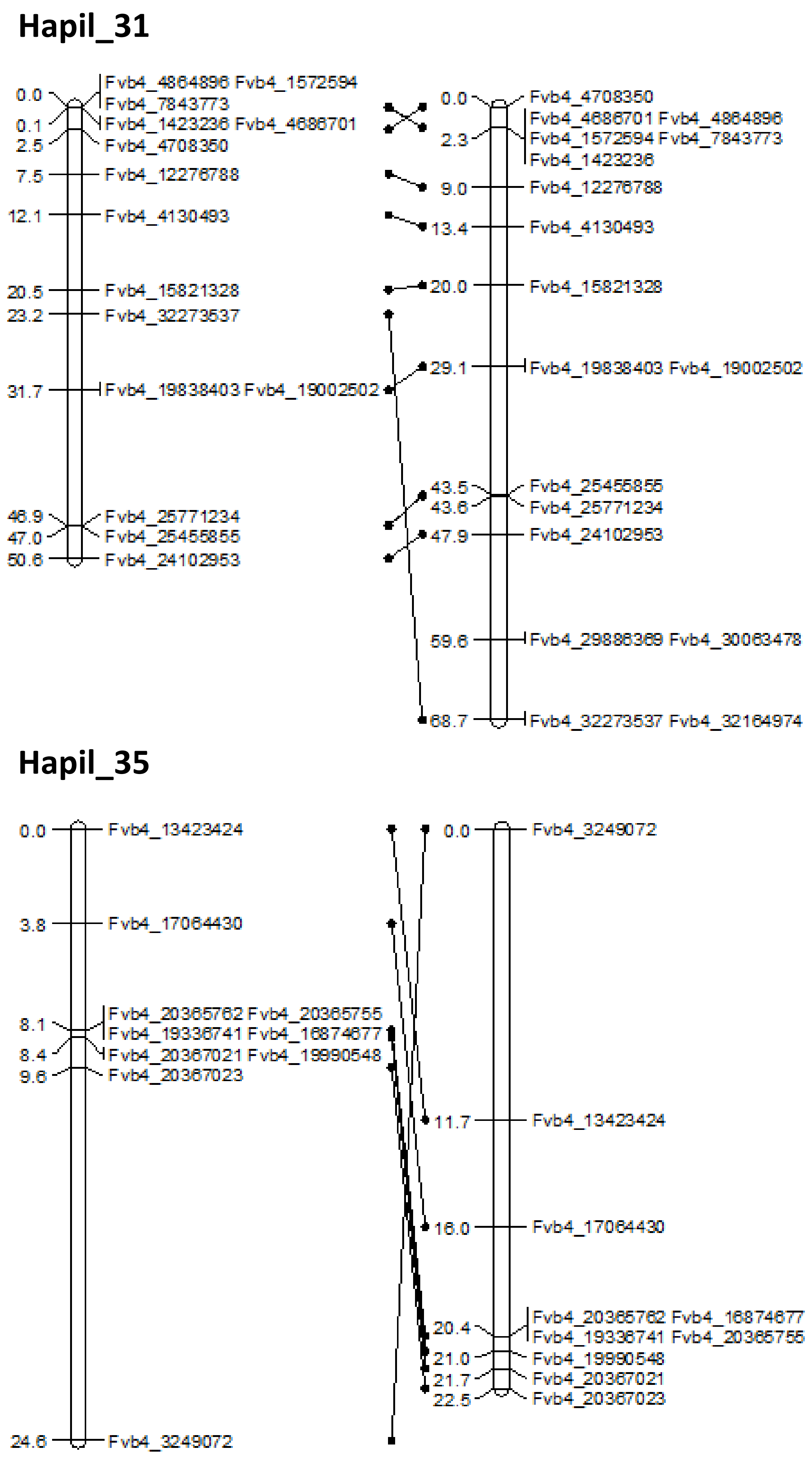

Hapil_31
0.0
0.1
2.5
7.5
12.1
20.5
23.2
31.7
46.9
47.0
50.6
Fvb4_4864896 Fvb4_1572594
Fvb4_7843773
Fvb4_1423236 Fvb4_4686701
Fvb4_4708350
Fvb4_12276788
Fvb4_4130493
Fvb4_15821328
Fvb4_32273537
Fvb4_19838403 Fvb4_19002502
Fvb4_25771234
Fvb4_25455855
Fvb4_24102953
0.0
2.3
9.0
13.4
20.0
29.1
43.5
43.6
47.9
59.6
68.7
Fvb4_4708350
Fvb4_4686701 Fvb4_4864896
Fvb4_1572594 Fvb4_7843773
Fvb4_1423236
Fvb4_12276788
Fvb4_4130493
Fvb4_15821328
Fvb4_19838403 Fvb4_19002502
Fvb4_25455855
Fvb4_25771234
Fvb4_24102953
Fvb4_29886369 Fvb4_30063478
Fvb4_32273537 Fvb4_32164974
Hapil_35
0.0
3.8
8.1
8.4
9.6
24.6
Fvb4_13423424
Fvb4_17064430
Fvb4_20365762 Fvb4_20365755
Fvb4_19336741 Fvb4_16874677
Fvb4_20367021 Fvb4_19990548
Fvb4_20367023
Fvb4_3249072
0.0
11.7
16.0
20.4
21.0
21.7
22.5
Fvb4_3249072
Fvb4_13423424
Fvb4_17064430
Fvb4_20365762 Fvb4_16874677
Fvb4_19336741 Fvb4_20365755
Fvb4_19990548
Fvb4_20367021
Fvb4_20367023

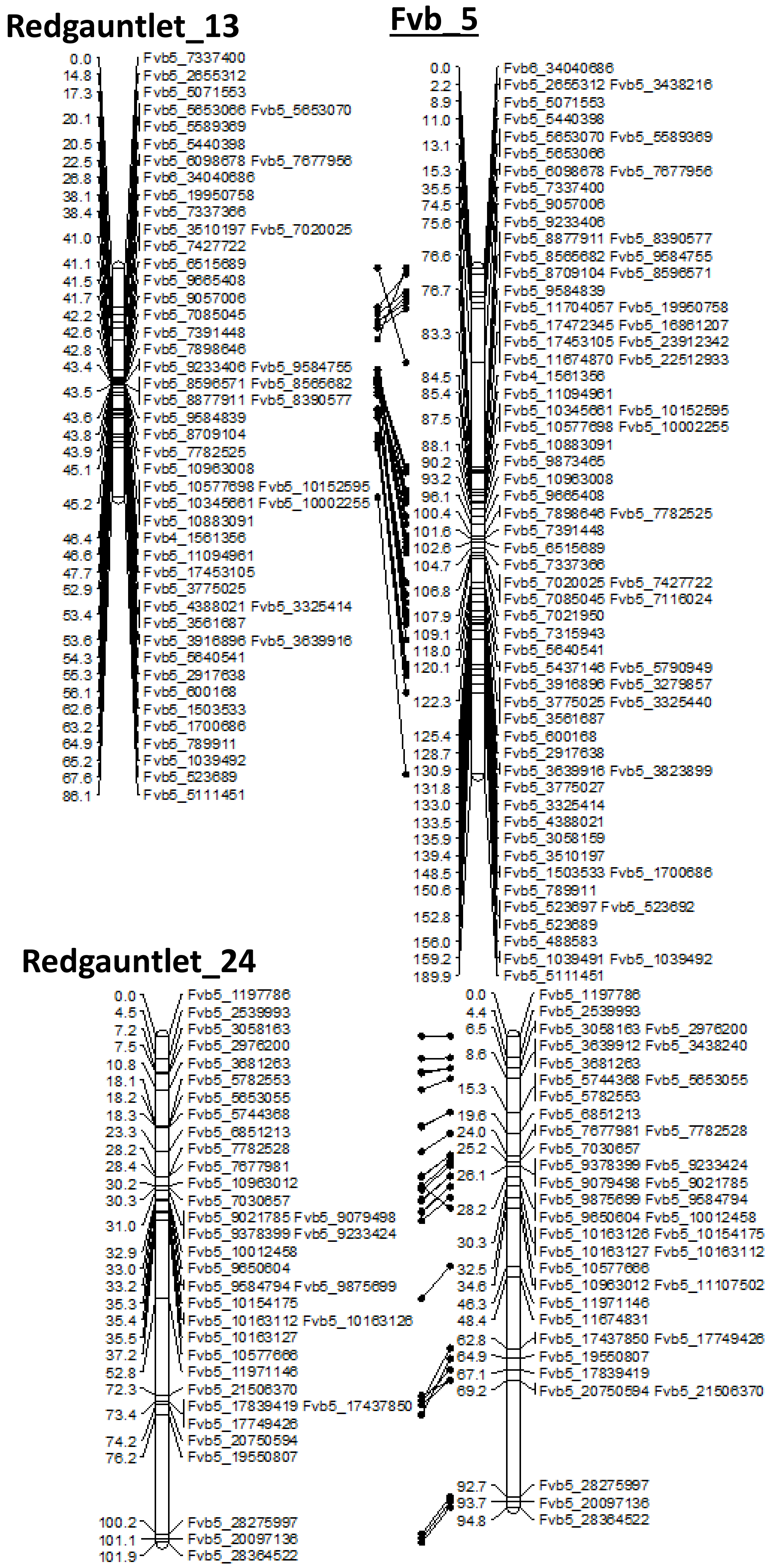

Redgauntlet_13
Fvb_5
0.0 Fvb5_7337400
14.8 Fvb5_2655312
17.3 Fvb5_5071553
20.1 Fvb5_5653066 Fvb5_5653070 Fvb5_5589369
20.5 Fvb5_5440398
22.5 Fvb5_6098678 Fvb5_7677956
26.8 Fvb6_34040686
38.1 Fvb5_19950758
38.4 Fvb5_7337366
41.0 Fvb5_3510197 Fvb5_7020025 Fvb5_7427722
41.1 Fvb5_6515689
41.5 Fvb5_9665408
41.7 Fvb5_9057006
42.2 Fvb5_7085045
42.6 Fvb5_7391448
42.8 Fvb5_7898646
43.4 Fvb5_9233406 Fvb5_9584755
43.5 Fvb5_8596571 Fvb5_8565682 Fvb5_8877911 Fvb5_8390577
43.6 Fvb5_9584839
43.8 Fvb5_8709104
43.9 Fvb5_7782525
45.1 Fvb5_10963008
45.2 Fvb5_10577698 Fvb5_10152595 Fvb5_10345661 Fvb5_10002255 Fvb5_10883091
46.4 Fvb4_1561356
46.6 Fvb5_11094961
47.7 Fvb5_17453105
52.9 Fvb5_3775025
53.4 Fvb5_4388021 Fvb5_3325414 Fvb5_3561687
53.6 Fvb5_3916896 Fvb5_3639916
54.3 Fvb5_5640541
55.3 Fvb5_2917638
58.1 Fvb5_600168
62.6 Fvb5_1503533
63.2 Fvb5_1700686
64.9 Fvb5_789911
65.2 Fvb5_1039492
67.6 Fvb5_523689
86.1 Fvb5_5111451
0.0 Fvb6_34040686
2.2 Fvb5_2655312 Fvb5_3438216
8.9 Fvb5_5071553
11.0 Fvb5_5440398
13.1 Fvb5_5653070 Fvb5_5589369 Fvb5_5653066
15.3 Fvb5_6098678 Fvb5_7677956
35.5 Fvb5_7337400
74.5 Fvb5_9057006
75.6 Fvb5_9233406
76.6 Fvb5_8877911 Fvb5_8390577 Fvb5_8565682 Fvb5_9584755 Fvb5_8709104 Fvb5_8596571
76.7 Fvb5_9584839 Fvb5_11704057 Fvb5_19950758
83.3 Fvb5_17472345 Fvb5_16861207 Fvb5_17453105 Fvb5_23912342 Fvb5_11674870 Fvb5_22512933
84.5 Fvb4_1561356
85.4 Fvb5_11094961
87.5 Fvb5_10345661 Fvb5_10152595 Fvb5_10577698 Fvb5_10002255
88.1 Fvb5_10883091
90.2 Fvb5_9873465
93.2 Fvb5_10963008
96.1 Fvb5_9665408
100.4 Fvb5_7898646 Fvb5_7782525
101.6 Fvb5_7391448
102.6 Fvb5_6515689
104.7 Fvb5_7337366
106.8 Fvb5_7020025 Fvb5_7427722 Fvb5_7085045 Fvb5_7116024
107.9 Fvb5_7021950
109.1 Fvb5_7315943
118.0 Fvb5_5640541
120.1 Fvb5_5437146 Fvb5_5790949 Fvb5_3916896 Fvb5_3279857
122.3 Fvb5_3775025 Fvb5_3325440 Fvb5_3561687
125.4 Fvb5_600168
128.7 Fvb5_2917638
130.9 Fvb5_3639916 Fvb5_3823899
131.8 Fvb5_3775027
133.0 Fvb5_3325414
133.5 Fvb5_4388021
135.9 Fvb5_3058159
139.4 Fvb5_3510197
148.5 Fvb5_1503533 Fvb5_1700686
150.6 Fvb5_789911
152.8 Fvb5_523697 Fvb5_523692 Fvb5_523689
156.0 Fvb5_488583
159.2 Fvb5_1039491 Fvb5_1039492
189.9 Fvb5_5111451
Redgauntlet_24
0.0 Fvb5_1197786
4.5 Fvb5_2539993
7.2 Fvb5_3058163
7.5 Fvb5_2976200
10.8 Fvb5_3681263
18.1 Fvb5_5782553
18.2 Fvb5_5653055
18.3 Fvb5_5744368
23.3 Fvb5_6851213
28.2 Fvb5_7782528
28.4 Fvb5_7677981
30.2 Fvb5_10963012
30.3 Fvb5_7030657
31.0 Fvb5_9021785 Fvb5_9079498 Fvb5_9378399 Fvb5_9233424
32.9 Fvb5_10012458
33.0 Fvb5_9650604
33.2 Fvb5_9584794 Fvb5_9875699
35.3 Fvb5_10154175
35.4 Fvb5_10163112 Fvb5_10163126
35.5 Fvb5_10163127
37.2 Fvb5_10577666
52.8 Fvb5_11971146
72.3 Fvb5_21506370
73.4 Fvb5_17839419 Fvb5_17437850 Fvb5_17749426
74.2 Fvb5_20750594
76.2 Fvb5_19550807
100.2 Fvb5_28275997
101.1 Fvb5_20097136
101.9 Fvb5_28364522
0.0 Fvb5_1197786
4.4 Fvb5_2539993
6.5 Fvb5_3058163 Fvb5_2976200
8.6 Fvb5_3639912 Fvb5_3438240 Fvb5_3681263
15.3 Fvb5_5744368 Fvb5_5653055 Fvb5_5782553
19.6 Fvb5_6851213
24.0 Fvb5_7677981 Fvb5_7782528
25.2 Fvb5_7030657
26.1 Fvb5_9378399 Fvb5_9233424 Fvb5_9079498 Fvb5_9021785
28.2 Fvb5_9875699 Fvb5_9584794 Fvb5_9650604 Fvb5_10012458
30.3 Fvb5_10163126 Fvb5_10154175 Fvb5_10163127 Fvb5_10163112
32.5 Fvb5_10577666
34.6 Fvb5_10963012 Fvb5_11107502
46.3 Fvb5_11971146
48.4 Fvb5_11674831
62.8 Fvb5_17437850 Fvb5_17749426
64.9 Fvb5_19550807
67.1 Fvb5_17839419
69.2 Fvb5_20750594 Fvb5_21506370
92.7 Fvb5_28275997
93.7 Fvb5_20097136
94.8 Fvb5_28364522

Fvb_5

Redgauntlet_25

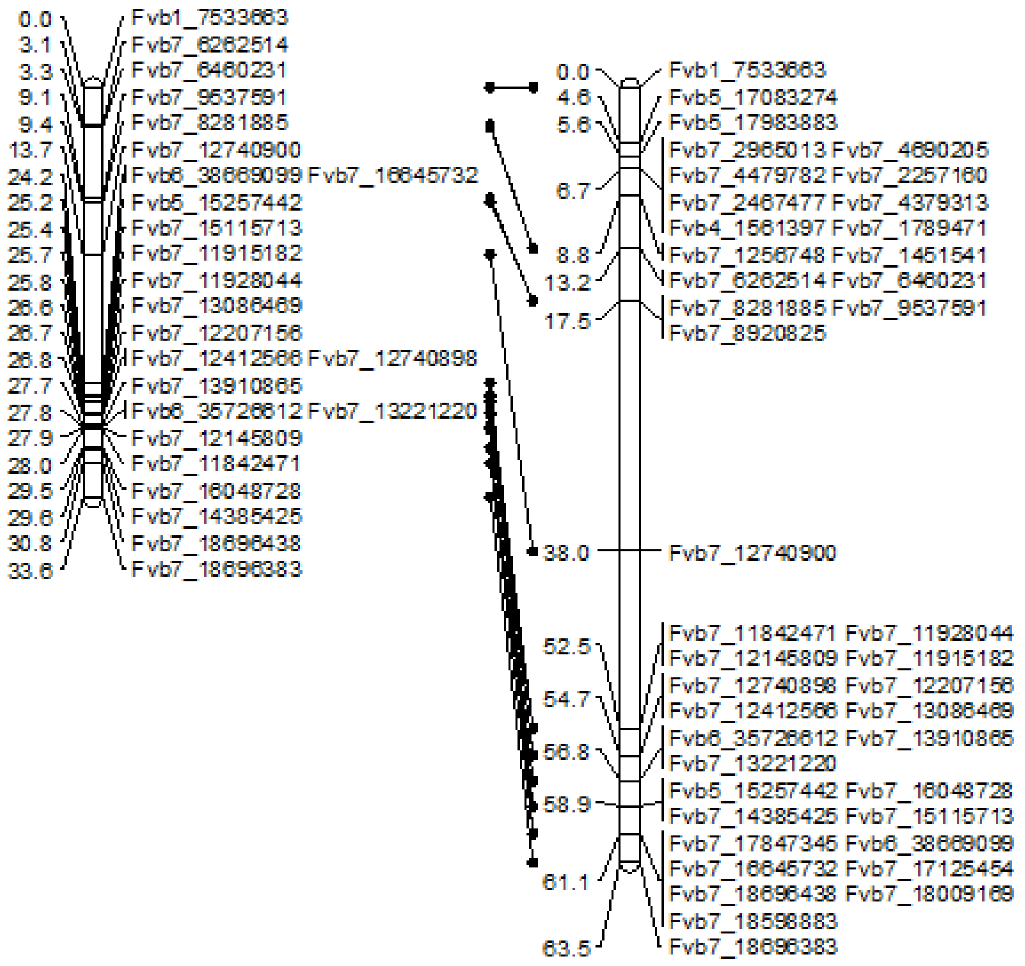

Redgauntlet_33

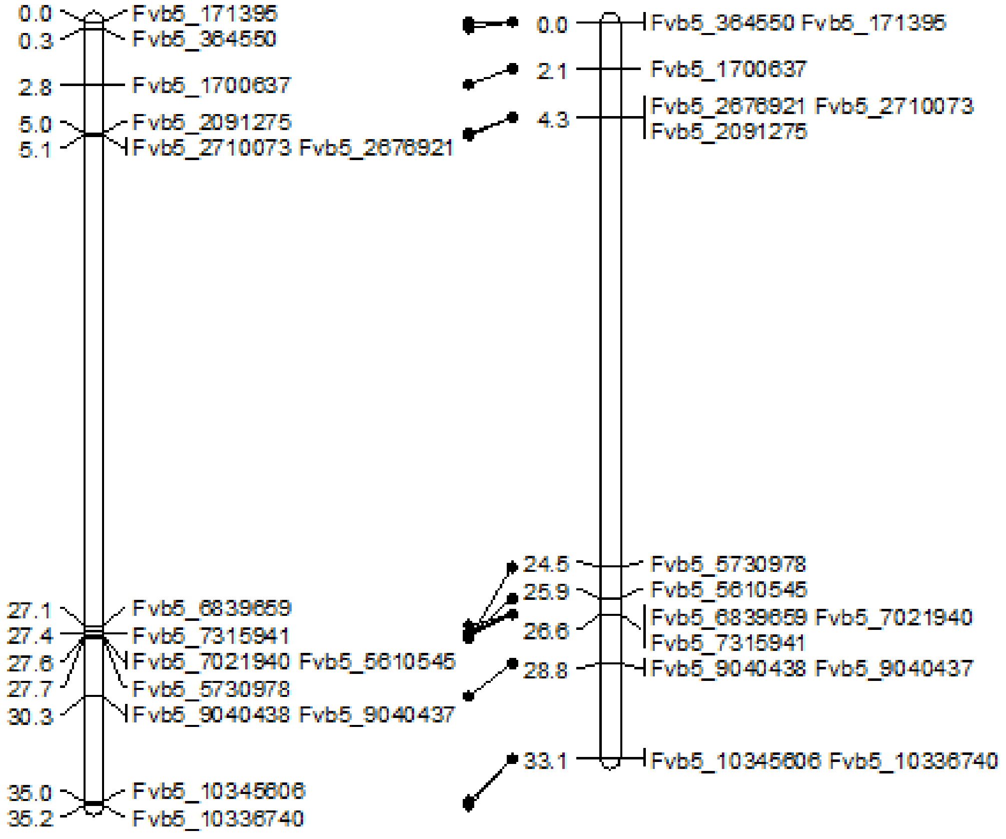

Fvb_5

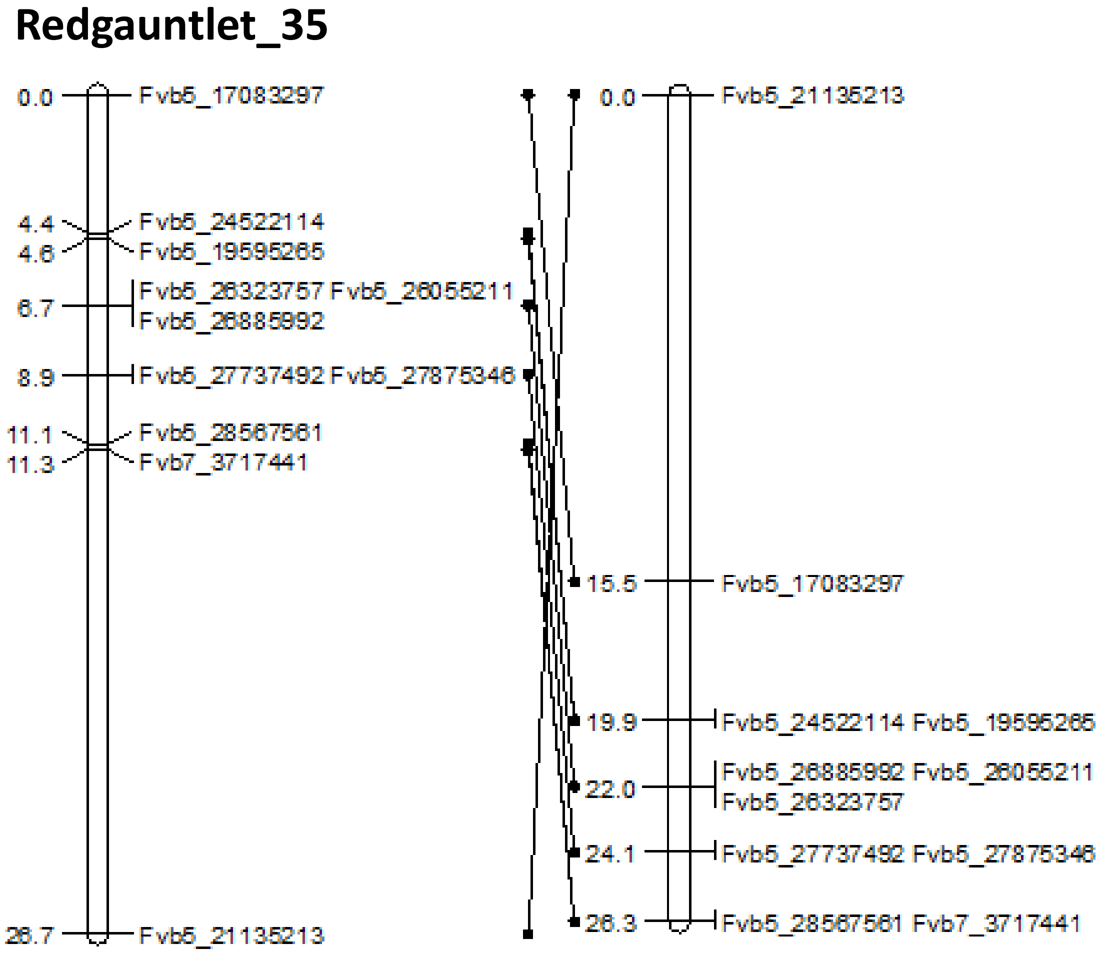
Redgauntlet_35
0.0
Fvb5_17083297
4.4
4.6
Fvb5_24522114
Fvb5_19595265
6.7
Fvb5_26323757 Fvb5_26055211
Fvb5_26885992
8.9
Fvb5_27737492 Fvb5_27875346
11.1
11.3
Fvb5_28567561
Fvb7_3717441
26.7
Fvb5_21135213
0.0
Fvb5_21135213
15.5
Fvb5_17083297
19.9
Fvb5_24522114 Fvb5_19595265
22.0
Fvb5_26885992 Fvb5_26055211
Fvb5_26323757
24.1
Fvb5_27737492 Fvb5_27875346
26.3
Fvb5_28567561 Fvb7_3717441

Fvb_5

Hapil_1

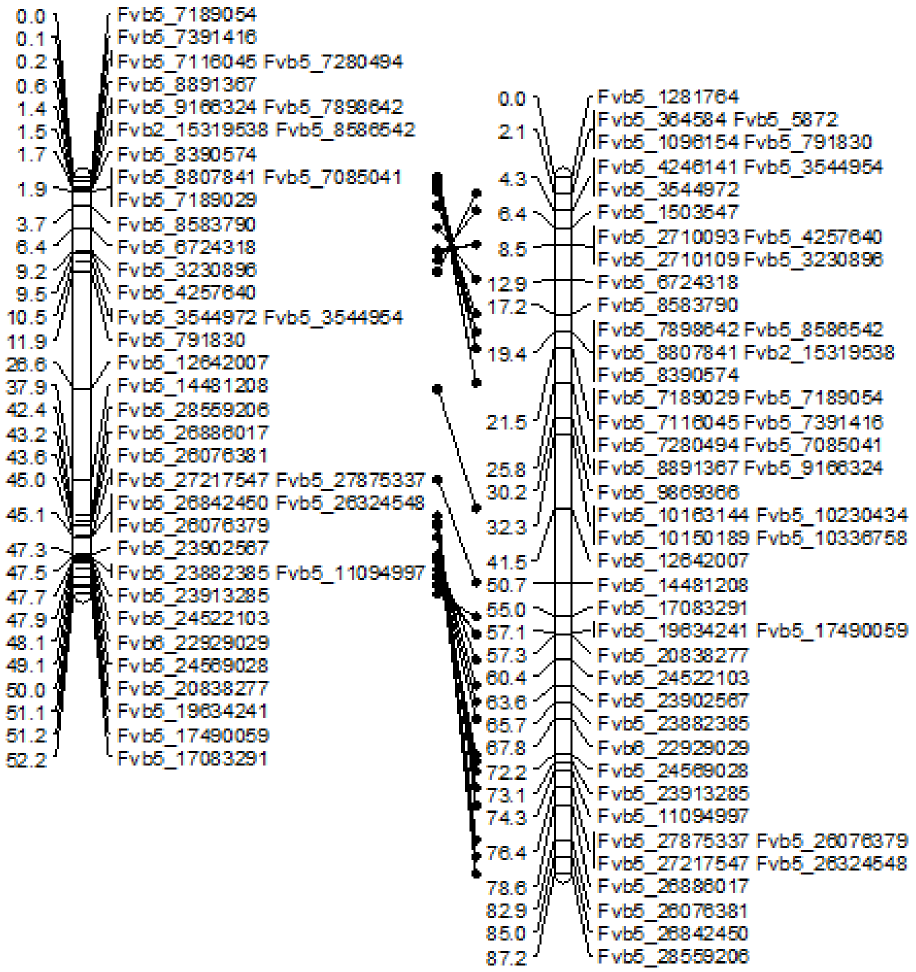

Hapil_6

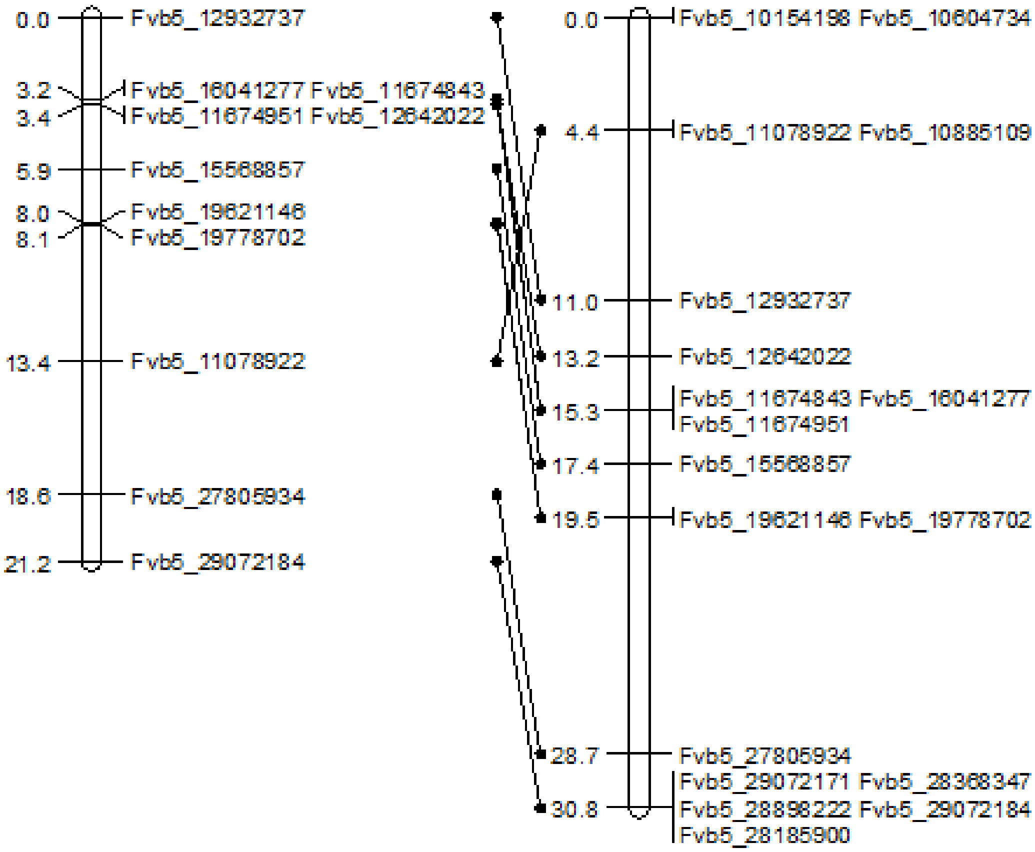

Fvb_5

Hapil_20

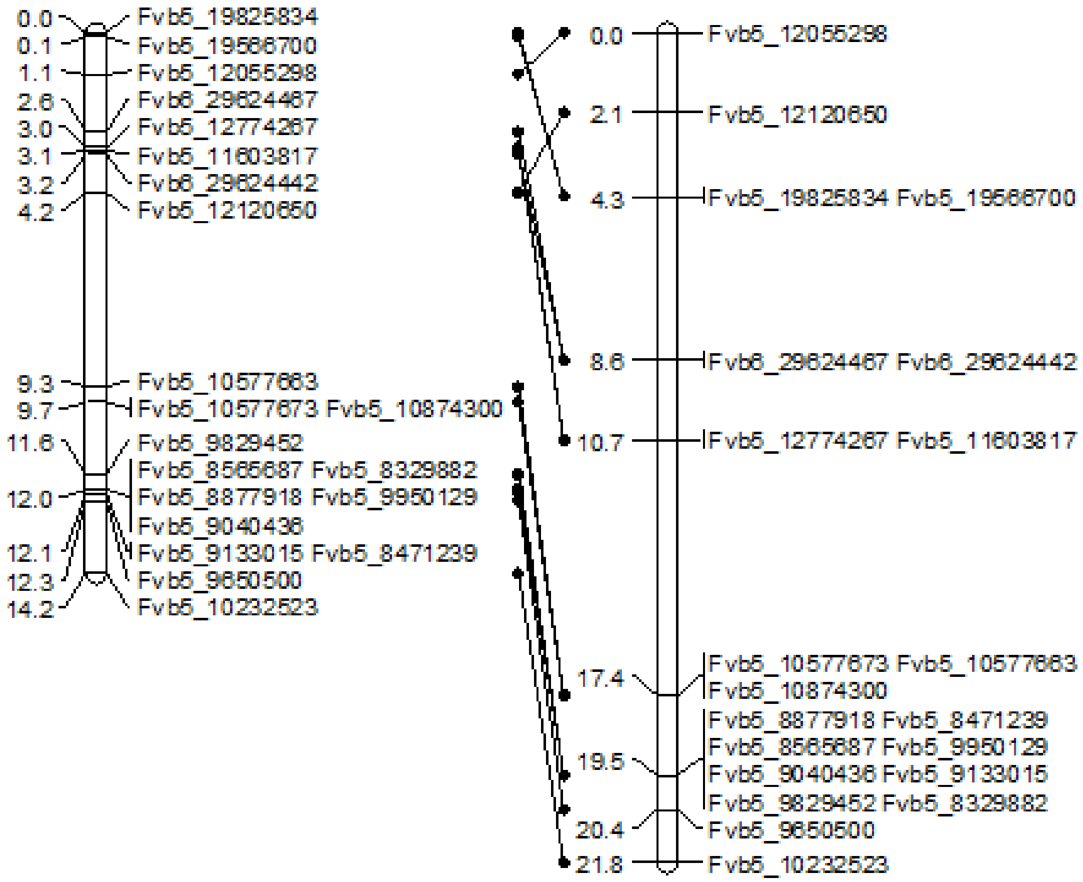

Hapil_28

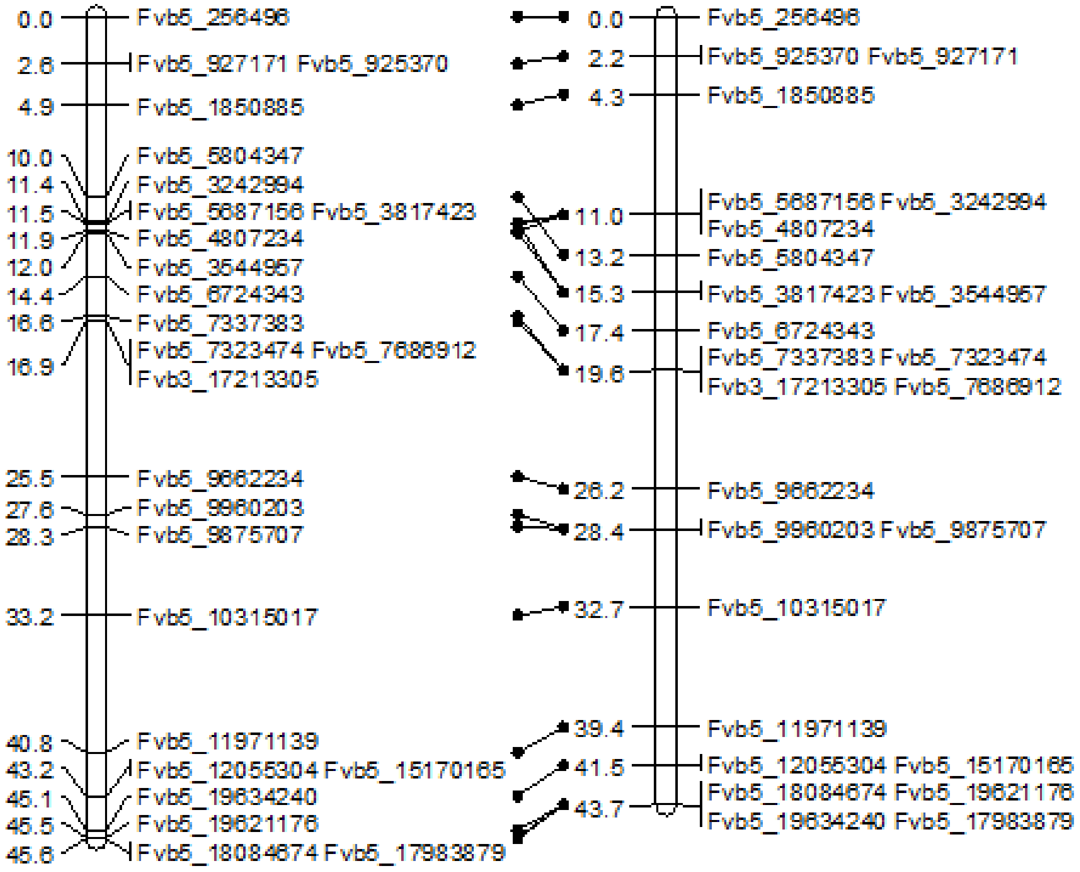

Fvb_6

Redgauntlet_5

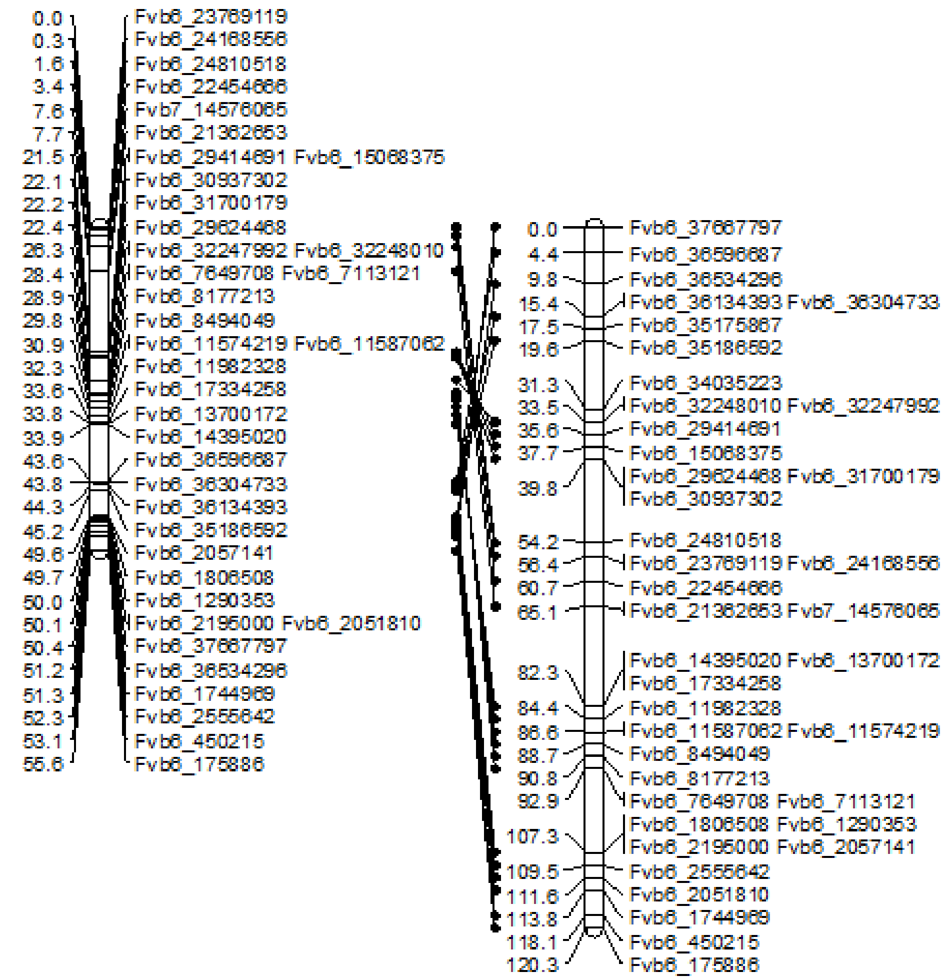

Redgauntlet_6

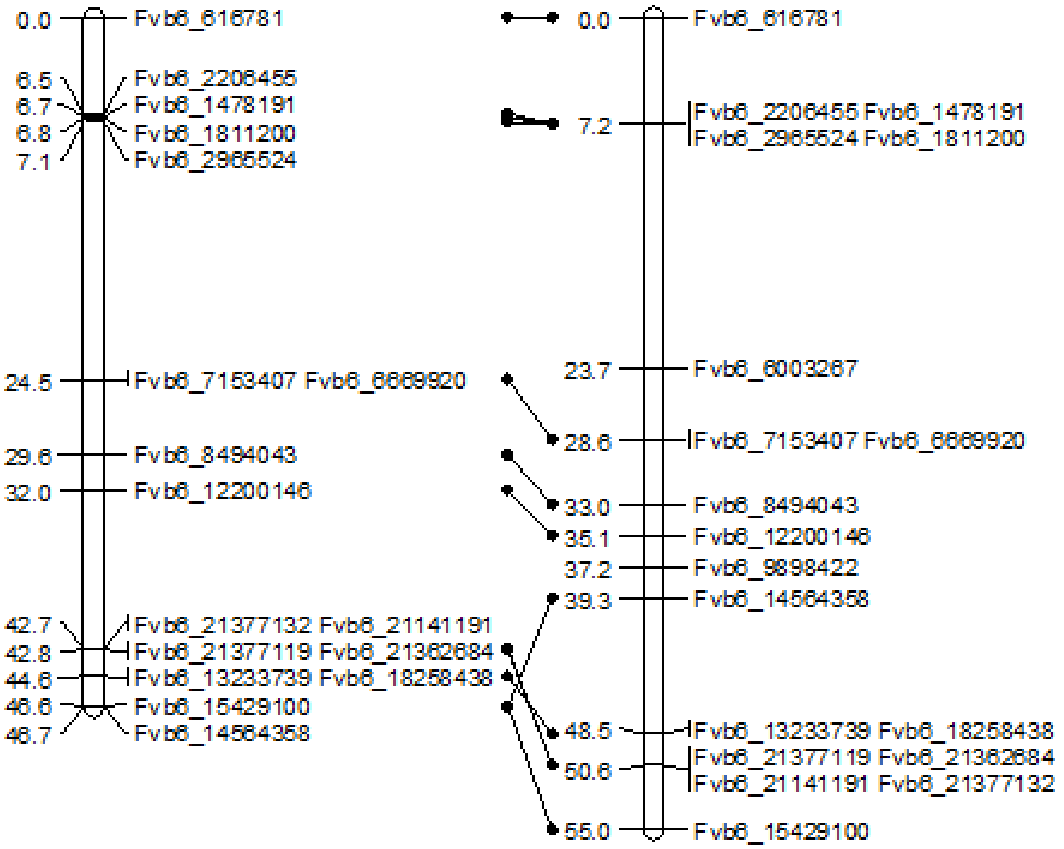

Fvb_6

Redgauntlet_31

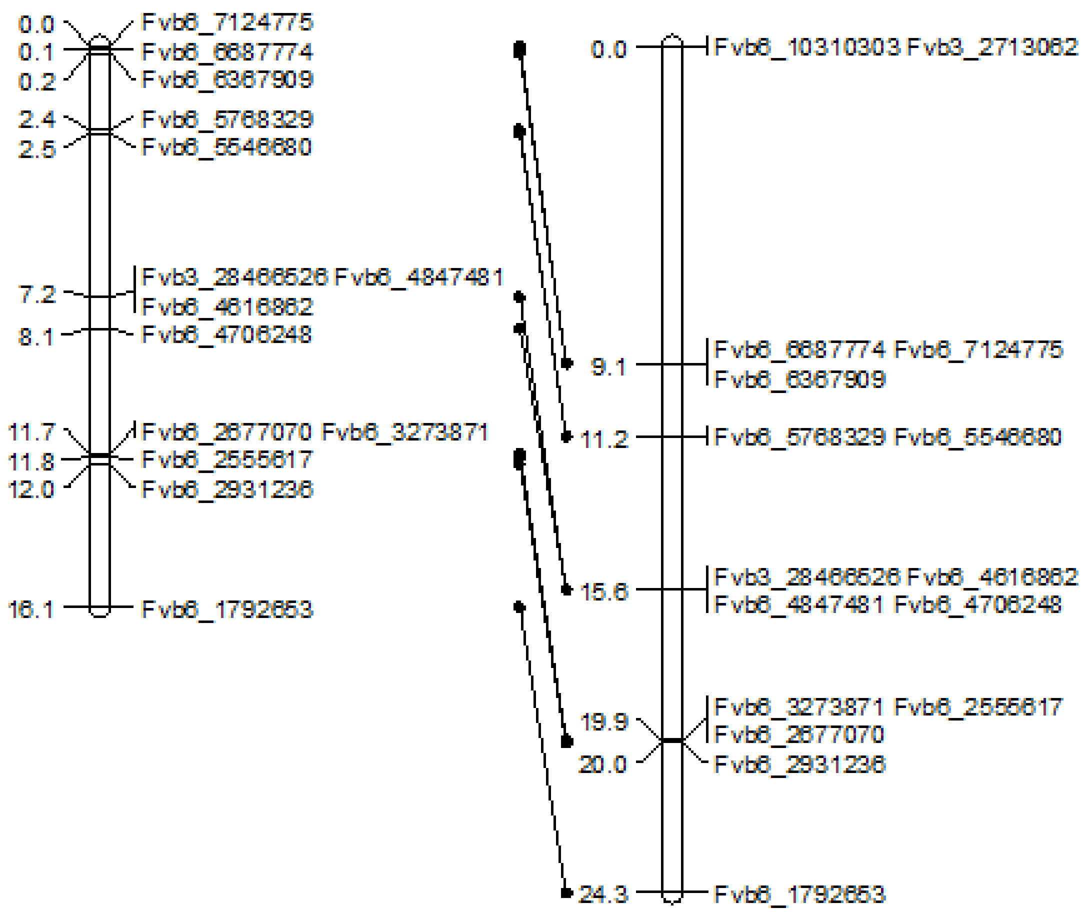

Redgauntlet_40

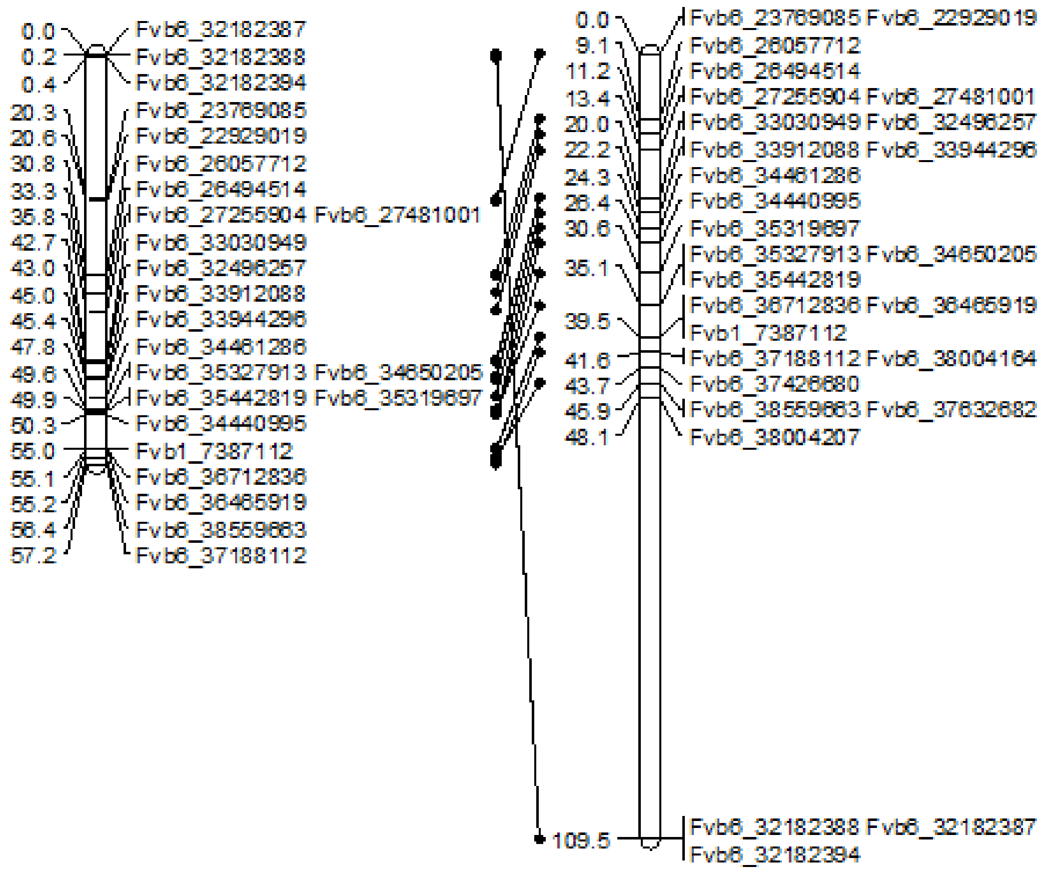

Fvb_6

Hapil_2

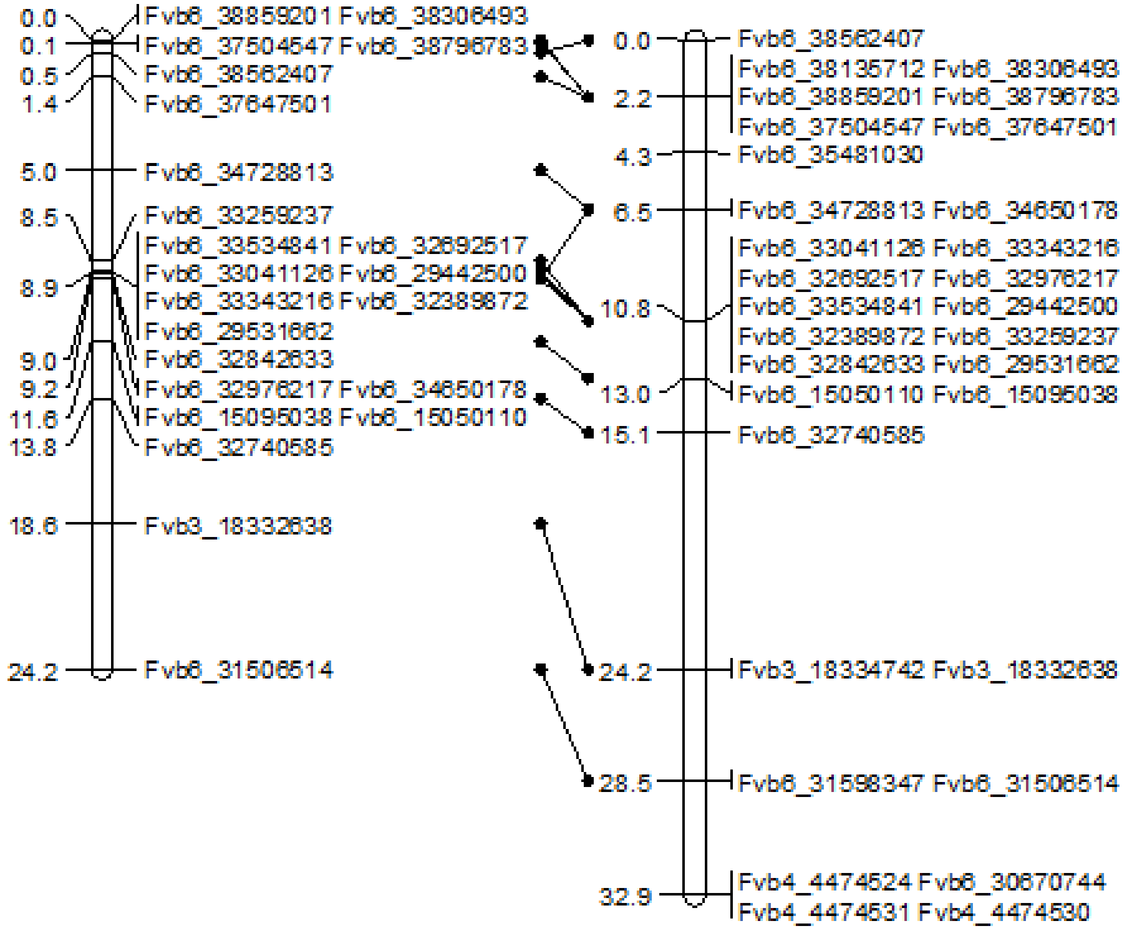

Hapil_3

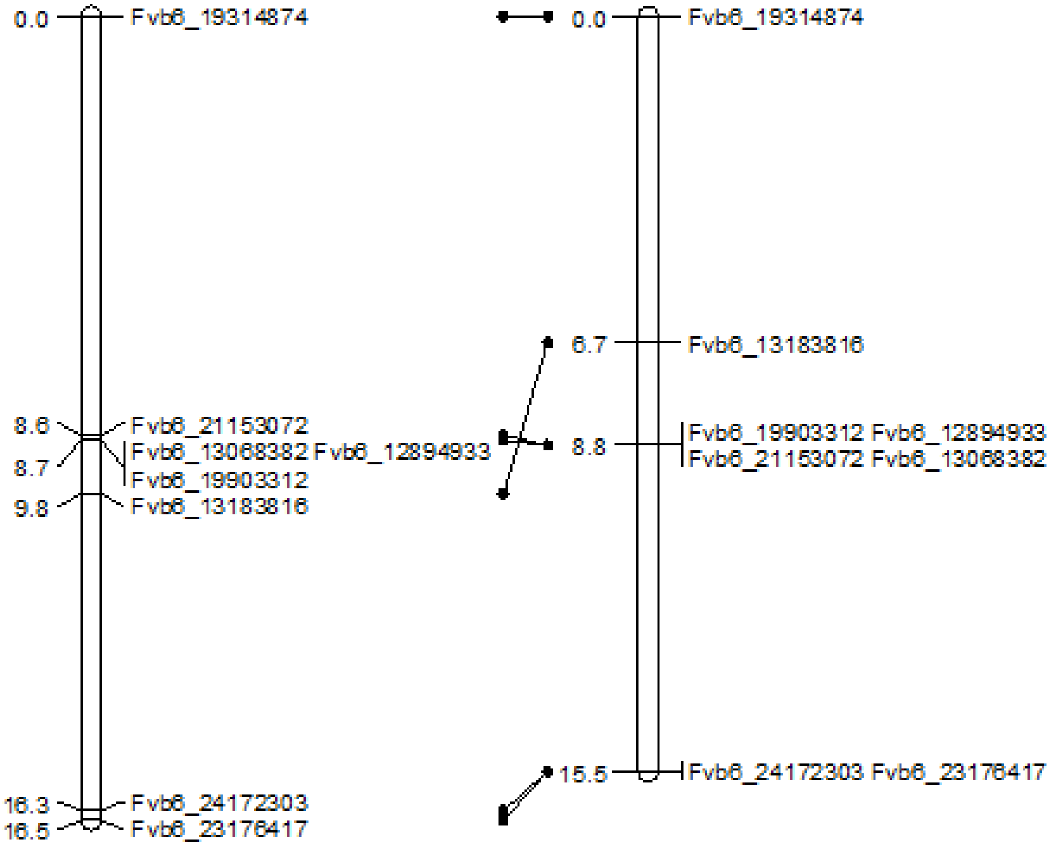

Fvb_6

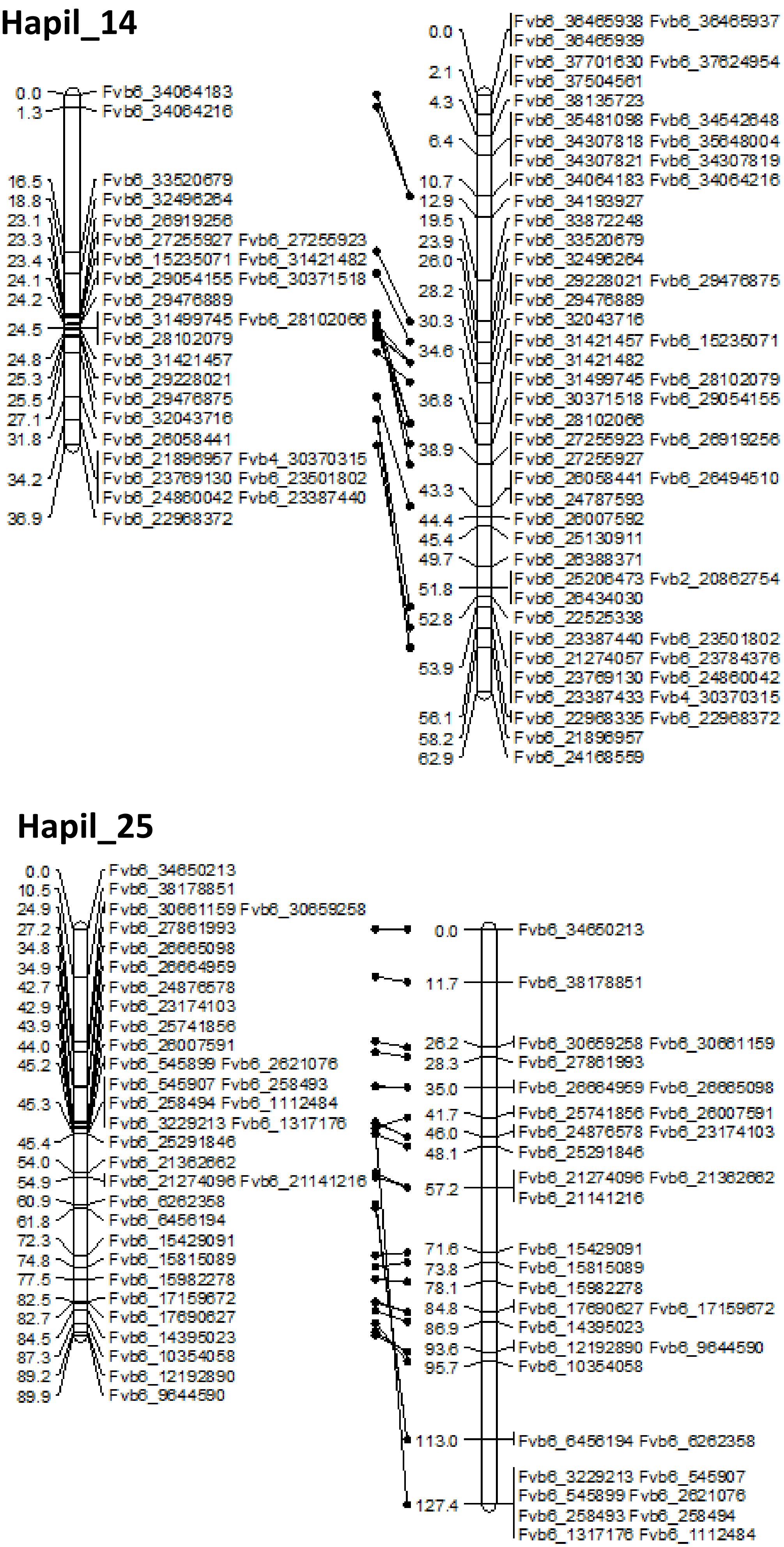

Hapil_14
0.0 Fvb6_34064183
1.3 Fvb6_34064216
16.5 Fvb6_33520679
18.8 Fvb6_32496264
23.1 Fvb6_26919256
23.3 Fvb6_27255927 Fvb6_27255923
23.4 Fvb6_15235071 Fvb6_31421482
24.1 Fvb6_29054155 Fvb6_30371518
24.2 Fvb6_29476889
24.5 Fvb6_31499745 Fvb6_28102066
Fvb6_28102079
24.8 Fvb6_31421457
25.3 Fvb6_29228021
25.5 Fvb6_29476875
27.1 Fvb6_32043716
31.8 Fvb6_26058441
Fvb6_21896957 Fvb4_30370315
34.2 Fvb6_23769130 Fvb6_23501802
Fvb6_24860042 Fvb6_23387440
36.9 Fvb6_22968372
0.0 Fvb6_38465938 Fvb6_38465937
Fvb6_38465939
2.1 Fvb6_37701630 Fvb6_37824954
Fvb6_37504561
4.3 Fvb6_38135723
Fvb6_35481098 Fvb6_34542648
6.4 Fvb6_34307818 Fvb6_35648004
Fvb6_34307821 Fvb6_34307819
10.7 Fvb6_34064183 Fvb6_34064216
12.9 Fvb6_34193927
19.5 Fvb6_33872248
23.9 Fvb6_33520679
26.0 Fvb6_32496264
28.2 Fvb6_29228021 Fvb6_29476875
Fvb6_29476889
30.3 Fvb6_32043716
34.6 Fvb6_31421457 Fvb6_15235071
Fvb6_31421482
36.8 Fvb6_31499745 Fvb6_28102079
Fvb6_30371518 Fvb6_29054155
Fvb6_28102066
38.9 Fvb6_27255923 Fvb6_26919256
Fvb6_27255927
43.3 Fvb6_26058441 Fvb6_26494510
Fvb6_24787593
44.4 Fvb6_26007592
45.4 Fvb6_25130911
49.7 Fvb6_26388371
51.8 Fvb6_25206473 Fvb2_20862754
Fvb6_26434030
52.8 Fvb6_22525338
Fvb6_23387440 Fvb6_23501802
53.9 Fvb6_21274057 Fvb6_23784376
Fvb6_23769130 Fvb6_24860042
Fvb6_23387433 Fvb4_30370315
56.1 Fvb6_22968335 Fvb6_22968372
58.2 Fvb6_21896957
62.9 Fvb6_24168559
Hapil_25
0.0 Fvb6_34650213
10.5 Fvb6_38178851
24.9 Fvb6_30661159 Fvb6_30659258
27.2 Fvb6_27861993
34.8 Fvb6_26665098
34.9 Fvb6_26664959
42.7 Fvb6_24876578
42.9 Fvb6_23174103
43.9 Fvb6_25741856
44.0 Fvb6_26007591
45.2 Fvb6_545899 Fvb6_2621076
Fvb6_545907 Fvb6_258493
45.3 Fvb6_258494 Fvb6_1112484
Fvb6_3229213 Fvb6_1317176
45.4 Fvb6_25291846
54.0 Fvb6_21362662
54.9 Fvb6_21274096 Fvb6_21141216
60.9 Fvb6_6262358
61.8 Fvb6_6456194
72.3 Fvb6_15429091
74.8 Fvb6_15815089
77.5 Fvb6_15982278
82.5 Fvb6_17159672
82.7 Fvb6_17690627
84.5 Fvb6_14395023
87.3 Fvb6_10354058
89.2 Fvb6_12192890
89.9 Fvb6_9644590
0.0 Fvb6_34650213
11.7 Fvb6_38178851
26.2 Fvb6_30659258 Fvb6_30661159
28.3 Fvb6_27861993
35.0 Fvb6_26664959 Fvb6_26665098
41.7 Fvb6_25741856 Fvb6_26007591
46.0 Fvb6_24876578 Fvb6_23174103
48.1 Fvb6_25291846
57.2 Fvb6_21274096 Fvb6_21362662
Fvb6_21141216
71.6 Fvb6_15429091
73.8 Fvb6_15815089
78.1 Fvb6_15982278
84.8 Fvb6_17690627 Fvb6_17159672
86.9 Fvb6_14395023
93.6 Fvb6_12192890 Fvb6_9644590
95.7 Fvb6_10354058
113.0 Fvb6_6456194 Fvb6_6262358
Fvb6_3229213 Fvb6_545907
127.4 Fvb6_545899 Fvb6_2621076
Fvb6_258493 Fvb6_258494
Fvb6_1317176 Fvb6_1112484

Fvb_6

Hapil_36

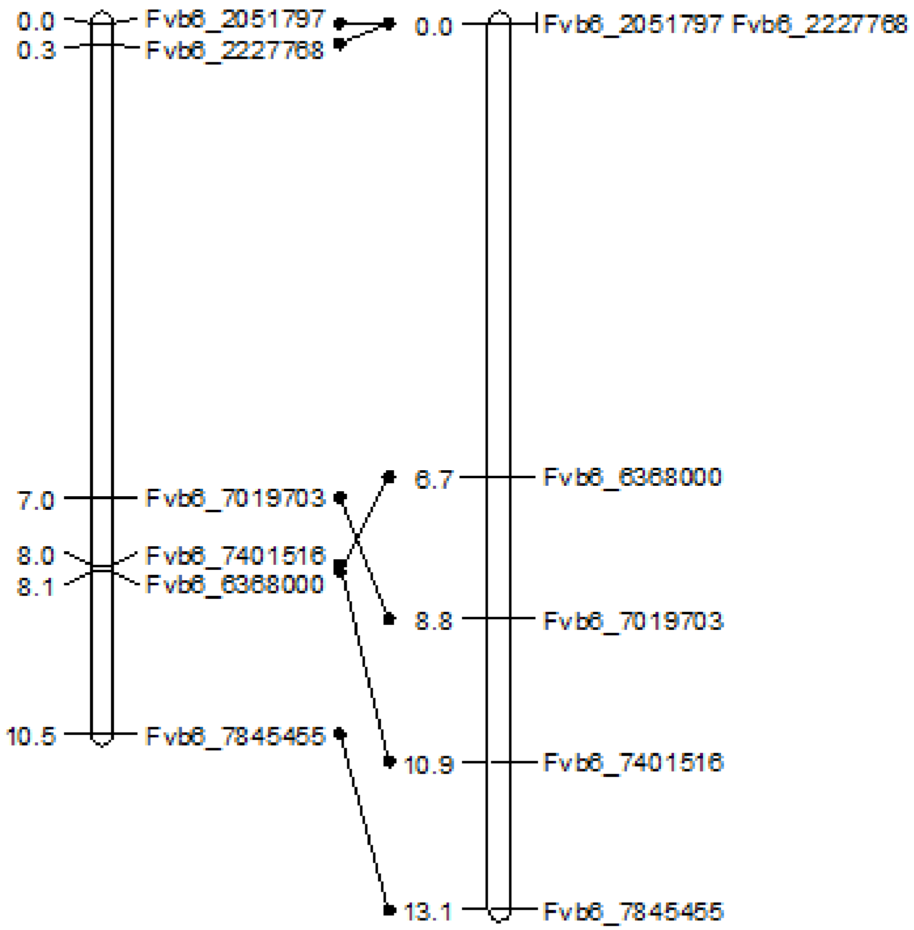

Fvb_7

Redgauntlet_7

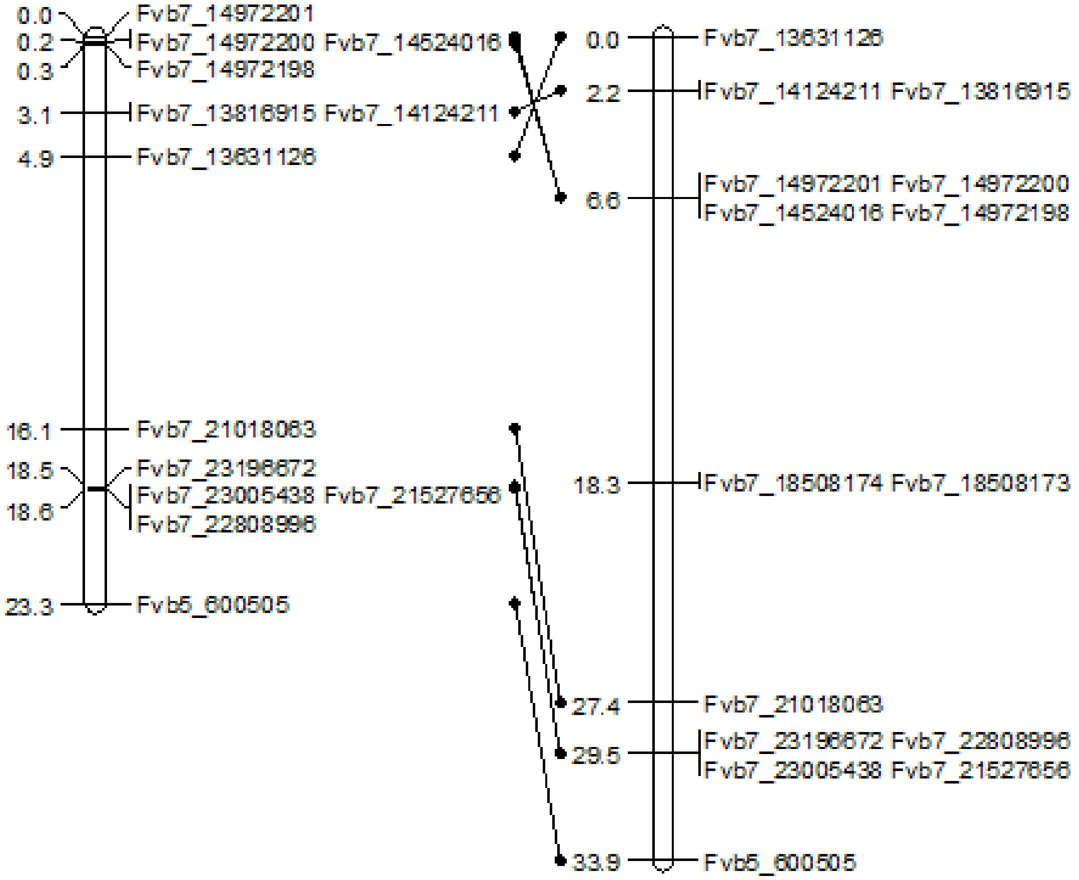

Redgauntlet_16

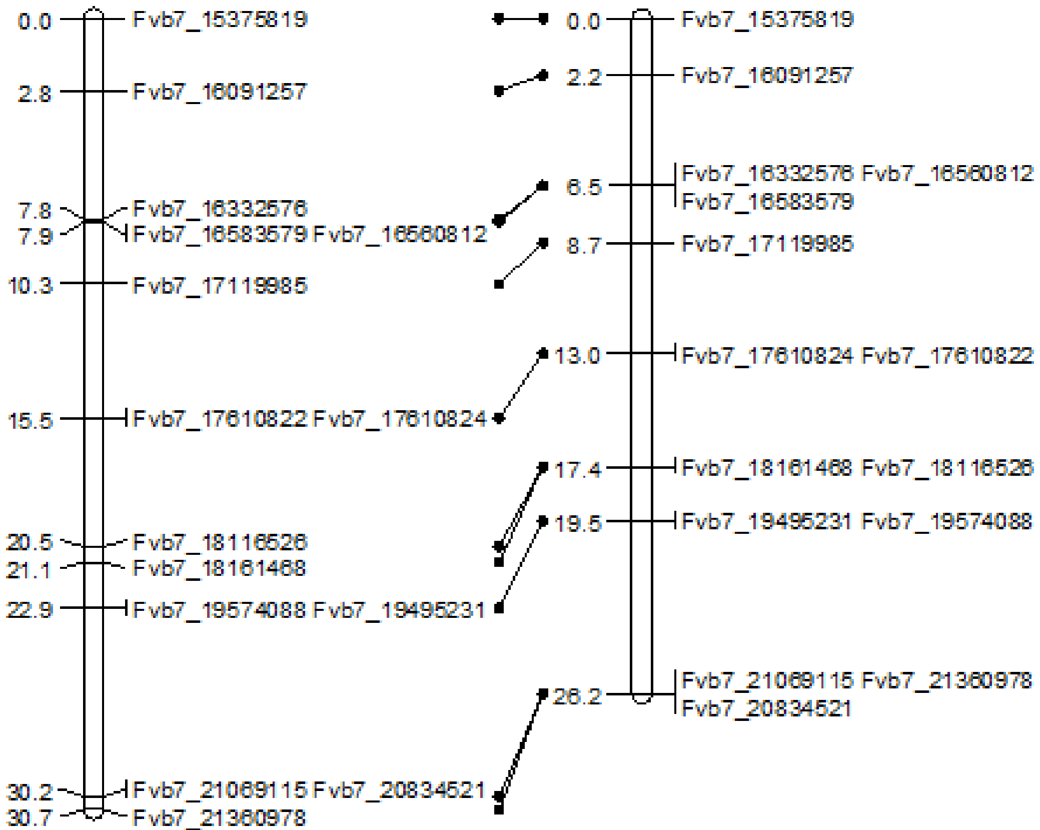

Fvb_7

Redgauntlet_20

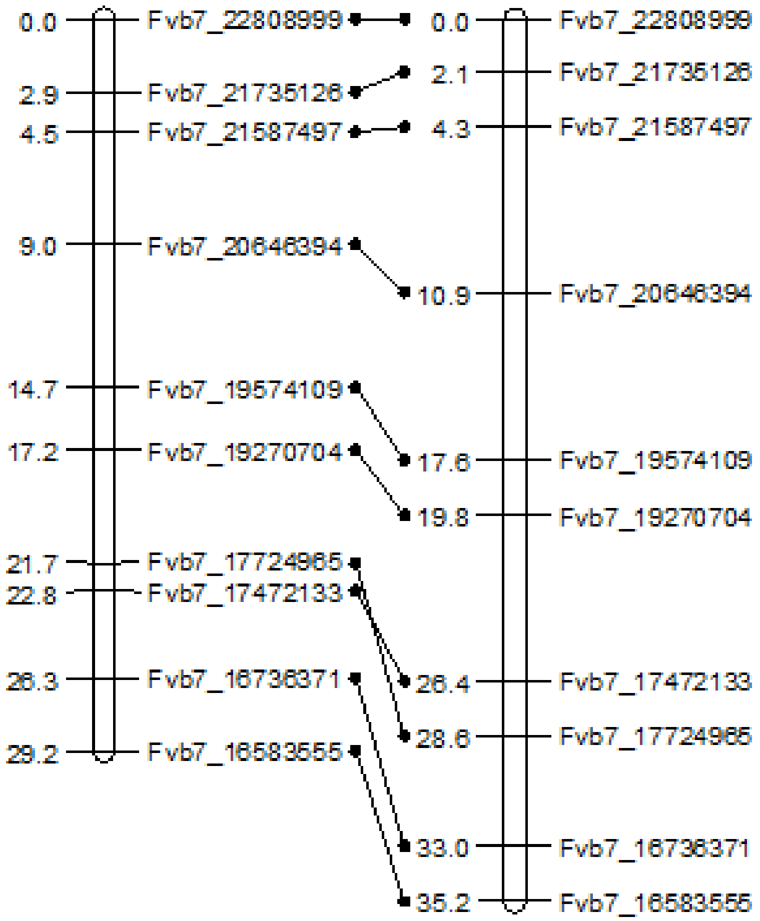

Redgauntlet_25

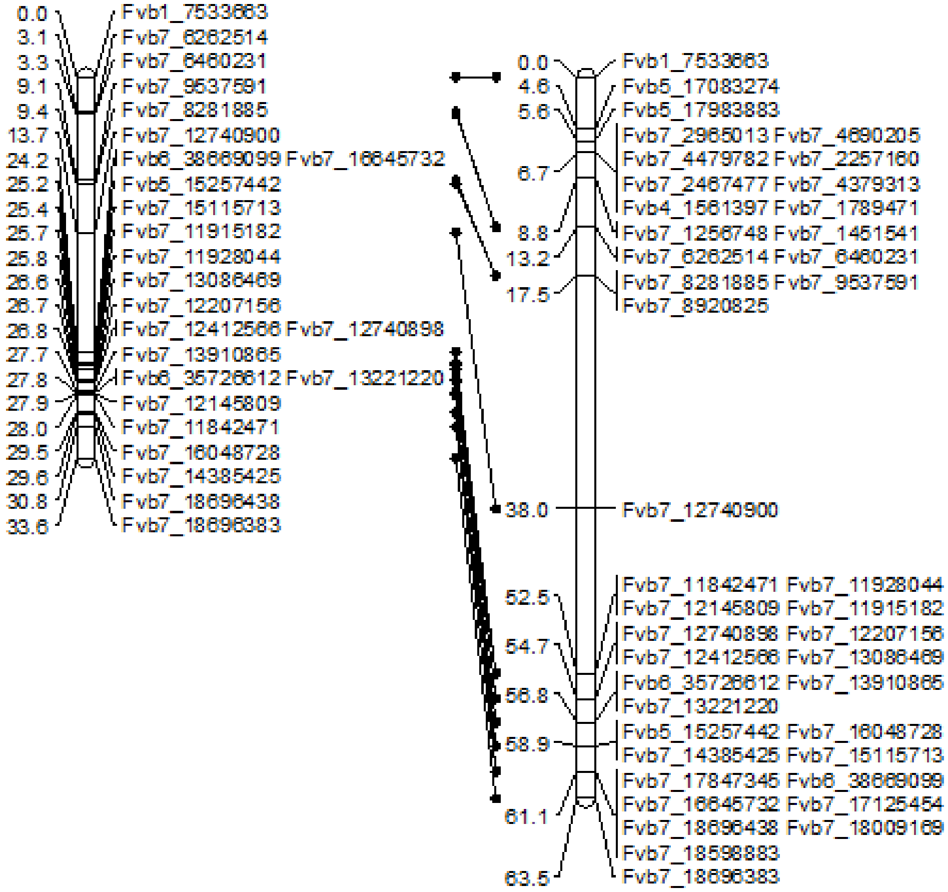

Fvb_7

Redgauntlet_38

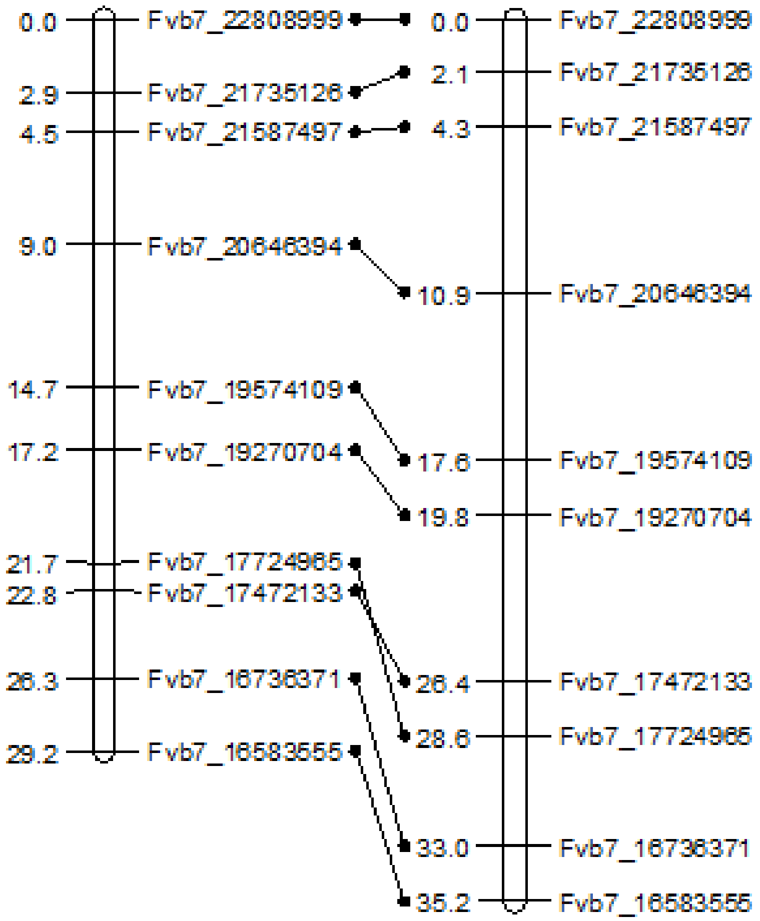

Fvb_7

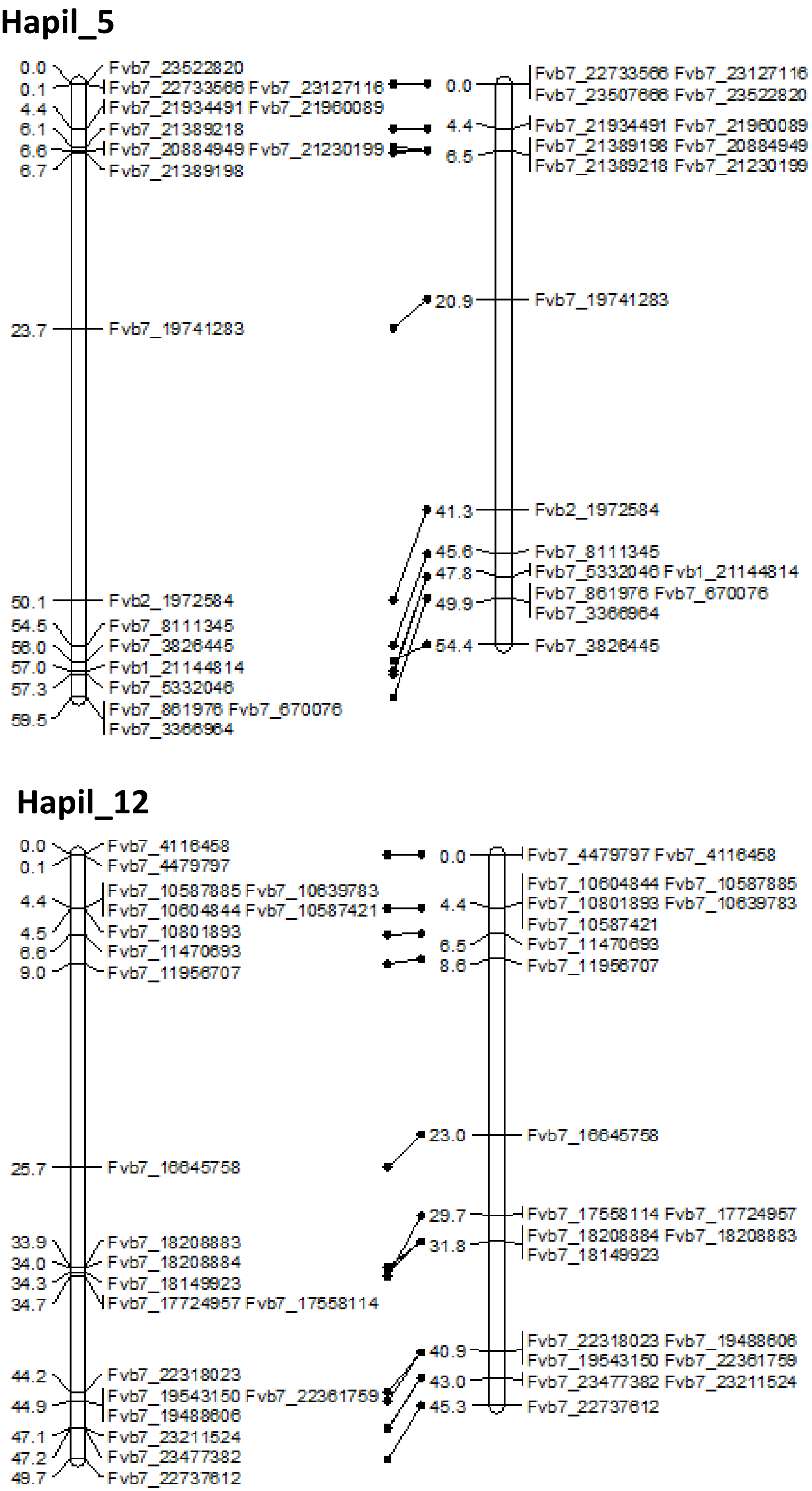
Hapil_5
0.0 Fvb7_23522820
0.1 Fvb7_22733566 Fvb7_23127116
4.4 Fvb7_21934491 Fvb7_21960089
6.1 Fvb7_21389218
6.6 Fvb7_20884949 Fvb7_21230199
6.7 Fvb7_21389198
23.7 Fvb7_19741283
50.1 Fvb2_1972584
54.5 Fvb7_8111345
56.0 Fvb7_3826445
57.0 Fvb1_21144814
57.3 Fvb7_5332046
59.5 Fvb7_861976 Fvb7_670076
Fvb7_3366964
0.0 Fvb7_22733566 Fvb7_23127116
Fvb7_23507666 Fvb7_23522820
4.4 Fvb7_21934491 Fvb7_21960089
6.5 Fvb7_21389198 Fvb7_20884949
Fvb7_21389218 Fvb7_21230199
20.9 Fvb7_19741283
41.3 Fvb2_1972584
45.6 Fvb7_8111345
47.8 Fvb7_5332046 Fvb1_21144814
49.9 Fvb7_861976 Fvb7_670076
Fvb7_3366964
54.4 Fvb7_3826445
Hapil_12
0.0 Fvb7_4116458
0.1 Fvb7_4479797
4.4 Fvb7_10587885 Fvb7_10639783
Fvb7_10604844 Fvb7_10587421
4.5 Fvb7_10801893
6.6 Fvb7_11470693
9.0 Fvb7_11956707
25.7 Fvb7_16645758
33.9 Fvb7_18208883
34.0 Fvb7_18208884
34.3 Fvb7_18149923
34.7 Fvb7_17724957 Fvb7_17558114
44.2 Fvb7_22318023
44.9 Fvb7_19543150 Fvb7_22361759
Fvb7_19488606
47.1 Fvb7_23211524
47.2 Fvb7_23477382
49.7 Fvb7_22737612
0.0 Fvb7_4479797 Fvb7_4116458
4.4 Fvb7_10604844 Fvb7_10587885
Fvb7_10801893 Fvb7_10639783
Fvb7_10587421
6.5 Fvb7_11470693
8.6 Fvb7_11956707
23.0 Fvb7_16645758
29.7 Fvb7_17558114 Fvb7_17724957
31.8 Fvb7_18208884 Fvb7_18208883
Fvb7_18149923
40.9 Fvb7_22318023 Fvb7_19488606
Fvb7_19543150 Fvb7_22361759
43.0 Fvb7_23477382 Fvb7_23211524
45.3 Fvb7_22737612

Fvb_7

Hapil_23

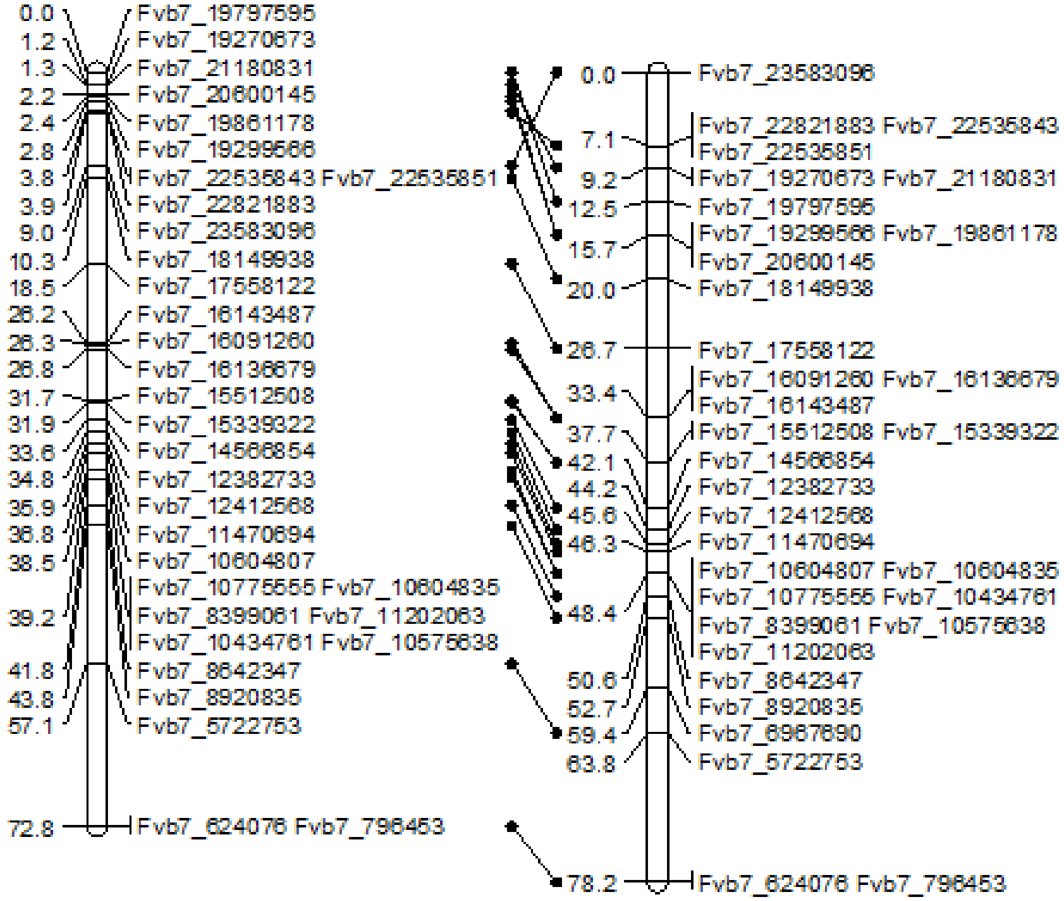

Hapil_32

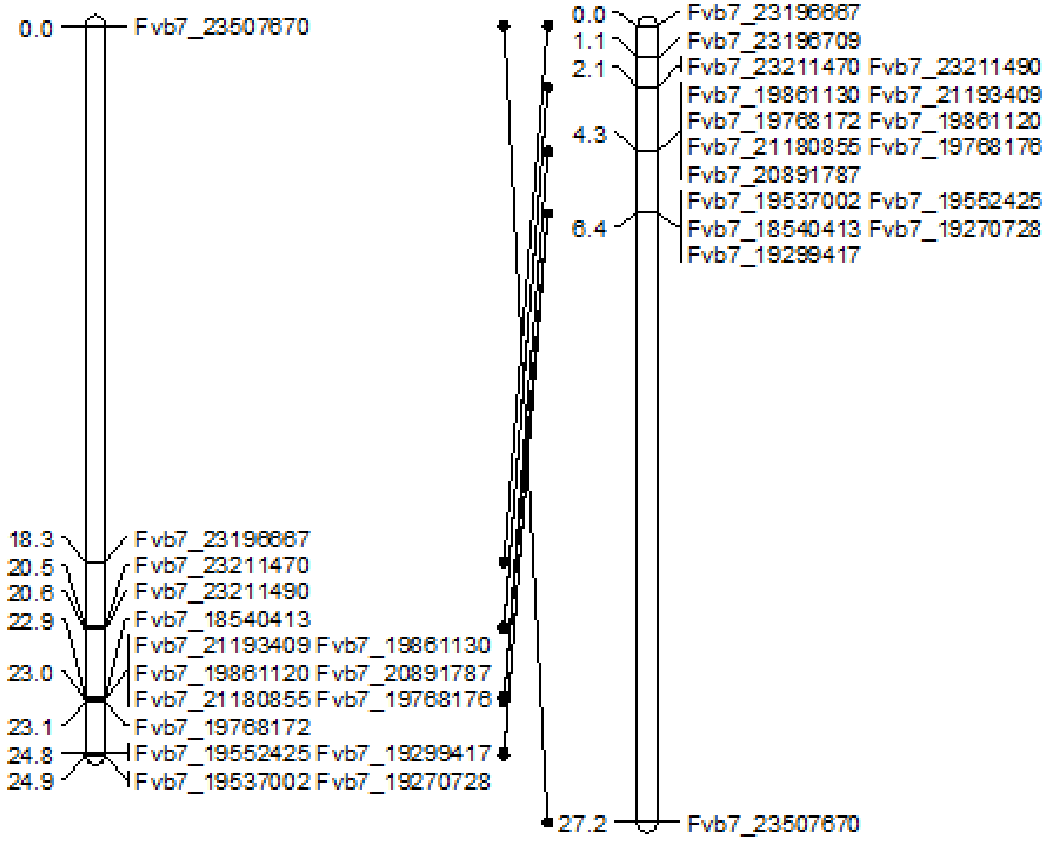

Supplement: File S3 [file peerj-05-3731-s004.pdf]
